# Back to Water: Signature of Adaptive Evolution in Cetacean Mitochondrial tRNAs

**DOI:** 10.1371/journal.pone.0158129

**Published:** 2016-06-23

**Authors:** Stefano Montelli, Antonella Peruffo, Tomaso Patarnello, Bruno Cozzi, Enrico Negrisolo

**Affiliations:** Department of Comparative Biomedicine and Food Science, University of Padova, Legnaro (PD), Italy; Saint Mary's University, CANADA

## Abstract

The mitochondrion is the power plant of the eukaryotic cell, and tRNAs are the fundamental components of its translational machinery. In the present paper, the evolution of mitochondrial tRNAs was investigated in the Cetacea, a clade of Cetartiodactyla that retuned to water and thus had to adapt its metabolism to a different medium than that of its mainland ancestors. Our analysis focussed on identifying the factors that influenced the evolution of Cetacea tRNA double-helix elements, which play a pivotal role in the formation of the secondary and tertiary structures of each tRNA and consequently manipulate the whole translation machinery of the mitochondrion. Our analyses showed that the substitution pathways in the stems of different tRNAs were influenced by various factors, determining a molecular evolution that was unique to each of the 22 tRNAs. Our data suggested that the composition, AT-skew, and GC-skew of the tRNA stems were the main factors influencing the substitution process. In particular, the range of variation and the fluctuation of these parameters affected the fate of single tRNAs. Strong heterogeneity was observed among the different species of Cetacea. Finally, it appears that the evolution of mitochondrial tRNAs was also shaped by the environments in which the Cetacean taxa differentiated. This latter effect was particularly evident in toothed whales that either live in freshwater or are deep divers.

## Introduction

Mitochondrial tRNAs are fundamental components of the translational machinery of the mitochondrion, which is the powerhouse of the eukaryotic cell. In most Metazoa, the mitochondrial genome (mtDNA) contains 22 tRNAs genes (hereafter named *trnX*, where X is the single letter IUPAC code for the corresponding amino acid). For most amino acids, a single tRNA represents the whole codon family. Leucine and Serine are the only known exceptions and possess two tRNAs belonging to two distinct families (i.e., *trnL1* and *tnL2*, and *trnS1* and *trnS2*, respectively). Occasionally, multiple copies of the same tRNA are present in animal mtDNAs. In this latter case, however, they are the product of duplication/multiplication processes and do not represent distinct codon families [1].

TRNAs play a key role in mitochondrial activity; thus, it is plausible that they experienced strong evolutionary constraints, particularly concerning their structural integrity, possibly further reinforced by the fact that there is a single tRNA for most amino acids.

In present paper, the evolution of mitochondrial tRNAs was investigated in the Cetacea, a clade of Cetartiodactyla that returned to the water and consequently adapted its metabolism to an environment different from that of its mainland ancestors [2,3]. Cetaceans breathe air, despite their multiple adaptations to life in water, and therefore represent a very interesting benchmark to test whether this array of adaptations has left any signatures on mitochondrial tRNAs. Cetacea is the most diverse group of current living aquatic mammals and includes 93 species [4]. A complete mtDNA genome is available for 49 species [5–17] (S1 Table). These taxa encompass all families and most of the currently recognised genera, thus providing a very good coverage of the clade.

**Fig 1:**
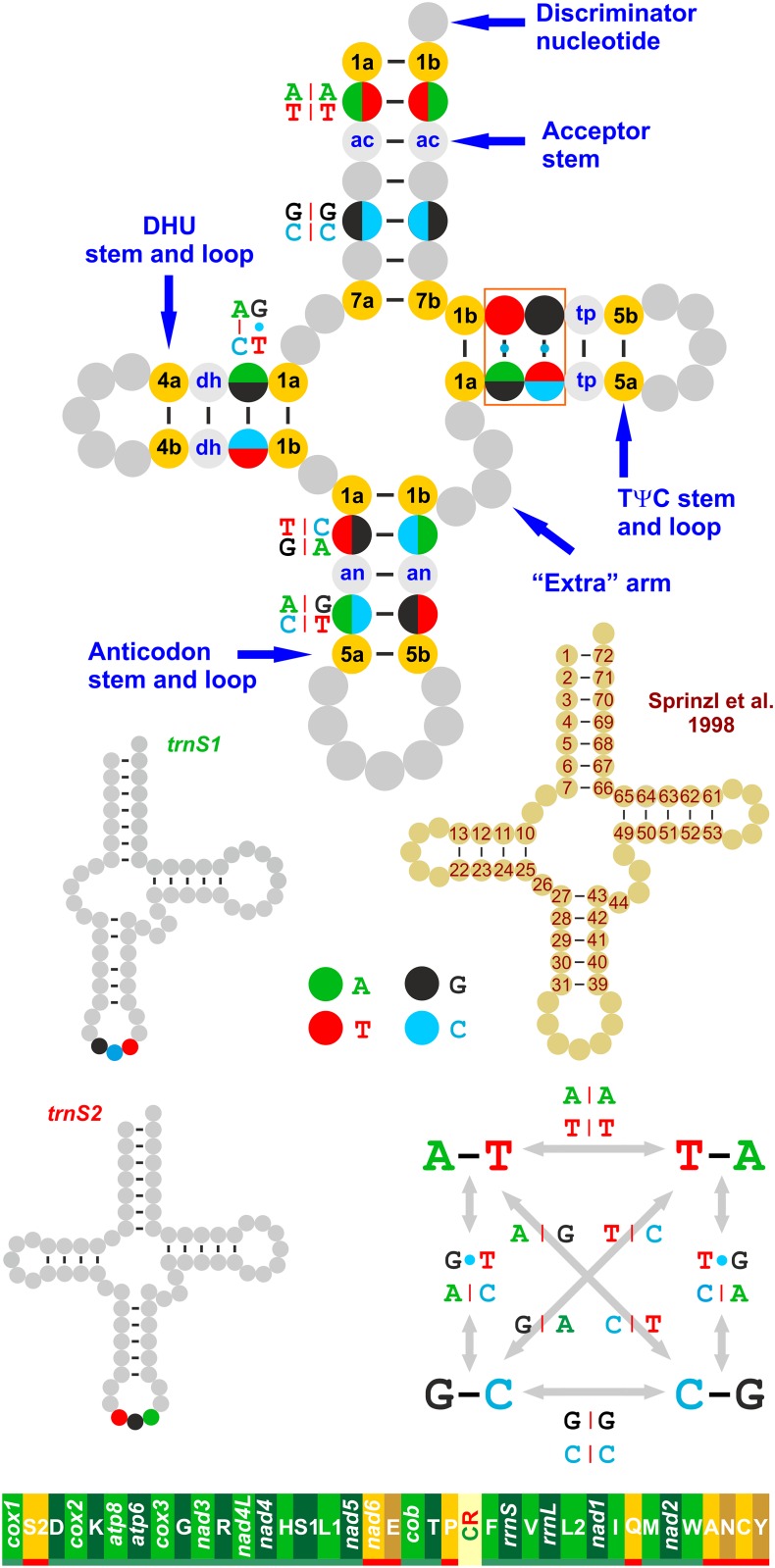
The tRNA nomenclature and the gene order of vertebrate mtDNA. Ac, pair of the acceptor stem; dh, pair of the DHU stem; an, pair of the anticodon stem; tp, pair of the TΨC stem. The numbering of pairs follows a 5’ → 3’ orientation. The 5’ nucleotide of a pair is marked with a, while the 3’ base is marked with b. The numbering scheme of Sprinzl et al. [20] is provided only for the stems. Base pairings are indicated as follows: –, canonical Watson-Crick base pairing; •, base pairing involving G and T; |, base pairing implying a mismatch. A The gene order of vertebrate mtDNA is depicted at the bottom, linearised starting from *cox1*. Genes encoded on the α-strand (right to left orientation) are underlined in green, while those encoded on the β-strand are underlined in red (left to right orientation). Gene nomenclature: *atp6* and *atp8*: ATP synthase subunits 6 and 8; *cob*: apocytochrome b; *cox1*-*3*: cytochrome c oxidase subunits 1–3; *nad1-6* and *nad4L*: NADH dehydrogenase subunits 1–6 and 4L; *rrnS* and *rrnL*: small and large subunit ribosomal RNA (rRNA) genes; and X: transfer RNA (tRNA) genes, where X is the one-letter abbreviation of the corresponding amino acid. In particular, L1 identifies the CTN codon family; L2 the TTR codon family, S1 the AGY codon family, and S2 the TCN codon family. CR, Control Region.

Cetacean tRNAs have a genomic placement matching the canonical gene order of the Vertebrata mtDNA [18] (Fig 1). This means that fourteen mitochondrial tRNAs (i.e., *trnD*, *trnF*, *trnG*, *trnH*, *trnI*, *trnK*, *trnL1*, *trnL2*, *trnM*, *trnR*, *trnS1*, *trnT*, *trnV* and *trnW*) are encoded in the α strand, while the remaining eight (*trnA*, *trnC*, *trnE*, *trnN*, *trnP*, *trnQ*, *trnS2*, and *trnY*) are found in the complementary β strand [18].

Every tRNA exhibits a secondary structure, usually a cloverleaf shape, where double-helix stems are alternated with single-stranded nucleotides (Fig 1). Finally, each tRNA is further arranged in space to generate the functional, L-shaped tertiary structure [19].

The double-stranded elements found in tRNAs are the acceptor stem, the DHU stem, the anticodon stem and the TΨC stem (Fig 1). The acceptor stem is invariably composed by 7 pairs (hereafter 1-7ac-pair) of bases (Fig 1), and the DHU stem contains 3 to 4 pairs (hereafter 1-4dh-pair) and is absent from the *trnS1* of many animal species, including all those studied in this paper. The pairs in the anticodon stem usually number five (hereafter 1-5an-pair), with two exceptions in mammalian *trnS1* and *trnS2*. In both these genes, there is an extra pair located in position 5’ with respect to the standard arrangement that does not have homologous structural counterparts in other tRNAs. This pair has been numbered here as the 0 pair. Finally, the TΨC stem exhibits a variable number of pairs ranging from four to six (hereafter 1-6tp-pair) (Fig 1). A general scheme is available for numbering the placement of every nucleotide in the secondary structure of a tRNA [20] (Fig 1). However, in the present work, we utilised the very simplified system presented in Fig 1, which strictly focused on the pairs found in the different stems. This alternative scheme is, in our view, much easier to follow for the general reader, who not necessarily accustomed to the more sophisticated system devised by Sprinzl et al. [20].

In present paper, the canonical Watson-Crick base pairings will be indicated by the standard dash symbol (–) (e.g., A–T). The base pairings involving G and T will be presented with a dot (•) symbol (e.g., G•T). Finally, the base pairings implying a mismatch (see below) will be described by a vertical dash (|) symbol (e.g., A|A).

Different arrangements can be observed when an identical pair is compared in the orthologous tRNAs of two species A and B. The pair is made by the same couple of bases in both taxa (e.g., A–T, G–C, and the opposite), or the pair is differently arranged in the two species. In this latter case, there are three possibilities (1–3). (1) With respect to species A, the B taxon exhibits a simple/double mismatch in the pair (e.g., A|A vs. A–T; C|C vs. A–T). In this case, the substitution/s pattern leads to a disruption of the secondary structure of the stem for that pair. Mismatches that do not prevent the formation of the cloverleaf structure or the tertiary structure are not rare in tRNAs. Mismatches can be corrected through editing processes or can persist in the tRNA stems as unusual pairings [21]. (2) With respect to species A, the B taxon exhibits the substitution of a single base of the pair (this can arise at the 5’ end as well as the 3’ end of the pair), which does not alter the secondary structure of the stem (e.g., G•T vs. A–T). This type of change is named hemi-compensatory base change (HCBC) [22] because its occurrence does not alter the secondary structure itself (Fig 1) [21,23,24]. (3) Finally, species B exhibits a couple of complementary bases different than species A (e.g., A–T vs. G–C). In this case, a fully compensatory base change (FCBC) occurs in species B with respect to A. This change is named fully compensatory because the substitution of both bases does not jeopardise the secondary structure integrity [22]. Two types of FCBCs exist: one (hereafter named type I) implies the substitution of a purine-pyrimidine pair with another purine-pyrimidine couple and vice versa, and the other (hereafter named type II) is characterised by a purine-pyrimidine vs. pyrimidine-purine substitution. The occurrence of type I is favoured over that of type II because type I can be produced through an intermediated HCBC. Conversely, type II requires passage through a mismatch step.

Our paper mainly focuses on identifying the factors that influenced the evolution of the elements of the tRNA double helix of Cetacea, which play a pivotal role in the formation of the secondary and tertiary structure of each tRNA and consequently modify the whole translation machinery of the mitochondrion. FCBCs, HFBCs and mismatches are globally indicated in this paper with the acronym CSBPSs (Change of Sequence in a Base Pair of a Stem).

## Materials and Methods

### Sequencing of the mtDNA of *Ziphius cavirostris* G. Cuvier, 1823

For the present study, we sequenced the complete mitochondrial genome of a specimen of *Z*. *cavirostris*. The striated muscle tissue (approximately 0.5 g) used as the starting material to extract the total DNA was obtained from a female specimen of *Z*. *cavirostris* that had been stored since 2007 at -80°C at the *Mediterranean marine mammal tissue bank* (MMMTB, www.marinemammals.eu) of the University of Padova (specimen # ID 135). MMMTB is a non-profit public organisation that preserves for scientific purposes the tissues of Cetacean specimens that beached and died naturally along the Italian Coasts. MMMTB promotes the study and conservation of Cetacea. MMMTB is officially supported by the Italian Ministry of Environment and is CITES credited. The scientific study of tissues obtained from MMMTB does not require the approval of an ethical committee.

The extraction was performed through a salting-out protocol [25]. The amplification and sequencing of mitochondrial DNA were performed using a mixture of mammalian universal primers [26] and primers specifically designed against available sequences belonging to the family Ziphiidae. The quality of DNA was assessed through electrophoresis in a 1% agarose gel. The PCR products were directly sequenced using the primers used for amplification. The sequencing was performed by BMR Genomics (http://www.bmr-genomics.it/; Padua, Italy). Both strands of PCR products were sequenced to ensure the standard accuracy required by this type of sequencing activity. The mtDNA consensus sequence was assembled using the SeqMan II program from the Lasergene software package (DNAStar, Madison, WI). The coverage of the whole consensus sequence was at minimum 2X and in most cases 3X to 4X. The genome was annotated following the strategy briefly described below [27,28].

Initially, the mtDNA sequence was translated into putative proteins using the Transeq program available on the EBI website. The true identity of these polypeptides was established using the BLAST program [29,30]). The boundaries of genes were determined as follows. The 5' ends of protein-coding genes (PCGs) were defined as the first legitimate in-frame start codon (ATN, GTG, TTG, GTT) in the open reading frame (ORF) that was not located within an upstream gene encoded on the same strand. The only exceptions were *atp6* and *nad4*, which were previously demonstrated to overlap with their upstream gene i.e., *atp8* and *nad4L*, respectively, in many mtDNAs [31]. The PCG terminus was defined as the first in-frame stop codon that was encountered. When the stop codon was located within the sequence of a downstream gene encoded on the same strand, a truncated stop codon (T or TA) adjacent to the beginning of the downstream gene was designated as the termination codon. This codon was thought to be completed by polyadenylation, thereby producing a complete TAA stop codon after transcript processing. Finally, pairwise comparisons with orthologous proteins were performed using the ClustalW program [32] to better define the limits of the PCGs.

Regardless of the real initiation codon, formyl-Met was assumed to be the starting amino acid for all proteins as has been previously demonstrated in other mitochondrial genomes [33,34]).

Transfer RNA genes were identified using the tRNAscan-SE program [35] or recognised manually as sequences having the appropriate anticodon and capable of folding into the typical cloverleaf secondary structure of tRNAs [31]. The validity of these predictions was further enhanced by comparison based on multiple alignment and structural information to published orthologous counterparts [36].

The boundaries of the ribosomal *rrnS* and *rrnL* genes were those defined by the pairs of tRNAs adjacent upstream/downstream to these genes (i.e., *trnF* and *trnV* for *rrnS*; *trnV* and *trnL2* for *rrnL*).

### Dataset construction

At least one complete mtDNA sequence for 49 cetacean species is currently available in GenBank (2015.09.30 release) (S1 Table). For some taxa, multiple sequences are available (e.g., *Physeter macrocephalus*). To ensure a balanced treatment of the different species, only one mtDNA sequence was included in the main dataset (see below). The only exception was *Orcinus orca*. For the killer whale, seven sequences were included, each representing one of the main clades recently identified within this taxon [13,37,38] that are possibly/probably distinct cryptic species; see de Bruyn et al. [39] for a different view. Before creating the datasets, both ingroup and outgroup mtDNA sequences were manually re-annotated to have high-quality annotated genomes. This was fundamental for identifying the correct boundaries of tRNAs. The analyses performed on tRNA evolution were very time-consuming; thus, the sequences of *Mesoplodon grayi*, *Mesoplodon ginkgodens* and *Neophocaena asiaeorientalis* became available too late to be fully implemented in our study. However, the sequences of *M*. *grayi and N*. *asiaeorientalis* were considered in some analyses (see the Results section). The complete reference dataset contained 94 taxa (listed as 94T-set in the paper). 94T-set included 46 cetaceans plus a broad selection of the main Artiodactyla lineages and two Perissodactyla. A list of taxa that were analysed in this paper is provided in S1 Table. The taxonomy of Cetacea used in the present paper follows that of Perrin [4]. The status of *Tursiops australis* as a distinct species is under scrutiny [4], but it was retained here provided that multiple mtDNA sequences exist for this taxon and were worthy of consideration, irrespective of the taxonomic validity of this species.

### Multiple alignments of orthologous genes

Initially, each set of the 13 orthologous protein-coding genes derived from 94T-set was aligned using the pipeline implemented in the TranslatorX server [40]. This webtool ensures that the alignment of DNA sequences is obtained using as a backbone the multiple alignment derived from the amino acid counterparts. The MAFFT program was used to produce the alignments [41,42]). Successively, the Gblocks program (with the most stringent parameters activated) was used to the select the most conserved positions of the alignments [43]. Finally, the 13 Gblocks-processed nucleotide alignments were concatenated into a single multiple alignment (94T.13PCG.set).

The sequences of the orthologous tRNAs obtained from 94T-set were manually aligned considering the secondary structures predicted with tRNA-scan or that were available in the literature (see S1 tRNAs multiple alignments) [36]. The same strategy was applied to produce multiple alignments necessary to investigate the intraspecific variation of every tRNA for the species of cetaceans for which several/many mtDNA sequences exist. In the case of the 94T-trnXs alignment, it was not possible to model the substitution process for the most variable portions located in the DHU and TΨC loops of some tRNAs. In contrast, it was always possible to model the substitution process within the Cetacea clade.

Irrespective of the strategy used to obtain the multiple alignments, these alignments were successively imported into MEGA 5.2.2 [44] for further bioinformatic analyses.

### Statistics of DNA/amino acid sequences

The AT-skew = (A-T)/(A+T) and the GC-skew = (G-C)/(G+C) were computed for the α strand of the full-length mtDNA of all 94 analysed taxa to evaluate the compositional biases [45]. The base compositions were determined with the EditSeq program from the Lasergene software package (DNAStar, Madison, WI).

The evaluation of the level of saturation in the DNA/amino acid substitution process was assessed for 94T.13PCG.set as well as for the orthologous tRNAs. In the case of 94T.13PCG.set, pairwise distances were calculated separately for whole codons, first + second positions, first positions, second positions, third positions and amino acids.

Initially, the p-distance and the maximum composite likelihood distance were calculated for each pairwise-comparison. Then, the (maximum composite likelihood distance—p-distance) difference was calculated for every pairwise comparison as a measure of the underestimation of the number of substitutions that is obtained through the p-distance. Indeed, the p-distance does not correct for possible multiple substitution events at a single position of the alignment. With no or minimal saturation, the (maximum composite likelihood distance—p-distance) difference is null or very small. In contrast, it exceeded the unit when the saturation process progressed sensibly. The (maximum composite likelihood distance—p-distance) difference was used instead of the more traditional (p-distance / maximum composite likelihood distance) ratio [46] because it allows for the calculation to be performed automatically on thousands of pairwise comparisons in a spreadsheet without the necessity of eliminating null maximum composite likelihood distances. Finally, the average (maximum composite likelihood distance—p-distance) difference was used as a global descriptor of saturation of the substitution process for every set of orthologous tRNAs.

The distances were computed with MEGA 5.2.2 [44]. The computations of the skews as well as other statistical calculations were performed using Microsoft Excel (Microsoft ^™^).

The total number of codons present in the whole set of Cetacea PCGs was calculated with the MEGA program. Stop codons were excluded from the calculation because they are not linked to a tRNA family. Analogously, start codons were not considered because different codons determine the same formyl-Met as the starting amino acid [33,34]. Finally, the total number of codons belonging to each codon family was calculated, and the abundance of each codon family was expressed as the number of codons per thousand codons.

### Phylogenetic analyses and the reference tree

Maximum likelihood phylogenetic analyses [47] were performed using the program RAxML 7.4.2 [48] implemented in the graphical user interface raxmlGUI 1.3.1 [49]. A nonparametric bootstrap test [50] was performed to assess the robustness of the topologies (1,000 replicates). Phylogenetic analyses were performed on nucleotide/amino acid datasets exhibiting the highest phylogenetic signals. In the case of DNA datasets, the GTR evolutionary model [51] was applied, while the heterogeneity of the substitution process was modelled with the CAT [52]. In the case of amino acid datasets, the MTMAM substitution matrix [53] was used in combination with the CAT algorithm. Partitioning schemes were used to test their effect on the tree topologies.

Phylogenetic analyses were performed on the position 2, positions 1+2, and amino-acid subsets of 94T.13PCG.set, which exhibited the highest signals, with and without partitions.

All of the obtained trees were identical to the topology depicted in S1 Fig. In the topology, most of the nodes received bootstrap support. The tree in S1 Fig was generated from the amino acid dataset. The topology revealed that many amino acids changed along the branch reaching the root of Cetacea. The mysticete *Caperea marginata* and, more markedly, the odontocetes *Kogia breviceps*, *P*. *macrocephalus*, *Platanista minor*, *Lipotes vexillifer*, *Pontoporia blainvillei*, *Inia geoffrensis*, and *Monodon monoceros* showed branches that were decidedly longer that those of other Cetacean species. No further details are presented here on the phylogeny of Cetacea. A comment must be introduced to explain this point. The phylogeny of Cetacea is a very active field of study, and several papers have been published on this topic [12,14,15,54–64]. The overall phylogenetic relationships among major lineages were consistently recovered in the studies mentioned above and are depicted in S1 Fig. In contrast, the vast majority of the published trees exhibit one or more points of disagreement. In the present paper, the topology of S1 Fig was used as a reference tree to map the evolution of CSBPSs. Alternative phylogenetic relationships were considered to test whether they could produce relevant changes in our results (data not shown). These topologies gave, at most, marginal variations restricted to single nodes and did not alter the global evolutionary pathway for the CSBPSs. Thus, they will not be described in detail in the present paper.

### Tracking the substitution patterns of orthologous tRNAs along the reference tree

The CSBPSs occurring in the multiple alignments of orthologous tRNAs were tracked along a reference tree according to the maximum likelihood method available in MEGA 5.2.2 [44] and according to the maximum parsimony approach implemented in the Mesquite program [65]. In the latter, the nucleotide changes were assumed to be unordered events. The mismatches occurring at the boundaries between DHU and TΨC arms and loops were not considered. This choice was dictated by the fact that in some cases, the length of the arms was variable without disrupting the secondary structure (S1 tRNAs Multiple Alignments).

## Results

Introductory note. Here, only the main results are provided. A more detailed description is presented in S1 Extended Results.

### The mitochondrial genome of *Ziphius cavirostris*

The mtDNA of a specimen of *Z*. *cavirostris*, sequenced for this paper, is briefly described here. The new mitochondrial genome was 16,352 bp long. This value was very close to the average value obtained for the dataset analysed in the present work (16,436 ± 124). The *Z*. *cavirostris* genome contained the 37 genes almost universally found in animal mtDNAs i.e., 13 PCGs, two ribosomal rRNAs and 22 tRNAs. The gene order was typical for vertebrate mtDNAs (Fig 1), with 28 genes encoded on the α-strand and nine present on the opposite β-strand. Most of the PCGs started with ATG and ended with TAA or the incomplete stop codons TA(a) and T(aa). The genes on the same/opposite strand overlapped, were contiguous or were separated by intergenic spacers encompassing a variable number of nucleotides (S2 Fig). The mtDNA sequence of *Z*. *cavirostris* is available in EBI/GenBank under accession number LN997430.

### Occurrence of CSBPSs in the tRNAs of Cetacea

The computation of the p-distance and (maximum composite likelihood distance—p-distance) difference allowed the level of conservation and the possible underestimation of the substitution patterns in Cetacea tRNAs (S3 and S4 Figs, respectively) to be determined. The most conserved tRNA was *trnG*, and the most variable was *trnH*. The minimum and maximum values for the (maximum composite likelihood distance—p-distance) difference were observed in *trnE* and *trnH*, respectively. The (maximum composite likelihood distance—p-distance) difference values demonstrated that the observed CSBPSs did not grossly underestimate the true number of CSBPSs in the tRNAs.

**Fig 2:**
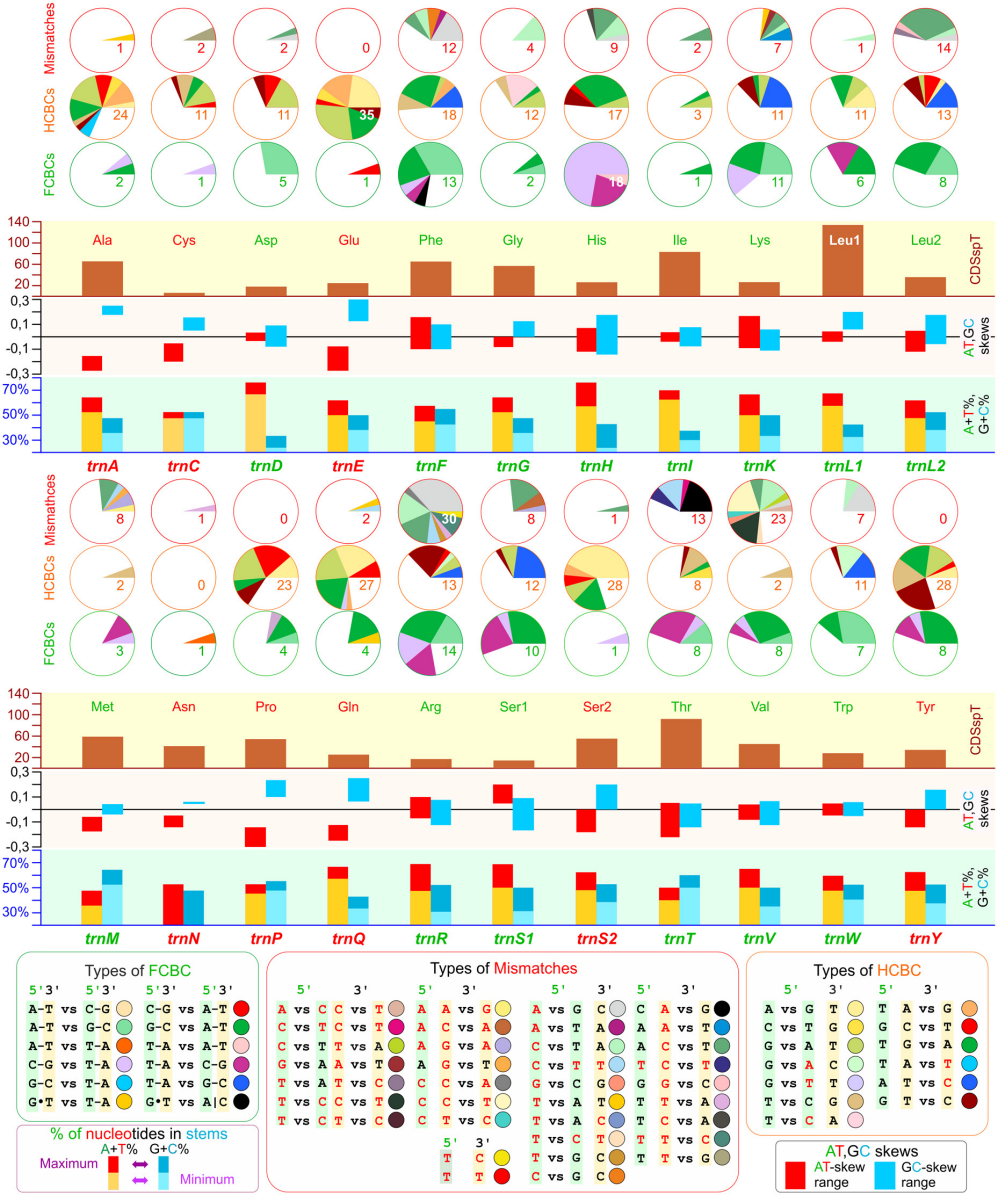
CSBPSs, skews, stems composition, and codon usage linked to the Cetacea tRNAs. CSBPS, change in sequence in a base pair of a stem; FCBC, fully compensatory base change; HCBC, hemi compensatory base change; Mismatch, mismatch in a base pair of a stem. The extension of the slices determining the number and type of FCBCs, HCBCs, and Mismatches for every tRNA was scaled, assuming that the whole coverage of a Pie graph was reached only in the tRNA exhibiting the maximum number of in CSBPSs. This approach allows, in our view, to better appreciate the variation of CSBPSs in the various tRNAs. CDSpT, codons per thousand codons associated to a tRNA. A+T%, percentage of A+T in the stems; G+C%, percentage of G+C in the stems. For A+T% and G+C%, the maximum and minimum values are provided. AT- and GC-skews, skews calculated for the stems of tRNAs. For AT- and GC-skews, the range of variation is provided for every tRNA.

A total of 603 CSBPSs (136 FCBCs, 320 HCBCs, and 147 mismatches) were identified in the tRNAs of Cetacea (Fig 2; S1 tRNA Multiple Alignments).

Most of the FCBCs were of type I (131), and only four were of type II. T–A vs. A–T occurred on the 5an-pair of *trnH*. G–T vs. T–A was found on the 7ac-pair of *trnQ*. C–G vs. A–T was identified on the 2tp-pair of *trnE*. A–T vs. T–A was detected on the 2tp-pair of *trnN* (Fig 2; S1 tRNA Multiple Alignments).

The number of FCBCs detected in a single tRNA ranged from 1 to 18 (Figs 1 and 2). Ten or more FCBCs were found only in α-strand-encoded tRNAs (Figs 1 and 2). Most of the β-strand tRNAs exhibited four or fewer FCBCs. The bases alternating on the type I FCBCs followed three patterns (a-c). (a) Only purines alternated at the 5’ end of the pair, and only pyrimidines occurred at the 3’ end (e.g., *trnD*). (b) Both purine/pyrimidine bases occurred in the substitution at the 5’ and 3’ ends of the pair (e.g., *trnA*) with a variable prevalence of the first/second type of base. (c) Only pyrimidines alternated at the 5’ end, and only purines occurred at the 3’ end of the pair (e.g., *trnC*) (S1 tRNA Multiple Alignments).

The 320 HCBCs were distributed mainly into four symmetrical types (Fig 2; S1 Extended Results). The number of HCBCs in a single tRNA ranged from 0 (*trnN*) to 35 in (*trnE*). The distribution of HCBCs was β-strand biased. Indeed, the α-strand tRNAs showed 144 HCBCs, while the β-strand tRNAs exhibited 176 HCBCs (Fig 2).

The 147 mismatches belonged to 35 different types (Fig 2; S1 Extended Results) and ranged from 0 (e.g., *trnP*) to 30 (*trnR*). The mismatches exhibited an uneven and α-strand-biased distribution (Fig 2, S1 Extended Results).

### Patterns of CSBPS distribution in Cetacea tRNAs

The distribution of CSBPSs was very variable in the tRNAs (Fig 2; S1 Extended Results). FCBCs, HCBCs, and mismatches were linked in their abundance (≥ 7) in several α-strand tRNAs (Fig 2). A second patter, mainly observed in β-strand tRNAs, had a high number of HCBCs coupled with a low number of FCBCs and mismatches. A low number of mismatches coupled with a moderate number of FCBCs and a higher number of HCBCs was present in *trnD*, *trnL1*, and *trnY*. A small number of base changes characterised *trnI* and *trnN*. Few FCBCs and HCBCs and a good number of mismatches occurred in *trnM*. Finally, *trnV* exhibited a high number of mismatches coupled with a good number of FCBCs and a low number of HCBCs (Fig 2; S1 tRNAs multiple alignments).

### Factors influencing the occurrence and type of CSBPSs

The total number of codons encoded by the cetacean mtDNAs as well the number of codons for each codon family were homogenous (Fig 2; S1 Extended Results). The number of FCBCs, HCBCs, and mismatches were not linked to the abundance of codon families (Fig 2; S1 Extended Results).

Globally, the occurrence of CSBPSs in different tRNAs was influenced by the combined action of (a) the base content variation and (b) the asymmetrical compositional biases of the stems. These latter in several cases were opposite to the values computed for the strand encoding the analysed tRNAs. In particular, the range of variation and the fluctuation of base content, AT- and GC-skews, had a major impact on the type and abundance of CSBPSs (Fig 2; S1 Extended Results; S5 and S6 Figs).

Finally, the abundance of FCBCs, HCBCs, and mismatches did not appear to be linked to the genomic placement of different tRNAs (Figs 1 and 2). A couple of examples support this statement. *TrnA* and *trnN*, both on the β-strand and adjacent, exhibited very different behaviours. In contrast, *trnR* and *trnT*, both on the α-strand and well separated, had very similar patterns.

### The stem positions associated with CSBPSs

**Fig 3:**
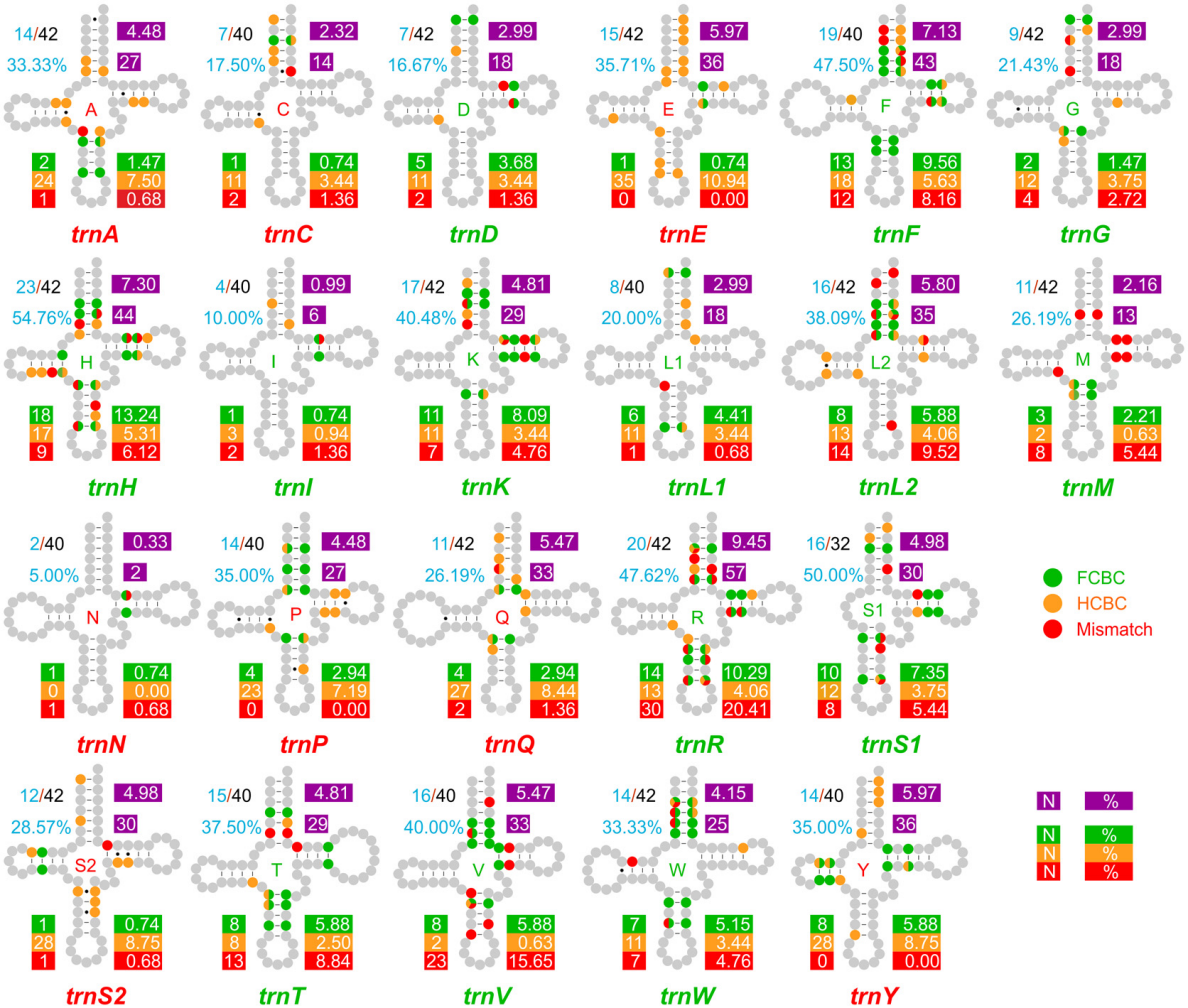
Distribution of CSBPSs in a single tRNA. CSBPS, change in sequence in a base pair of a stem; FCBC, fully compensatory base change; HCBC, hemi compensatory base change; Mismatch, mismatch in a base pair of a stem; SPIC, stem position involved in base change. The number/percentage of SPICs is provided in cyan. A green background is used to mark the number of FCBCs occurring in a tRNA as well as the percentage that they represent with respect to the total number of FCBCs. The orange and red backgrounds are used for the number/percentage of HCBCs and Mismatches. A purple background is used for number/percentage of CSBPSs.

**Fig 4:**
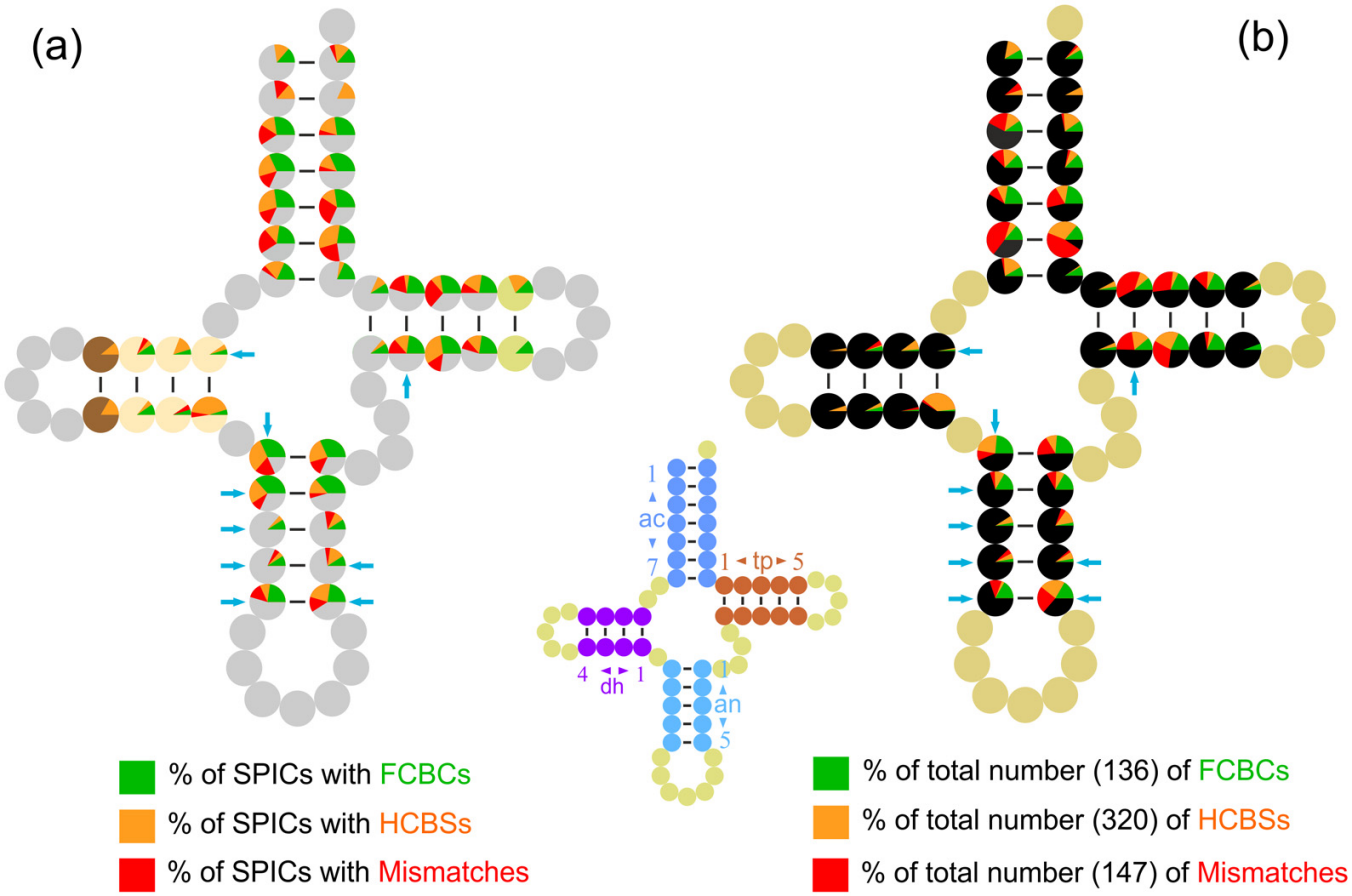
Global distribution of SPICs and CSBPSs in cetacean tRNAs. CSBPS, change in sequence in a base pair of a stem; FCBC, fully compensatory base change; HCBC, hemi compensatory base change; Mismatch, mismatch in a base pair of a stem; SPIC, stem position involved in base change. (a) Percent of SPICs in the whole set of tRNAs involved in CSBPSs. The extension of the slices determining the percent and type of FCBCs, HCBCs, and Mismatches was scaled assuming that the whole coverage of a Pie graph was reached only when the considered position resulted in an SPIC for the entire set of tRNAs. (b) Percent of CSBPSs occurring in a considered SPIC with respect to the total number of CSBPSs. The cyan arrows point to stem positions known to be interested in species of Mammalia by an editing activity in at least one tRNA.

The stem positions involved in base changes (hereafter named SPICs) were mapped and analysed in the different tRNAs (Figs 3 and 4). One, two and even all three types of substitutions were observed in the same SPIC (e.g., *trnF*, Fig 3). The number of SPICs was very variable within the 22 tRNAs (Fig 3). Due to the heterogeneity of the substitution patterns, a perfect correspondence did not exist between the percentage of SPICs and the global percentage of CSBPSs occurring in a single tRNA. Despite these vagaries, the percentage of SPICs was in good agreement with the global percentage of CSBPSs. The percentages of SPICs and FCBCs exhibited similar behaviours. Many more discrepancies existed among the percentage of SPICs and the global percentages of HCBCs and mismatches (S1 Extended Results). Each tRNA exhibited a unique pattern of SPICs and associated types of FCBCs, HCBCs, and mismatches.

The distributions of SPICs and CSBPSs are summarised in Fig 4. The occurrence of FCBCs was very variable in the pairs. Some pairs (e.g., 2ac-pair) never presented an FCBC (Fig 4a). In contrast, other pairs (e.g., 4ac-pair) were hot spots for the occurrence of FCBCs. In general, the acceptor, the anticodon, and the TΨC stems contained most of the SPICs associated with FCBCs. The DHU stem had a very limited number of SPICs associated with FCBCs (S1 Extended Results).

The SPICs associated with HCBCs were variably distributed in the different tRNAs (Fig 4a; S1 Extended Results). Some positions were heavily involved with HCBCs (e.g., the 5’ end of dh1-pair), while others never exhibited an HCBC. Furthermore, the 5’ end and 3’ end could behave differently in the same pair (S1 Extended Results).

The SPICs associated with mismatches were more abundant on acceptor and TΨC stems. Additionally, the anticodon stem presented several SPICs linked to mismatches. Very few SPICs hosting mismatches occurred in the DHU stem. Mismatches were never detected in some positions (Fig 4a). When the global percentage of FCBCs, HCBCs, and mismatches occurring at the different SPICs was evaluated, the patterns that emerged largely mirrored the abundance of SPICs described above (Fig 4b).

The occurrence of HCBCs and of mismatches exhibited an evident 5’ end or 3’ end distributional bias in the pairs of some tRNAs (Fig 3; S8 and S9 Figs; S1 Extended Results).

Finally, the known distribution of positions in the stems, where post-transcriptional modifications occur, was mapped and compared to the SPIC behaviour (Fig 4). A simple pattern linking these positions with CSBPSs/SPICs was not identified.

### Phylogenetic distribution of CSBPSs

**Fig 5:**
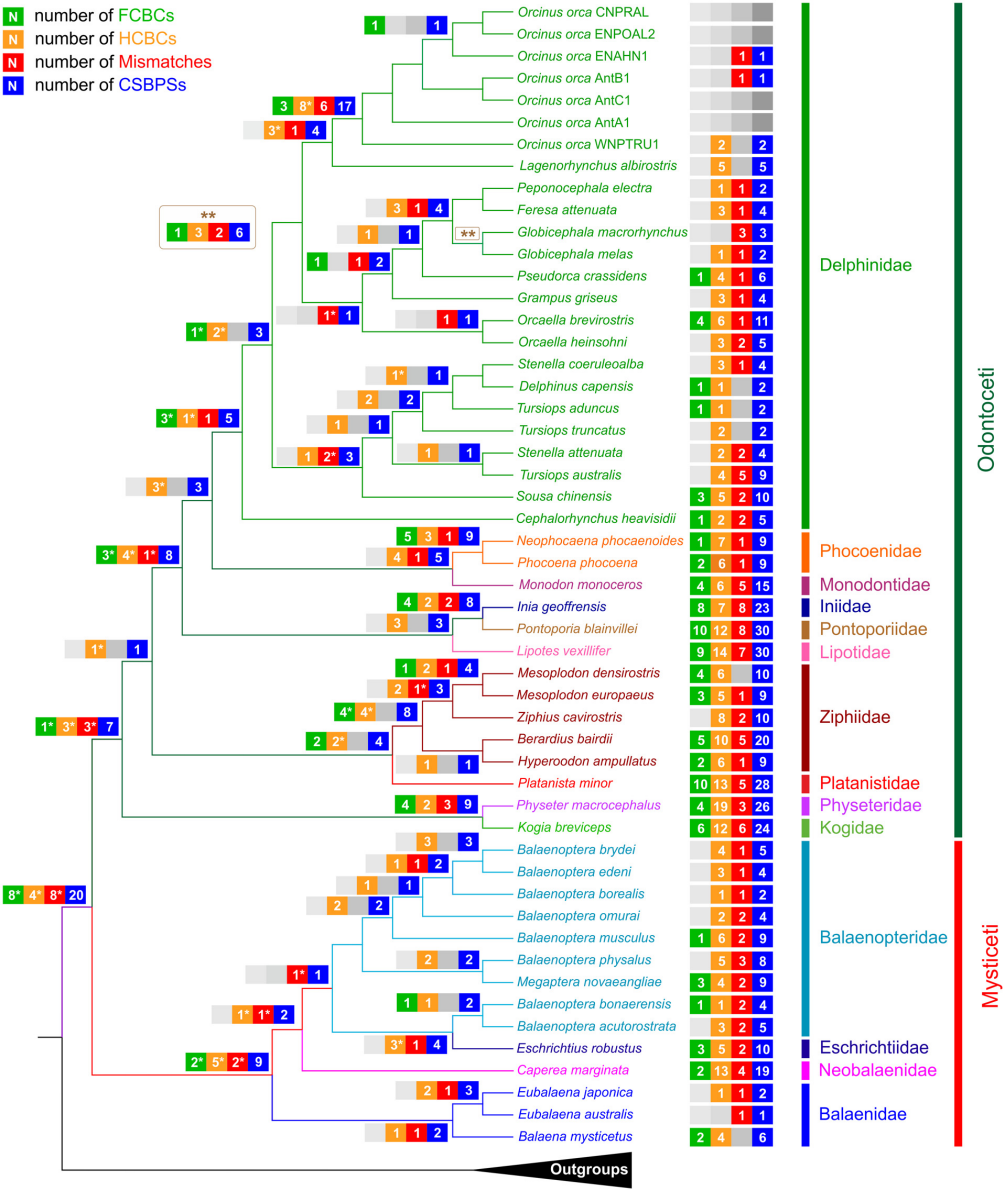
Mapping of CSBPSs in the Cetacea phylogenetic tree. CSBPS, change in sequence in a base pair of a stem; FCBC, fully compensatory base change; HCBC, hemi compensatory base change; Mismatch, mismatch in a base pair of a stem; Number of FCBCs, HCBCs, and Mismatches (included their sum CSBPSs) occurring at the nodes of the reference phylogenetic tree. The asterisk associated with some CSBPS values indicates that these CSBPSs were subjected to successive changes in one/some of the taxa downstream of the considered node. For details on the type of FCBCs, HCBCs, and mismatches occurring at a single node, please refer to S7–S9 Figs.

The distribution of CSBPSs along the reference tree is summarised in Fig 5, while the full details are provided in S7–S9 Figs.

A total of 70.32% of the CSBPSs were associated with living species of Cetacea, while the remaining 29.68% were divided among the internal nodes of the tree. In living species, the percentage of FCBCs was 66.91%, that of HCBCs was 72.19%, and that of mismatches was 69.39% (Fig 5).

The four FCBCs of type II that were identified in Cetacea (see above) had a variable taxonomic distribution. Two were restricted to a single species i.e., *K*. *breviceps* (*trnN*), and *I*. *geoffrensis* (*trnE*) (S1 tRNA Multiple Alignments). The FCBC occurring in *trnH* appeared at the onset of Cetacea. During the cladogenetic process, successive FCBCs of type I, HCBCs and a mismatch were substituted for this CSBPS in some cetacean species (S7–S9 Figs, S1 tRNAs Multiple Alignments, *trnH*). Finally, the FCBC of type II found in *trnQ* characterised most of Odontoceti (except for *P*. *macrocephalus* + *K*. *breviceps*) and was followed by successive HCBCs.

The analysis of the distribution of the CSBPSs revealed the occurrence of a dynamic, continuous, and ongoing evolutionary mechanism of changes in the stems of tRNAs. The oldest CSBPSs were followed by successive changes (marked with an asterisk in Fig 5) that occurred in descendant groups at different taxonomic ranks (S7–S9 Figs). The CSBPSs were in several cases molecular signatures for the different clades (S1 Extended Results). In contrast, in other cases, the CSBPSs represented events of convergent/parallel evolution (S1 Extended Results). Finally, the substitution pattern produced in some cases a secondary reversion to the condition observed in outgroups due to the limited possibility of combinations of the four bases (S1 Extended Results).

The distribution of CSBPSs in living Cetacea exhibited a broad range of variation (Fig 5; S7–S9 Figs, S1 tRNA Multiple Alignments). Most of Mysticeti showed a smaller number of CSBPSs as Odontoceti. *E*. *robustus* and, more markedly, *C*. *marginata* were two exceptions to this behaviour. Within Odontoceti, most of Delphinidae exhibited a lower number of CSBPSs than did members of other families. *P*. *blainvillei* and *L*. *vexillifer* showed the maximum number of CSBPSs. Other species with at least 20 CSBPSs were *Berardius bairdii*, *I*. *geoffrensis*, *K*. *breviceps*, *Pl*. *minor*, and *P*. *macrocephalus*. The *O*. *orca* complex was a peculiar case to be analysed. If this taxon was considered an assembly of multiple cryptic species, a very low number of CSBPSs could be detected. In contrast, if different specimens of *O*. *orca* were considered to be derived from a single species, then a minimum of 17 CSBPSs could be assigned to this taxon (Fig 5).

A comparison of the reference topology (S1 Fig) with the distribution of CSBPSs (Fig 5) showed that a good agreement existed among the lengths of the branches and the numbers of CSBPSs.

*M*. *grayi* and *N*. *asiaeorientalis* were not considered in most of the analyses performed in the present paper (see Materials and Methods above). However, it was possible to include the tRNAs of these species in the multiple alignments (S1 tRNA Multiple Alignments).

The analysis of these alignments allowed us to identify for *M*. *grayi* at least 5 FCBCs, 3 HCBCs and 2 mismatches. Particularly interesting was the presence in the 2tp-pair of *trnN* of a type II FCBC (i.e., A–T vs. T–A). The total number of 10 CSBPSs found in *M*. *grayi* agreed with the values obtained for other *Mesoplodon* species (Fig 5).

Two HCBCs were unquestionably peculiar to *N*. *asiaeorientalis*. They were located at the 5tp-pair of *trnD* and at the 2tp-pair of *trnS2*. Furthermore, *N*. *asiaeorientalis* shared with *N*. *phocaenoides* most of the CSBPSs recorded for this latter species (Fig 5; S1 tRNA Multiple Alignments).

### Intraspecific variation in CSBPSs

The intraspecific level of CSBPS variation was studied in nine cetacean species (S1 Extended Results, S2 Table). The analyses were performed for *Balaenoptera physalus*, *M*. *densirostris*, *Mesoplodon europaeus*, *O*. *orca*, *P*. *macrocephalus*, *Tursiops aduncus*, *Tursiops australis*, *Tursiops truncates*, and *Z*. *cavirostris*. Intraspecific FCBCs were not identified, regardless of the number of mtDNAs analysed (8–152). In contrast, a variable but limited number of HCBCs and mismatches was detected. The number of tRNAs containing these CSBPSs varied from 1 (*M*. *europaeus* and *T*. *australis*) to 13 (*B*. *physalus*). In general, the intraspecific variation in CSBPS was limited.

## Discussion

### Proximate causes of tRNA evolution in Cetacea

As outlined in the introduction, tRNAs are at the core of mitochondrial activity and play a key role in the synthesis of mtDNA-encoded proteins.

Our analysis focussed on the changes that occurred in the stems of 22 tRNAs because they have a major impact on the structural integrity of these molecules. It is the conservation of the structural integrity, tightly linked to the ability to properly deliver the amino acid inside the mitochondrial ribosome, that dictates the limit in variation that every tRNA can afford during its evolution.

The results in the present paper show that the 22 mitochondrial tRNAs experienced continuous change during the cladogenesis of Cetacea. However, these changes occurred in different tRNAs with very different paces in terms of both numbers and types. This finding supports the view of a complex relationship among tRNAs and several factors that can produce variation in the stem pairs. Furthermore, it is evident that a fine tuning of the actions exerted by several causes determined the diversity of effects described above.

A total of 136 FCBCs, 320 HCBCs and 147 mismatches were identified in the analysis of 94T-set. A few more CSBPSs were detected in *M*. *grayi*, *N*. *asiaeorientalis* and in the study of the intraspecific variation of selected species (see above). These latter CSBPSs did not substantially alter the main outputs derived from the analysis of 94T-set. Thus, the discussion will be focused mostly on the results obtained from the principal dataset.

Type I FCBCs dominated the evolution of cetacean tRNA stems (97%), while type II FCBCs were very rare events. The occurrence of type I FCBCs was strongly favoured and promoted by the pivotal role played by the G•T, T•G pairs involved in most HFBCs. Indeed, these pairs provide a very efficient switch to pass from A–T to G–C and vice versa without disrupting the stem integrity through two rounds of HCBCs. In contrast, a type II FCBC requires an intermediate mismatch involving a couple of identical bases, potentially hampering the stem structure or the simultaneous substitution of both nucleotides of pair with different types of bases, a very rare event.

It has been shown that A–T and G–C pairs (and their symmetrical opposites) represent the top peaks of the fitness landscape describing the evolutionary history of mitochondrial tRNAs [66]. In contrast, G•T and A|C pairs are regarded as valleys of lower fitness that must be crossed to go from one peak to the other [66]. However, the two valleys are structurally very different because G•T does not jeopardise the tRNA stem structure, while A|C hampers the stem integrity. Thus, in the fitness landscape, the G•T valley can easily be crossed. In contrast, the pathway passing through A|C to go from A–T to G–C and vice versa is much steeper. This statement is corroborated by different types of scientific evidence ranging from structural biology [23] and free energy calculations for different base pairings [24] to comparative sequence analysis and homology modelling [21] applied not only to tRNAs but to various types of RNA molecules. Finally, the valley connecting the two peaks representing the alternative pairs of a type II FCBC is very deep and difficult to be crossed, as demonstrated by the extremely limited number of these events in Cetacea. The difficulty in passing through such a valley is further corroborated by the fact that the mismatch can be almost/fully fixed, as shown in present study for *trnN*. However, a mismatch is not necessarily a defect for a stem provided that it may represent a recognition signal for molecular partners [36]. Thus, it can remain in tRNA for a long time.

Our analysis showed that the most active stem positions are involved with CSBPSs, and the positions that are affected by post-transcriptional modifications on tRNAs are linked by a complex pattern, also considering that our knowledge of the occurrence of the latter in Mammalia is rather limited [19].

The abundance of codon families does not appear to have had a major impact on CSBPS evolution. The composition of the strand encoding the different tRNAs as well as the AT- and GC-skews exerts some control on the global pattern observed for CSBPSs as previously outlined by Helm et al. [36]. However, the distribution of FCBCs, HCBCs and mismatches is much more influenced and controlled by the base composition, AT-skew, and GC-skew of the tRNA stems. Indeed, our analysis has shown that it is the range of variation of composition and skews, particularly the extent of fluctuation from positive to negative values in the tRNA stems, that deeply affects the dynamics of the observed changes. Furthermore, this behaviour can be heterogeneous even in different stems of the same tRNA and determines, at a micro-scale level, the occurrence and abundance of different CSBPSs. The latter can be limited to single species or extended to a variable number of taxa. A stochastic component certainly influences this process and acts at the most dynamic pairs of the stems generating the convergent/parallel evolutionary changes described above. This does not mean that tRNA stems evolve at a very fast pace, as demonstrated by the values of the (maximum composite likelihood distance—p-distance) difference, which demonstrated that the substitution process is very far from saturation.

The type and distribution of CSBPSs is not only variable among the 22 tRNAs but also very different among different species. Thus, some CSBPSs can be proficiently used as good molecular signatures to delimit and define clades within Cetacea. The CSBPS intraspecific variation was limited, and no FCBCs were detected among the analysed species. However, the samples were small, and a much better coverage is necessary to fully assess this point.

The analysis of the distribution of CSBPSs in the Cetacea tree allowed us to hypothesise the role played by some factors in the evolutionary changes that occurred in the mitochondrial genome of Cetacea during their return to water. These evolutionary pathways are discussed in the next paragraph.

### Anatomical and physiological requirements and tRNA evolution

Recent evidence [67] suggests that gene families associated with stress-responsive proteins and anaerobic metabolism are expanded in cetaceans, while genes linked to sensory receptors and body hair are contracted (for the latter, see also Nery et al. [68]). This confirms that whale and dolphin genomes reflect the physiological need for a breath-holding based metabolism and intense stress due to increased reactive oxygen species and a high-salt environment. The high energy requirements of life in the water are testified also by the parallel evolution of the *IDH2* gene, which encodes an enzyme involved in aerobic metabolism in cetaceans, primates and bats [69].

In fact, a novel expansion of a polyalanine tract of the homeobox (Hox) genes *Hoxd12* and *Hoxd13* in cetaceans implicates a selective pattern of development of the specific morphology of the thoracic limb that is transformed into the typical flipper [70]. Conversely, in contrast to that expected, the primate-dolphin comparison showed that the evolution of *microcephalin* (*MCPH1 brain-development gene*) was not associated with brain size in cetaceans [71], thus failing to pinpoint an evolutionary factor responsible for the highest brain mass in the clade of mammals.

According to our data, the distribution of CSBPSs is very variable in the different cetacean families (Fig 5). Most species of Delphinidae exhibit fewer CSBPSs than do other toothed whales. The *O*. *orca* complex represents the main exception to this behaviour. The smaller number of CSBPSs observed in Delphinidae has possibly been influenced by a combination of the physiological requirements of the species belonging to this family (see below) as well the relative younger age of the clade with respect to that of other Cetacea lineages [55].

Phocoenidae and Monodontidae exhibit a number of CSBPSs slightly higher than but still comparable to that of Delphinidae. The shape of the body and the general food preferences of these three families are similar and may cause the differences in the deep divers, including Kogiidae, Physeteridae and Ziphiidae, and in the estuarine and freshwater species, including Pontoporiidae, Iniidae, Lipotidae and Platanistidae. Toothed whales that live in estuarine and freshwater habitats and cetaceans that repeatedly hunt at great depths face different but equally challenging physiological stresses only partially shared by taxa living in less extreme environments [72,73]. We also emphasise that some physiological parameters (including bradycardia at great depths) show specific characteristics (high percentage of arrhythmias), at least in the trained bottlenose dolphin *T*. *truncatus* [74], indicating the persistence of ancestral terrestrial traits in cardiac functions that would be difficult to maintain during the routine deep foraging of beaked and sperm whales.

The reference tree used to map the CSBPSs (S1 Fig) was obtained from the analysis of the amino acid alignment. The length of the branches provides good evidence for the amount of positive selection that occurred in the 13 proteins encoded in the mitochondrial genome. The branches connecting estuarine, freshwater and deep divers of Odontoceti are among the longest observed in the reference tree (S1 Fig). Indeed, they are significantly longer that those connecting other cetacean taxa (p < 0.0005; one-tailed Student’s t-test, unequal sample sizes, and unequal variances). This finding supports the view that in these taxa, the proteins were subjected to positive selection, as shown previously for *P*. *macrocephalus* [75]. CSBPSs are much more abundant in taxa that are characterised by long branches (Fig 5; S1 Fig) than in other cetacean species (p < 0.00005; one-tailed Student’s t-test, unequal sample sizes, and unequal variances). This match supports the view that not only did PCGs and their protein products experience positive selection, but tRNAs were subjected to an acceleration of the substitution process, which increased the number of CSBPSs. We suggest here that the challenging environments inhabited by estuarine, freshwater and deep water Odontoceti were responsible for at least part of the increased rate of base changes observed in the mitochondrial genomes of these taxa. Extreme environmental conditions have left their signature in the control region and in the coding genes of the mitochondrial genomes of high-altitude mammals [76]. Similarly, the mtDNA of the pika *Ochotona curzoniae* appears to harbour evidence of adaptation to cold and hypoxia [77]. In general, signatures of adaptive evolution have been found in the mitochondrial genomes of various mammals with specialised metabolic requirements [78].

Alternatively, it could be argued that the diverse numbers of CSBPSs observed in the various toothed whales are simply the result of a random substitution process linked to the different ages of the species. In this scenario, older species exhibit a higher number of CSBPSs because there was more time available for random substitution to occur.

To test this second hypothesis, which it is not necessarily an alternative to the environment-driven evolution described above, we compared the abundance of CSBPSs with the age of the taxa.

Different time estimations exist for the appearance and split of major phyletic lineages of Cetacea [12,15,55–58,62,63]. Dating is not consistent in different papers, and discrepancies exist (see the references cited above).

The currently most complete dating for the Cetacea clade is that provided by McGowen et al. [55]. According to these authors and considering only the dates relevant for the present paper, we have the following estimates (in brackets is the 95% interval range): (a) 24.21 MYA (15.83–31.93) for the split between *P*. *microcephalus* and *Kogia* genus; (b) 16.68 MYA (11.35–22.51) for the split between *I*. *geoffrensis* and *P*. *blainvillei*; c) 22.15 MYA (16.93–27.30) for the occurrence of the last common ancestor of *L*. v*exillifer* and *I*. *geoffrensis* + *P*. *blainvillei*; d) 32.43 MYA (27.92–37.07) for the appearance of the lineage leading to *Platanista* genus; e) 10.08 MYA (7.34–12.88) for the onset of Delphinidae; and f) 13.80 MYA (9.99–19.32) for the origin of Balaenopteridae + *E*. *robustus*. These estimations are more or less in agreement with the molecular dating based on complete mtDNA published by Hassanin et al. [15]. These authors provide the following estimates: a) 21.9 ± 3.6 MYA for the split between *P*. *macrocephalus* and *K*. *breviceps*; b) 14.0 ± 3.0 MYA for the split between *I*. *geoffrensis* and *P*. *blainvillei*; and c) 19.9 ± 3.2 MYA for the last common ancestor of *L*. *vexillifer* and *I*. *geoffrensis* + *P*. *blainvillei*.

Despite the variation in the absolute values of the estimates, the split between *P*. *macrocephalus* and *Kogia* occurred clearly before the separation between *I*. *geoffrensis* and *P*. *blainvillei*. Furthermore, *L*. *vexillifer* belongs to an older branch than the two species just mentioned. Finally, the differentiation of the lineage giving birth to the *Platanista* genus was a very early cladogenetic event. All of these taxa exhibit similar numbers of CSBPSs, and younger species may present more CSBPSs that do older species (*O*. *blainvillei* vs. *K*. *breviceps*). Similarly, even if we assigned to Balaenopteridae species the CSBPSs of the intermediated nodes present in the pathways connecting the root of this family with current taxa, thus spanning the whole 13.80 MYA of evolution, we still have much lower values than those of *P*. *blainvillei*. An analogous reasoning can be applied to Delphinidae.

As shown in the Results section, the substitution process in the stems of tRNAs is far from saturation. Thus, the observed distribution of CSBPSs cannot be explained in terms of pure random drift, even if random drift cannot be fully excluded and certainly plays/played some role in Cetacea tRNA evolution.

*C*. *marginata* was the only baleen whale with a high number of CSBPSs (Fig 5). This species is the sole living representative of a lineage thought to be extinct [79], and its physiology and habitat requirements are poorly known [80]. Thus, it is currently impossible to identify the main forces that shaped the evolution of mitochondrial tRNAs in this taxon.

### Concluding remarks

During the transition from terrestrial to aquatic environments, the body plan and the physiology of Cetacea were extensively modified, and strong molecular signatures of these changes are becoming well documented in their nuclear genomes [81]. The results presented in this paper show that mitochondrial genomes harbour in their sequences evidence of the transition from terrestrial to aquatic environments and also permit differentiation among the different habitats currently inhabited by Cetacea.

The evolution of CSBPSs was not constant during the cladogenetic process that lead to current Cetacea and experienced two peaks of acceleration. The first peak occurred during the return to the water by the common ancestors of whales, dolphins and their relatives. The second one arose with the entering of some taxa into the more demanding environments represented by freshwater, estuarine and deep water habitats (Fig 5).

We outline here that the “extreme environment” hypothesis of the evolution of cetacean tRNAs represents the best interpretation of our data given the analyses performed in the present work. However, we do not claim that the evolution of the tRNAs was shaped only/mostly by harsh environmental conditions. The process was certainly influenced by other causes, including the genetic drift described above. Thus, further studies are necessary to improve our current understanding of the evolution of cetacean tRNAs.

## Supporting Information

S1 Extended Results File including more details on the analyses performed in this paper. (PDF)

S1 Fig The phylogeny of Cetartiodactyla. Maximum likelihood (-lnL = 66963.002109) phylogram depicting the phylogenetic relationships among the major clades of Cetartiodactyla. The tree was created by analysing the amino acid 94T-set (3716 positions) with the RAxML 7.4.2 program implemented in raxmlGUI 1.3.1. The evolutionary model was MTMAM + F + CAT. Thirteen partitions were applied: one for every protein. The numbers represent bootstrap values expressed in percent. Only bootstrap values ≥ 50% are provided for the nodes. The scale bar represents 0.05 substitutions/site.
(PDF)

S2 Fig The mitochondrial genome of *Ziphius cavirostris*. The gene order is depicted and linearised starting from cox1. Genes encoded on the α-strand (right to left orientation) are underlined in green, while those encoded on the β-strand are underlined in red (left to right orientation). Gene nomenclature: *atp6* and *atp8*: *ATP synthase subunits 6* and *8*; *cob*: *apocytochrome b*; *cox1-3*: *cytochrome c oxidase sub-units 1–3*; *nad1-6* and *nad4L*: *NADH dehydrogenase subunits 1–6 and 4L*; *rrnS* and *rrnL*: small and large subunit ribosomal RNA (rRNA) genes; and X: transfer RNA (tRNA) genes, where X is the one-letter abbreviation of the corresponding amino acid. In particular, L1 identifies the CTN codon family, L2 the TTR codon family, S1 the AGY codon family, and S2 the TCN codon family. CR, Control Region. OL, origin of duplication of the light strand. A black circle located between two adjacent genes denotes the presence of an intergenic spacer (in white is the number of nucleotides forming the spacer). A white circle located between two adjacent genes denotes the presence of an overlapping segment (in red is the number of nucleotides forming the segment). Isp, intergenic spacer; start, start of the gene; end, end of the gene; size, size of the gene. For protein-coding genes, the start codon is provided in cyan and the stop codon in red (with incomplete stop codons written in parentheses). The anticodon is provided for every tRNA (e.g., tga for *trnS2*).
(PDF)

S3 Fig Secondary structure of Cetacea tRNA and level of conservation (*trnA*-*trnK*). pDis, p-Distance calculated for each pairwise-comparison orthologous tRNAs. MLdis, maximum composite likelihood distance calculated for every pairwise-comparison. DIF (MLdis–pDis), the difference between MLdis and pDis. The average values and the standard deviation are provided for both pDis and DIF. The values were computed for each set of orthologous tRNAs.
(PDF)

S4 Fig Secondary structure of Cetacea tRNA and level of conservation (*trnL1*-*trnV*). pDis, p-Distance calculated for each pairwise-comparison orthologous tRNAs. MLdis, maximum composite likelihood distance calculated for every pairwise-comparison. DIF (MLdis–pDis), the difference between MLdis and pDis. The average values and the standard deviation are provided for both pDis and DIF. The values were computed for each set of orthologous tRNAs.
(PDF)

S5 Fig AT-skew vs. A+T% and GC-skew vs. G+C% in the 94T-set mtDNAs. The values were calculated on the α-strand of the full-length mtDNA genomes. The X axis provides the skew values, while the Y axis provides the A+T% and G+C% values.
(PDF)

S6 Fig AT-skew vs. A+T% (A), and GC-skew vs. G+C% (B) in Cetacea mtDNAs. The values were calculated on the α-strand of the full-length mtDNA genomes. The X axis provides the AT- and GC-skew values, while the Y axis provides the A+T% and G+C% values. Species with a placement that is difficult to identify in the main plots are depicted in the frames.
(PDF)

S7 Fig Mapping of FCBCs on the Cetacea phylogenetic tree. FCBC, fully compensatory base change; SPIC, stem position involved in base change. The tRNA and the stem pair involved in FCBCs are mapped on the corresponding nodes of the reference phylogenetic tree. The tRNAs are depicted with the single-letter IUPAC code used for the corresponding amino acid. In particular, L1 identifies the CTN codon family, L2 the TTR codon family, S1 the AGY codon family, and S2 the TCN codon family. The stem pair involved in FCBC is provided in superscript. The asterisk, associated with some FCBCs indicates that these FCBCs were subjected to successive changes in one/some of the taxa located downstream of the considered node.
(PDF)

S8 Fig Mapping of HFBCs on the Cetacea phylogenetic tree. HFBC, hemi-compensatory base change; SPIC, stem position involved in base change. The tRNA and the SPICs involved in HFBCs are mapped on the corresponding nodes of the reference phylogenetic tree. The tRNAs are depicted with the single-letter IUPAC code used for the corresponding amino acid. In particular, L1 identifies the CTN codon family, L2 the TTR codon family, S1 the AGY codon family, and S2 the TCN codon family. The SPIC involved in HFBC is provided in superscript. The asterisk associated with some HFBCs indicates that these HFBCs were subjected to successive changes in one/some of the taxa located downstream of the considered node. A SPIC located on the 5’ side of a stem-pair is marked in orange, while a SPIC placed on the 3’ end of a pair is purple.
(PDF)

S9 Fig Mapping of Mismatches on the Cetacea phylogenetic tree. Mismatch, mismatch in a base pair of a stem; SPIC, stem position involved in base change. The tRNAs and SPICs involved in mismatches are mapped on the corresponding nodes of the reference phylogenetic tree. The tRNAs are depicted with the single-letter IUPAC code used for the corresponding amino acid. In particular, L1 identifies the CTN codon family, L2 the TTR codon family, S1 the AGY codon family, and S2 the TCN codon family. The SPIC involved in a mismatch is provided in superscript. The asterisk associated with some mismatches indicates that these mismatches were subjected to successive changes in one/some of the taxa located downstream of the considered node. A SPIC located on the 5’ side of a stem-pair is marked in orange, and a SPIC placed on the 3’ end of a pair is purple.
(PDF)

S1 Table List of taxa, accession numbers in GenBank and references. (PDF)

S2 Table Intraspecific CSBPSs identified in some Cetacea. (PDF)

S1 tRNA Multiple Alignments Multiple alignments of orthologous tRNAs. (PDF)
