# Supplementary material for: Back to Water: Signature of Adaptive Evolution in Cetacean Mitochondrial tRNAs
Source: PLoS One. 2016 Jun 23;11(6):e0158129. doi: 10.1371/journal.pone.0158129 (PMC4919058; doi:10.1371/journal.pone.0158129)
Supplement: S1 Extended Results — (PDF) [file pone.0158129.s001.pdf]

S1 Supplementary Extended Results

The mitochondrial genome of *Ziphius cavirostris*

The mtDNA of a specimen of *Z. cavirostris,* sequenced for this paper, is briefly described here. The new mitochondrial genome was 16,352 bp long. This value was very close to the average value obtained for the dataset analysed in the present work (16,436 ± 124). The *Z. cavirostris* genome contained the 37 genes almost universally found in animal mtDNAs i.e., 13 PCGs, two ribosomal rRNAs and 22 tRNAs. The gene order was typical for vertebrate mtDNAs (Fig 1), with 28 genes encoded on the α-strand and nine present on the opposite β-strand. Most of the PCGs started with ATG and ended with TAA or the incomplete stop codons TA(a) and T(aa). The genes on the same/opposite strand overlapped, were contiguous or were separated by intergenic spacers encompassing a variable number of nucleotides (S2 Fig). The mtDNA sequence of *Z. cavirostris* is available in EBI/GenBank under accession number LN997430.

Occurrence of CSBPSs in the tRNAs of Cetacea

The computation of values of the p-distance and (maximum composite likelihood distance – p-distance) difference allowed the level of conservation and the possible underestimation of the substitution patterns in Cetacea tRNAs (S3 and S4 Figs) to be determined. The most conserved tRNA was *trnG* (average-pDis = 0.049 ± 0.026) and the most variable was *trnH* (average-pDis = 0.134 ± 0.059). The minimum DIFF value (average-DIF = 0.000 ± 0.002) and the maximum value (average-DIF = 0.027 ± 0.019) were observed respectively in *trnE* and *trnH*. The (maximum composite likelihood distance – p-distance) difference values demonstrated that the observed CSBPSs did not grossly underestimate the true number of CSBPSs in the tRNAs.

A total of 603 CSBPSs (136 FCBCs, 320 HCBCs, 147 mismatches) were identified in the tRNAs of Cetacea (Fig 2; S10 tRNAs multiple alignments).

Most of FCBCs were of type I (131). These FCBCs were split in 36 A–T vs. G–C, 49 G–C vs. A–T, 24 C–G vs. T–A, 22 T–A vs C–G. The distribution on α-/β-strand tRNAs of type I FCBCs was 35/1, 38/11, 19/5, and 22/2. Four FCBCs were of type II. T–A vs. A–T occurred on the 5an-pair of *trnH*. G–T vs. T–A was found on the 7ac-pair of *trnQ*. C–G vs. A–T was identified on the 2tp-pair of *trnE*. A–T vs. T–A was detected on the 2tp-pair of *trnN* (Fig 2; S10 tRNAs multiple alignments). This latter case showed the complete pathway of the process leading to a type II FCBC. Indeed the ougroups had the T–A pair, most of the ingroup taxa exhibited the mismatch T|T, and *K. brevices* and *Mesoplodon grayi* (see below for this latter species) exhibited the A–T pair. Finally, a peculiar FCBC

occurred in *trnF* (i.e. G•T vs. A|C), where a double change of bases generated a correct pairing against a mismatch.

The number of FCBCs, detected in a single tRNA, ranged from 1 (*trnC, trnE*, *trnI*, *trnN, trnS2*) to 18 *(trnH*). Ten or more FCBCs were found only in the α-strand encoded *trnF*, *trnH*, *trnK*, *trnR*, *trnS1* (Figs 1 and 2). Except for *trnY* (8 FCBCs), the β-strand tRNAs exhibited four or less FCBCs. The bases alternating on the type I FCBCs followed three patterns (a-c). (a) Only purines alternated at the 5' end of the pair and solely pyrimidines occurred at the 3'end (*trnD*, *trnG*, *trnI*, *trnL2* and *trnW*). (b) Both purine/pyrimidine bases occurred in the substitution at 5' and 3'ends of the pair (*trnA*, *trnF*, *trnH*, *trnK*, *trnL1*, *trnP*, *trnR*, *trnS1, trnT, trnV, trnY*) with a variable prevalence of the first/second type of base. (c) Only pyrimidines alternated at the 5'end and only purines occurred at the 3'end of the pair (*trnC*, *trnM*, *trnS2*) (S10 tRNAs multiple alignments).

The 320 HCBCs were distributed mainly in four symmetrical types a-d: (a) [5' (A vs. G), 3' (T)] (43) and its counterpart [5' (T), 3' (A vs. G)] (16); (b) [5'(C vs. T), 3' (G)] (6) and its opposite [5' (G), 3' (C vs. T)] (26); (c) [5' (G vs. A), 3' (T)] (67) and the opposite [5' (T), 3' (G vs. A)] (60); (d) [5' (T vs. C), 3' (G)] (23) and its counterpart [5' (G), 3' (T vs. C)] (33). Abundant was also the type (31) [5' (A), 3' (T vs. C)]. The α-strand tRNAs showed 144 HCBCs, while the β-strand tRNAs exhibited 176 HCBCs (Fig 2).

The HCBCs had a distribution β-strand biased. The maximum number of HCBCs occurred in *trnE* (35). Numerous HCBCs (≥23) were present in *trnA*, *trnP*, *trnQ*, *trnS2*, and *trnY*. The *trnC* (11 HCBCs) and more markedly *trnN* (0 HCBC) were exceptions. The HCBCs distribution was variable in the α-strand tRNAs with values ranging from 17 (*trnH*) to 2 (*trnM, trnV*).

The 147 mismatches belonged to 35 different types (Fig 2). Only the most abundant are described in details below. The symmetrical [5' (A vs. G), 3'(C)] and [5'(C), 3' (A vs. G)] mismatches occurred respectively 25 and 6 times. The [5' (C vs. T), 3' (A)] and [5' (A), 3' (C vs. T)] mismatches were found respectively 23 and 34 times. The remaining 31 types occurred 59 times and accounted for the 40.14% of the whole mismatches set. The distribution of mismatches was uneven and α-strand biased. Indeed ten α-strand tRNAs (i.e., *trnF*, *trnH*, *trnK*, *trnL2*, *trnM*, *trnR*, *trnS1*, *trnT, trnV* and *trnW*) accounted for most (131, 89.16%) of observed mismatches. Conversely, only seven mismatches occurred in the eight tRNAs of the β-strand.

Patterns of CSBPSs distribution in the Cetacea tRNAs

The distribution of CSBPSs was very variable in the tRNAs (Fig 2). Eight tRNAs of the α-strand (*trnF*, *trnH*, *trnK*, *trnL2*, *trnR*, *trnS1*, *trnT* and *trnW*) exhibited a linked distribution of FCBCs, HCBCs, and mismatches with all types of CSBPS ≥ 7. A second pattern, mainly observed in β-strand

tRNAs, implied a high number of HCBCs coupled with a low number of FCBCs and mismatches (*trnA*, *trnC*, *trnE*, *trnG*, *trnP*, *trnQ*, *trnS2*). The *trnD*, *trnL1*, and *trnY* exhibited a low number of mismatches coupled with a moderate number of FCBCs and a higher number of HCBCs. A low numbers of base changes characterised *trnI*, and *trnN*. Few FCBCs and HCBCs and good number of mismatches occurred in *trnM*. Finally, *trnV* exhibited a high number of mismatches coupled with a good number of FCBCs and a low number of HCBCs (Fig 2; S10 tRNAs multiple alignments).

Factors influencing the occurrence and type of CSBPSs

The total number of codons encoded by the cetacean mtDNAs was homogenous (average 3777.47 ± 1.23). The minimum (3775) and maximum values (3781) were observed respectively in *Orcaella brevirostris* and *P. minor*. The number of codons for each codon family was also homogenous. The most abundant family was Leu1 (codons per thousand codons = 133.10 ± 3.72). The less abundant family was Cys (codons per thousand codons = 6.07 ± 0.22) (Fig 2).

FCBCs, HCBCs, and mismatches were simultaneously abundant in α-strand tRNAs exhibiting a broad range of codons per thousand codons (*trnH*, *trnK*, *trnR*, *trnS1*, *trnL2*, *trnF*, *trnT*) (Fig 2). Mismatches were very low/absent in all the β-strand tRNAs. Mismatches were very low in *trnL1* (1) and *trnI* (2), both encoded on α-strand, representing the first and third most abundant family. The smallest number of CSBPSs occurred in *trnN* (0 FCBC; 0 HCBC; 1 mismatch), one of the less abundant families (codons per thousand codons = 25.35 ± 0.50). On the opposite *trnI*, one of the most abundant tRNAs, ranked as the second for the minimum number of FCBCs (1), HCBCs (3) and mismatches (2). In general, there was not a simple pattern linking codon family abundances and richness of FCBCs, HCBCs, and mismatches (Fig 2).

The bases contents, the AT-/GC-skews were computed for the tRNAs stems and compared with those of the encoding mtDNAs to test their effects on CSBPSs distribution (Fig 2; S5 and S6 Figs).

The tRNAs of β-strand exhibited negative/null AT-skew, and positive/null GC-skews values (Fig 2). The α-strand tRNAs showed a more composite pattern. The *trnS1* had always positive AT-skew values. In other tRNAs the AT-skews were negative/null (e.g., *trnG*), or varied from negative to positive values (e.g. *trnF*) within a more or less broad range. The GC-skew values were positive in *trnL1*, and positive/null in *trnG*. In other tRNAs the GC-skew values varied from negative to positive. The A+T contents were very variable (Fig 2).

The majority of tRNAs exhibited always A+T content ≥ 50%. However, *trnC*, *trnF*, *trnL2*, *trnP*, *trnR*, *trnS2*, *trnW*, and *trnY* exhibited some sequences with A+T < 50%. The A+T content was always < 50% in *trnM* stems.

On α-strand: (a) the tRNAs (i.e., *trnD*, *trnI*) with high A+T content (≥ 62.50%) and limited variation of AT-skews (± 0.04) and GC-skews (± 0.091) were linked to a small number of FCBCs; (b) a low A+T content (≤ 47.62%) combined with small AT-skew values (-0,176 – -0.059), and small GC-skew values (-0.040 – 0.043) was associated to a small number of FCBCs in *trnM*; (c) a high variation of A+T content coupled to a broad range variation of AT-, and GC-skews was linked to a high number of CSBPSs (e.g., *trnF*, *trnH*, *trnL2*, *trnR, trnT*). On β-strand a combination of always negative AT-skews and always positive GC-skews coupled with variable A+T content was linked to a high number of HCBCs, and low number of both FCBCs and mismatches (e.g., *trnA*, *trnE*, *trnP*, *trnQ*). An invariable A+T content, limited variation of AT-skews, and constant GC-skews characterized *trnN*, that exhibited one FCBC, one mismatch and none HCBC.

Globally, the occurrence of CSBPSs in different tRNAs was influenced by the combined action of the base content variation, and asymmetrical compositional biases of the stems that in several cases were opposite to the values computed for the strand encoding the analysed tRNAs. Particularly the range of variation and the fluctuation of bases content, AT-,and GC-skews had a major impact on the type and abundance of CSBPs (e.g., *trnH* vs. *trnN*) (Fig 2; S5 and S6 Figs).

Finally, the abundance of FCBCs, HCBCs, and mismatches did not appear to be linked to the genomic placement of different tRNAs (Figs 1 and 2). A couple of examples support this statement. *TrnA* and *trnN,* both on β-strand and adjacent, exhibited very different behaviours. Conversely *trnR* and *trnT*, both on α-strand and well separated, had very similar patterns.

The stem-positions associated to CSBPSs

The stem positions involved in base changes (hereafter named SPICs) were mapped and analysed in the different tRNAs (Figs 3 and 4). One, two and even all three types of substitution were observed in the same SPIC (e.g., *trnF*, Fig 3). The number of SPICs was very variable within the 22 tRNAs (Fig 3). The smallest number (2) of SPICs (5.00 % of the 40 stem-positions) occurred in *trnN*. At the opposite, *trnH* presented 23 SPICs (54.76% of the 42 stem-positions) (Fig 3). Due to the heterogeneity of the substitution patterns, a perfect correspondence did not exist between the percentage of SPICs and global percentage of CSBPSs occurring in a single tRNA. Thus, *trnH*, exhibiting the maximum number of SPICs, hosted 7.30% of the total 603 CSBPSs. Conversely, *trnR,* having a lower percentage of SPICs (47.62%) hosted 9.45 % of total CSBPSs. Similarly, *trnQ* with only 11 SPICs (26.19%) contained 5.47% of total CSBPSs, a value observed in tRNAs with SPICs percentages ≥ 35% (e.g., *trnY*). Despite these vagaries, the percentage of SPICs was in good agreement with the global percentage of CSBPSs. The percentages of SPICs and FCBCs exhibited a similar behaviour. Much more discrepancies existed among the percentage of SPICs and global

percentages of HCBCs, and mismatches. A couple of examples corroborate this statement. The SPICs percentage for *trnE* was 35.71 % while the HCBCs percentage was 10.94%, more than twice the percentage of HCBCs (5.31%) observed in *trnH*, that possessed the highest percentage of SPICs (see above). Similarly, *trnH* and *trnM* presented comparable percentages (6.12% vs. 5.44%) of mismatches but very different percentages of SPICs (54.76% vs. 26.19%) (Fig 3). Every tRNA exhibited a unique pattern of SPICs and associated types of FCBCs, HCBCs, mismatches.

The distributions of SPICs and CSBPSs were summarised in Fig 4. The occurrence of FCBCs was very variable in the pairs. None tRNA presented a FCBC in the 2ac-, and 4dh-pairs (Fig 4a). Conversely the 4ac-pair (7 tRNAs), the 2an-pair (7 tRNAs), and 2an-pair (8 tRNAs) were hot spots for the presence of FCBCs. The acceptor stem (3-6ac-pairs), the anticodon stem (1-2,5an-pairs) and the TΨC stem (2-4tp-pairs) contained most of SPICs associated to FCBCs. The DHU stem had a very limited number of SPICs associated to FCBCs.

The SPICs associated to HCBCs were variably distributed in the different tRNA. However, some hot spots emerged. The 5'end of dh1-pair hosted at least an HCBCs in nine tRNAs. On the opposite, the 3'end of 2dh-pair never exhibited an HCBC and the same was true for the 5'end of the 5tp-pair. The 5'-end and 3'-end could behave differently in the same pair. Thus, the 5'end of 3tp-pair hosted an HCBC in seven different tRNA while the 3'end presented an HCBC only in two tRNAs. No HCBC occurred in 3'-end of 2dh-pair.

The SPICs associated to mismatches were more abundant on acceptor and TΨC stems. The anticodon stem presented several SPICs associated to mismatches. Very a few SPICs hosting mismatches occurred in the DHU stem. Mismatches were never detected in some positions (e.g., 5'end of 1ac-pair, 4dh-pair, 1tp-pair) (Fig 4a). When the global percentage of FCBCs, HCBCs, mismatches occurring at the different SPICs was evaluated, the patterns that emerged largely mirrored the abundance of SPICs just described above (Fig 4b).

The occurrence of HCBCs exhibited an evident 5'end or 3'end distributional bias in the pairs of some tRNAs. In *trnG* 11 of the 12 HCBCs occurred at the 5'end of the involved pairs (Fig 3; S8 Fig). Likewise, in *trnH* 15 of the 17 HCBCs were located in the 3'end of the pairs (Fig 3; S8 Fig). Also, *trnF*, *trnK*, *trnR*, *trnS1*, *trnT* exhibited HCBCs distributional biases. The distribution of mismatches exhibited an evident 5'end or 3'end bias in *trnF*, *trnG*, *trnR*, *trnS1*, and *trnW* (Fig 3; S9 Fig).

Finally the known distribution of positions in the stems, where occur posttranscriptional modifications, was mapped and compared with the SPICs behaviour (Fig 4). A simple pattern linking these positions with CSBPSs/ SPICs was not identified.

Phylogenetic distribution of CSBPSs

The distribution of CSBPSs along the reference tree is summarized in Fig 5 while the full details are provided in the S7-S9 Figs.

The 70.32% of the CSBPSs were associated to living species of Cetacea while the remaining 29.68% was divided among the internal nodes of the tree. In living species, the percentage of FCBCs was 66.91%, that of HCBCs was 72.19%, and that of mismatches was 69.39% (Fig 5).

The four FCBCs of type II identified in Cetacea (see above) had a variable taxonomic distribution. Two were restricted to a single species i.e. *K. breviceps* (*trnN*), and *I. geoffrensis* (*trnE*) (S10 tRNAs multiple alignments). The FCBC occurring in *trnH* appeared at the onset of Cetacea. During the cladogenetic process, successive FCBCs of type I, HCBCs and a mismatch substituted this CSBPS in some cetacean species (S7-S9 Figs, S10 tRNAs multiple alignments, *trnH*). Finally, the FCBC of type II, found in *trnQ,* characterized most of Odontoceti (except *P. macrocephalus* + *K. breviceps*) and was followed by successive HCBCs.

The analysis of the distribution of the CSBPSs revealed the occurrence of a dynamic, continuous, and still ongoing evolutionary mechanism of changes on the stems of tRNAs. The oldest CSBPSs were followed by successive changes (marked with an asterisk in Fig 5) that occurred in descendant groups at different taxonomic ranks (S7-S9 Figs). The CSBPSs were in several cases molecular signatures for the different clades as shown in the examples described below a referred for simplicity to a single tRNA.

The A–T vs. G–C, a FCBC of type I occurring at the 1dh-pair of *trnH,* distinguished the Ziphiidae from other Cetacea (Fig 5; S7 Fig). Similarly the [5'(G vs. A), 3' (T)] HCBC, observed in the 7ac-pair of *trnH*, differentiated *P. blainvillei* from other cetacean taxa (Fig 5; S8 Fig, S10 tRNAs multiple alignments, *trnH*). Finally, the T|C vs. T–A, a mismatch located at the 3'end of the 2tp-pair of the same tRNA, sets apart Delphinidae from other Cetacea (Fig 5; S9 Fig).

In other cases, the CSBPSs represented events of convergent/parallel evolution. Thus, the T–A vs. C–G FCBC, located at the tp4-pair of *trnF*, was shared by *Balaena misticetus* and *Eschrichtius robustus* (Fig 5; S7 Fig, S10 tRNAs multiple alignments, *trnF*). Analogously, *Neophocaena phocaenoides, O. brevirostris, Feresa attenuata* and *O. orca* complex exhibited the same HCBC [5'(G vs. A), 3' (T)], at the 4ac-pair of *trnD* (Fig 5; S8 Fig, S10 tRNAs multiple alignments, *trnD*). Finally, the T|T vs. A–T mismatch, at the 6ac-pair of the *trnC*, appeared independently in *Sousa chinensis* and *O. orca* complex (Fig 5; S9 Fig, S10 tRNAs multiple alignments, *trnC*).

The substitution pattern produced in some cases the secondary reversion to the condition observed in outgroups, due to the limited possibility of combinations of the four bases. Thus, in *M. monoceros* a

secondary A–T vs. G–C FCBC occurred in the 5ac-pair of *trnW* (Fig 5; S7 Fig, S10 tRNAs multiple alignments, *trnW*). Similar reversions were observed also for HCBCs and mismatches.

The distribution of CSBPSs in living Cetacea exhibited a broad range of variation (Fig 5; S7-S9 Figs, S10 tRNAs multiple alignments). Most of Mysticeti showed a smaller number CSBPSs than Odontoceti. *E. robustus* (10 CSBPSs) and, more markedly, *C. marginata* (19 CSBPSs) were two exceptions to this behaviour. Within Odontoceti, most of Delphinidae exhibited a lower number of CSBPSs than members of other families. *P. blainvillei* and *L. vexillifer* showed the maximum number (30) of CSBPSs. Other species with at least 20 CSBPSs were *Berardius bairdii* (20), *I. geoffrensis* (23), *K. breviceps* (24), *Pl. minor* (28), and *P. macrocephalus* (26). *O. orca* complex was a peculiar case to be analysed. If this taxon was considered as an assembly of multiple cryptic-species a very low number of CSBPSs could be detected. Conversely, if different specimens of *O. orca* were considered to be derived from a single species, then a minimum of 17 CSBPSs could be assigned to this taxon (Fig 5).

Comparison of the reference topology (S1 Fig) with the distribution of CSBPSs (Fig 5) showed that a good agreement existed among the lengths of the branches and the numbers of CSBPSs.

The distribution of CSBPSs was further investigated in the ten species of Odontoceti with a minimum of 4 FCBCs (Fig 5). None tRNA, exhibiting at least one FCBC, was shared by all taxa. However, when a tRNA was shared by different species, a FCBC could be located in the same stem-pair in different taxa (e.g. 5ac-pair of *trnH* in *P. blainvillei and P. macrocephalus*) (S7 Fig, S10 tRNAs multiple alignments). Similar patterns were detected for HCBCs and mismatches (S8 and S9 Figs, S10 tRNAs multiple alignments).

M. grayi and *N. asiaorientalis* were not considered in most of the analyses performed in present paper (see above Materials and methods). However, it was possible to include the tRNAs of these species in the multiple alignments (S10 tRNAs multiple alignments).

The analysis of these alignments allowed to identify for *M. grayi* at least 5 FCBCs (*trnH*, 3tp-pair; *trnN*,; *trnQ*, 1-an-pair; *trnT*, 1an-pair; *trnW*, 5tp-pair), 3 HCBCs (*trnC*, 5' end of 1ac-pair; *trnF*, 5' end of 3an-pair; *trnQ*, 5'end of 3an-pair) and 2 mismatches (*trnL2*, 5' end of 7ac-pair; *trnV*, 3' end of 4an-pair). Particularly interesting was the presence in the 2tp-pair of *trnN* of a FCBC of type II (i.e. A–T vs. T–A). The total number of 10 CSBPSs found in *M. grayi* was in agreement with values obtained for other *Mesoplodon* species (Fig 5).

Two HCBCs resulted unquestionably peculiar to *N. asiaorientalis*. They were located respectively at the 5tp-pair of *trnD* [(G), (T vs. C)], and at 2tp-pair of *trnS2* [5'(A vs. G), 3'(T)]. Furthermore, *N. asiaorientalis* shared with *N. phocaenoides* most of CSBPSs recorded for this latter species (Fig 5; S10 tRNAs multiple alignments).

Intraspecific variation of CSBPSs

The intraspecific level of CSBPSs variation was studied in nine cetacean species (S2 Table). The analyses were performed for *Balaenoptera physalus*, *M. densirostris*, *Mesoplodon europapaeus*, *O. orca*, *P. macrocephalus*, *Tursiops aduncus*, *Tursiops australis*, *Tursiops truncates,* and *Z. cavirostris*. Intraspecific FCBCs were not identified, irrespective to the number of mtDNAs analysed (8–152). Conversely, a variable, but limited, number of HCBCs and mismatches was detected. *B. physalus* exhibited intraspecific HCBCs and mismatches in 13 tRNAs. *M. densirostris* presented CSBPSs in six tRNAs, while *M. europaeus* showed one HCBC in *trnL2*. In the *O. orca* complex intraspecific CSBPSs, not present in 7 sequences included in 94T-set, were found in seven tRNAs. *P. macrocephalus* exhibited CSBPSs in four tRNAs. *T. aduncus,* showed one HCBC in *trnF* and *trnT*. *T. australis* presented a mismatch in *trnS1*. In *T. truncatus* CSBPSs were found in *trnD*, *trnF*, *trnL2*, *trnR*, and *trnV*. Finally in *Z. cavirostris* a single HCBC was identified in *trnD*, *trnH*, *trnK*, *trnL2*, *trnS1*, and *trnT*. As a general behaviour, the intraspecific variation of CSBPSs resulted limited.
