# Supplementary material for: Back to Water: Signature of Adaptive Evolution in Cetacean Mitochondrial tRNAs
Source: PLoS One. 2016 Jun 23;11(6):e0158129. doi: 10.1371/journal.pone.0158129 (PMC4919058; doi:10.1371/journal.pone.0158129)
Supplement: S1 Fig — Maximum likelihood (-lnL = 66963.002109) phylogram depicting the phylogenetic relationships among the major clades of Cetartiodactyla. The tree was created by analysing the amino acid 94T-set (3716 positions) with the RAxML 7.4.2 program implemented in raxmlGUI 1.3.1. The evolutionary model was MTMAM + F + CAT. Thirteen partitions were applied: one for every protein. The numbers represent bootstrap values expressed in percent. Only bootstrap values ≥ 50% are provided for the nodes. The scale bar represents 0.05 substitutions/site. (PDF) [file pone.0158129.s002.pdf]

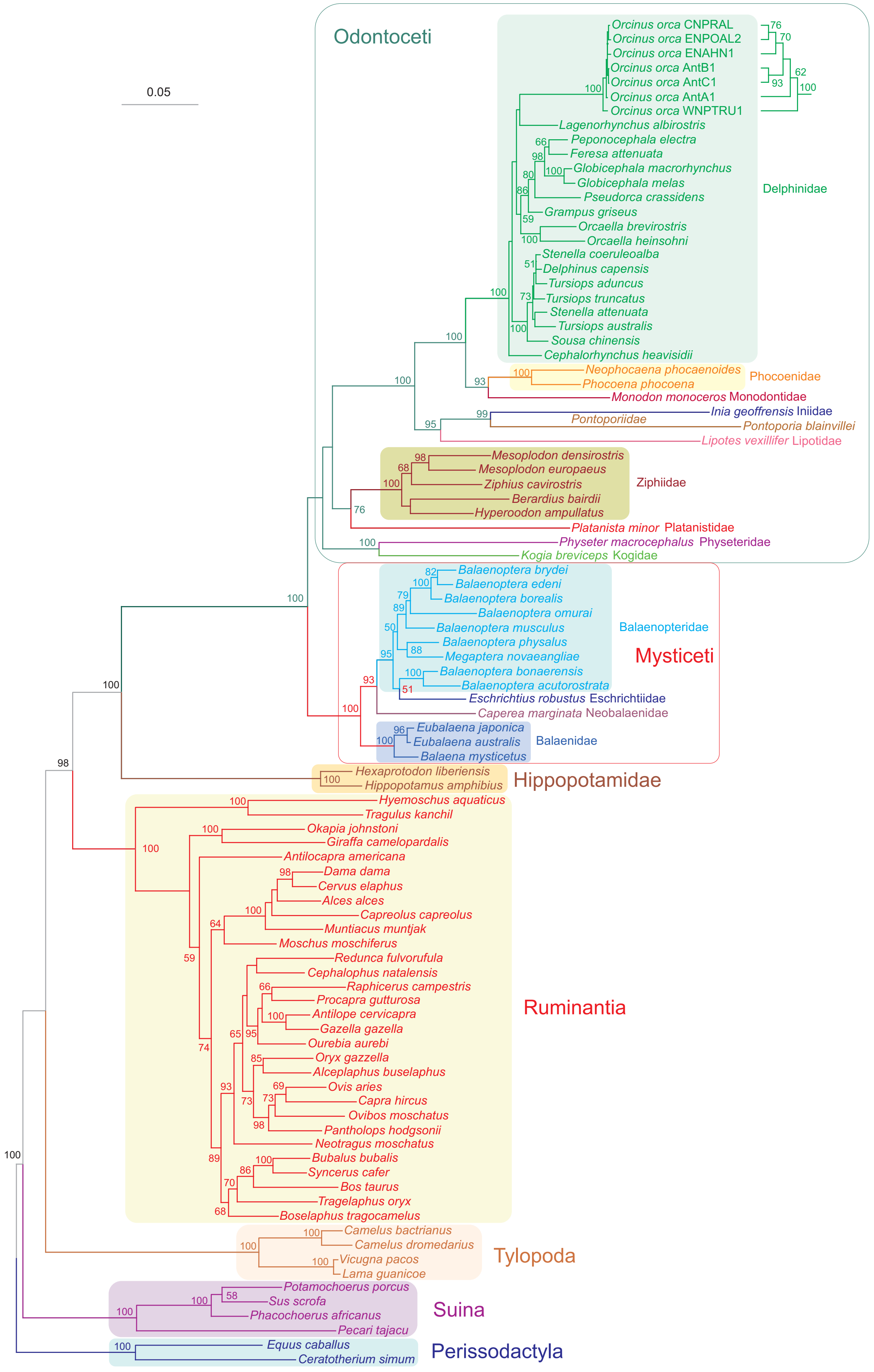

Figure S1. The phylogeny of Cetartiodactyla.
Maximum likelihood (-lnL = 66963.002109) phylogram depicting the phylogenetic relationships among the major clades of Cetartiodactyla. The tree was created by analysing the amino acid 94T-set (3716 positions) with the RAxML 7.4.2 program implemented in raxmlGUI 1.3.1. The evolutionary model was MTMAM + F + CAT. Thirteen partitions were applied: one for every protein. The numbers represent bootstrap values expressed in percent. Only bootstrap values ≥ 50% are provided for the nodes. The scale bar represents 0.05 substitutions/site.
