# Supplementary material for: Back to Water: Signature of Adaptive Evolution in Cetacean Mitochondrial tRNAs
Source: PLoS One. 2016 Jun 23;11(6):e0158129. doi: 10.1371/journal.pone.0158129 (PMC4919058; doi:10.1371/journal.pone.0158129)
Supplement: S2 Fig — The gene order is depicted and linearised starting from cox1. Genes encoded on the α-strand (right to left orientation) are underlined in green, while those encoded on the β-strand are underlined in red (left to right orientation). Gene nomenclature: atp6 and atp8: ATP synthase subunits 6 and 8; cob: apocytochrome b; cox1-3: cytochrome c oxidase sub-units 1–3; nad1-6 and nad4L: NADH dehydrogenase subunits 1–6 and 4L; rrnS and rrnL: small and large subunit ribosomal RNA (rRNA) genes; and X: transfer RNA (tRNA) genes, where X is the one-letter abbreviation of the corresponding amino acid. In particular, L1 identifies the CTN codon family, L2 the TTR codon family, S1 the AGY codon family, and S2 the TCN codon family. CR, Control Region. OL, origin of duplication of the light strand. A black circle located between two adjacent genes denotes the presence of an intergenic spacer (in white is the number of nucleotides forming the spacer). A white circle located between two adjacent genes denotes the presence of an overlapping segment (in red is the number of nucleotides forming the segment). Isp, intergenic spacer; start, start of the gene; end, end of the gene; size, size of the gene. For protein-coding genes, the start codon is provided in cyan and the stop codon in red (with incomplete stop codons written in parentheses). The anticodon is provided for every tRNA (e.g., tga for trnS2). (PDF) [file pone.0158129.s003.pdf]

Ziphius cavirostris mtDNA — 16552 bp

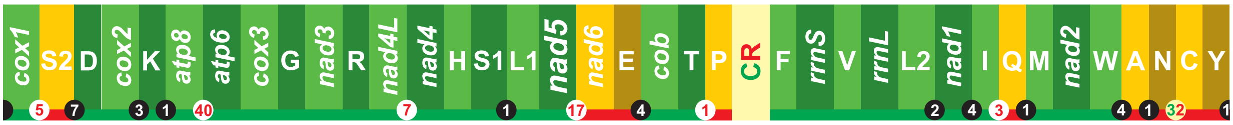

	start	end	size		
cox1	1	1551	1551	ATG	AGA
trnS2	1547	1615	69	tga	
isp (*trnS2*-*trnD*)	1616	1622	7		
trnD	1623	1690	68	gtc	
cox2	1691	2374	684	ATG	TAA
isp (*cox2*-*trnK*)	2375	2377	3		
trnK	2378	2444	67	ttt	
isp (*trnK*-*atp8*)	2445	2445	1		
atp8	2446	2646	201	ATG	TAA
atp6	2607	3286	680	ATG	TA(a)
cox3	3287	4071	785	ATG	TA(a)
trnG	4072	4140	69	tcc	
nad3	4141	4487	347	ATA	TA(a)
trnR	4488	4557	70	tcg	
nad4L	4558	4854	297	GTG	TAA
nad4	4848	6225	1378	ATG	T(aa)
trnH	6226	6295	70	gtg	
trnS1	6296	6355	60	gct	
isp (*trnS1*-*trnL1*)	6356	6356	1		
trnL1	6357	6426	70	tag	
nad5	6427	8247	1821	ATA	TAA
nad6	8231	8758	528	ATG	TAA
trnE	8759	8827	69	ttc	
isp (*trnE*-*cob*)	8828	8831	4		
cob	8832	9971	1140	ATG	AGA

	start	end	size		
trnT	9972	10042	71	tgt	
trnP	10042	10108	67	tgg	
Control Region	10109	10989	881		
trnF	10990	11063	74	gaa	
rrnS	11064	12035	972		
trnV	12036	12102	67	tac	
rrnL	12103	13682	1580		
trnL2	13683	13757	75	taa	
isp (*trnL2*-*nad1*)	13758	13759	2		
nad1	13760	14716	957	ATG	TAG
isp (*nad1*-*trnI*)	14717	14720	4		
trnI	14721	14789	69	gat	
trnQ	14787	14859	73	ttg	
isp (*trnQ*-*trnM*)	14860	14860	1		
trnM	14861	14929	69	cat	
nad2	14930	15971	1042	ATG	T(aa)
trnW	15972	16038	67	tca	
isp (*trnW*-*trnA*)	16039	16042	4		
trnA	16043	16111	69	tgc	
isp (*trnA*-*trnN*)	16112	16112	1		
trnN	16113	16186	74	gtt	
OL	16187	16218	32		
trnC	16219	16285	67	gca	
trnY	16286	16351	66	gta	
isp (*trnY*-*cox1*)	16352	16352	1		

Figure S2. The mitochondrial genome of *Ziphius cavirostris*.
Tthe gene order is depicted and linearised starting from ***cox1***. Genes encoded on the **α-strand** (right to left orientation) are underlined in **green**, while those encoded on the **β-strand** are underlined in **red** (left to right orientation). Gene nomenclature: ***atp6*** and ***atp8***: ATP synthase subunits 6 and 8; ***cob***: apocytochrome b; ***cox1-3***: cytochrome c oxidase subunits 1–3; ***nad1-6*** and ***nad4L***: NADH dehydrogenase subunits 1–6 and 4L; ***rrnS*** and ***rrnL***: small and large subunit ribosomal RNA (rRNA) genes; and X: transfer RNA (tRNA) genes, where X is the one-letter abbreviation of the corresponding amino acid. In particular, L1 identifies the CTN codon family, L2 the TTR codon family, S1 the AGY codon family, and S2 the TCN codon family. **CR**, Control Region. **OL**, origin of duplication of the light strand. A black circle located between two adjacent genes denotes the presence of an intergenic spacer (in white is the number of nucleotides forming the spacer). A white circle located between two adjacent genes denotes the presence of an overlapping segment (in red is the number of nucleotides forming the segment). **Isp**, intergenic spacer; **start**, start of the gene; **end**, end of the gene; **size**, size of the gene. For protein-coding genes, the **start codon** is provided in **cyan** and the **stop codon** in **red** (with incomplete stop codons written in parentheses). The anticodon is provided for every tRNA (e.g., **tga** for ***trnS2***).
