# Supplementary material for: Back to Water: Signature of Adaptive Evolution in Cetacean Mitochondrial tRNAs
Source: PLoS One. 2016 Jun 23;11(6):e0158129. doi: 10.1371/journal.pone.0158129 (PMC4919058; doi:10.1371/journal.pone.0158129)
Supplement: S4 Fig — pDis, p-Distance calculated for each pairwise-comparison orthologous tRNAs. MLdis, maximum composite likelihood distance calculated for every pairwise-comparison. DIF (MLdis–pDis), the difference between MLdis and pDis. The average values and the standard deviation are provided for both pDis and DIF. The values were computed for each set of orthologous tRNAs. (PDF) [file pone.0158129.s005.pdf]

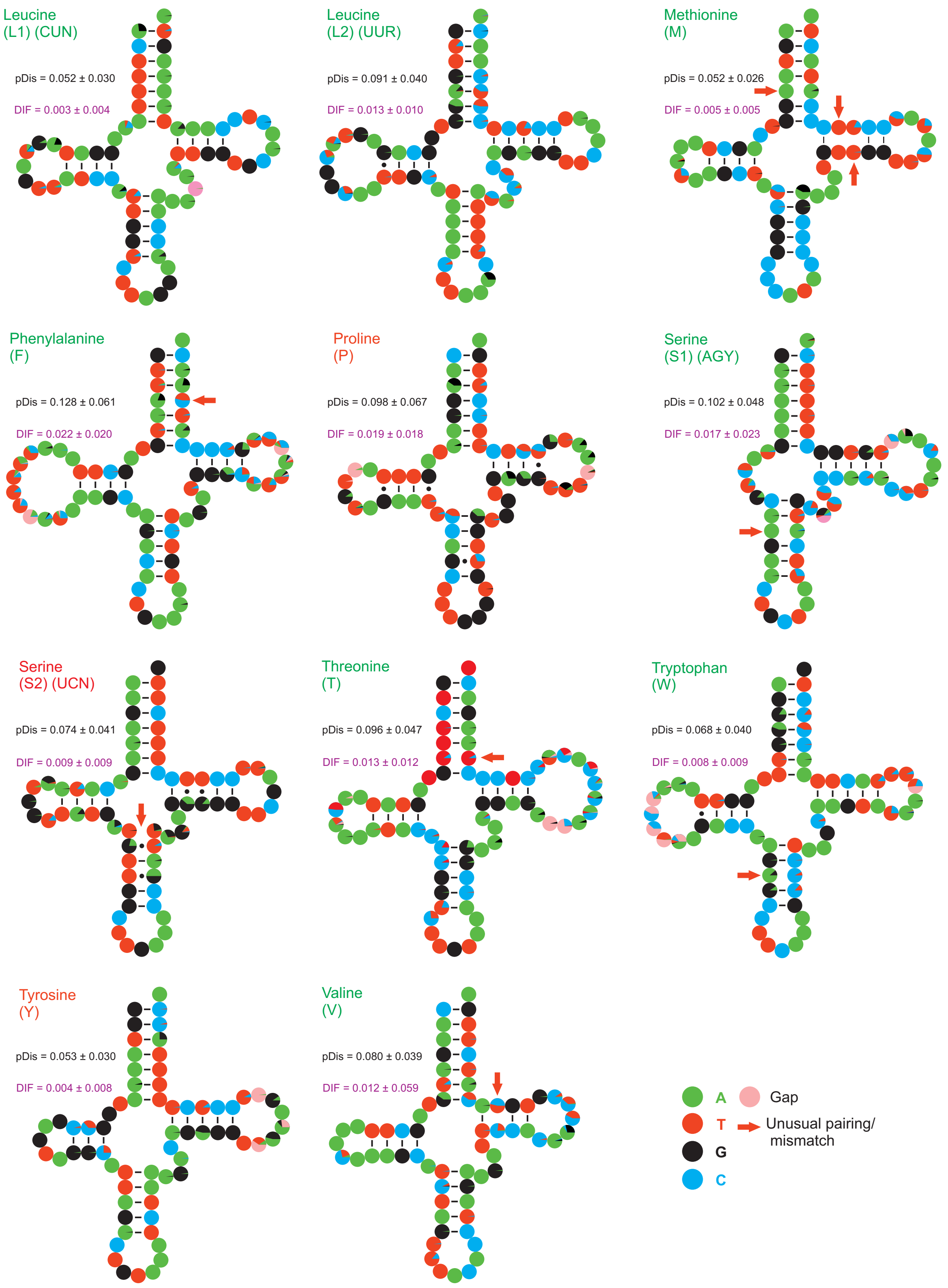

Figure S4. Secondary structure of Cetacea tRNA and level of conservation (*trnL1*-*trnV*).
pDis, p-Distance calculated for each pairwise-comparison orthologous tRNAs. **MLdis**, maximum composite likelihood distance calculated for every pairwise-comparison. **DIF** (**MLdis**–**pDis**), the difference between **MLdis** and **pDis**. The **average values** and the **standard deviation** are provided for both **pDis** and **DIF**. The values were computed for each set of orthologous tRNAs.
