# Supplementary material for: Back to Water: Signature of Adaptive Evolution in Cetacean Mitochondrial tRNAs
Source: PLoS One. 2016 Jun 23;11(6):e0158129. doi: 10.1371/journal.pone.0158129 (PMC4919058; doi:10.1371/journal.pone.0158129)
Supplement: S5 Fig — The values were calculated on the α-strand of the full-length mtDNA genomes. The X axis provides the skew values, while the Y axis provides the A+T% and G+C% values. (PDF) [file pone.0158129.s006.pdf]

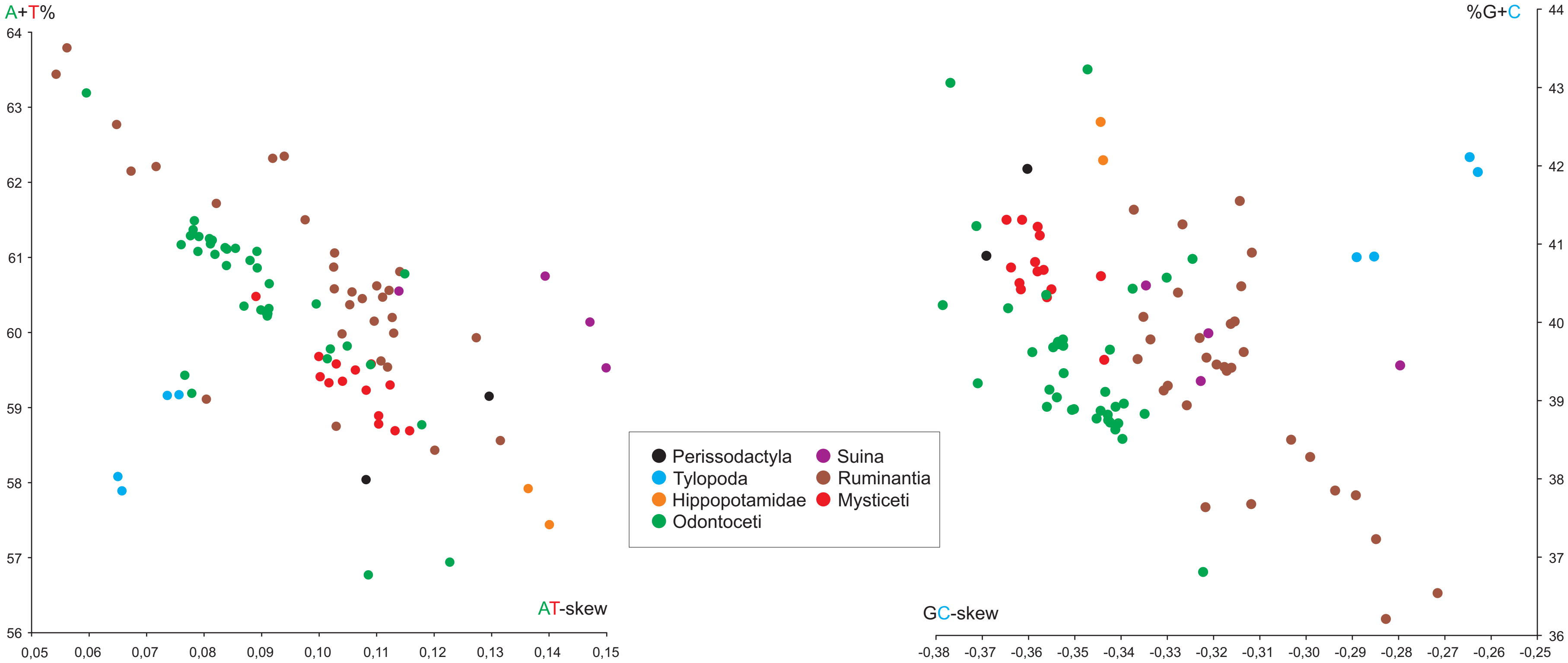

Figure S5. AT-skew vs. A+T% and GC-skew vs. G+C% in the 94T-set mtDNAs.
The values were calculated on the **α-strand** of the full-length mtDNA genomes. The X axis provides the skews values, while the Y axis provides the A+T% and G+C% values.
