# Supplementary material for: Back to Water: Signature of Adaptive Evolution in Cetacean Mitochondrial tRNAs
Source: PLoS One. 2016 Jun 23;11(6):e0158129. doi: 10.1371/journal.pone.0158129 (PMC4919058; doi:10.1371/journal.pone.0158129)
Supplement: S6 Fig — The values were calculated on the α-strand of the full-length mtDNA genomes. The X axis provides the AT- and GC-skew values, while the Y axis provides the A+T% and G+C% values. Species with a placement that is difficult to identify in the main plots are depicted in the frames. (PDF) [file pone.0158129.s007.pdf]

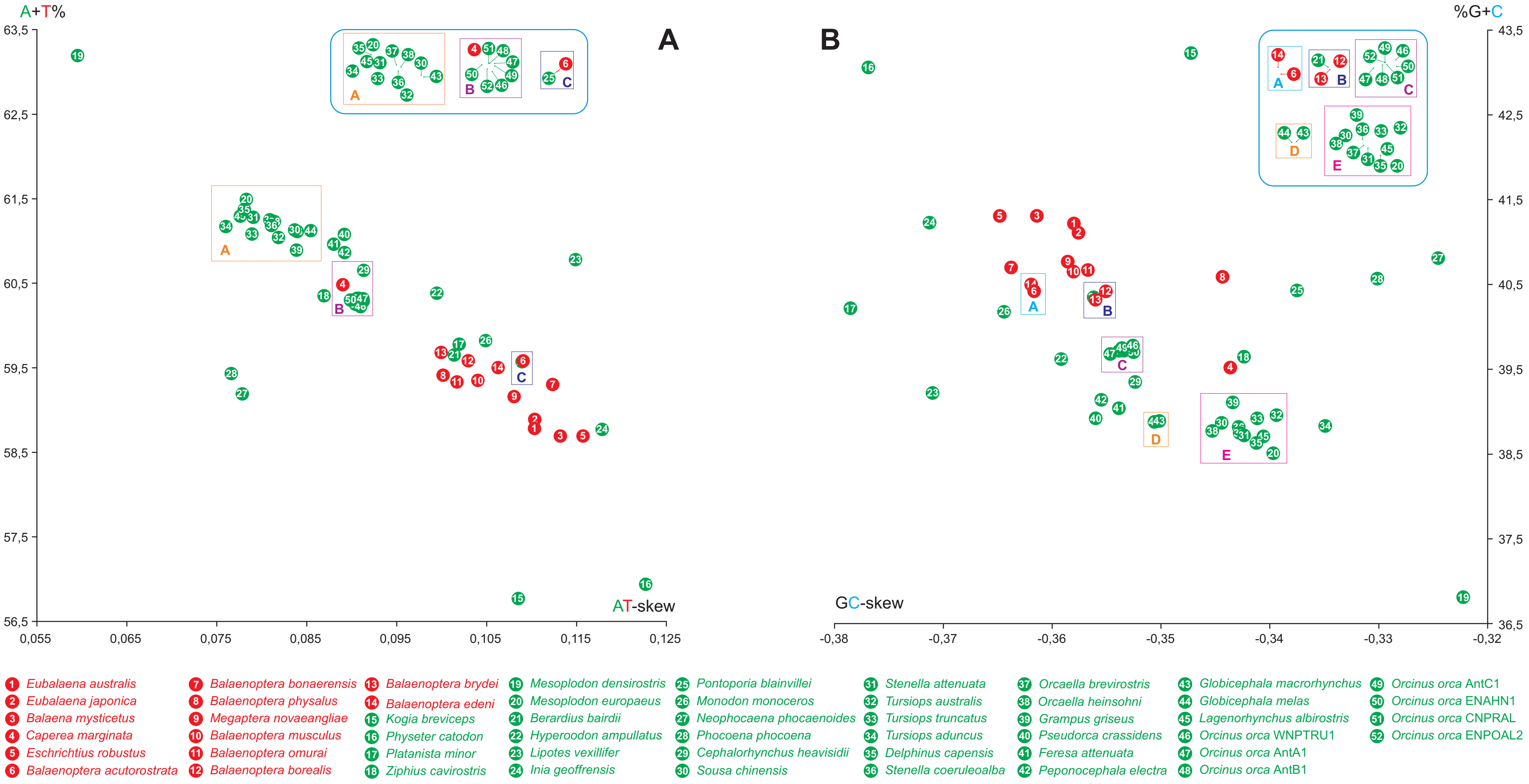

Figure S6. AT-skew vs. A+T% (A), and GC-skew vs. G+C% (B) in Cetacea mtDNAs.
The values were calculated on **α-strand** of the full-length mtDNA genomes. The X axis provides the **AT-** and **GC**-skews values, while the Y axis provides the **A+T%,G+C%** values. Species with a placement that is difficult to identify in the main plots are depicted in the frames.
