# Supplementary material for: Back to Water: Signature of Adaptive Evolution in Cetacean Mitochondrial tRNAs
Source: PLoS One. 2016 Jun 23;11(6):e0158129. doi: 10.1371/journal.pone.0158129 (PMC4919058; doi:10.1371/journal.pone.0158129)
Supplement: S7 Fig — FCBC, fully compensatory base change; SPIC, stem position involved in base change. The tRNA and the stem pair involved in FCBCs are mapped on the corresponding nodes of the reference phylogenetic tree. The tRNAs are depicted with the single-letter IUPAC code used for the corresponding amino acid. In particular, L1 identifies the CTN codon family, L2 the TTR codon family, S1 the AGY codon family, and S2 the TCN codon family. The stem pair involved in FCBC is provided in superscript. The asterisk, associated with some FCBCs indicates that these FCBCs were subjected to successive changes in one/some of the taxa located downstream of the considered node. (PDF) [file pone.0158129.s008.pdf]

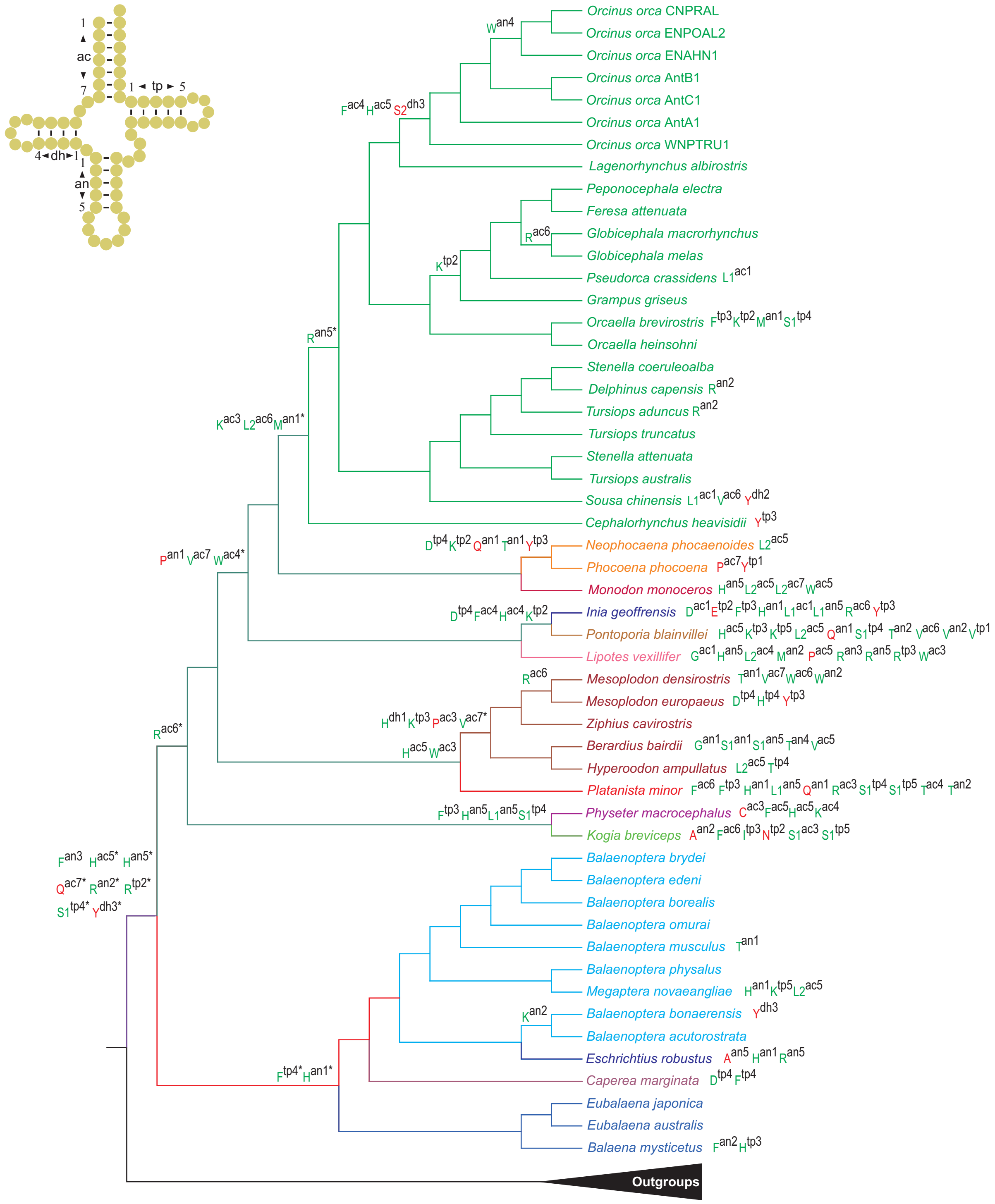

Figure S7. Mapping of FCBCs on the Cetacea phylogenetic tree.
FCBC, fully compensatory base change; **SPIC**, stem position involved in base change. The tRNA and the stem pair involved in **FCBCs** are mapped on the corresponding nodes of the reference phylogenetic tree. The tRNAs are depicted with the single-letter IUPAC code used for the corresponding amino acid. In particular, **L1** identifies the CTN codon family, **L2** the TTR codon family, S1 the AGY codon family, and **S2** the TCN codon family. The stem pair involved in **FCBC** is provided in superscript. The **asterisk**, associated with some **FCBCs** indicates that these **FCBCs** were subjected to successive changes in one/some of the taxa located downstream of the considered node.
