# Supplementary material for: Back to Water: Signature of Adaptive Evolution in Cetacean Mitochondrial tRNAs
Source: PLoS One. 2016 Jun 23;11(6):e0158129. doi: 10.1371/journal.pone.0158129 (PMC4919058; doi:10.1371/journal.pone.0158129)
Supplement: S9 Fig — Mismatch, mismatch in a base pair of a stem; SPIC, stem position involved in base change. The tRNAs and SPICs involved in mismatches are mapped on the corresponding nodes of the reference phylogenetic tree. The tRNAs are depicted with the single-letter IUPAC code used for the corresponding amino acid. In particular, L1 identifies the CTN codon family, L2 the TTR codon family, S1 the AGY codon family, and S2 the TCN codon family. The SPIC involved in a mismatch is provided in superscript. The asterisk associated with some mismatches indicates that these mismatches were subjected to successive changes in one/some of the taxa located downstream of the considered node. A SPIC located on the 5’ side of a stem-pair is marked in orange, and a SPIC placed on the 3’ end of a pair is purple. (PDF) [file pone.0158129.s010.pdf]

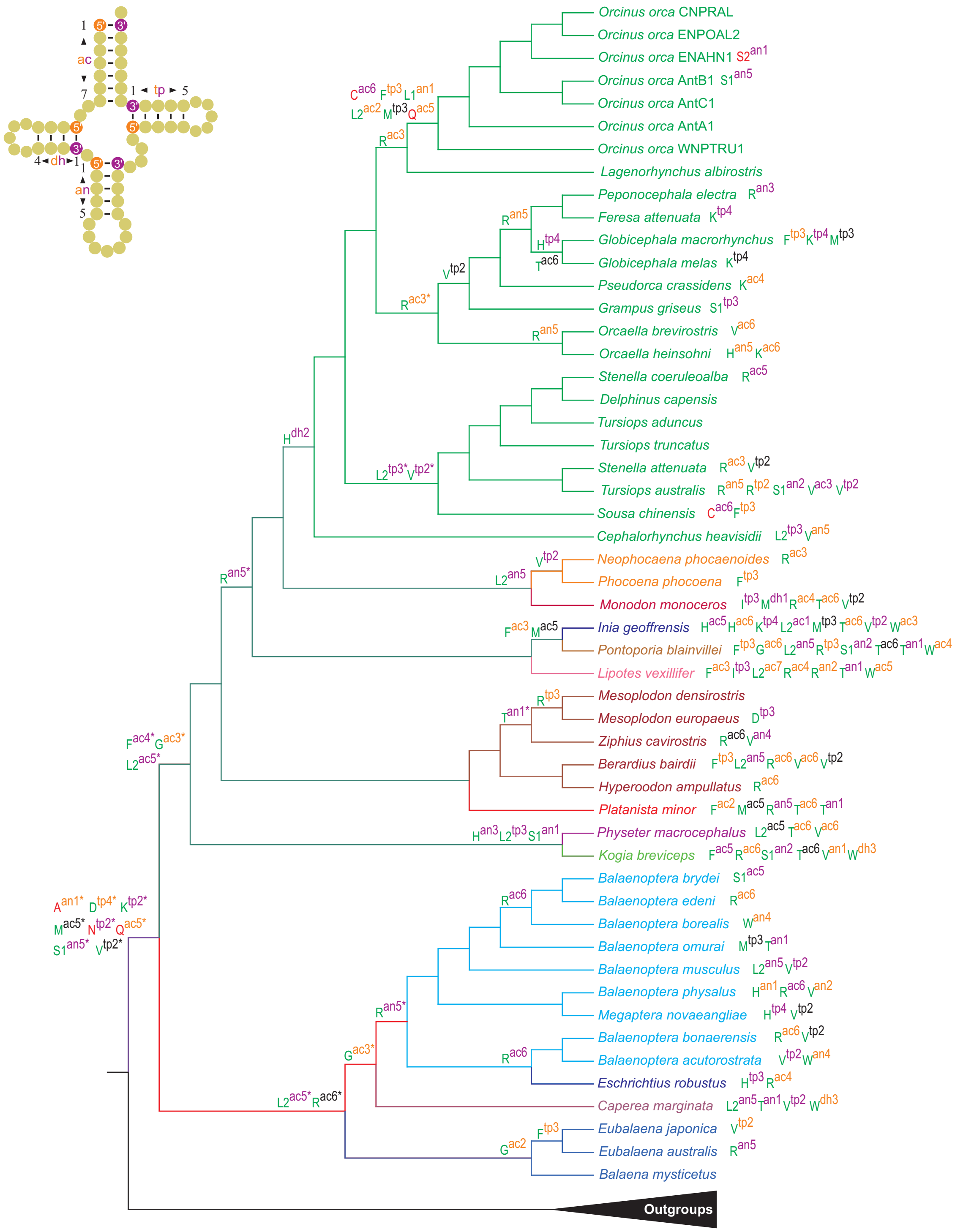

Figure S9. Mapping of mismatches on the Cetacea phylogenetic tree.

Mismatch, **mismatch** in a base pair of a stem; **SPIC**, stem position involved in base change. The tRNAs and **SPICs** involved in **mismatches** are mapped on the corresponding nodes of the reference phylogenetic tree. The tRNAs are depicted with the single-letter IUPAC code used for the corresponding amino acid. In particular, **L1** identifies the CTN codon family, **L2** the TTR codon family, **S1** the AGY codon family, and **S2** the TCN codon family. The **SPIC** involved in a **mismatch** is provided in **superscript**. The **asterisk** associated with some **mismatches** indicates that these **mismatches** were subjected to successive changes in one/some of the taxa located downstream of the considered node. A **SPIC** located on the **5' side** of a stem-pair is marked in **orange**, and a **SPIC** placed on the **3' end** of a pair is **purple**.
