# Supplementary material for: Back to Water: Signature of Adaptive Evolution in Cetacean Mitochondrial tRNAs
Source: PLoS One. 2016 Jun 23;11(6):e0158129. doi: 10.1371/journal.pone.0158129 (PMC4919058; doi:10.1371/journal.pone.0158129)
Supplement: S1 Table — (PDF) [file pone.0158129.s011.pdf]

Table S1. List of taxa, accession number in GenBank and reference (1/3)

			**Perissodactyla**		
		Equidae	*Equus caballus* Linnaeus, 1758	X79547	Xu and Arnason 1994
		Rhinocerotidae	*Ceratotherium simum* (Burchell, 1817)	Y07726	Xu and Arnason 1997
		**Cetartiodactyla Montgelard, Catzeflis and Douzery, 1997**			
**Suina**		Suidae	*Phacochoerus africanus* (Gmelin, 1788)	DQ409327	Wu et al. 2007
**Suina**		Suidae	*Potamochoerus porcus* (Linnaeus, 1758)	JN632688	Hassanin et al. 2012
**Suina**		Suidae	*Sus scrofa* Linnaeus, 1758	FJ237000	Unpublised; Alves and Fernandez
**Suina**		Tayassuidae	*Pecari tajacu* (Linnaeus, 1758)	AP003427	Unpublished; Yasue et al.
**Tylopoda**		Camelidae	*Camelus bactrianus* Linnaeus, 1758	EF212037	Ji et al. 2009
**Tylopoda**		Camelidae	*Camelus dromedarius* Linnaeus, 1758	JN632608	Hassanin et al. 2012
**Tylopoda**		Camelidae	*Lama guanicoe* (Müller, 1776)	EU681954	Di rocco et al. 2010
**Tylopoda**		Camelidae	*Vicugna pacos* (Linnaeus, 1758)	NC_002504	Ursing et al. 2000
**Ruminantia**	Pecora	Antilocapridae	*Antilocapra americana* Ord, 1815	JN632597	Hassanin et al. 2012
**Ruminantia**	Pecora	Bovidae Alcelaphinae	*Alceplaphus buselaphus* (Pallas, 1766)	JN632593	Hassanin et al. 2012
**Ruminantia**	Pecora	Bovidae Antilopinae	*Antilope cervicapra* (Linnaeus, 1758)	JN632598	Hassanin et al. 2012
**Ruminantia**	Pecora	Bovidae Antilopinae	*Gazella gazella* (Pallas, 1766)	JN632640	Hassanin et al. 2012
**Ruminantia**	Pecora	Bovidae Antilopinae	*Neotragus moschatus* Von Dueben, 1846	JN632669	Hassanin et al. 2012
**Ruminantia**	Pecora	Bovidae Antilopinae	*Ourebia aurebi* (Zimmermann, 1783)	JN632680	Hassanin et al. 2012
**Ruminantia**	Pecora	Bovidae Antilopinae	*Pantholops hodgsonii* (Abel, 1826)	DQ191826	Xu et al. 2005
**Ruminantia**	Pecora	Bovidae Antilopinae	*Procapra gutturosa* (Pallas, 1777)	JN632689	Hassanin et al. 2012
**Ruminantia**	Pecora	Bovidae Antilopinae	*Raphicerus campestris* (Thunberg, 1811)	JN632693	Hassanin et al. 2012
**Ruminantia**	Pecora	Bovidae Bovinae	*Bos taurus* Linnaeus, 1758	AY526085	Unpublished; Chung and Ha
**Ruminantia**	Pecora	Bovidae Bovinae	*Boselaphus tragocamelus* (Pallas, 1766)	EF536350	Hassanin et al. 2012
**Ruminantia**	Pecora	Bovidae Bovinae	*Bubalus bubalis* (Linnaeus, 1758)	AF547270	Unpublished; Verma et al
**Ruminantia**	Pecora	Bovidae Bovinae	*Syncerus cafer* (Sparrman, 1779)	EF536353	Hassanin et al. 2012
**Ruminantia**	Pecora	Bovidae Bovinae	*Tragelaphus oryx* (Pallas, 1766)	JN632704	Hassanin et al. 2012
**Ruminantia**	Pecora	Bovidae Caprinae	*Capra hircus* Linnaeus, 1758	GU295658	Hassanin et al. 2010
**Ruminantia**	Pecora	Bovidae Caprinae	*Ovibos moschatus* (Zimmermann, 1780)	FJ207536	Hassanin et al. 2009
**Ruminantia**	Pecora	Bovidae Caprinae	*Ovis aries* Linnaeus, 1758	AF010406	Hiendleder et al. 1998
**Ruminantia**	Pecora	Bovidae Cephalophinae	*Cephalophus natalensis* A. Smith, 1834	JN632618	Hassanin et al. 2012
**Ruminantia**	Pecora	Bovidae Hippotraginae	*Oryx gazzella* (Linnaeus, 1758)	JN632678	Hassanin et al. 2012
**Ruminantia**	Pecora	Bovidae Reduncinae	*Redunca fulvorufula* (Afzelius, 1815)	JN632695	Hassanin et al. 2012
**Ruminantia**	Pecora	Cervidae Capreolinae	*Alces alces* (Linnaeus, 1758)	JN632595	Hassanin et al. 2012
**Ruminantia**	Pecora	Cervidae Capreolinae	*Capreolus capreolus* (Linnaeus, 1758)	JN632610	Hassanin et al. 2012
**Ruminantia**	Pecora	Cervidae Cervinae	*Cervus elaphus* Linnaeus, 1758	AB245427	Unpublished; Wada et al
**Ruminantia**	Pecora	Cervidae Cervinae	*Dama dama dama* (Linnaeus, 1758)	JN632629	Hassanin et al. 2012
**Ruminantia**	Pecora	Cervidae Muntiacinae	*Muntiacus muntjak* (Zimmermann, 1780)	AY225986	Unpublished; Shi et al
**Ruminantia**	Pecora	Giraffidae	*Giraffa camelopardalis* (Linnaeus, 1758)	JN632645	Hassanin et al. 2012
**Ruminantia**	Pecora	Giraffidae	*Okapia johnstoni (*P. L. Sclater, 1901)	JN632674	Hassanin et al. 2012
**Ruminantia**	Pecora	Moschidae	*Moschus moschiferus* Linnaeus, 1758	JN632662	Hassanin et al. 2012
**Ruminantia**	Tragulina	Tragulidae	*Hyemoschus aquaticus* (Ogilby, 1841)	JN632650	Hassanin et al. 2012
**Ruminantia**	Tragulina	Tragulidae	*Tragulus kanchil* Raffles, 1821	JN632709	Hassanin et al. 2012
		Hippopotamidae	*Hexaprotodon liberiensis* (Morton, 1849)	JN632625	Hassanin et al. 2012
		Hippopotamidae	*Hippopotamus amphibius* Linnaeus, 1758	AJ010957	Ursing and Arnason 1998

Table S1. List of taxa, accession number in GenBank and reference (2/3)

**Cetacea**	Mysticeti	Balaenidae	*Balaena mysticetus* Linnaeus, 1758	AJ554051	Arnason et al. 2004
**Cetacea**	Mysticeti	Balaenidae	*Eubalaena australis* (Gray, 1821)	AP006473	Sasaki et al. 2005
**Cetacea**	Mysticeti	Balaenidae	*Eubalaena japonica* (Lacépède, 1818)	AP006474	Sasaki et al. 2005
**Cetacea**	Mysticeti	Balaenopteridae	*Balaenoptera acutorostrata* Lacépède, 1804	AJ554054	Arnason et al. 2004
**Cetacea**	Mysticeti	Balaenopteridae	*Balaenoptera bonaerensis* Burmeister, 1867	AP006466	Sasaki et al. 2005
**Cetacea**	Mysticeti	Balaenopteridae	*Balaenoptera borealis* Lesson, 1828	AP006470	Sasaki et al. 2005
**Cetacea**	Mysticeti	Balaenopteridae	*Balaenoptera brydei* Olsen, 1913	AP006469	Sasaki et al. 2005
**Cetacea**	Mysticeti	Balaenopteridae	*Balaenoptera edeni* Anderson, 1879	AB201258	Sasaki et al. 2006
**Cetacea**	Mysticeti	Balaenopteridae	*Balaenoptera musculus* (Linnaeus, 1758)	X72204	Arnason and Gullberg 1993
**Cetacea**	Mysticeti	Balaenopteridae	*Balaenoptera omurai* Wada et al., 2003	AB201256	Sasaki et al. 2006
**Cetacea**	Mysticeti	Balaenopteridae	*Balaenoptera physalus* (Linnaeus, 1758)	X61145	Valverde et al. 1994
**Cetacea**	Mysticeti	Balaenopteridae	*Megaptera novaeangliae* (Borowski, 1781)	AP006467	Sasaki et al. 2005
**Cetacea**	Mysticeti	Eschrichtiidae	*Eschrichtius robustus* (Lilljeborg, 1860)	AJ554053	Arnason et al. 2004
**Cetacea**	Mysticeti	Neobalaenidae	*Caperea marginata* (Gray, 1846)	AJ554052	Arnason et al. 2004
**Cetacea**	Odontoceti	Delphinidae	*Cephalorhynchus heavisidii* (Gray, 1828)	JN632624	Hassanin et al. 2012
**Cetacea**	Odontoceti	Delphinidae	*Delphinus capensis* Gray, 1828	EU557094	Xiong et al. 2009
**Cetacea**	Odontoceti	Delphinidae	*Feresa attenuata* Gray, 1874	JF289171	Vilstrup et al. 2011
**Cetacea**	Odontoceti	Delphinidae	*Globicephala macrorhynchus* Gray, 1846	JF339976	Vilstrup et al. 2011
**Cetacea**	Odontoceti	Delphinidae	*Globicephala melas* (Traill, 1809)	JF339972	Vilstrup et al. 2011
**Cetacea**	Odontoceti	Delphinidae	*Grampus griseus* (G. Cuvier, 1812)	EU557095	Xiong et al. 2009
**Cetacea**	Odontoceti	Delphinidae	*Lagenorhynchus albirostris* (Gray, 1846)	AJ554061	Arnason et al. 2004
**Cetacea**	Odontoceti	Delphinidae	*Orcaella brevirostris* (Owen in Gray, 1866)	JF289177	Vilstrup et al. 2011
**Cetacea**	Odontoceti	Delphinidae	*Orcaella heinsohni* Beasley, Robertson and Arnold, 2005	JF339977	Vilstrup et al. 2011
**Cetacea**	Odontoceti	Delphinidae	[a]*Orcinus orca* (Ant_A1) Linnaeus, 1758	GU187217	Morin et al. 2010
**Cetacea**	Odontoceti	Delphinidae	[b]*Orcinus orca* (Ant_B1) Linnaeus, 1758	GU187215	Morin et al. 2010
**Cetacea**	Odontoceti	Delphinidae	[c]*Orcinus orca* (Ant_C1) Linnaeus, 1758	GU187210	Morin et al. 2010
**Cetacea**	Odontoceti	Delphinidae	[d]*Orcinus orca* (CNPNRAL) Linnaeus, 1758	GU187189	Morin et al. 2010
**Cetacea**	Odontoceti	Delphinidae	[e]*Orcinus orca* (ENAHN1) Linnaeus, 1758	GU187178	Morin et al. 2010
**Cetacea**	Odontoceti	Delphinidae	[f]*Orcinus orca* (ENPOAL2) Linnaeus, 1758	GU187201	Morin et al. 2010
**Cetacea**	Odontoceti	Delphinidae	[g]*Orcinus orca* (WNPTRU1) Linnaeus, 1758	GU187159	Morin et al. 2010
**Cetacea**	Odontoceti	Delphinidae	*Peponocephala electra* (Gray, 1846)	JF289175	Vilstrup et al. 2011
**Cetacea**	Odontoceti	Delphinidae	*Pseudorca crassidens* (Owen, 1846)	JF289173	Vilstrup et al. 2011
**Cetacea**	Odontoceti	Delphinidae	*Sousa chinensis* (Osbeck, 1765)	EU557091	Xiong et al. 2009
**Cetacea**	Odontoceti	Delphinidae	*Stenella attenuata* (Gray, 1846)	EU557096	Xiong et al. 2009
**Cetacea**	Odontoceti	Delphinidae	*Stenella coeruleoalba* (Meyen, 1833)	EU557097	Xiong et al. 2009
**Cetacea**	Odontoceti	Delphinidae	*Tursiops aduncus* (Ehrenberg, 1833)	EU557092	Xiong et al. 2009
**Cetacea**	Odontoceti	Delphinidae	*Tursiops australis* Charlton-Robb et al., 2011	KF570364	Moura et al. 2013
**Cetacea**	Odontoceti	Delphinidae	*Tursiops truncatus* (Montagu, 1821)	EU557093	Xiong et al. 2009
**Cetacea**	Odontoceti	Iniidae	*Inia geoffrensis* (Blainville, 1817)	AJ554059	Arnason et al. 2004
**Cetacea**	Odontoceti	Lipotidae	*Lipotes vexillifer* Miller, 1918	AY789529	Yan et al. 2005
**Cetacea**	Odontoceti	Monodontidae	*Monodon monoceros* Linnaeus, 1758	AJ554062	Arnason et al. 2004

[a-g], sequences representative of the main clades identified within the *Orcinus orca* complex (Morin et al. 2010)

Table S1. List of taxa, accession number in GenBank and reference (3/3)

**Cetacea**	Odontoceti	Phocoenidae	***Neophocaena asiaeorientalis* Pilleri & Gihr, 1972	KP170488	Unpublished; Liu et al.
**Cetacea**	Odontoceti	Phocoenidae	*Neophocaena phocaenoides* (G. Cuvier, 1829)	KC777291	Unpublished; Xu et al
**Cetacea**	Odontoceti	Phocoenidae	*Phocoena phocoena* (Linnaeus, 1758)	AJ554063	Arnason et al. 2004
**Cetacea**	Odontoceti	Kogidae	*Kogia breviceps* (Blainville, 1838)	AJ554055	Arnason et al. 2004
**Cetacea**	Odontoceti	Physeteridae	*Physeter macrocephalus* Linnaeus, 1758	AJ277029	Arnason et al. 2000
**Cetacea**	Odontoceti	Platanistidae	*Platanista minor* Owen, 1853	AJ554058	Arnason et al. 2004
**Cetacea**	Odontoceti	Pontoporidae	*Pontoporia blainvillei* (Gervais and d'Orbigny, 1844)	AJ554060	Arnason et al. 2004
**Cetacea**	Odontoceti	Ziphiidae	*Berardius bairdii* Stejneger, 1883	AJ554057	Arnason et al. 2004
**Cetacea**	Odontoceti	Ziphiidae	*Hyperoodon ampullatus* (Forster, 1770)	AJ554056	Arnason et al. 2004
**Cetacea**	Odontoceti	Ziphiidae	*Mesoplodon densirostris* (Blainville, 1817)	KF032861	Unpublished; Morin et al
**Cetacea**	Odontoceti	Ziphiidae	*Mesoplodon europaeus* (Gervais, 1855)	KC776688	Unpublished; Morin et al
**Cetacea**	Odontoceti	Ziphiidae	***Mesoplodon grayi* von Haast, 1876	KF981442	Thompson et al 2015.
**Cetacea**	Odontoceti	Ziphiidae	*** *Mesoplodon ginkgodens* Nishiwaki and Kamiya, 1958	KR534596	Yao et al 2015
**Cetacea**	Odontoceti	Ziphiidae	*Ziphius cavirostris* G. Cuvier, 1823	**LN997430**	**this paper**

******, species used only in selected comparisons and not fully implemented in the analyses; *******, this species become available too late to be implemented in the analyses**.**

References

Arnason U, Gullberg, A. 1993 Comparison between the complete mtDNA sequences of the blue and the fin whale, two species that can hybridize in nature**.** *J. Mol. Evol.* **37**, 312-322.

Arnason U, Gullberg A, Gretarsdottir S, Ursing B, Janke A. 2000 The mitochondrial genome of the sperm whale and a new molecular reference for estimating eutherian divergence dates. *J. Mol. Evol.* **50**, 569-578.

Arnason U., Gullberg A, Janke A. 2004 Mitogenomic analyses provide new insights into cetacean origin and evolution. *Gene* **333**, 27-34.

Di Rocco F, Zambelli A, Mate L, and Vidal-Rioja L. 2010 The complete mitochondrial DNA sequence of the guanaco (*Lama guanicoe*): comparative analysis with the vicuna (*Vicugna vicugna*) genome**.** *Genetica* **138**, 813-818.

Hassanin A, Ropiquet A, Couloux A, Cruaud C**.** 2009 Evolution of the mitochondrial genome in mammals living at high altitude: new insights from a study of the tribe Caprini (Bovidae, Antilopinae). *J. Mol. Evol.* **68**, 293-310.

Hassanin A, Bonillo C, Nguyen BX, and Cruaud, C**.** 2010 Comparisons between mitochondrial genomes of domestic goat (*Capra hircus*) reveal the presence of numts and multiple sequencing errors**.** *Mitochondrial DNA* **21**, 68-76.

Hassanin A, Delsuc F, Ropiquet A, Hammer C, Jansen van Vuuren B, Matthee C, Ruiz-Garcia M, Catzeflis F, Areskoug V, Nguyen TT, Couloux, A. 2012 Pattern and timing of diversification of Cetartiodactyla (Mammalia, Laurasiatheria), as revealed by a comprehensive analysis of mitochondrial genomes. *C. R. Biol.* **335**, 32-50.

Hiendleder S, Lewalski H, Wassmuth R, Janke A. 1998 The complete mitochondrial DNA sequence of the domestic sheep (*Ovis aries*) and comparison with the other major ovine haplotype. *J. Mol. Evol.* **47**, 441-448.

Ji R, Cui P, Ding F, Geng J, Gao H, Zhang H, Yu J, Hu S, Meng H. 2009 Monophyletic origin of domestic bactrian camel (*Camelus bactrianus*) and its evolutionary relationship with the extant wild camel (*Camelus bactrianus ferus*). *Anim. Genet.* **40**, 377-382.

Morin PA, Archer FI, Foote AD, Vilstrup J, Allen EE, Wade P, Durban J, Parsons K, Pitman R, Li L, Bouffard P, Abel Nielsen SC, Rasmussen M, Willerslev E, Gilbert, MT, Harkins T. 2010 Complete mitochondrial genome phylogeographic analysis of killer whales (*Orcinus orca*) indicates multiple species. *Genome Res.* **20**, 908-916.

Moura AE, Nielsen SC, Vilstrup JT, Moreno-Mayar JV, Gilbert MT, Gray HW, Natoli A, Moller L, Hoelzel AR. 2013 Recent diversification of a marine genus (*Tursiops* spp.) tracks habitat preference and environmental change. *Syst. Biol.* **62**, 865-877.

Sasaki T, Nikaido M, Hamilton H, Goto M, Kato H, Kanda N, Pastene LA, Cao Y, Fordyce RE, Hasegawa M, Okada N. 2005 Mitochondrial phylogenetics and evolution of mysticete whales. *Syst. Biol.* **54**, 77-90.

Sasaki T, Nikaido M, Wada S, Yamada, TK, Cao Y, Hasegawa M, Okada, N. 2006 *Balaenoptera omurai* is a newly discovered baleen whale that represents an ancient evolutionary lineage. *Mol. Phylogenet. Evol.* **41**, 40-52.

Thompson KF, Patel S, Williams L, Tsai P, Constantine R, Baker CS, Millar CD. 2015 High coverage of the complete mitochondrial genome of the rare Gray's beaked whale (*Mesoplodon grayi*) using Illumina next generation sequencing. Mitochondrial DNA. In press; doi:10.3109/19401736.2013.878908

Ursing BM, Arnason U. 1998 Analyses of mitochondrial genomes strongly support a hippopotamus-whale clade. *Proc. R. Soc. Lond., B, Biol. Sci*. **265**, 2251-2255.

Ursing BM, Slack KE, Arnason U. 2000 Subordinal artiodactyl relationships in the light of phylogenetic analysis of 12 mitochondrial protein-coding genes. *Zool. Scr.* **29**, 83-88.

Valverde JR, Marco R, Garesse, R. 1994 A conserved heptamer motif for ribosomal RNA transcription termination in animal mitochondria. *Proc. Natl. Acad. Sci. U.S.A.* **91**, 5368-5371.

Vilstrup JT, Ho SY, Foote AD, Morin PA, Kreb D, Krutzen M, Parra GJ, Robertson KM, de Stephanis R, Verborgh P, Willerslev E, Orlando L, Gilbert MT. 2011 Mitogenomic phylogenetic analyses of the Delphinidae with an emphasis on the Globicephalinae. *BMC Evol. Biol.* **11**, 65.

Wu GS, Yao YG, Qu KX, Ding ZL, Li H, Palanichamy MG, Duan, ZY, Li N, Chen YS, Zhang YP. 2007 Population phylogenomic analysis of mitochondrial DNA in wild boars and domestic pigs revealed multiple domestication events in East Asia. *Genome Biol.* **8**, R245.

Xiong Y, Brandley MC, Xu S, Zhou K, Yang G. 2009 Seven new dolphin mitochondrial genomes and a time-calibrated phylogeny of whales. *BMC Evol. Biol.* **9**, 20.

Xu SQ, Yang YZ, Zhou J, Jing GE, Chen YT, Wang J, Yang HM, Wang J, Yu J, Zheng XG, Ge RL. 2005 A mitochondrial genome sequence of the Tibetan antelope (*Pantholops hodgsonii*). *Genomics Proteomics Bioinformatics* **3**, 5-17.

Xu X, Arnason U. 1994 The complete mitochondrial DNA sequence of the horse, *Equus caballus*: extensive heteroplasmy of the control region. *Gene* **148**, 357-362.

Xu X, Arnason, U. 1997 The complete mitochondrial DNA sequence of the white rhinoceros, *Ceratotherium simum*, and comparison with the mtDNA sequence of the Indian rhinoceros, *Rhinoceros unicornis*. *Mol. Phylogenet. Evol.* **7**, 189-194.

Yan J, Zhou K, Yang G. 2005 Molecular phylogenetics of 'river dolphins' and the baiji mitochondrial genome. *Mol. Phylogenet. Evol.* **37**, 743-750.

Yao CJ, Chen CH, Hsiao CD. 2015The complete mitogenome of Ginkgo-toothed beaked whale (*Mesoplodon ginkgodens*) (Chordata: Ziphiidae). *Mitochondrial DNA*, 1-2. In press; doi:10.3109/19401736.2015.1053122.
