# Supplementary material for: Back to Water: Signature of Adaptive Evolution in Cetacean Mitochondrial tRNAs
Source: PLoS One. 2016 Jun 23;11(6):e0158129. doi: 10.1371/journal.pone.0158129 (PMC4919058; doi:10.1371/journal.pone.0158129)
Supplement: S2 Table — (PDF) [file pone.0158129.s012.pdf]

Table S2. Intraspecific CSBPSs identified in some Cetacea

Balaenoptera physalus (152 mtDNAs)	Thirteen tRNAs exhibited intraspecific HCBCs and mismatches in *B. physalus*. Only mismatches were identified in *trnD* (1 seq, 1 mismatch), *trnL2* (5 seqs, 1 mismatch), *trnR* (2 seq, 1 mismatch), *trnS1* (1seq, 1 mismatch), and *trnT* (3 seqs, 3 mismatches). A combination of HCBCs and mismatches was found in *trnF* (4 seqs, 2 HCBC and 2 mismatches) *trnH* (20 seqs with 4 mismatches, 12 seqs with 1 HCBC), *trnP* (2 seqs, 1 HCBC + 1 mismatch). Only HCBCs were identified in *trnA* (4 seqs, 3 HCBCs), *trnC* (2 seqs, 1 HCBC), *trnE* (1 seq, 1 HCBC) *trnL1* (14 seqs, 1 HCBC) *trnN* (1 seq, 1 HCBC). The number of sequences interested by a single CSBPS ranged from 1 to 12.
Mesoplodon densirostris (11 mtDNAs)	Six tRNAs of *M. densirostris* presented CSBPSs i.e. *trnL2* (7 seqs, 1HCBC), *trnQ* (1 seq, 1 HCBC), *trnR* (7 seqs, 1 HCBC), trnS2 (3 seqs, 2 HCBCs), trnV (3 seqs, 2 mismatches), and trnW (7 seqs, 2HCBCs). In *M. europaeus* only trnL2 showed a CSBP (1 seq, 1 HCBC).
Mesoplodon europaeus (11 mtDNAs)	In *M. europaeus* only *trnL2* showed a CSBP (1 seq, 1 HCBC).
Orcinus orca complex (86 mtDNAs)	In the *O. orca* complex we report here only the intraspecific CSBPSs not present in the seven sequences included in 94T-set. Seven tRNAs shown extra CSBPSs i.e. *trnC* (1 seq, 1 HCBC), *trnD* (2 seqs, 1 HCBC), *trnG* (1 seq, 1 mismatch), *trnK* (2 seqs, 1 HCBC), *trnS1* (2 seqs with 1 mismatch, 1 seq with 1 HCBC), *trnT* (1 seq, 1 HCBC), *trnW* (34 seqs, 1 HCBC).
Physeter microcephalus (18 mtDNAs)	Four tRNAs of *P. macrocephalus* exhibited extra CSBPSs i.e. *trnA* (1 seq, 1 HCBC), *trnF* (1 seq, 1 HCBC), *trnS2* (7 seq, 1 HCBC), and *trnT* (1 seq, 1 mismatch).
Tursiops aduncus (20 mtDNAs)	In *T. aduncus*, one HCBC was found in *trnF* (5 seqs) and *trnT* (10 seqs).
Tursiops australis (8 mtDNAs)	In *T. australis* one mismatch was identified in *trnS1* (1 seq).
Tursiops truncatus (48 mtDNAs)	In *T. truncatus* CSBPSs were found in *trnD* (30 seqs, 1 HCBC), *trnF* (2 seqs, 1 mismatch), *trnL2* (1 seq, 1 MISM), *trnR* (2 seqs, 2 mismatches), and *trnV* (1 seq, 1 HCBC).
Ziphius cavirostris (20 mtDNAs)	In *Z. cavirostris* a single HCBC was identified in *trnD* (8 seqs), *trnH* (2 seqs), *trnK* (2 seqs) *trnL2* (3 seqs), *trnS1* (1 seq), and *trnT* (1 seq).

The intraspecific CSBPSs are described following this scheme: tRNA involved (number of sequences (seqs) showing the CSBPS, number and type of CSBPSs associated).

CSBPS, change of sequence in a base pair of a stem; HCBC, hemi-compensatory base change in a base pair of a stem; mismatch, a mismatch in a base pair of a stem.
