# Supplementary material for: Back to Water: Signature of Adaptive Evolution in Cetacean Mitochondrial tRNAs
Source: PLoS One. 2016 Jun 23;11(6):e0158129. doi: 10.1371/journal.pone.0158129 (PMC4919058; doi:10.1371/journal.pone.0158129)
Supplement: S1 tRNA Multiple Alignments — (PDF) [file pone.0158129.s013.pdf]

trnA (ALA) multiple alignment

```
                                            10        20        30        40        50        60        70
                                             |         |         |         |         |         |         |
                                    1234567  1234      4321 12345  ant  54321    12345       543217654321d
                                                            *               *
Ceratotherium simum trnA            GAGGGTTTAGCTTAA-TTAAAGTGTTTGATTTGCATTCAGTTGATGTAAGATAGAGTCTTGCAGCCCTTA
Equus caballus trnA                 AAGGGCTTAGCTTAA-TAAAAGAGTTTGATTTGCGTTCAATTGATGTGAGATAGAGTCTCGCAGTCCTTA
Pecari tajacu trnA                  GGGGATTTAGCTTAA-TTAAAGTGTTTGATTTGCGTTCAATTGATGTAGGA-GTAGTCCTGCAATCCTTA
Phacochoerus africanus trnA         GAGGACTTAGCTTAA-TTAAAGTGTTTGATTTGCATTCAATTGATGTAGGAT-GGGTCCTGCAGTCCTTA
Potamochoerus porcus trnA           GAGGACTTAGCTTAA-TTAAAGTGTTTGATTTGCATTCAATTGATGTAGGAT-GAGTCCTGCAGCCCTTA
Sus scrofa trnA                     GAGGATTTAGCTTAA-TTAAAGTGTTTGATTTGCATTCAATTGATGTAGGAT-AAGTCCTGCAGTCCTTA
Camelus bactrianus trnA             AAGGGCTTAGCTTAA-TTAAAGCAGTTGGTTTGCATTCAATTGATGTAGGATAGAGTCTTGCAGTCCTTA
Camelus dromedarius trnA            AAGGGCTTAGCTTAA-TTAAAGCAGTTGGTTTGCATTCAATTGATGTAGGATAGAGTCTTGCAGTCCTTA
Lama guanicoe trnA                  AAGGGCTTAGCTTAA-TTAAAGTAGTTGATTTGCATTCAATTGATGTAGGATAGAGTCTTGCAGTCCTTA
Vicugna pacos trnA                  AAGGGCTTAGCTTAA-TTAAAGTAGTTGATTTGCATTCAATTGATGTAGGATAGAGTCTTGCAGTCCTTA
Hyemoschus aquaticus trnA           GAGGATTTAGCTTAA-TTAAAGTAATTGGTTTGCATTCAGTTGATGTAAGATATGGTCTTGCAATCCTTA
Tragulus kanchil trnA               GGGGATTTAGCTTAA-TTAAAGTGGTCGATTTGCATTCGATTGATGTAAGATGAAGTCTTGCAGTCCTTA
Giraffa camelopardalis trnA         GAGGATTTAGCTTAA-TTAAAGTGGTTGATTTGCATTCAATTGATGTAAGGTATAGTCTTGCAATCCTTA
Okapia johnstoni trnA               GAGGATTTAGCTTAA-TTAAAGTGGTTGATTTGCATTCAATTGATGTAAGATATAGTCTTGCAGTCCTTA
Antilocapra americana trnA          AAGGATTTAGCTTAA-TAAAAGT GTTGATTTGCATTCAATTGATGTAAGATGAGGTCTTGCAATCCTTA
Moschus moschiferus trnA            GAGGACTTAGCTTAA-TTAAAGTAATTGATTTGCATTCAGTTGATGTAAGATATAGTCTTGCAGTCCTTA
Muntiacus muntjak trnA              GAGGATTTAGCTTAA-TTAAAGTGGTTGATTTGCATTCAATTGATGTAAGATATGGTCTTGCAGTCCTTA
Capreolus capreolus trnA            GAGGATTTAGCTTAA-TTAAAGTGGTTGATTTGCATTCAATTGATGTAAGATGTGGTCTTGCAATCCTTA
Alces alces trnA                    GAGGATTTAGCTTAA-TTAAAGTAGTTGATTTGCATTCAACTGATGTAAGATGTGGTCTTGCAATCCTTA
Cervus elaphus trnA                 GAGGATTTAGCTTAA-TTAAAGTGGTTGATTTGCATTCAATTGATGTAAGATATGGTCTTGCAATCCTTA
Dama dama trnA                      GAGGATTTAGCTTAA-TTAAAGTGGTTGATTTGCATTCAATTGATGTAAGATATGGTCTTGCAATCCTTA
Boselaphus tragocamelus trnA        GAGGATTTAGCTTAA-TTAAAGTGGTTGATTTGCATTCAATTGATGTAAGATACAGTCTTGCAATCCTTA
Tragelaphus oryx trnA               GAGGATTTAGCTTAA-TTAAAGTGATTGATTTGCGTTCAGTTGATGTAAGGTATAGTCTTGCAATCCTTA
Bos taurus trnA                     GAGGATTTAGCTTAA-TTAAAGTGGTTGATTTGCATTCAATTGATGTAAGGTGTAGTCTTGCAATCCTTA
Bubalus bubalis trnA                GAGGATTTAGCTTAA-TTAAAGTGGTTGATTTGCATTCAATTGATGTAAGATATGGTCTTGCAATCCTTA
Syncerus caffer trnA                GAGGATTTAGCTTAA-TTAAAGTGGTTGATTTGCATTCAATTGATGTAAGGTATAGTCTTGCAATCCTTA
Neotragus moschatus trnA            GAGGACTTAGTTTAA-TTAAAGCGGTTGATTTGCATTCAATTGATGTAAGATGTAGTCTTGCAGTCCTTA
Alcelaphus buselaphus trnA          GAGGACTTAGCTTAA-TGAAAGTGGTTGATTTGCGTTCAATTGATGTAAGGTGTAGTCTTGCAGTCCTTA
Oryx gazella trnA                   GAGGACTTAGCTTAA-TTAAAGTGGTTGATTTGCATTCAGTTGATGTAAGATATAGTCTTGCAGTCCTTA
Pantholops hodgsonii trnA           GAGGATTTAGCTTAA-TTAAAGTGGTTGATTTGCATTCAATTGATGTAAGATATGATCTTGCAGTCCTTA
Ovibos moschatus trnA               GAGGATTTAGCTTAA-TTAAAGTGGTTGATTTGCATTCAATTGATGTAAGATGTGGTCTTGCAATCCTTA
Capra hircus trnA                   AAGGATTTAGCTTAA-TTAAAGTGGTTGATTTGCATTCAATTGATGTAAGATATGGTCTTGCAGTCCTTA
Ovis aries trnA                     GAGGATTTAGCTTAA-TTAAAGTGGTTGATTTGCATTCAATTGATGTAAGATGTAGTCTTGCAGTCCTTA
Cephalophus natalensis trnA         GAGGACTTAGCTTAA-TTAAAGTGGTTGATTTGCATTCAATTGATGTAAGATATGATCTTGCAGTCCTTA
Redunca fulvorufula trnA            GAGGACTTAGCTTAA-TTAAAGTGTTTGATTTGCGTTCAGTTGATGTAAGATATAGTCTTGCAGTCCTTA
Ourebia ourebi trnA                 GAGGACTTAGCTTAA-TTAAAGTGGTTGATTTGCGTTCAGTTGATGTAAGATGTAATCTTGCAGTCCTTA
Antilope cervicapra trnA            GAGGACTTAGCTTAA-TTAAAGTGGTTGATTTGCGTTCAGTTGATGTAAGATATAATCTTGCAGTCCTTA
Gazella gazella trnA                GAGGACTTAGCTTAA-TTAAAGTGGTTGATTTGCGTTCAGTTGATGTAAGATGTAATCTTGCAGTCCTTA
Procapra gutturosa trnA             GAGGACTTAGCTTAA-TAAAAGTGGTTGATTTGCATTCAATTGATGTAAGATATAATCTTGCAGTCCTTA
Raphicerus campestris trnA          GAGGACTTAGCTTAA-TTAAAGTGGTTGATTTGCATTCAATTGATGTAAGATATAATCTTGCAGTTCTTA
Hexaprotodon liberiensis trnA       GGGGACTTAGCTTAA-TAAAAGCAGTTGAGTTGCATTCAATTGATGTGAGGCGTGATCTTGCAGTCTCTA
Hippopotamus amphibius trnA         AGGGACTTAGCTTAA-TAAAAGCAGTTGAGTTGCATTCAATTGATGTGAGGTGCGGTCTTGCAGTCTCTA
Eubalaena australis trnA            GAGGATTTAGCTTAA-TTAAAGTGTTTGATTTGCATTCAATTGATGTAAGATATAGTCTTGCAGTCCTTA
Eubalaena japonica trnA             GAGGATTTAGCTTAA-TTAAAGTGTTTGATTTGCATTCAATTGATGTAAGATATAGTCTTGCAGTCCTTA
Balaena mysticetus trnA             GAGGATTTAGCTTAA-TTAAAGTGTTTGATTTGCATTCAATTGATGTAAGATATAGTCTTGCAGTCCTTA
Caperea marginata trnA              GAGGATTTAGCTTAA-TTAAAGTGTTTGATTTGCATTCAATTGATGTAAGA-ATAGTCTTGCAGTCCTTA
Eschrichtius robustus trnA          GAGGATTTAGCTTAA-TTAAAGCGTTTGGTTTGCGTCCAATTGATGTGAGATATAGTCTTGCAGTCCTTA
Balaenoptera acutorostrata trnA     GAGGATTTAGCTTAA-TTAAAGTGTTTGATTTGCATTCAATTGATGTAAGATATAGTCTTGCAGTCCTTA
Balaenoptera bonaerensis trnA       GAGGATTTAGCTTAA-TTAAAGCGTTTGATTTGCATTCAATTGATGTAAGATATAGTCTTGCAGTCCTTA
Balaenoptera physalus trnA          GAGGATTTAGCTTAA-TTAAAGCGTTTGACTTGCATTCAATTGATGTAAGATATAGTCTTGCAGTCCTTA
Megaptera novaeangliae trnA         GAGGATTTAGTTTAA-TTAAAGCGTTTGACTTGCATTCAAATGATGTAAGATATAGTCTTGCAGTCCTTA
Balaenoptera musculus trnA          GAGGATTTAGCTTAA-TTAAAGTGTTTGATTTGCATTCAATTGATGTAAGATATAGTCTTGCAGTCCTTA
Balaenoptera omurai trnA            GAGGATTTAGCTTAA-TTAAAGTGTTTGATTTGCATTCAATTGATGTAAGATATAGTCTTGCAATCCTTA
Balaenoptera borealis trnA          GAGGATTTAGCTTAA-TTAAAGTGTTTGATTTGCATTCAATTGATGTAAGATATAGTCTTGCAATCCTTA
Balaenoptera brydei trnA            GAGGATTTAGCTTAA-TTAAAGTGTTTGATTTGCATTCAATTGATGTAAGATATGGTCTTGCAATCCTTA
Balaenoptera edeni trnA             GAGGATTTAGCTTAA-TTAAAGTGTTTGATTTGCATTCAATTGATGTAAGATATGGTCTTGCAATCCTTA
Kogia breviceps trnA                GAGGGCTTAGCTTAA-TTAAAGTGTCTGATTTGCATTCAGTTGATGTAAGATATAGTCTTGCAGTCCTTA
Physeter macrocephalus trnA         GAGGACTTGACTTAA-TTAAAGTGTTTGATTTGCATTCAGTTGATGTAAGATATAGTCTTGCAGTCCTTG
Platanista minor trnA               GAGGATTTAGCTTAA-TTAAAGTATTTGATTTGCGTTCAGATGATGTAAGATGTAGTCTTGCAATCCTTA
Ziphius cavirostris trnA            GAGGACTTAGCTTAA-TGAAAGTGTTTGATTTGCATTCAATTGATGTAAGATATGATCTTGCAGTCCTTA
Mesoplodon densirostris trnA        GAGGACTTAGCTTAA-TAAAAGTGTTTGATTTGCATTCAATTGATGTAAGATATGATCTTGCAGTCCTTA
Mesoplodon europaeus trnA           GAGGACTTAGCTTAA-TTAAAGTGTTTGATTTGCGTTCAATTGATGTGAGATATGATCTTGCAGTCCTTG
**Mesoplodon grayi trnA             GAGGACTTAGCTTAA-TAAAAGTGTTTGATTTGCGTTCAATTGATGTGAGATATGATCTTGCAGTCCTTA
Berardius bairdii trnA              GAGGACTTAGCTTAA-TTAAAGTGTTTGATTTGCATTCAATTGATGTAAGGTATAGTCTTGCAGTCCTTA
Hyperoodon ampullatus trnA          GAGGACTTAGCTTAA-TGAAAGTGTTTGATTTGCGTTCAGTTGATGTAAGATATAATCTTGCAGTCCTTA
Lipotes vexillifer trnA             GAGGATTTAGCTTAA-TTAAAGTGTTTGATTTGCATTCAATTGATGTAAGATGAGATCTTGCAATCCTTA
Inia geoffrensis trnA               GAGGATTTAGCTTAAGTTAAAGCGTTTGATTTGCATTCAATTGATGTGAGATGGGATCTTGCAGTCCTTA
Pontoporia blainvillei trnA         GAGGATTTAGCTTAA-TTAAAGCGTTTGATTTGCATTCAATTGATGTGAGGTGTGGTCTTGCAGTCCTTA
Monodon monoceros trnA              GAGGATTTAGCTTAA-TTAAAGTGTTTGATTTGCATTCAATTGATGTGAGATATGGTCTTGCAATCCTTA
**Neophocaena_asiaeorientalis trnA  GAGGATTTAGCTTAA-TTAAAGTGTTTGATTTGCATTCAATTGATGTGAGATATGGTCTTGCAATCCTTA
Neophocaena phocaenoides trnA       GAGGATTTAGCTTAA-TTAAAGTGTTTGATTTGCATTCAATTGATGTGAGATATGGTCTTGCAATCCTTA
Phocoena phocoena trnA              GAGGATTTAGCTTAA-TTAAAGTGTTTGATTTGCATTCAATTGATGTGAGATATGGTCTTGCAATCCTTA
Cephalorhynchus heavisidii trnA     GAGGATTTAGCTTAA-TTAAAGTGTTTGATTTGCATTCAATTGATGTAAGATATAATCTTGCAATCCTTA
Sousa chinensis trnA                GAGGATTTAGCTTAA-TTAAAGTGTTTGATTTGCATTCAATTGATGTAAGATATAGTCTTGCAGTCCTTA
Stenella attenuata trnA             GAGGATTTAGCTTAA-TTAAAGTGTTTGATTTGCATTCAATTGATGTAAGATATAGTCTTGCAATCCTTA
Tursiops australis trnA             GAGGATTTAGCTTAA-TTAAAGTGTTTGATTTGCATTCAATTGATGTAAGATATAGTCTTGCAATCCTTA
Tursiops truncatus trnA             GAGGATTTAGCTTAA-TTAAAGTGTTTGATTTGCATTCAATTGATGTAAGATATAGTCTTGCAATCCTTA
Tursiops aduncus trnA               GAGGATTTAGCTTAA-TTAAAGTGTTTGATTTGCATTCAATTGATGTAAGATATAATCTTGCAATCCTTA
Delphinus capensis trnA             GAGGATTTAGCTTAA-TTAAAGTGTTTGATTTGCATTCAATTGATGTAAGATATAGTCTTGCAATCCTTA
Stenella coeruleoalba trnA          GAGGATTTAGCTTAA-TTAAAGTGTTTGATTTGCATTCAATTGATGTAAGATATAGTCTTGCAATCCTTA
Orcaella brevirostris trnA          GAGGATTTAGCTTAA-TTAAAGTATTTGATTTGCATTCAATTGATGTAAGATATAGTCTTGCAATCCTTA
Orcaella heinsohni trnA             GAGGATTTAGCTTAA-TTAAAGTATTTGATTTGCATTCAATTGATGTAAGATATAGTCTTGCAATCCTTA
Grampus griseus trnA                GAGGATTTAGCTTAA-TTAAAGTGTTTGATTTGCATTCAATTGATGTAAGATATAGTCTTGCAATCCTTA
Pseudorca crassidens trnA           GAGGATTTAGCTTAA-TTAAAGTGTTTGATTTGCAGTCAATTGATGTAAGATATAGTCTTGCAATCCTTA
Feresa attenuata trnA               GAGGATTTAGCTTAA-TTAAAGTGTTTGATTTGCATTCAATTGATGTAAGATATAGTCTTGCAATCCTTA
Peponocephala electra trnA          GAGGATTTAGCTTAA-TTAAAGTGTTTGATTTGCATTCAATTGATGTAAGATATGGTCTTGCAATCCTTA
Globicephala macrorhynchus trnA     GAGGATTTAGCTTAA-TTAAAGTGTTTGATTTGCATTCAATTGATGTAAGATATAGTCTTGCAATCCTTA
Globicephala melas trnA             GAGGATTTAGCTTAA-TTAAAGTGTTTGATTTGCATTCAATTGATGTAAGATATAGTCTTGCAATCCTTA
Lagenorhynchus albirostris trnA     GAGGATTTAGCTTAA-TTAAAGTGTTTGATTTGCATTCAATTGATGTAGGATATAGTCTTGCAATCCTTA
Orcinus orca WNPTRU1 trnA           GAGGATTTAGCTTAA-TTAAAGTGTTTGATTTGCATTCAATTGATGTAGGATATAGTCTTGCAATCCTTA
Orcinus orca AntA1 trnA             GAGGATTTAGCTTAA-TTAAAGTGTTTGATTTGCATTCAATTGATGTAGGATATAGTCTTGCAATCCTTA
Orcinus orca AntB1 trnA             GAGGATTTAGCTTAA-TTAAAGTGTTTGATTTGCATTCAATTGATGTAGGATATAGTCTTGCAATCCTTA
Orcinus orca AntC1 trnA             GAGGATTTAGCTTAA-TTAAAGTGTTTGATTTGCATTCAATTGATGTAGGATATAGTCTTGCAATCCTTA
Orcinus orca ENAHN1 trnA            GAGGATTTAGCTTAA-TTAAAGTGTTTGATTTGCATTCAATTGATGTAGGATATAGTCTTGCAATCCTTA
Orcinus orca CNPNRAL trnA           GAGGATTTAGCTTAA-TTAAAGTGTTTGATTTGCATTCAATTGATGTAGGATATAGTCTTGCAATCCTTA
Orcinus orca ENPOAL2 trnA           GAGGATTTAGCTTAA-TTAAAGTGTTTGATTTGCATTCAATTGATGTAGGATATAGTCTTGCAATCCTTA
                                                            *               *
                                    1234567  1234      4321 12345  ant  54321    12345       543217654321d
                                    0000000  1111      2222 22222  333  33344    44445       5566666666667
                                    1234567  0123      0123 56789  234  78901    67890       8901234567890
```

N, the most common base for the position.

N, half compensatory base change in the stem pair (e.g. T – G vs C – G; A-T vs G-T).

N, N, half compensatory base change in the stem pair exhibiting a mismatch (e.g. T–A vs A–A). Different colours are used to better differentiate the changes.

N, fully compensatory base change in the stem pair exhibiting a mismatch (e.g. C–G vs T–T).

N, type I fully compensatory base change in the stem pair (i.e. purine – pyrimidine vs purine – pyrimidine, e.g. G – C vs A – T)

N, N, type II fully compensatory base change in the stem pair (i.e. purine – pyrimidine vs pyrimidine – purine, e.g. A – T vs T – A). Different colours are used to better differentiate the changes.

N, a mismatch in the in the stem pair; N, substitution pattern not modelled; *, pair in the stem in which a mismatch is prominent; N, N, molecular signature for a taxon.

X, position 1-7 in the acceptor stem; X position 1-4 in the DHU stem; X position 1-5 in the anticodon stem; X position 1-4(5) in the TΨC stem; ant, anticodon; d, discriminator nucleotide.

trnC (CYS) multiple alignment

```
                                                 10        20        30        40        50        60        70
                                                  |         |         |         |         |         |         |
                                         1234567 1234      4321 12345 ant 54321    1234      43217654321d
Ceratotherium simum trnC                 GGCCCTGTGGTGAA-----TT-TCATGTTGAATTGCAAATTCAGAGAAGCAGCTTCAA-TTCTGCCGGGGCTT
Equus caballus trnC                      AGTCCCGTGGTGAA----TTT-TCACATTGAATTGCAAATTCAAAGGAGCAGCTTCAATTTCTGCCGGGGCTT
Pecari tajacu trnC                       AGTCTCGTGGTGAA----TTA-TCATGTTGAATTGCAAATTCAAAGAAGCAGCTTCAA-TTCTGCCGGGGCTT
Phacochoerus africanus trnC              AGTTCCGTGGTGAA----ATA-TCATGTTGAATTGCAAATTCAAAGAAGCAGCTTCAA-TTCTGCCGGGACTT
Potamochoerus porcus trnC                GGTTCCGTAGTGAA----ATA-CCATATTGAATTGCAAATTCAAAGAAGCAGCTTCAA-TTCTGCCGGGACTT
Sus scrofa trnC                          AGTTCCGTGGTGAA-----TA-TCATGTTGAATTGCAAATTCAAAGAAGCAGCTTCAA-TTCTGCCGGGACTT
Camelus bactrianus trnC                  AGCCTTGTGGTGTA-----AA-ACATATTGAATTGCAAATTCAAAGAAGCAGCTTCAAACTCTGCCGGGGCTT
Camelus dromedarius trnC                 AGCCCTGTGGTGTA-----TA-ACATATTGAATTGCAAATTCAAAGAAGCAGCTTCAAACTCTGCCGGGGCTT
Lama guanicoe trnC                       AGTCCTGTGGTGAT-----TA-ACACATTGAATTGCAAGTTCAAAGAAGCAGCTTCAAACTCTGCCGGGGCTT
Vicugna pacos trnC                       AGTCCTGTGGTGAT-----TA-ACACATTGAATTGCAAGTTCAAAGAAGCAGCTTCAAACTCTGCCGGGGCTT
Hyemoschus aquaticus trnC                AGCTCTGTGGTGAA---ATTT-TCATGTTGAATTGCAAATTCAAAGAAGCAGCTTCAAGATCTGCCGGGGCTT
Tragulus kanchil trnC                    AGCCCTGTGGTGAAAAAAGATCTCATATTGAATTGCAAATTCAAAGAAGCAGCTTCAAGCTCTGCCGGGGCTT
Giraffa camelopardalis trnC              GGTCCTGTAGTGAA----TTTCTCACGTTGAATTGCAAATTCAAAGAAGCAGCTTCAA-TTCTGCCGGGGCTT
Okapia johnstoni trnC                    AGTCCTGTAGTGAA----TTT-TCATGTTGAATTGCAAATTCGAAGAAGCAGCTTCAA-TTCTGCCGGGGCTT
Antilocapra americana trnC               AGTCCTGTGGTGAA----TTT-ACACGTTGAATTGCAAATTCGAAGAAGCAGCTTCAAACTCTGCCGGGGCTT
Moschus moschiferus trnC                 AGTCCTGTGGTGAA-----TT-TCACGTTGAATTGCAAATTCAGAGAAGCAGCTTCAA-TTCTGCCGGGGCTT
Muntiacus muntjak trnC                   GGTCCTGTGGTGAA----ATT-TCATGTTGAATTGCAAATTCAAAGAAGCAGCTTCAAACTCTGCCGGGGCTT
Capreolus capreolus trnC                 AGCCCTGTGGTGAA-----TG-TCATGTTGAATTGCAAATTCAAAGAAGCAGCTTCAA-TTCTGCCGGGGCTT
Alces alces trnC                         AGCCCTGTGGTGAA----ATT-TCATGTTGAATTGCAAATTCAAAGAAGCAGCTTCAA-TTCTGCCGGGGCTT
Cervus elaphus trnC                      AGTCCTGTGGTGAA----ATT-TCATGTTGAATTGCAAATTCAAAGAAGCAGCTTCAAACTCTGCCGGGGCTT
Dama dama trnC                           AGTCCTGTGGTGAA-----TT-TCATGTTGAATTGCAAATTCAAAGAAGCAGCTTCAAACTCTGCCGGGGCTT
Boselaphus tragocamelus trnC             AGCCTTGTGGTGAA----ATT-TCACGTTGAATTGCAAATTCAGAGAAGCAGCTTCAA-TTCTGCCGGGGCTT
Tragelaphus oryx trnC                    GGCTCTGTGGTGAA----TTT-ACACGTTGAATTGCAAATTCAGAAAAGCAGCTTTAA-TTCTGCCGGGGCTT
Bos taurus trnC                          AGCCCTGTGGTGAA----TTT-ACACGTTGAATTGCAAATTCAGAGAAGCAGCTTCAA-TTCTGCCGGGGCTT
Bubalus bubalis trnC                     GGCCTTGTGGTGAA----TTT-ACACGTTGAATTGCAAATTCAGAGAAGCAGCTTTAA-TTCTGCCGGGGCTT
Syncerus caffer trnC                     GGCCCTGTAGTGAA----TTT-ACACGTTGAATTGCAAATTCAGAGAAGCAGCTTCAAATTCTGCCGGGGCTT
Neotragus moschatus trnC                 GGCCTTGTGGTGAA----TTT-TCACGCTGAATTGCAAATTCAGAGAAGCAGCT-CAAACTCTGCCGGGGCTT
Alcelaphus buselaphus trnC               AGCCCTGTGGTGAA----TTA-ACATATTGAATTGCAAGTTCAAAGAAGCAGCTTCAAACTCTGCCGGGGCTT
Oryx gazella trnC                        AGCTCCGTGGTGAA----TTA-ACATATTGAATTGCAAATTCAAAGAAGCAGCTTCAA-TTCTGCCGGGGCTT
Pantholops hodgsonii trnC                AGTCCTGTAGTGAA----TTA-ACATATTGAATTGCAAATTCAAAGAAGCAGCTTCAA-CTCTGCCGGGGCTT
Ovibos moschatus trnC                    GGTCCTGTAGTGAA---- A AACATATTGAATTGCAAATTCAAAGAAGCAGCTTTAA-TTCTGCCGGGGCTT
Capra hircus trnC                        AGTCCTGTAGTGAA----TTA-ACATATTGAATTGCAAATTCAAAGAAGCAGCTTCAAACTCTGCCGGGGCTT
Ovis aries trnC                          GGTCCTGTAGTGAA----TTA-ACATATTGAATTGCAAATTCAAAGAAGCAGCTTCAAACTCTGCCGGGGCTT
Cephalophus natalensis trnC              GGCCTTGTGGTGAA----TTA-ACATATTGAATTGCAAATTCAAAGAAGCAGCTTCAA-TTCTGCCGGGGCTT
Redunca fulvorufula trnC                 GGCTCTGTGGTGTG----TTA-ACATATTGAATTGCAAATTCAAAGAAGCAGCTTCAA-TTCTGCCGGGGCTT
Ourebia ourebi trnC                      GGTCCTGTGGTGAA----TTA-ACATATTGAATTGCAAATTCAAAGAAGCAGCTTCAA-TTCTGCCGGGGCTT
Antilope cervicapra trnC                 GGCCTCGTGGTGAA----TTA-ACATATTGAATTGCAAATTCAAAGAAGCAGCTTCAA-TTCTGCCGGGGCTT
Gazella gazella trnC                     GGCCTCGTGGTGAA----TTA-ACATATTGAATTGCAAATTCAAAGAAGCAGCTTCAA-TACTGCCGGGGCTT
Procapra gutturosa trnC                  GGCCCTGTGGTGAA-----TT-ACATATTGAATTGCAAATTCAAAGAAGCAGCTTCAA-TTCTGCCGGGGCTT
Raphicerus campestris trnC               GGTCCTGTGGTGTA----TTAAACACGTTGAATTGCAAATTCAAAGAAGCAGCTTCAA-CTCTGCCGGGGCTT
Hexaprotodon liberiensis trnC            GGTCCTGTAGTGAA----ATATTCATATTGAATTGCAAATTCAAAGAAGCAGCTTCAA-TTCTGCCGGGGCTT
Hippopotamus amphibius trnC              GGTCCTGTAGTGAA----ATATTCATATTGAATTGCAAATTCAAAGAAGCAGCTTCAA-TTCTGCCGGGGCTT
Eubalaena australis trnC                 AGTCCTGTGGTGAA-----TAGTCATTTTGAATTGCAAATTCAAGGAAGCAGCTTCAAATCCTGCCGGGGCTT
Eubalaena japonica trnC                  AGTCCTGTGGTGAA-----TAGTCATTTTGAATTGCAAATTCAAGGAAGCAGCTTCAAATCCTGCCGGGGCTT
Balaena mysticetus trnC                  AGTCCTGTGGTGAA-----TAGTCATTTTGAATTGCAAATTCAAGGAAGCAGCTTCAAATCCTGCCGGGACTT
Caperea marginata trnC                   AGTCCTGTGGTGAA-----TAGTCATTTTGAATTGCAAATTCAAGGAAGCAGCTTCAAATCCTGCCGGGACTT
Eschrichtius robustus trnC               AGTCCTGTGGTGAA-----TGATCATTTTGAATTGCAAATTCAAGGAAGCAGCTTCAAATCCTGCCGGGACTT
Balaenoptera acutorostrata trnC          AGTCCTGTGGTGAA-----TGATCATTTTGAATTGCAAATTCAAGGAAGCAGCTTCAAATCCTGCCGGGACTT
Balaenoptera bonaerensis trnC            AGTCCTGTAGTGAA-----TGATCATTTTGAATTGCAAATTCAAAGAAGCAGCTTCAAATCCTGCCGGGACTT
Balaenoptera physalus trnC               AGTCCTGTGGTGAA-----TGATCATTTTGAATTGCAAATTCAAGGAAGCAGCTTCAAATCCTGCCGGGACTT
Megaptera novaeangliae trnC              AGTCCTGTGGTGAA-----TAATCATTTTGAATTGCAAATTCAAGGAAGCAGCTTCAAATCCTGCCGGGACTT
Balaenoptera musculus trnC               AGTCCTGTGGTGAA-----TAGTCATTTTGAATTGCAAATTCAAGGAAGCAGCTTCAAATCCTGCCGGGACTT
Balaenoptera omurai trnC                 AGTCCTGTGGTGAA-----TAATCATTTTGAATTGCAAATTCAAGGAAGCAGCTTCAAATCCTGCCGGGACTT
Balaenoptera borealis trnC               AGTCCTGTGGTGAA-----TGATCATTTTGAATTGCAAATTCAAGGAAGCAGCTTCAAATCCTGCCGGGACTT
Balaenoptera brydei trnC                 AGTCCTGTGGTGAA-----TGATCATTTTGAATTGCAAATTCAAGGAAGCAGCTTCAAATCCTGCCGGGACTT
Balaenoptera edeni trnC                  GGTCCTGTGGTGAA-----TGATCATTTTGAATTGCAAATTCAAGGAAGCAGCTTCAAATCCTGCCGGGACTT
Kogia breviceps trnC                     AGTCCTGTAGTGAA-----TGATCATGTTGAATTGCAAATTCAAAGGAGCAGCTTCAA-TCCTGCCGGGACTT
Physeter macrocephalus trnC              AGCCTTGTAGTGAA-----TGATCATGTTGAATTCGAAATTCAAAGGAGCAGCTTCAA-TCCTGCCGGGGCTT
Platanista minor trnC                    AGTCCTGTGGTGAA-----TGATCATGTTGAATTCGAAATTCAAAGAAGCAGCTTCAA-TCCTGCCGGGACTT
Ziphius cavirostris trnC                 AGTCCTGTAGTGAA-----TAGTCATTTTGAATTGCAAATTCAAAGAAGCAGCTTCAA-TCCTGCCGGGACTT
Mesoplodon densirostris trnC             AGTCCTGTAGTGAA-----TAGTCATTTTGAATTGCAAATTCAAAGAAGCAGCTTCAA-CCCTGCCGGGACTT
Mesoplodon europaeus trnC                AGTCCTGTAGTGAA-----TAGTCATTTTGAATTGCAAATTCAAAGAAGCAGCTTCAA-TCCTGCCGGGACTT
**Mesoplodon grayi trnC                  GGTCCTGTAGTGAA-----TGATCATTTTGAATTGCAAATTCAAAGAAGCAGCTTCAA-TTCTGCCGGGACTT
Berardius bairdii trnC                   AGTCCTGTGGTGAA-----TAGTCATTTTGAATTGCAAATTCAAGGAAGCAGCTTCAA-TCCTGCCGGGACTT
Hyperoodon ampullatus trnC               AGTCCTGTAGTGAA-----TAGTCATTTTGAATTGCAAGTTCAAAGAAGCAGCTTCAA-TCCTGCCGGGACTT
Lipotes vexillifer trnC                  AGTCCTGTGGTGAA-----TAATCACTTTGAATTGCAAATTCAAGGGAGCAGCTTCAA-TCCTGCCGGGACTT
Inia geoffrensis trnC                    AGTCCTGTAGTGAG----TTAATCATTTTGAATTGCAAATTCAAAGAAGCAGCTTCAA-TTCTGCCGGGACTT
Pontoporia blainvillei trnC              GGTCCTGTGGTGAA----CTAGTCATTTTGAATTGCAAATTCAAGGAAGCAGCTTCAA-TCCTGCCGGGACTT
Monodon monoceros trnC                   AGTCCTGTGGTGAG-----TAGTCATTTTGAATTGCAAATTCAAAGAAGCAGCTTCAA-TCCTGCCGGGACTT
**Neophocaena asiaeorientalis trnC       GGTCCTGTAGTGAA-----TAATCATTTTGAATTGCAAATTCAAAGAAGCAGCTTCAA-TCCTGCCGGGACTT
Neophocaena phocaenoides trnC            GGTCCTGTAGTGAA-----TAATCATTTTGAATTGCAAATTCAAAGAAGCAGCTTCAA-TCCTGCCGGGACTT
Phocoena phocoena trnC                   GGTCCTGTAGTGAG-----TAATCATTTTGAATTGCAAATTCAAAGAAGCAGCTTCAA-TCCTGCCGGGACTT
Cephalorhynchus heavisidii trnC          AGTCCTGTAGTGAA-----TGATCATTTTGAATTGCAAATTCAAAGAAGCAGCTTCAA-TTCTGCCGGGACTT
Sousa chinensis trnC                     AGTCCTGTAGTGAA-----TAATCATTTTGAATTGCAAATTCAAAGAAGCAGCTTCAA-TCCTGCCTGGACTT
Stenella attenuata trnC                  GGTCCTGTAGTGAA-----TGATCATTTTGAATTGCAAATTCAAAGAAGCAGCTTCAA-TCCTGCCGGGACTT
Tursiops australis trnC                  GGTCCTGTAGTGAA-----TAATCATTTTGAATTGCAAATTCAAAGAAGCAGCTTCAA-TCCTGCCGGGACTT
Tursiops truncatus trnC                  AGTCCTGTAGTGAA-----TGATCATTTTGAATTGCAAATTCAAAGAAGCAGCTTCAA-TCCTGCCGGGACTT
Tursiops aduncus trnC                    AGTCCTGTAGTGAA-----TGATCATTTTGAATTGCAAATTCAAAGAAGCAGCTTCAA-TCCTGCCGGGACTT
Delphinus capensis trnC                  AGTCCTGTAGTGAA-----TGATCATTTTGAATTGCAAATTCAAAGAAGCAGCTTCAA-TCCTGCCGGGACTT
Stenella coeruleoalba trnC               AGTCCTGTAGTGAA-----TAATCATTTTGAATTGCAAATTCAAAGAAGCAGCTTCAA-TCCTGCCGGGACTT
Orcaella brevirostris trnC               AGTCCTGTAGTGAA-----TAATCATTTTGAATTGCAAATTCAAAGAAGCAGCTTCAA-TCCTGCCGGGACTT
Orcaella heinsohni trnC                  AGTCCTGTGGTGAA-----TGATCATTTTGAATTGCAAATTCAAAGAAGCAGCTTCAA-TCCTGCCGGGACTT
Grampus griseus trnC                     AGTCCTGTAGTGAA-----TAATCATTTTGAATTGCAAATTCAAAGAAGCAGCTTCAA-TCCTGCCGGGACTT
Pseudorca crassidens trnC                AGTCCTGTGGTGAA-----TGATCATTTTGAAGTGCAAATTCAAAGGAGCAGCTTCAA-TCCTGCCGGGACTT
Feresa attenuata trnC                    AGTCCTGTAGTGAA-----TAATCATTTTGAATTGCAAATTCAAAGGAGCAGCTTCAA-TCCTGCCGGGACTT
Peponocephala electra trnC               AGTTCTGTGGTGAA-----TAATCATTTTGAATTGCAAATTCAAAGGAGCAGTTTCAA-TCCTGCCGGGACTT
Globicephala macrorhynchus trnC          AGTCCTGTAGTGAA-----TAATCATTTTGAATTGCAAATTCAAAGGAGCAGCTTCAA-TCCTGCCGGGACTT
Globicephala melas trnC                  AGTCCTGTGGTGAA-----TAATCATTTTGAATTGCAAATTCAAAGGAGCAGCTTCAA-TCCTGCCGGGACTT
Lagenorhynchus albirostris trnC          AGTCTTGTAGTGAA-----TAATCATTTTGAATTGCAAATTCAAAGAAGCAGCTTCAA-TTCTGCCGGGACTT
Orcinus orca WNPTRU1 trnC                AGTCCTGTGGTGAA-----TAATCATTTTGAATTGCAAATTCAAAGAAGCAGCTTCAA-TCCTGCCTGGGCTT
Orcinus orca AntA1 trnC                  AGTCCTGTGGTGAA-----TAATCATTTTGAATTGCAAATTCAAAGAAGCAGCTTTAA-TCCTGCCTGGGCTT
Orcinus orca AntB1 trnC                  AGTCCTGTGGTGAA-----TAATCATTTTGAATTGCAAATTCAAAGAAGCAGCTTTAA-TCCTGCCTGGGCTT
Orcinus orca AntC1 trnC                  AGTCCTGTGGTGAA-----TAATCATTTTGAATTGCAAATTCAAAGAAGCAGCTTTAA-TCCTGCCTGGGCTT
Orcinus orca ENAHN1 trnC                 AGTCCTGTGGTGAA-----TAATCATTTTGAATTGCAAATTCAAAGAAGCAGCTTTAA-TCCTGCCTGGGCTT
Orcinus orca CNPNRAL trnC                AGTCCTGTGGTGAA-----TAATCATTTTGAATTGCAAATTCAAAGAAGCAGCTTTAA-TCCTGCCTGGGCTT
Orcinus orca ENPOAL2 trnC                AGTCCTGTGGTGAA-----TAATCATTTTGAATTGCAAATTCAAAGAAGCAGCTTTAA-TCCTGCCTGGGCTT
                                         1234567 1234      4321 12345 ant 54321    1234      43217654321d
                                         0000000 1111      2222 22333 333 44444    4555      66666666777 7
                                         1234567 0123      3456 89012 567 01234    9012      23456789012 3
```

N, the most common base for the position.

N, half compensatory base change in the stem pair (e.g. T – G vs C – G; A-T vs G-T).

N, N, half compensatory base change in the stem pair exhibiting a mismatch (e.g. T–A vs A–A). Different colours are used to better differentiate the changes.

N, fully compensatory base change in the stem pair exhibiting a mismatch (e.g. C–G vs T–T).

N, type I fully compensatory base change in the stem pair (i.e. purine – pyrimidine vs purine – pyrimidine, e.g. G – C vs A – T).

N, N, type II fully compensatory base change in the stem pair (i.e. purine – pyrimidine vs pyrimidine – purine, e.g. A – T vs T – A). Different colours are used to better differentiate the changes.

N, a mismatch in the in the stem pair; N, substitution pattern not modelled; *, pair in the stem in which a mismatch is prominent. N, N, molecular signature for a taxon.

X, position 1-7 in the acceptor stem; X position 1-4 in the DHU stem; X position 1-5 in the anticodon stem; X position 1-4 in the TΨC stem; ant, anticodon; d, discriminator nucleotide.

trnD (**ASP**) multiple alignment

```
                                          10        20        30        40        50        60
                                           |         |         |         |         |         |
                                  1234567  1234      4321 12345   ant   54321    12345      543217654321d
                                                                                   *          *
Ceratotherium simum trnD          GAGATATTAGTAAAA--ATTACATAACTTTGTCAAAGTTAAATTATAGGCTAAACCCCTATATATCTCT
Equus caballus trnD               GAGGTATTAGTAAAA--ATTACATGACTTTGTCAAAGTTAAATTATAGGTTAAACCCCTATATACCTCT
Pecari tajacu trnD                GAGATATTAGTAAAAACATTACATAACTTTGTCAAGGTTAAATTATAGTGAGAACCCTATATATCTCT
Phacochoerus africanus trnD       GAGATATTAGTAAAA-TATTACATAACTTTGTCAAAGTTATATTATAGGTGAAAGCCCTGTATATCTCC
Potamochoerus porcus trnD         GAGATACTAGTAAAA-TATTACATAACTTTGTCGAAGTTGTATTACAGGTGAAAGCCCTGTGTATCTCT
Sus scrofa trnD                   GAGGTATTAGTAAAA-TATTACATAACTTTGTCGAAGTTATATTATAGGTGAAAGCCCTATATGCCTCT
Camelus bactrianus trnD           GAGATATTAGTAAAA-T-TTACATAGCCTTGTCAAGGCTAAATTACAGGTGAAACCCCTGTATATCTCT
Camelus dromedarius trnD          GAGATATTAGTAAAA-T-TTACATAGCCTTGTCAAGGCTAAGTTACAGGTGAAAGCCCCGTATATCTCT
Lama guanicoe trnD                GAGGTATTAGTAAAA-C-TTACATGACCTTGTCAAGGTTAAATTATAGGTGAAAACCCTGTATACCTCT
Vicugna pacos trnD                GAGGTATTAGTAAAA-C-TTACATGACCTTGTCAAGGTCAAATTATAGGTGAAAACCCTGTATACCTCT
Hyemoschus aquaticus trnD         GAGGTATTAGTAAAA-TATTATATAACTTTGTCGAAGTTAAGTTACAGGCAAGAG-CCTGTATATCTCA
Tragulus kanchil trnD             GAGATATTAGTAAAA-TATTATATAACTTTGTCAAGGTTAAGTTGCAGACAAAAA-CCTGCATATCTCA
Giraffa camelopardalis trnD       GAGATACTAGTAAAA-TATTATATAACTTTGTCAAAGTTAAGTTACAGATGAAAATCCTGTGTACCTCA
Okapia johnstoni trnD             GAAGTGTTAGTAAAA-CATTACATGACTTTGTCAAAGTTAAGTTATAGGTGAGAACCCTATACACCTCA
Moschus moschiferus trnD          GAGATGTTAGTAAAA-CATTACATAACTTTGTCGAAGTTAAATTACAAGTGAAAATCCTGTACATCTCA
Muntiacus muntjak trnD            GAGGTTTTAGTAAAA-TATTATATAACTTTGTCAAAGTTAAGTTACAGGTGAAAACCCCGTATGCCTCA
Capreolus capreolus trnD          GAGGTATTAGTAAAAATATTATATGACTTTGTCAAGGTTAAGTTACAGGTGAAAGCCCCGTATACCTCA
Alces alces trnD                  GAGGCGTTAGTAAAA-TATTATATAACTTTGTCAAGGTTAAGTTACAGGTGAAAACCCCGTACACCTCA
Cervus elaphus trnD               GAGGTGTTAGTAAAA-TATTATATAACTTTGTCAAGGTTAAGTTACAGGTGAAAACCCCGTACATCTCA
Dama dama trnD                    GAGGTGTTAGTAAAA-CATTATATAACTTTGTCAGGGTTAAGTTACAGGTGAAAACCCCGTACACCTCA
Boselaphus tragocamelus trnD      GAGATGTTAGTAAAA-TATTATATAACTTTGTCGAAGTTAAGTTACAAGTGAAAACCCTGTACATCTCA
Tragelaphus oryx trnD             GAGATGTTAGTAAAA-TATTACATAACCTTGTCAAGGTTAAATTACAAGTGAAAATCCTGTACATCTCA
Bos taurus trnD                   GAGGTGTTAGTAAAA-CATTATATAATTTTGTCAAAGTTAAGTTACAAGTGAAAGTCCTGTACACCTCA
Bubalus bubalis trnD              GAGATGTTAGTAAAAACATTATATAACTTTGTCGAAGTTAAGTTACAAGTGAAAACCCTGTACATCTCA
Syncerus caffer trnD              GAGATGTTAGTAAAA-CATTATATAACTTTGTCGAAGTTAAGTTACAAGTGAAAATCCTGTACATCTCA
Neotragus moschatus trnD          GAGATGTTAGTAAAT-TATTATATAACTTTGTCAAGGTTAAGTCACGAGTGAAAACCCCGTACATCTCA
Alcelaphus buselaphus trnD        GAGACGTTAGTAAAA-TATTACATAACTTTGTCAAGGTTAAATTACAGGTGAAAACCCCGTACATCTCA
Oryx gazella trnD                 GAGGTGTTAGTAAAA-CATTACATAACCTTGTCAAGATTAAATTACAGGTGAAAATCCCGTACATCTCA
Pantholops hodgsonii trnD         GAGAAGTTAGTAAAA-CATTACATAATCTTGTCAAGATTAAATTACAGGTGAAAATCCCGTACATCTCA
Ovibos moschatus trnD             GAGATGTTAGTAAAA-CATTACATAATCTTGTCAAGATTAAATTACAGGTGAAAATCCCGTACATCTCA
Capra hircus trnD                 GAGATGTTAGTAAAA-TATTACATAATCTTGTCAAGATTAAATTACAGGTGAAAATCCCGTACATCTCA
Ovis aries trnD                   GAGATGTTAGTAAAA-CATTACATAACCTTGTCAAGATTAAATTACAGGTGAAAATCCCGTACATCTCA
Cephalophus natalensis trnD       GAGACGTTAGTAAAA-CATTACGTAATCTTGTCAAGATTAAATTACAGGTGGAAATCCCGTACATCTCA
Redunca fulvorufula trnD          GAGATGTTAGTAAAA-CATTACATAATCTTGTCAAGATTAAATTACAGGTGAAAATCCCGTACATCTCG
Antilocapra americana trnD        GAGATATTAGTAAAA-TATTACATGACCTTGTCAAGGTTAAATTATAGGTGAAAGCCCTATATACCTCG
Ourebia ourebi trnD               AAGGTGTTAGTAAAA-CATTACATAACCTTGTCAAGATTAAATTACAGGTGAAAGCCCTGTACACCTTA
Antilope cervicapra trnD          AAGATGTTAGTAAAA-TATTACATAACCTTGTCAAGGTTAAATTACAGGTGAAAACCCCGTACATCTTA
Gazella gazella trnD              AAGATGTTAGTAAAA-TATTACATAACCTTGTCAAGGTTAAATTACAGGTGAAAACCCCGTACATCTTG
Procapra gutturosa trnD           AAGATGTTAGTAAAA-CATTACATAACTTTGTCAAGGTTAAATTACAGGTGAAAATCCCGTACATCTTA
Raphicerus campestris trnD        AAGATGTTAGTAAAA-CATTACATAACCTTGTCAAGGCTAAATTACAGGTGAAAGCCCCGTACATCTTA
Hexaprotodon liberiensis trnD     GAGGTATTAGTAAAA-T-TTACATGACTTCGTCAAAGTTAAGTTATAGGTGAAAATCCTGTATATCTCC
Hippopotamus amphibius trnD       GAGGTATTAGTAAAA--ATTACATAACTTCGTCAAAGTTAAGTTACAGGTGAAAACCCTGTCTACCTCC
Eubalaena australis trnD          GAGATATTAGTAAAA-TCTTATATAACTTTGTCAAAGTTAAGTTACAAGTGAAAATCCTGTATATCTCT
Eubalaena japonica trnD           GAGATATTAGTAAAA-TCTTATATAACTTTGTCAAAGTTAAGTTACAAGTGAAAACCCTGTATATCTCT
Balaena mysticetus trnD           GAGATATTAGTAAAA-TCTTATATAACTTTGTCAAAGTTAAGTTACAAGTGAAAATCCTGTATATCTCT
Caperea marginata trnD            GAGATATTAGTAAAA-TCTTATATAACTTTGTCAAAGTTAAGTTACAAGTGAAAATCTTGTATATCTCC
Eschrichtius robustus trnD        GAGATATTAGTAAAA-CCTTATATAACTTTGTCAAAGTTAAGTTACAAGTGAGAACCCTGTATATCTCC
Balaenoptera acutorostrata trnD   GAGATATTAGTAAAA-CCTTACATAACTTTGTCAAAGTTAAGTTACAAGTGAGAATCCTGTATATCTCC
Balaenoptera bonaerensis trnD     GAGATATTAGTAAAA-TCTTATATAACTTTGTCAAAGTTAAGTTACAAGTGAGAATCCTGTATATCTCC
Balaenoptera physalus trnD        GAGATATTAGTAAAA-CCTTATATAACTTTGTCAAAGTTAAGTTACAAGTGAAAATCCTGTATATCTCC
Megaptera novaeangliae trnD       GAGATATTAGTAAAA-CCTTATATAACTTTGTCAAAGTTAAGTTACAAGTGAAAATCCTGTATATCTCC
Balaenoptera musculus trnD        GAGATATTAGTAAAA-CCTTATATAACTTTGTCAAAGTTAAGTTACAAGTGAAAATCCTGTATATCTCC
Balaenoptera omurai trnD          GAGATATTAGTAAAA-CCTTACATAACTTTGTCAAAGTTAAGTTACAAGTGAAAGCCCTGTATATCTCC
Balaenoptera borealis trnD        GAGATATTAGTAAAA-CCTTATATAACTTTGTCAAAGTTAAGTTACAAGTGAAAATCCTGTATATCTCC
Balaenoptera brydei trnD          GAGATATTAGTAAAA-TCTTACATAACTTTGTCAAAGTTAAGTTACAAGTGAAAATCCTGTATATCTCC
Balaenoptera edeni trnD           GAGATATTAGTAAAA-TCTTACATAACTTTGTCAAAGTTAAGTTACAAGTGAAAATCCTGTATATCTCC
Kogia breviceps trnD              GAGATATTAGTAAAA-TCTTACATAACTTTGTCAAAGTTAAATTACAAGTGAAAACCCTGTATATCTCC
Physeter macrocephalus trnD       GAGATATTAGTAAAA-CCTTACATAACTTTGTCAAAGTTAAATTACAAGTGAAAACCCTGTATATCTCC
Platanista minor trnD             GAGATATTAGTAAAG-TCTTATATAACTTTGTCAAAGTTAAGTCACAAGTGAAAATCCTGTATATCTCT
Ziphius cavirostris trnD          GAGATATTAGTAAAA-TTTTACATAACTTCGTCAAAGTTAAGTCACAAGTGAAAATCCTGTATATCTCC
Mesoplodon densirostris trnD      GAGGTATTAGTAAAG-TCTTACATAACTTTGTCAAAGTTAAATCACAAGTGAAAATCCTGTATATCTCT
Mesoplodon europaeus trnD         GAGATATTAGTAAAG-TCTTACATAACTTTGTCAAAGTTAAGTCACAAGTGAAAATTCGTATATCTCC
**Mesoplodon grayi trnD           GAGGTATTAGTAAAG-TTTTACATAACTTTGTCAAAGTTAAATCACAAGTGAAAATCCTGTATATCTCT
Berardius bairdii trnD            GAGGTATTAGTAAAA-TTTTACATAACTTTGTCAAAGTTAAGTCACAAGTGAAAACCCTGTATATCTCC
Hyperoodon ampullatus trnD        GAGGTATTAGTAAAA-TCTTACATAACTTTGTCAAAGTTAAGTCACAAGTGAAAATCCTGTATATCTCT
Lipotes vexillifer trnD           GAGATATTAGTAAAA-CTTTACATAACTTTGTCAAAGTTAAGTCACAAGTGAAAATCCTGTATATCTCA
Inia geoffrensis trnD             AAGATATTAGTAAAA-CCTTACATAACTTTGTCAAAGTTAAATCACAAGTGAAAACCTTGTATATCTTT
Pontoporia blainvillei trnD       GAGATATTAGTAAAG--CTTACATAACTTTGTCAAAGTTAAGACACAAGTGAAAACCTTGTATATCTCT
Monodon monoceros trnD            GAGATATTAGTAAAG-CCTTACATAACTTTGTCAGAGTTAAGTCACAAGTGAAAATCCTGTATATCTCT
**Neophocaena_asiaeorientalis trnD GAGATATTAGTAAAG-CCTTACATAACTTTGTCAAAGTTAAGTCACAAGTGAAAATTCTGTATATCTCC
Neophocaena phocaenoides trnD     GAGGTATTAGTAAAA-TTTTACATAACTTTGTCAAAGTTAAATTACAAGTGAAAGCTTGTATATCTCC
Phocoena phocoena trnD            GAGATATTAGTAAAA-TCTTACATAACTTTGTCAAAGTTAAGTCACAAGTGGAATCTTGTATATCTCC
Cephalorhynchus heavisidii trnD   GAGATATTAGTAAAA-TCTTACGTAACTTTGTCAAAGTTAAATCACAAGTGAAAATCCTGTATATCTCC
Sousa chinensis trnD              GAGATATTAGTAAAG-TCTTACGTAACTTTGTCAAAGTTAAATCACAAGTGAAAATCCTGTATATCTCC
Stenella attenuata trnD           GAGATATTAGTAAAG-TCTTACGTAACTTTGTCAAAGTTAAATCACAAGTGAAAATCCTGTATATCTCC
Tursiops australis trnD           GAGATATTAGTAAAG-TCTTACGTAACTTTGTCAAAGTTAAATCACAAGTGAAAATCCTGTATATCTCC
Tursiops truncatus trnD           GAGATATTAGTAAAG-TCTTACGTAACTTTGTCAAAGTTAAATCACAAGTGAAAATCCTGTATATCTCT
Tursiops aduncus trnD             GAGATATTAGTAAAG-TCTTACGTAACTTTGTCAAAGTTAAATCACAAGTGAAAATCCTGTATATCTCC
Delphinus capensis trnD           GAGATATTAGTAAAG-TCTTACGTAACTTTGTCAAAGTTAAATCACAAGTGAAAATCCTGTATATCTCC
Stenella coeruleoalba trnD        GAGATATTAGTAAAG-TCTTACGTAACTTTGTCAAAGTTAAATCACAAGTGAAAATCCTGTATATCTCC
Orcaella brevirostris trnD        GAGGTATTAGTAAAG-TCTTACGTAACTTTGTTAAAGTTAAATCACAAGTGAAAATCCTGTATATCTCC
Orcaella heinsohni trnD           GAGATATTAGTAAAG-TCTTACGTAACTTTGTCAAAGTTAAATCACAAGTGAAAACCCTGTATATCTCC
Grampus griseus trnD              GAGATATTAGTAAAA-CCTTACGTAACTTTGTCAAAGTTAAATCACAAGTGAAAATCCTGTATATCTCC
Pseudorca crassidens trnD         GAGATATTAGTAAAG-TCTTACGTAACTTCGTCAAAGTTAAATCACAAGTGAAAATCCTGTATATCTCT
Feresa attenuata trnD             GAGGTATTAGTAAAG-TCTTACGTAACTTTGTCAAAGTTAAATCACAAGTGAAAATCCTGTATATCTCC
Peponocephala electra trnD        GAGATATTAGTAAAG-TCTTACGTAACTTTGTCAAAGTTAAATCACAAGTGAAAACCCTGTATATCTCC
Globicephala macrorhynchus trnD   GAGATATTAGTAAAG-CCTTACGTAACTTTGTCAAAGTTAAATCACAAGTGAAAATCCTGTATATCTCC
Globicephala melas trnD           GAGATATTAGTAAAG-TCTTACGTAACTTTGTCAAAGTTAAATCACAAGTGAAAATCCTGTATATCTCC
Lagenorhynchus albirostris trnD   GAGATATTAGTAAAG-TCTTACATAACTTTGTCAAAGTTAAATCACAAGTGAAAATCCTGTATATCTCC
Orcinus orca WNPTRU1 trnD         GAGGTATTAGTAAAG-TCTTACGTAACTTTGTCAAAGTTAAATCACAAGTGAAAATCCTGTATATCTCC
Orcinus orca AntA1 trnD           GAGGTATTAGTAAAG-TCTTACGTAACTTTGTCAAAGTTAAATCACAAGTGAAAATCCTGTATATCTCC
Orcinus orca AntB1 trnD           GAGGTATTAGTAAAG-TCTTACGTAACTTTGTCAAAGTTAAATCACAAGTGAAAATCCTGTATATCTCC
Orcinus orca AntC1 trnD           GAGGTATTAGTAAAG-TCTTACGTAACTTTGTCAAAGTTAAATCACAAGTGAAAATCCTGTATATCTCC
Orcinus orca ENAHN1 trnD          GAGGTATTAGTAAAG-TCTTACGTAACTTTGTCAAAGTTAAATCACAAGTGAAAATCCTGTATATCTCC
Orcinus orca CNPNRAL trnD         GAGGTATTAGTAAAG-TCTTACGTAACTTTGTCAAAGTTAAATCACAAGTGAAAATCCTGTATATCTCC
Orcinus orca ENPOAL2 trnD         GAGGTATTAGTAAAG-TCTTACGTAACTTTGTCAAAGTTAAATCACAAGTGAAAATCCTGTATATCTCC
                                                                                   *          *
                                  1234567  1234      4321 12345   ant   54321    12345      543217654321d
                                  0000000  1111      1222 22222   333   33334    44444      555666666666
                                  1234567  0123      9012 45678   123   67890    56789      789012345678 9
```

N, the most common base for the position.

N, half compensatory base change in the stem pair (e.g. T – G vs C – G; A-T vs G-T).

N, N, half compensatory base change in the stem pair exhibiting a mismatch (e.g. T–A vs A–A). Different colours are used to better differentiate the changes.

N, fully compensatory base change in the stem pair exhibiting a mismatch (e.g. C–G vs T–T).

N, type I fully compensatory base change in the stem pair (i.e. purine – pyrimidine vs purine – pyrimidine, e.g. G – C vs A – T).

N, N, type II fully compensatory base change in the stem pair (i.e. purine – pyrimidine vs pyrimidine – purine, e.g. A – T vs T – A). Different colours are used to better differentiate the changes.

N, a mismatch in the in the stem pair; N, substitution pattern not modelled; *, pair in the stem in which a mismatch is prominent. N, N, molecular signature for a taxon.

X, position 1-7 in the acceptor stem; X position 1-4 in the DHU stem; X position 1-5 in the anticodon stem; X position 1-5 in the TΨC stem; ant, anticodon; d, discriminator nucleotide.

trnE (GLU) multiple alignment

```
                                                10        20        30        40        50        60
                                                 |         |         |         |         |         |
                                        1234567  1234     4321 12345  ant  54321    12345       543217654321d
Ceratotherium simum trnE                GTTCTTGTAGTTGAAGTACAACGAAGGTTTTTCATGCCGTTGGTCATGGTTGGAGTCCATGTAAGAATA
Equus caballus trnE                     GTTCTTATAGTTGAAATACAACGATGATTTTTCATGTCATTGGTCGTGGTTAGATTCCACGTGGGAATA
Pecari tajacu trnE                      GTTCTTATAGTTGAATAACAACGATGGTTTTTCATATCATTGGTCATGGTTGGATTCCATGTGAGAATA
Phacochoerus africanus trnE             GTTCTTGTAGTTGAAGTACAACGATGATTTTTCATGTCATTGGTCATGGTTAGATTCCATGTGAGAATA
Potamochoerus porcus trnE               GTTCTTGTAGTTGAAGTACAACGATGATTTTTCATGTCATTGGTCATGGTTAGATTCCATGTGAGAATA
Sus scrofa trnE                         GTTCTTGTAGTTGAAGTACAACGATGATTTTTCATGTCATTGGTCGTGGTTAAATTCCATGTGAGAATA
Camelus bactrianus trnE                 GTTCTTATAGTTGAACTACAACGATGATTTTTCATGTCATTGGTCATGGTTTGAGTCCATGTAGGAATA
Camelus dromedarius trnE                GTTCTTATAGTTGAACTACAACGATGATTTTTCATGTCATTGGTCATGGTTTGAGTCCATGTAGGAATG
Lama guanicoe trnE                      GTTCTTATAGTTGAATTACAACGATGATTTTTCATGTCATTAGTCGTGGTTAGAGTCCATGTAGGAATG
Vicugna pacos trnE                      GTTCTTATAGTTGAATTACAACGATGATTTTTCATGTCATTAGTCGTGGTTAGAGTCCATGTAGGAATA
Hyemoschus aquaticus trnE               GTTCTTGTAGTTGAACAACAACGATGGTTTTTCATATCATTGGTCATGGT TAATTCCATGTAAGAATG
Tragulus kanchil trnE                   GTTTTCGTAGTTGAATGACAACGATGGTTTTTCATATCATTAGTCGTGGTTAGATTCCATGCGAGAATA
Giraffa camelopardalis trnE             GTTCTTATAGTTGAATGACAACGATGGTTTTTCATATCATTAGTCATGGTTAGATTCCATGTAAGAATA
Okapia johnstoni trnE                   GTTCTTGTAGTTGAATGACAACGATGATTTTTCATATCATTAGTCGTGGTTAGATCCCATGCAGGAATA
Antilocapra americana trnE              GTTCTTATAGTTGAATGACAACGATGGTTTTTCATATCATTGGTCATGGTTAGATTCCATGTGAGAATA
Moschus moschiferus trnE                GTTCTTGTAGTTGAATGACAACGATGGTTTTTCATATCATTAGTCGTGGTTAGATTCCATGCAAGAATA
Muntiacus muntjak trnE                  GTTCTTGTAGTTGAATGACAACGATGGTTTTTCATATCATTAGTCGTGGTTAGATTCCATGCGAGAATA
Capreolus capreolus trnE                GTTCTTGTAGTTGAATGACAACGATGGTTTTTCATATCATTAGTCGTGGTTTTACTCCATGCGAGAATA
Alces alces trnE                        GTTCTTGTAGTTGAATGACAACGATGATTTTTCATATCATTAGTCGTGGTTAGATTCCATGCGAGAATA
Cervus elaphus trnE                     GTTCTTGTAGTTGAATGACAACGATGGTTTTTCATATCATTAGTCATGGTTAGATTCCATGTGAGAATA
Dama dama trnE                          GTTCTTGTAGTTGAATGACAACGATGGTTTTTCATATCATTAGTCATGGTTAGATTCCATGTGAGAATA
Boselaphus tragocamelus trnE            GTTCTTGTAGTTGAATGACAACGATGGTTTTTCATATCATTAGTCATGGTTAAATTCCATGCAAGAATA
Tragelaphus oryx trnE                   GTTCTTATAGTTGAATGACAACGATGGTTTTTCATATCATTAGTCATGGTTTAATTCCATGTGAGAATA
Bos taurus trnE                         GTTCTTGTAGTTGAATGACAACGATGGTTTTTCATATCATTAGTCATGGTTAGATTCCATGTAAGAATA
Bubalus bubalis trnE                    GTTCTTGTAGTTGAATGACAACGATGGTTTTTCATATCATTGGTCGTGGTTGAATTCCACGTGAGAATA
Syncerus caffer trnE                    GTTCTTGTAGTTGAATGACAACGATGGTTTTTCATATCATTAGTCGTGGTTAAATTCCATGCGAGAATA
Neotragus moschatus trnE                GTTCTTGTAGTTGAATGACAACGATGGTTTTTCATATCATTAGTCGTGGTTAAATTCCACGTGAGAATA
Alcelaphus buselaphus trnE              GTTCTTGTAGTTGAATAACAACGATGGTTTTTCATATCATTAGTCGTGGTTAAATTCCATGCGAGAATA
Oryx gazella trnE                       GTTCTCGTAGTTGAATGACAACGATGGTTTTTCATATCATTAGTCGTGGTTAGATTCCATGTGAGAATA
Pantholops hodgsonii trnE               GTTCTCGTAGTTGAATGACAACGATGGTTTTTCATATCATTGGTCATGGTTAGATTCCATGCGAGAGTG
Ovibos moschatus trnE                   GTTCTTGTAGTTGAATAACAACGATGGTTTTTCATATCATTGGTCATGGTTAGAGTCCATGTGAGAATA
Capra hircus trnE                       GTTCTTGTAGTTGAATAACAACGATGGTTTTTCATATCATTGGTCATGGTTAGATTCCATGTGAGAATA
Ovis aries trnE                         GTTCTTATAGTTGAATGACAACGATGGTTTTTCATATCATTGGTCATGGTTAGATTCCATGTGAGAATG
Cephalophus natalensis trnE             GTTCTTGTAGTTGAATGACAACGATGGTTTTTCATATCATTAGTCGTGGTTAGATTCCATGCAAGAATA
Redunca fulvorufula trnE                GTTCTTGTAGTTGAATAACAACGATGGTTTTTCATATCATTAGTCGTGGTTAGATTCCATGTGAGAATA
Ourebia ourebi trnE                     GTTCTTATAGTTGAATGACAACGATGGTTTTTCATATCATTAGTCATGGTTAGATTCCATGTGAGAATA
Antilope cervicapra trnE                GTTCTTGTAGTTGAATGACAACGATGGTTTTTCATATCATTAGTCATGGTTAGATTCCATGCGAGAATA
Gazella gazella trnE                    GTTCTTGTAGTTGAATGACAACGATGGTTTTTCATATCATTAGTCGTGGTTAGATTCCATGCGAGAATA
Procapra gutturosa trnE                 GTTCTTGTAGTTGAATGACAACGATGGTTTTTCATATCATTAGTCGTGGTTAGATTCCATGCGAGAATA
Raphicerus campestris trnE              GTTCTTGTAGTTGAATGACAACAATGGTTTTTCATATCATTAGTCGTGGTTAGATTCCATGCGAGAATA
Hexaprotodon liberiensis trnE           GTTCTTGTAGTTGAATTACAACGATGGTTTTTCATGCCGTTGATTATGGTTAGAGTCCATATGAGAATA
Hippopotamus amphibius trnE             GTTCTTATAGTTGAATTACAACGATGGTTTTTCATGCCGTTGGTTATGGTTAGAGTCCATATGGGAATA
Eubalaena australis trnE                GTTCTTGTAGTTGAATAACAACGATGATTTTTCATGTCATTAGTCATGGTTGGAGTCCATGTAAGAATA
Eubalaena japonica trnE                 GTTCTTGTAGTTGAATAACAACGATGATTTTTCATGTCATTAGTCATGGTTAGAGTCCATGTAAGAATA
Balaena mysticetus trnE                 GTTCTTGTAGTTGAATAACAACGATGATTTTTCATGTCATCAGTCATGGTTGAAGTCCATGTGAGAATA
Caperea marginata trnE                  GTTCTTATAGTTGAATAACAACGGTGATTTTTCATGTCATTGGTCATGGTTGAAGTCCATGTGGGAATA
Eschrichtius robustus trnE              GTTCTTATAGTTGAATGACAACGATGATTTTTCATGTCATTGGTCATGGTTGAAGTCCATGTGAGAATG
Balaenoptera acutorostrata trnE         GTTCTTGTAGTTGAATGACAACGATGATTTTTCATGTCATAAGTCATGGTTGA ATCCATGTGAGAATA
Balaenoptera bonaerensis trnE           GTTCTTGTAGTTGAATGACAACGATGATTTTTCATGTCAT GGTCATGGTTGA ATCCATGTGAGAATA
Balaenoptera physalus trnE              GTTCTTGTAGTTGAATAACAACGATGATTTTTCATGTCATTGGTCATGGTTGAAGTCCATGTGAGAATA
Megaptera novaeangliae trnE             GTTCTTGTAGTTGAATAACAACGATGATTTTTCATGTCATTGGTCATGGTTGAAGTCCATGTGAGAATG
Balaenoptera musculus trnE              GTTCTTGTAGTTGAATAACAACGATGATTTTTCATGTCATTGGTCATGGTTGAAGTCCATGTGAGAATA
Balaenoptera omurai trnE                GTTCTTGTAGTTGAATGACAACGATGATTTTTCATGTCATTGGTCATGGTTGAAGTCCATGTGAGAATA
Balaenoptera borealis trnE              GTTCTTGTAGTTGAATGACAACGATGATTTTTCATGTCATTGGTCATGGTTGAAGTCCATGTGAGAATA
Balaenoptera brydei trnE                GTTCTTGTAGTTGAATGACAACGATGATTTTTCATGTCATTGGTCATGGTTGAAGTCCATGTGAGAATA
Balaenoptera edeni trnE                 GTTCTTGTAGTTGAATGACAACGATGATTTTTCATGTCATTGGTCGTGGTTGAAGTCCATGTGAGAATA
Kogia breviceps trnE                    GTTCTTGTAGTTGAATAACAACGATGATTTTTCATGTCATTGGTCATGGTTTGAGTCCATGTGAGAATG
Physeter macrocephalus trnE             GTTCTTGTAGTTGAATAACAACGGTGACTTTTCATGTCATTGGTCGTGGTTTGAGTCCATGTAGGAATG
Platanista minor trnE                   GTTCTTGTAGTTGAATAACAACGATGGTTTTTCATGTCATTGGTCATGGTTAGAATCCATGTGAGAATA
Ziphius cavirostris trnE                GTTCTTGTAGTTGAATAACAACGATGATTTTTCATGTCATTGGTCATGGTTAAAGTCCATGTGGGAATA
Mesoplodon densirostris trnE            GTTCTTATAGTTGAAT-ACAACGATGATTTTTCATGTCATTAGTCATGGTTAGAGTCCATGTAAGAATG
Mesoplodon europaeus trnE               GTTCTTATAGTTGAAT-ACAACGATGATTTTTCATGTCATTGGTCATGGTTAGAGTCCATGTAAGAATA
**Mesoplodon grayi trnE                 GTTCTTATAGTTGAAT-ACAACGATGATTTTTCATGTCATTGGTCATGGTTAGAATCCATGTAGGAATA
Berardius bairdii trnE                  GTTCTTATAGTTGAATAACAACGATGATTTTTCGTGTCATTGGTCATGGTTGGAGTCCATGTAAGGATA
Hyperoodon ampullatus trnE              GTTCTTGTAGTTGAATAACAACGATGATTTTTCATGTCATTGGTCATGGTTAGAGTCCATGTGAGAATA
Lipotes vexillifer trnE                 GTTCTTATAGTTGAATAACAACGATGGTTTTTCATATCATAGGTCATGGTTGAAGTCCATGTGAGAATA
Inia geoffrensis trnE                   GTTCTTATAGTTGAATAACAACGATGATTTTTCATGTCATTGGTCCTGGTTAAAGCCTAGGTGAGAATG
Pontoporia blainvillei trnE             GTTCTTATAGTTGAATAACAACGATGATTTTTCATGTCATTGGTCATGGTTGGAGTCCATGTGAGAATA
Monodon monoceros trnE                  GTTCTTATAGTTGAATTACAACGATGATTTTTCATGTCATTGGTCGTGGTTAGAACCATGTGAGAATA
**Neophocaena asiaeorientalis trnE      GTTCTCATAGTTGAATTACAACGATGATTTTTCATGTCATTGGTCATGGTTGGAGTCCATGTGAGAGTA
Neophocaena phocaenoides trnE           GTTCTCATAGTTGAATTACAACGATGATTTTTCATGTCATTGGTCATGGTTGGAGTCCATGTGAGAGTA
Phocoena phocoena trnE                  GTTCTTATAGTTGAATAACAACGATGATTTTTCATGTCATTGGTCATGGTTGGAGTCCATGTGAGAATA
Cephalorhynchus heavisidii trnE         GTTCTTGTAGTTGAATTACAACGATGATTTTTCATGTCATTGGTCATGGTTGGAGTCCATGTGAGAATA
Sousa chinensis trnE                    GTTCTTGTAGTTGAATTATAACGATGATTTTTCATGTCATTGGTCATGGTTAGAGTCCATGTGAGAATA
Stenella attenuata trnE                 GTTCTTGTAGTTGAATTACAACGATGATTTTTCATGTCATTGGTCATGGTTGGAGTCCATGTGAGAATA
Tursiops australis trnE                 GTTCTTGTAGTTGAATTACAACGATGATTTTTCATGTCATTGGTCATGGTTGGAGTCCATGTGAGAATA
Tursiops truncatus trnE                 GTTCTTGTAGTTGAATTACAACGATGATTTTTCATGTCATTGGTCATGGTTGAAGTCCATGTGAGAATA
Tursiops aduncus trnE                   GTTCTTGTAGTTGAATTACAACGATGATTTTTCATGTCATTGGTCATGGTTGGAGTCCATGTGAGAATA
Delphinus capensis trnE                 GTTCTTGTAGTTGAATTACAACGATGATTTTTCATGTCATTGGTCATGGTTGGAGTCCATGTGAGAATA
Stenella coeruleoalba trnE              GTTCTTGTAGTTGAATTACAACGATGATTTTTCATGTCATTGGTCATGGTTGGAGTCCATGTGAGAATA
Orcaella brevirostris trnE              GTTCTTGTAGTTGAATTACAACGATGATTTTTCATGTCATTGGTCGTGGTTAGAGTCCATGTGAGAATA
Orcaella heinsohni trnE                 GTTCTTGTAGTTGAATTACAACGATGATTTTTCATGTCATTGGTCATGGTTGAAGTCCATGTGAGAATA
Grampus griseus trnE                    GTTCTTGTAGTTGAATTACAACGATGATTTTTCATGTCATTGGTCATGGTTGGAGTCCATGTGAGAATA
Pseudorca crassidens trnE               GTTCTTGTAGTTGAATTACAACGATGATTTTTCATGTCATTGGTCGTGGTTGAAGTCCATGTGGGAATA
Feresa attenuata trnE                   GTTCTTGTAGTTGAATTACAACGATGGTTTTTCATATCATTGGTCATGGTTGGAGTCCATGTGAGAATA
Peponocephala electra trnE              GTTCTTGTAGTTGAATTACAACGATGGTTTTTCATATCATTGGTCATGGTTAGAGTCCATGTGAGAATA
Globicephala macrorhynchus trnE         GTTCTTATAGTTGAATTACAACGATGGTTTTTCATATCATTGGTCATGGTTGGAGTCCATGTGAGAATA
Globicephala melas trnE                 GTTCTTATAGTTGAATTACAACGATGGTTTTTCATATCATTGGTCATGGTTGGAGTCCATGTGAGAATA
Lagenorhynchus albirostris trnE         GTTCTTGTAGTTGAATTACAACGATGATTTTTCATGTCATTGGTCATGGTTGAAGACCATGTGAGAATA
Orcinus orca WNPTRU1 trnE               GTTCTTGTAGTTGAATTACAACGATGATTTTTCATGTCATTGGTCATGGTTGGAGTCCATGTGAGAACA
Orcinus orca AntA1 trnE                 GTTCTTGTAGTTGAATTACAACGATGATTTTTCATGTCATTGGTCATGGTTGGAGTCCATGTGAGAACA
Orcinus orca AntB1 trnE                 GTTCTTGTAGTTGAATTACAACGATGATTTTTCATGTCATTGGTCATGGTTGGAGTCCATGTGAGAACA
Orcinus orca AntC1 trnE                 GTTCTTGTAGTTGAATTACAACGATGATTTTTCATGTCATTGGTCATGGTTGGAGTCCATGTGAGAACA
Orcinus orca ENAHN1 trnE                GTTCTTGTAGTTGAATTACAACGATGATTTTTCATGTCATTGGTCATGGTCGGAGTCCATGTGAGAACA
Orcinus orca CNPNRAL trnE               GTTCTTGTAGTTGAATTACAACGATGATTTTTCATGTCATTGGTCATGGTTGGAGTCCATGTGAGAACA
Orcinus orca ENPOAL2 trnE               GTTCTTGTAGTTGAATTACAACGATGATTTTTCATGTCATTGGTCATGGTTGGAGTCCATGTGAGAACA
                                        1234567  1234     4321 12345  ant  54321    12345       543217654321d
                                        0000000  1111     1222 22222  333  33334    44444       5556666666666
                                        1234567  0123     9012 45678  123  67890    56789       7890123456789
```

N, the most common base for the position.

N, half compensatory base change in the stem pair (e.g. T – G vs C – G; A-T vs G-T).

N, N, half compensatory base change in the stem pair exhibiting a mismatch (e.g. T–A vs A–A). Different colours are used to better differentiate the changes.

N, fully compensatory base change in the stem pair exhibiting a mismatch (e.g. C–G vs T–T).

N, type I fully compensatory base change in the stem pair (i.e. purine – pyrimidine vs purine – pyrimidine, e.g. G – C vs A – T).

N, N, type II fully compensatory base change in the stem pair (i.e. purine – pyrimidine vs pyrimidine – purine, e.g. A – T vs T – A). Different colours are used to better differentiate the changes.

N, a mismatch in the in the stem pair; N, substitution pattern not modelled; *, pair in the stem in which a mismatch is prominent. N, N, molecular signature for a taxon.

X, position 1-7 in the acceptor stem; X position 1-4 in the DHU stem; X position 1-5 in the anticodon stem; X position 1-5 in the TΨC stem; **ant**, anticodon; **d**, discriminator nucleotide.

trnF (PHE) multiple alignment

```
                                   10        20        30        40        50        60        70
                                    |         |         |         |         |         |         |
                             1234567  1234          4321 12345  ant  54321    1234        43217654321d
                               *                                                                *
Ceratotherium simum trnF     GTTAATGTAGCTTAACAA----CTAAAGCAGGGCATTGAAAATGCCCAGATGAGCCCAC--CAGCTCCATAAACA
Equus caballus trnF          GTTAATGTAGCTTAATAA---TATAAAGCAAGGCACTGAAAATGCCTAGATGAGTATTC--TTACTCCATAAACA
Pecari tajacu trnF           GTTAATGTAGCTTAAACA---AATAAAGCAAAGCACTGAAAATGCTTAGATGAGTAAG--CCACTCCATAAACA
Phacochoerus africanus trnF  GTTAATGTAGCTTAAACT---ATCAAAGCAAGGCACTGAAAATGCCTAGATGAGCCTT--GTAGCTCCATAAACA
Potamochoerus porcus trnF    GTTAATGTAGCTTAAATT---ACCAAAGCAAGGCACTGAAAATGCCTAGATGAGCCTCA--TAGCTCCATAAACA
Sus scrofa trnF              GTTAATGTAGCTTAAATT---ATCAAAGCAAGGCACTGAAAATGCCTAGATGAGCCTCA--CAGCTCCATAAACA
Camelus bactrianus trnF      GTTAATGTAGCTTAACT-----TCAAAGCAAGGCGCTGAAAATGCCTGGATGGGCACC---TGGCCCCATGAACA
Camelus dromedarius trnF     GTTGATGTAGCTTAACT-----TCAAAGCAAGGCGCTGAAAATGCCTGGATGGGCACC---TAGCCCCATAAACA
Lama guanicoe trnF           GTTAATGTAGCTTAACT-----TAAAAGCAAGGCGCTGAAAATGCCTAGATGGGCTCA---CGGCCCCATAAACA
Vicugna pacos trnF           GTTAATGTAGCTTAACT------TAAAGCAAGGCGCTGAAAATGCCTAGATGGGCTCA---CGGCCCCATAAACA
Hyemoschus aquaticus trnF    GTTAATGTAGCTTAATC----ACCAAAGCAAGGCACTGAAAATGCCTAGATGAGCATCCC-ATGCTCCATAAACA
Tragulus kanchil  trnF       GTTAATGTAGCTTAATAA----TAAAAGCAAGGCACTGAAAATGCCTAGATGAGTAACA-CATACTCCATAAACA
Giraffa camelopardalis trnF  GTTGATGTAGCTTAAWTA----CCAAAGCGAGGCACTGAAAATGCCTATATGGGTGAGT--TAACCCCATGAACA
Okapia johnstoni trnF        GTTAATGTAGCTTAAACA----CCAAAGCAAAGCACTGAAAATGCTTAGATGAGTATGT--TAACTCCATAAACA
Antilocapra americana trnF   GTTGATGTAGCTTAAAT------TAAAGCAAGGCGCTGAAAATGCCTAGATGAGTGCA--CTAACTCCATAAACA
Moschus moschiferus trnF     GTTAATGTAGCTTAATAT-----TAAAGCAAGGCACTGAAAATGCCTAGATGAGTATA--TAAACTCCATAAACA
Muntiacus muntjak trnF       GTTAATGTAGCTTAAACA----ATAAAGCAAGGCACTGAAAATGCCTAGATGAGTGTA--TTAACTCCATAAACA
Capreolus capreolus trnF     GTTAATGTAGCTTAAACA----ATAAAGCAAGGCACTGAAAATGCCTAGATGAGTGTA--CTAACTCCATAAACA
Alces alces trnF             GTTAATGTAGCTTAAATA----ACAAAGCAAGGCACTGAAAATGCCTAGATGAGTATAT--TAACTCCATAAACA
Cervus elaphus trnF          GTTGATGTAGCTTAAATG----GCAAAGCAAGGCACTGAAAATGCCTAGATGAGTATAT--TAACTCCATAAACA
Dama dama trnF               GTTAATGTAGCTTAAACA----GTAAAGCAAGGCACTGAAAATGCCTAGATGAGTGTA--TTAACTCCATAAACA
Boselaphus tragocamelus trnF GTTAATGTAGCTTAAAGT----CCAAAGCAAGGCACTGAAAATGCCTAGATGAGTACAC--CAACTCCATAAACA
Tragelaphus oryx trnF        GTTAATGTAGCTTAAAA-----CTAAAGCAAGGCGCTGAAAATGCCTAGATGAGTCCAC--CAACTCCATAAACA
Bos taurus trnF              GTTGATGTAGCTTAAC------CCAAAGCAAGGCACTGAAAATGCCTAGATGAGTCTCC--CAACTCCATAAACA
Bubalus bubalis trnF         GTTAATGTAGCTTAAAA-----CCAAAGCAAGGCACTGAAAATGCCTAGATGAGTTCTCC-CAACTCCATAAACA
Syncerus caffer trnF         GTTAATGTAGCTTAAAA-----CCAAAGCAAGGCACTGAAAATGCCTAGATGAGTTTTCCCCAACTCCATAAACA
Neotragus moschatus trnF     GTTAATGTAGCTTAAAA-----CTAAAGCAAGGCACTGAAAATGCCTAGATGAGTACC--CGAACTCCATAAACA
Alcelaphus buselaphus trnF   GTTAATGTAGCTTAAAA-----CCAAAGCAAGGCACTGAAAATGCCTAGATGAGTGTA--CGGACTCCATGAACA
Oryx gazella trnF            GTTCATGTAGCTTAAAA-----CCAAAGCAAGGCACTGAAAATGCCTAGATGAGTATAT--TAACTCCATAAACA
Pantholops hodgsonii  trnF   GTTAATGTAGCTTAACCC----TTAAAGCAAGGCACTGAAAATGCCTAGATGAGTATG--TTTACTCCATAAACA
Ovibos moschatus trnF        GTTAATGTAGCTTAAAA-----TTAAAGCAAGGCACTGAAAATGCCTAGATGAGTACA--CTAACTCCATAAACA
Capra hircus trnF            GTTGATGTAGCTTAAAC-----TTAAAGCAAGGCACTGAAAATGCCTAGATGAGTGTA--CCAACTCCATAAACA
Ovis aries trnF              GTTAATGTAGCTTAAAC-----TTAAAGCAAGGCACTGAAAATGCCTAGATGAGTCTA--CTGACTCCATGAACA
Cephalophus natalensis trnF  GTTGATGTAGCTTAAAA-----CTAAAGCAAGGCACTGAAAATGCCTAGATGAGTACT--CCAACTCCATAAACA
Redunca fulvorufula trnF     GTTAATGTAGCTTAAAC-----TTAAAGCAAGGCACTGAAAATGCCTAGATGAGTATC--CTAACTCCATAAACA
Ourebia ourebi trnF          GTTAATGTAGCTTAAAC-----CAAAAGCAAGGCACTGAAAATGCCTAGATGAGTATGT--CAACTCCATAAACA
Antilope cervicapra trnF     GTTAATGTAGCTTAAAT-----CCAAAGCAAGGCACTGAAAATGCCTAGATGAGTACAC--CAACTCCATAAACA
Gazella gazella trnF         GTTAATGTAGCTTAAAT-----CCAAAGCAAGGCACTGAAAATGCCTAGATGAGTATAT--TAACTCCATAAACA
Procapra gutturosa trnF      GTTAATGTAGCTTAAAC-----CCAAAGCAAGGCACTGAAAATGCCTAGATGAGTATAC--TAACTCCATAAACA
Raphicerus campestris trnF   GTTAATGTAGCTTAAGA-----CCAAAGCAAGGCACTGAAAATGCCTAGATGAGTATAT--TAACTCCATAAACA
Hexaprotodon liberiensis trnF GTTAACGTAGCTCAAACA---CCCAAAGCGAGGCACTGAAAATGCCTAGATGGGCTCGCC-TAGCCCCGTAAACA
Hippopotamus amphibius trnF  GTTAACGTAGCTCAAACA---CCCAAAGCGAGGCACTGAAAATGCCTAGATGGGCTCACC-CAGCCCCGTAAACA
Eubalaena australis trnF     GTTAATGTAGCTTAAATATTT-GTAAAGCAAGACACTGAAAATGTCTAGATGGACTTAAC-TAACCCCATTGACA
Eubalaena japonica trnF      GTTAATGTAGCTTAAATATTT-GTAAAGCAAGACACTGAAAATGTCTAGATGGACTTAAC-TAACCCCATTGACA
Balaena mysticetus trnF      GTTAATGTAGCTTAAATATTT-ATAAAGCAAAACACTGAAAATGTTTAGATGGGTTAAT-TAACCCCATTGACA
Caperea marginata trnF       GTTAATGTAGCTTAAACATCT-ATAAAGCAAGACACTGAAAATGTCTAGATGGGCCAAC-CAGCCCCATTAGCA
Eschrichtius robustus trnF   GTTAATGTAGCTTAAACATTCATTAAAGCAAGACACTGAAAATGTCTAGATGGGTCCAAC-CGACCCCATTGACA
Balaenoptera acutorostrata trnF GTTAATGTAGCTTAAACACTTACTAAAGCAAGACACTGAAAATGTCTAGATGGGCTAGC-CAACCCCATTAACA
Balaenoptera bonaerensis trnF GTTAATGTAGCTTAAATACCT-ACAAAGCAAGACACTGAAAATGTCTAGATGGGTCTAGC-CAACCCCATTAACA
Balaenoptera physalus trnF   GTTGATGTAGCTTAAACACTT-ACAAAGCAAGACACTGAAAATGTCTAGATGGGTCTAGC-CAACCCCATTGACA
Megaptera novaeangliae trnF  GTTAATGTAGCTTAAACACTC-ACAAAGCAAGACACTGAAAATGTCTAGATGGGTCTAT-CAACCCCATTGACA
Balaenoptera musculus trnF   GTTAATGTAGCTTAAGCACTC-ATAAAGCAAGACACTGAAAATGTCTAGATGGGTTAAT-TAACCCCATTGGCA
Balaenoptera omurai trnF     GTTAATGTAGCTTAAAT-TTT-ACAAAGCAAGACACTGAAAATGTCTAGATGGGTTCAC-CAACCCCATTGACA
Balaenoptera borealis trnF   GTTAATGTAGCTTAAACAATT-ACAAAGCAAGACACTGAAAATGTCTAGATGGGTTCAC-CAACCCCATTGACA
Balaenoptera brydei trnF     GTTAATGTAGCTTAAACAATT-ACAAAGCAAGACACTGAAAATGTCTAGATGGGTTCAC-CAGCCCCATTGACA
Balaenoptera edeni trnF      GTTAATGTAGCTTAAACAATT-ACAAAGCAAGACACTGAAAATGTCTAGATGGGTTCAC-CAACCCCATTGACA
Kogia breviceps trnF         GTTAACGTAGCTTAAAATTTTCACAAAGCAAGACACTGAAAATGTCTAGATGGACTTG-C-CAGTCCCGCTAACA
Physeter macrocephalus trnF  GTTAGTGTAGTTtAAGTTTTTAATAAAGCAAGACACTGAAGATGTCTAGACGGACCCG-C-TAGTCCCACCAACA
Platanista minor trnF        GCTAACGTAGCTTAAACATCTCAAAAAGCAAGACACTGAAAATGTCTAGATGGACTTA-C-TAGTCCCGTTAACA
Ziphius cavirostris trnF     GTTAATGTAGCTTAAATTTTCACTAAAGCAAGACACTGAAAATGTCTAGATGGGCATATA-CTGCCCCATCAACA
Mesoplodon densirostris trnF GTTAATGTAGCTTAAATTTTTACTAAAGCAAGACACTGAAAATGTCTAGATGGGCATATA-CCGCCCCATCAACA
Mesoplodon europaeus trnF    GTTAATGTAGCTTACATTTTTACTAAAGCAAGACACTGAAAATGTCTAGACGGGCATACA-CCGCCCCATCAACA
**Mesoplodon grayi trnF      GTTAATGTAGCTTAAATTTT-ACTAAAGCAAGGCACTGAAAATGTCTAGATGGGCATACA-TCGCCCCATCAACA
Berardius bairdii trnF       GTTAATGTAGCTTAAACTTCCACTAAAGCAAGACACTGAAAATGTCTAGATGGCA-TA-C-CTGCCCCATCAACA
Hyperoodon ampullatus trnF   GTTAATGTAGCTTAAATTTTTACTAAAGCAAGACACTGAAAATGTCTAGATGGGCATGTA-CTGCCCCATCAACA
Lipotes vexillifer trnF      GTCAATGTAGCTTAAACTTTT-A-AAAGCAAGACACTGAAAATGTCTAAATGGGCTAAAC-TAGCCCCATTAACA
Inia geoffrensis trnF        GTAGATGTAGCTTAAA-CACC-ACAAAGCAAGACACTGAAGATGTCTAGACGGACTCA--CAGTCCCATCAACA
Pontoporia blainvillei trnF  GTAGATGTAGCTTAAATCAC--ATAAAGCAAGACACTGAAAATGTCTAGATGGACTCA--CAGCCCCATCAACA
Monodon monoceros trnF       GTTAATGTAGCTTAAGTTTTC-AAAAAGCAAGACACTGAAAATGTCTAGATGGGCCTTA--CCGCCCCATCAACA
**Neophocaena asiaeorientalis trnF GTTAATGTAGCTTAAATTTTC-A-AAAGCAAGACACTGAAAATGTCTAGATGGGCCT-A--TCGCCCCATCAACA
Neophocaena phocaenoides trnF GTTAATGTAGCTTAAATTTTC-A-AAAGCAAGACACTGAAAATGTCTAGATGGGCCT-A--TCGCCCCATCAACA
Phocoena phocoena trnF       GTTAATGTAGCTTAAACTTTC-AAAAAGCAAGACACTGAAAATGTCTAGATGGCT-T-A--TCGCCCCATCAACA
Cephalorhynchus heavisidii trnF GTTAATGTAGCTTAAAATTTT-ATAAAGCAAGACACTGAAAATGTCTAGATGGGCATTA--CTGCCCCATCAACA
Sousa chinensis trnF         GTTAATGTAGCTTAAATTTTT-ATAAAGCAAGACACTGAAAATGTCTAGATGGACATTA--TTGCCCCATCAACA
Stenella attenuata trnF      GTTAATGTAGCTTAAATTTTT-ATAAAGCAAGACACTGAAAATGTCTAGATGGGCATTA--TTGCCCCATCAACA
Tursiops australis trnF      GTTAATGTAGCTTAAATTTTT-ATAAAGCAAGACACTGAAAATGTCTAGATGGGCATTA--TTGCCCCGTCAACA
Tursiops truncatus trnF      GTTAATGTAGCTTAAATTTTT-ATAAAGCAAGACACTGAAAATGTCTAGATGGGCATTA--TTGCCCCATCAACA
Tursiops aduncus trnF        GTTAATGTAGCTTAAATTTTT-ACAAAGCAAGACACTGAAAATGTCTAGATGGGCATTA--TTGCCCCATCAACA
Delphinus capensis trnF      GTTAATGTAGCTTAAATTTTT-ACAAAGCAAGACACTGAAAATGTCTAGATGGGCATTA--TTGCCCCATCAACA
Stenella coeruleoalba trnF   GTTAATGTAGCTTAAATTTTT-ACAAAGCAAGACACTGAAAATGTCTAGATGGGCATTA--TTGCCCCATCAACA
Orcaella brevirostris trnF   GTTAATGTAGCTTAAAATTTT-ATAAAGCAAGACACTGAAAATGTCTAGATGGATATTA--CTGTCCCATCAACA
Orcaella heinsohni trnF      GTTAATGTAGCTTAAATTCTT-ACAAAGCAAGACACTGAAAATGTCTAGATGGGCATTA--TTGCCCCATCAACA
Grampus griseus trnF         GTTAATGTAGCTTAAACTTTT-ATAAAGCAAGACACTGAAAATGTCTAGATGGGCATTA--TTGCCCCGTCAACA
Pseudorca crassidens trnF    GTTAATGTAGCTTAAACTTTT-ATAAAGCAAGACACTGAAAATGTCTAGATGGGCATTA--TTGCCCCGTCAACA
Feresa attenuata trnF        GTTAATGTAGCTTAAATTTTT-ATAAAGCAAGACACTGAAAATGTCTAGATGAGCATTA--CTGCCCCATCAACA
Peponocephala electra trnF   GTTAATGTAGCTTAAATTTTT-ATAAAGCAAGACACTGAAAATGTCTAGACGGCATTA--TTGCCCCATCAACA
Globicephala macrorhynchus trnF GTTAATGTAGCTTAAATTTTT-ATAAAGCAAGACACTGAAAATGTCTAGATGGACATTA--TTGCCCCATCAACA
Globicephala melas trnF      GTTAATGTAGCTTAAATTTTT-ATAAAGCAAGACACTGAAAATGTCTAGATGGGCATTA--TTGCCCCATCAACA
Lagenorhynchus albirostris trnF GTTAATGTAGCTTAAATTTTC-ATAAAGCAAGACACTGAAAATGTCTAGATGGGCATTA--TTGCCCCATCAACA
Orcinus orca WNPTRU1 trnF     GTTGATGTAGCTTAAATTTTT-ATAAAGCAAGACACTGAAAATGTCTAGATGGACATTA--TTGCCCCGTTAACA
Orcinus orca AntA1 trnF       GTTGATGTAGCTTAAATTTTT-ATAAAGCAAGACACTGAAAATGTCTAGATGGACATTA--TTGCCCCATTAACA
Orcinus orca AntB1 trnF       GTTGATGTAGCTTAAATTTT--ATAAAGCAAGACACTGAAAATGTCTAGATGGACATTA--TTGCCCCATTAACA
Orcinus orca AntC1 trnF       GTTGATGTAGCTTAAATTTT--ATAAAGCAAGACACTGAAAATGTCTAGATGGACACTA--TTGCCCCATTAACA
Orcinus orca ENAHN1 trnF      GTTGATGTAGCTTAAATTTTT-ATAAAGCAAGACACTGAAAATGTCTAGATGGACATTA--TTGCCCCATTAACA
Orcinus orca CNPNRAL trnF     GTTGATGTAGCTTAAATTTTT-ATAAAGCAAGACACTGAAAATGTCTAGATGGACATTA--TTGCCCCATTAACA
Orcinus orca ENPOAL2 trnF     GTTGATGTAGCTTAAATTTTT-ATAAAGCAAGACACTGAAAATGTCTAGATGGACATTA--TTGCCCCATTAACA
                               *                                                                *
                             1234567  1234          4321 12345  ant  54321    1234        43217654321d
                             0000000  1111          2222 33333  334  44444    5555        66666677777 7
                             1234567  0123          6789 12345  890  34567    2345        45678901234 5
```

N, the most common base for the position.

N, half compensatory base change in the stem pair (e.g. T – G vs C – G; A-T vs G-T).

N, N, half compensatory base change in the stem pair exhibiting a mismatch (e.g. T–A vs A–A). Different colours are used to better differentiate the changes.

N, fully compensatory base change in the stem pair exhibiting a mismatch (e.g. C–G vs T–T).

N, type I fully compensatory base change in the stem pair (i.e. purine – pyrimidine vs purine – pyrimidine, e.g. G – C vs A – T).

N, N, type II fully compensatory base change in the stem pair (i.e. purine – pyrimidine vs pyrimidine – purine, e.g. A – T vs T – A). Different colours are used to better differentiate the changes.

N, a mismatch in the in the stem pair; N, substitution pattern not modelled; *, pair in the stem in which a mismatch is prominent. N, N, molecular signature for a taxon.

X, position 1-7 in the acceptor stem; X position 1-4 in the DHU stem; X position 1-5 in the anticodon stem; X position 1-4 in the TΨC stem; **ant**, anticodon; **d**, discriminator nucleotide.

trnG (GLY) multiple alignment

```
                                             10        20        30        40        50        60        70
                                              |         |         |         |         |         |         |
                                        1234567  1234      4321 12345  ant  54321   12345     543217654321d
                                          *                                                          *
Ceratotherium simum trnG                ATTCTTTTAGTATTAAA-CAGTACAATTGACTTCCAATCAATCAGCTTCGGTAA-AACCCGAAAAAGAATA
Equus caballus trnG                     ATTCTTTTAGTATTGAC-CAGTACAATTGACTTCCAATCAATCAGCTTCGGTAT-AACCCGAAAAAGAATA
Pecari tajacu trnG                      ATTCTTTTAGTATCAAA-AAGTACAACTGACTTCCAATCAGTTAGCTTCGGTAA-AGCCCGAAAAAGAATA
Phacochoerus africanus trnG             ACTCTTTTAGTATCAAG-CAGTACAATTGACTTCCAATCAATTAGTTTCGGTAG-ACTCCGAAAAAGAGTA
Potamochoerus porcus trnG               ACTCTTTTAGTATCAAA-TAGTACAATTGACTTCCAATCAATCAGCTTCGGTAA-ACCCCGAAAAAGAGTA
Sus scrofa trnG                         ACTCTTTTAGTATTAAG-TAGTACAATTGACTTCCAATCAATCAGTTTCGGTAA-ACTCCGAAAAAGAGTA
Camelus bactrianus trnG                 GTCCTTTTAGTATCAAT-TAGTACAACTGACTTCCAATCAGTTAGCTTCGGATAGCCCCCGAAAAAGGATA
Camelus dromedarius trnG                GTCCTTTTAGTATTAAT-TAGTACAACTGACTTCCAATCAGTTAGCTTCGGATGATCCCCGAAAAAGGATA
Lama guanicoe trnG                      GTCCTTTTAGTATTAAT-TAGTACAACTGACTTCCAATCAGTTAGATTCGGA-GAGACCCGAAAAAGGATA
Vicugna pacos trnG                      GTCCTTTTAGTATTAAT-TAGTACAACTGACTTCCAATCAGTTAGATTCGGA-GAGACCCGAAAAAGGATA
Hyemoschus aquaticus trnG               ATTCTTTTAGTATTAAC-CAGTACAACTGACTTCCAATCAATTAGCTTCGGT-C-AACCCGGAAAAGAATA
Tragulus kanchil trnG                   ATTCTTTTAGTATTAAA-CAGTACAATTGACTTCCAATCAATTAGCTTCGGTAG-AACCCGAAAAAGAATA
Giraffa camelopardalis trnG             GTTCTTTTAGTATCAAC-CAGTACAACTGACTTCCAATCAGTTAGTTTCGGT-CTAGTCCGAAAAAGAATA
Okapia johnstoni trnG                   ATTCTTTTAGTATCGAC-CAGTACAACTGACTTCCAATCAGTTAGCTTCGGTA-TAACCCGAAAAAGAATA
Antilocapra americana trnG              ATTCTTTTAGTATTAAC-CAGTACAGCTGACTTCCAATCAGCCAGTTCCGGTCA-AACCCGGAAGAGAATA
Moschus moschiferus trnG                ATTCTTTTAGTATCAAC-TAGTACAACTGACTTCCAATCAGTTAGTTTCGGTTA-AATCCGAAAAAGAATA
Muntiacus muntjak trnG                  ATTCTTTTAGTATTAAC-CAGTACAACTGACTTCCAATCAGTTAGTTTCGGTA-TAGCCCGAAAAAGAATA
Alces alces trnG                        ATTCTTTTAGTATTAAT-CAGTACAACTGACTTCCAATCAGTTAGTTTCGGTAATAATCCGAAAAAGAATA
Capreolus capreolus trnG                ATCCTTTACGTATGACT-TAGCACCACTGACTTCCAATCAGTTAGCTTCGGTAGTA-TCCGAAAAAGAATA
Cervus elaphus trnG                     ATTCTTTTAGTATTAAC-TAGTACAGCTGACTTCCAATCAGCTAGTTTCGGTA-TAACCCGAAAAAGAATA
Dama dama trnG                          ATTCTTTTAGTATTAAT-CAGTACAACTGACTTCCAATCAGTTAGTTTCGGTAA-ACCCCGAGAAAGAATA
Boselaphus tragocamelus trnG            ATTCTTTTAGTATCAAT-CAGTACAACTGACTTCCAATCAGTTAGTTTCGGTC-TAACCCGAAAAAGAATA
Tragelaphus oryx trnG                   ATTCTTTTAGTATTAAT-CAGTACAACTGACTTCCAATCAGTTAGCCTTGGTC-TAACCCAAGAAAGAATA
Bos taurus trnG                         ATTCTTTTAGTATTAAC-TAGTACAGCTGACTTCCAATCAGCTAGTTTCGGTC-TAGTCCGAAAAAGAATA
Bubalus bubalis trnG                    ATTCTTTTAGTATCAAG-CAGTACAGCTGACTTCCAATCAGCTAGTTTCGGTCT-AACCCGAAAAAGAATA
Syncerus caffer trnG                    ATTCTTTTAGTATCAAA-CAGTACAGCTGACTTCCAATCAGCTAGTTTCGGTCC-AACCCGAAAAGGAATA
Neotragus moschatus trnG                ATTCTTTTAGTATTAAT-CAGTACAACTGACTTCCAATCAGTTAGTTTCGGTCA-AATCCGAAAAAGAATA
Alcelaphus buselaphus trnG              GCTCTTTTAGTATTAAC-TAGTACAACTGACTTCCAATCAGTTAGTTTCGGT-CAAACCCGGAAAGAACA
Oryx gazella trnG                       GTTCTTTTAGTATTAAT-TAGTACAACTGACTTCCAATCAGTTAGTTTCGGT-CTATCCGAAAAAGAATA
Pantholops hodgsonii trnG               GTCCTTTTAGTATTAAT-AAGTACAACTGACTTCCAATCAGTTAGTTTCGGT-CTAACCCGAAAAAGAACA
Ovibos moschatus trnG                   GTTCTTTTAGTATTAAT-CAGTACAACTGACTTCCAATCAGTTAGTTTCGGT-TAAACCCGAAAAAGATCA
Capra hircus trnG                       GTCCTTTTAGTATTAAT-CAGTACAACTGACTTCCAATCAGTTAGTTTCGGTACAA-TCCGAAAAAGAACA
Ovis aries trnG                         GTCCTTTTAGTATTAAT-TAGTACAACTGACTTCCAATCAGTTAGTTTCGGT-CTATCCGAAAAAGAACA
Cephalophus natalensis trnG             ATTCTTTTAGTATCAAT-CAGTACAACTGACTTCCAATCAGTTAGTTTCGGTCTAA-CCCGAAAAAGAATA
Redunca fulvorufula trnG                ATTCTTTTAGTATTAAC-TAGTACAACTGACTTCCAATCAGTTAGTTTCGGTTCAA-CCCGAAAAAGAATA
Ourebia ourebi trnG                     ATTCTTTTAGTATTAAT-CAGTACAACTGACTTCCAATCAGTTAGTTTCGGTCCAA-TCCGAAAAAGAATA
Antilope cervicapra trnG                ATTCTTTTAGTATTAAT-AAGTACAACTGACTTCCAATCAGTTAGTTTCGGTCTAA-CCCGAAAAAGAATA
Gazella gazella trnG                    ATTCTTTTAGTATTAAC-AAGTACAACTGACTTCCAATCAGTTAGTTTCGGTCCAA-CCCGAAAAAGAATA
Procapra gutturosa trnG                 ATTCTTTTAGTATTAAT-CAGTACAACTGACTTCCAATCAGTTAGTTTCGGTCAAA-CCCGAAAAAGAATA
Raphicerus campestris trnG              ATTCTTTTAGTATTAAT-TAGTACAACTGACTTCCAATCAGTTAGCTTCGGTCAAA-CCCGAAAAAGAATA
Hexaprotodon liberiensis trnG           GTTCTTTTAGTATTAAAACAGTACAGCTGACTTCCAATCAGCTAGCCTCAGT-GCACTCTGGAGAGAACA
Hippopotamus amphibius trnG             GTTCTTTTAGTATTAAAACAGTACAGCTGACTTCCAATCAGCTAGCCTCAGT-GCACTCTGGAAGGAACA
Eubalaena australis trnG                GCTCTTTTAGTATTAAT-AAGTACAACTGACTTCCAATCAGTTAGTTTCGGTA-TACTCCGAAAAAGAACA
Eubalaena japonica trnG                 GCTCTTTTAGTATTAAT-AAGTACAACTGACTTCCAATCAGTTAGTTTCGGTA-TACTCCGAAAAAGAACA
Balaena mysticetus trnG                 GCTCTTTTAGTATCAAT-AAGTACAACTGACTTCCAATCAGTTAGTTTCGGTA-TACCCCGAAAAAGAACA
Caperea marginata trnG                  GTCCTTTTAGTATTAAT-CAGTACAGCTGACTTCCAATCAGTTAGTTTCGGTC-CATTCCGAAAAAGAACA
Eschrichtius robustus trnG              GTCCTTTTAGTATCAAT-GAGTACAACTGACTTCCAATCAGTTAGTTTCGGTA-TATCCCGAAAAAGAACA
Balaenoptera acutorostrata trnG         GTCCTTTTAGTATTAAC-AAGTACAACTGACTTCCAATCAGTTAGTTTCGGTA-CACCCCGAAAAAGAACA
Balaenoptera bonaerensis trnG           GTCCTTTTAGTATTAAT-TAGTACAACTGACTTCCAATCAGTTAGTTTCGGTA-CATCCCGAAAAAGAACA
Balaenoptera physalus trnG              GTCCTTTTAGTATTAAC-AAGTACAACTGACTTCCAATCAGTTAGTTTCGGTG-TACCCCGAAAAAGAACA
Megaptera novaeangliae trnG             GTTCTTTTAGTATCAAC-AAGTACAACTGACTTCCAATCAGTTAGTTTCGGTA-TACCCCGAAAAAGAACA
Balaenoptera musculus trnG              GTTCTTTTAGTATTAAC-AAGTACAACTGACTTCCAATCAGTTAGTTTCGGTA-TACCCCGAAAAAGAACA
Balaenoptera omurai trnG                GTTCTTTTAGTATTAAC-AAGTACAACTGACTTCCAATCAGTTAGTTTCGGTA-TACCCCGAAAAAGAGCA
Balaenoptera borealis trnG              GTTCTTTTAGTATTAAT-AAGTACAACTGACTTCCAATCAGTTAGTTTCGGTA-TACCCCGAAAAAGAACA
Balaenoptera brydei trnG                GTTCTTTTAGTATTAAC-AAGTACAATTGACTTCCAATCAGTTAGTTTCGGTA-TACCCCGAAAAAGAACA
Balaenoptera edeni trnG                 GTTCTTTTAGTAT-AAC-AAGTACAACTGACTTCCAATCAGTTAGTTTCGGTA-TACCCCGAAAAAGAACA
Kogia breviceps trnG                    GTCCTTTTAGTATTAAT-CAGTACAACTGACTTCCAATCAGTTAGCTTCGGTA-CACCCCGAAAAAGAACA
Physeter macrocephalus trnG             GTCCTTTTAGTATTAAC-AAGTACAACTGACTTCCAATCAGTTAGTTTCGGTA-AACTCCGAAAAAGAACA
Platanista minor trnG                   GTTCTTTTAGTATTAAT-AAGTACAACTGACTTCCAATCAGTTAGTTTTGGTC-TACCCCGAAAAAGAACA
Ziphius cavirostris trnG                GTCCTTTTAGTATTAAT-AAGTACAACTGACTTCCAATCAGTTAGTTTCGGTG-CACTCCGAAAAAGAACA
Mesoplodon densirostris trnG            GTCCTTTTAGTATTAAC-AAGTACAACTGACTTCCAATCAGTTAGTTTCGGTA-TACCCCGAAAAAGAACA
Mesoplodon europaeus trnG               GTCCTTTTAGTATTAAT-AAGTACAACTGACTTCCAATCAGTTAGTTTCGGTA-TACCCCGAAAAAGAACA
**Mesoplodon grayi trnG                 GTCCTTTTAGTATTAAT-AAGTACAACTGACTTCCAATCAGTTAGTTTCGGTA-TACCCCGAAAAAGAACA
Berardius bairdii trnG                  GTCCTTTTAGTATCAAT-AAGTACAGCTGACTTCCAATCAGCTAGTTTCGGTA-TACCCCGAAAAAGAACA
Hyperoodon ampullatus trnG              GTCCTTTTAGTATTAAC-AAGTACAGCTGACTTCCAATCAGTTAGTTTCGGTA-TACCCCGAAAAAGAACA
Lipotes vexillifer trnG                 ATTCTTTTAGTATTAAT-AAGTACAACTGACTTCCAATCAGTTAGTTTCGGAACACTCCGAAAAAGAATA
Inia geoffrensis trnG                   GTCCTTTTAGTATCAAC-AAGTACAACTGACTTCCAATCAGTTAGTTTCGGTA-AAATCCGAAAAAGAACA
Pontoporia blainvillei trnG             GTTCTCTTAGTATCAAT-AAGTACAACTGACTTCCAATCAGTTAGTTTCGGTC-CACCCCGAAAAAGAACA
Monodon monoceros trnG                  GTCCTTTTAGTATTAAC-AAGTACAACTGACTTCCAATCAGTTAGTTTCGGTA-GACCCCGAAAAAGAACA
**Neophocaena_asiaeorientalis trnG      GTTCTTTTAGTATTAAT-TAGTACAACTGACTTCCAATCAGTTGGTTTCGGTC-TACCCCGAAAAAGAACA
Neophocaena phocaenoides trnG           GTTCTTTTAGTATTAAT-TAGTACAACTGACTTCCAATCAGTTGGTTTCGGTC-TACCCCGAAAAAGAACA
Phocoena phocoena trnG                  GTCCTTTTAGTATTAAG-TAGTACAACTGACTTCCAATCAGTTAGTTTCGGTC-TACCCCGAAAAAGAACA
Cephalorhynchus heavisidii trnG         GTCCTTTTAGTATTAAC-TAGTACAACTGACTTCCAATCAGTTAGTTTCGGTA-CACTCCGAAAAAGAACA
Sousa chinensis trnG                    GTCCTTTTAGTATTAAT-AAGTACAACTGACTTCCAATCAGTTAGTTTCGGTA-CACCCCGAAAAAGAACA
Stenella attenuata trnG                 GTCCTTTTAGTATTAAT-AAGTACAACTGACTTCCAATCAGTTAGTTTCGGTA-CACTCCGAAAAAGAACA
Tursiops australis trnG                 GTCCTTTTAGTATTAAT-AAGTACAACTGACTTCCAATCAGTTAGTTTCGGTA-CACTCCGAAAAAGAACA
Tursiops truncatus trnG                 GTCCTTTTAGTATTAAT-AAGTACAACTGACTTCCAATCAGTTAGTTTCGGTA-CACCCCGAAAAAGAACA
Tursiops aduncus trnG                   GTCCTTTTAGTATTAAT-AAGTACAACTGACTTCCAATCAGTTAGTTTCGGTA-CACCCCGAAAAAGAACA
Delphinus capensis trnG                 GTCCTTTTAGTATTAAT-AAGTACAACTGACTTCCAATCAGTTAGTTTCGGTA-CACCCCGAAAAAGAACA
Stenella coeruleoalba trnG              GTCCTTTTAGTATTAAT-AAGTACAACTGACTTCCAATCAGTTAGTTTCGGTA-CACCCCGAAAAAGAACA
Orcaella brevirostris trnG              GTCCTTTTAGTATTAAT-AAGTACAACTGACTTCCAATCAGTTAGTTTCGGTA-TACTCCGAAAAAGAACA
Orcaella heinsohni trnG                 GTCCTTTTAGTATTAAT-AAGTACAACTGACTTCCAATCAGTTAGTTTCGGTA-TACTCCGAAAAAGAACA
Grampus griseus trnG                    GTCCTTTTAGTATTAAC-AAGTACAACTGACTTCCAATCAGTTAGTTTCGGTA-CACTCCGAAAAAGAACA
Pseudorca crassidens trnG               GTCCTTTTAGTATTAAT-AAGTACAACTGACTTCCAATCAGTTAGTTTCGGTA-CACTCCGAAAAAGAACA
Feresa attenuata trnG                   GTCCTTTTAGTATTAAC-AAGTACAACTGACTTCCAATCAGTTAGTTTCGGTA-TACCCCGAAAAAGAACA
Peponocephala electra trnG              GTCCTTTTAGTATTAAT-AAGTACAACTGACTTCCAATCAGTTAGTTTCGGTA-CACTCCGAAAAAGAACA
Globicephala macrorhynchus trnG         GTCCTTTTAGTATTAAC-AAGTACAACTGACTTCCAATCAGTTAGTTTCGGTA-CACTCCGAAAAAGAACA
Globicephala melas trnG                 GTCCTTTTAGTATTAAC-AAGTACAACTGACTTCCAATCAGTTAGTTTCGGTA-CACTCCGAAAAAGAACA
Lagenorhynchus albirostris trnG         GTCCTTTTAGTATTAAC-AAGTACAGCTGACTTCCAATCAGTTAGTTTCGGTA-CACTCCGAAAAAGAACA
Orcinus orca WNPTRU1 trnG               GTCCTTTTAGTATTAAT-AAGTACAACTGACTTCCAATCAGTTAGTTTCGGTA-TACCCCGAAAAAGAACA
Orcinus orca AntA1 trnG                 GTCCTTTTAGTATTAAT-AAGTACAACTGACTTCCAATCAGTTAGTTTCGGTA-TACCCCGAAAAAGAACA
Orcinus orca AntB1 trnG                 GTCCTTTTAGTATTAAT-AAGTACAACTGACTTCCAATCAGTTAGTTTCGGTA-TACCCCGAAAAAGAACA
Orcinus orca AntC1 trnG                 GTCCTTTTAGTATTAAT-AAGTACAACTGACTTCCAATCAGTTAGTTTCGGTA-TACCCCGAAAAAGAACA
Orcinus orca ENAHN1 trnG                GTCCTTTTAGTATTAAT-AAGTACAACTGACTTCCAATCAGTTAGTTTCGGTA-TACCCCGAAAAAGAACA
Orcinus orca CNPNRAL trnG               GTCCTTTTAGTATTAAT-AAGTACAACTGACTTCCAATCAGTTAGTTTCGGTA-TACCCCGAAAAAGAACA
Orcinus orca ENPOAL2 trnG               GTCCTTTTAGTATTAAT-AAGTACAACTGACTTCCAATCAGTTAGTTTCGGTA-TACCCCGAAAAAGAACA
                                          *                                                          *
                                        1234567  1234      4321 12345  ant  54321   12345     543217654321d
                                        0000000  1111      2222 22223  333  33444   44455     566666666667
                                        1234567  0123      1234 67890  345  89012   78901     901234567890 1
```

N, the most common base for the position.

N, half compensatory base change in the stem pair (e.g. T – G vs C – G; A-T vs G-T).

N, N, half compensatory base change in the stem pair exhibiting a mismatch (e.g. T–A vs A–A). Different colours are used to better differentiate the changes.

N, fully compensatory base change in the stem pair exhibiting a mismatch (e.g. C–G vs T–T).

N, type I fully compensatory base change in the stem pair (i.e. purine – pyrimidine vs purine – pyrimidine, e.g. G – C vs A – T).

N, N, type II fully compensatory base change in the stem pair (i.e. purine – pyrimidine vs pyrimidine – purine, e.g. A – T vs T – A). Different colours are used to better differentiate the changes.

N, a mismatch in the in the stem pair; N, substitution pattern not modelled; *, pair in the stem in which a mismatch is prominent. N, N, molecular signature for a taxon.

X, position 1-7 in the acceptor stem; X position 1-4 in the DHU stem; X position 1-5 in the anticodon stem; X position 1-5 in the TΨC stem; ant, anticodon; d, discriminator nucleotide.

trnH (HIS) multiple alignment

```
                                              10        20        30        40        50        60        70
                                               |         |         |         |         |         |         |
                                      1234567  1234      4321 12345  ant  54321    12345        543217654321d
Ceratotherium simum trnH              GCAGATATAGTTTAA-CAAAAACATTAGATTGTGAATCTAATAATAGAAGCT-TAACACTTCTTATCTACC
Equus caballus trnH                   GTAAATATAGTTTAA-CAAAAACACTAGATTGTGGATCTAGAAACAGAAACT-TAATATTTCTTATTTACC
Pecari tajacu trnH                    GTAAATATAGTTTAA--AAAAACTCTAGATTGTGAACCTAGAAACAGAAGAC-TAAACCTTCTTATTTACC
Phacochoerus africanus trnH           GTAGATATAGTTTAA-CAAAAACCCTAGATTGTGGATCTAGTAATAGAAAAT-TAAATATTCTTATCTACC
Potamochoerus porcus trnH             GTAGATATAGTTTAA-CAAAAACACTAGATTGTGGATCCAGTAATAGAATCT-TAAATATTCTTATCTACC
Sus scrofa trnH                       GTAGATATAGTTTAA-TAAAAACCCTAGATTGTGAATCTAGTAATAGAAAAT-TAAATATTCTTATCTACC
Camelus bactrianus trnH               GTAAATATAGTTTAA-AAAGAACACTAGATTGTGAATCTAGTAATAAGAGAT-CAAACCTCTTTATTTACC
Camelus dromedarius trnH              GTAAATATAGTTTAA-AAAGAACACTAGATTGTGAATCTAGTAATAAGAGAT-CAAGCCTCTTTATTTACC
Lama guanicoe trnH                    GTAAGTATAGTTTAA-GAAGAACACTAGATTGTGAATCTAGCAGTAAGAGAT-TAAAACTCTTTACTTACC
Vicugna pacos trnH                    GTAAGTATAGTTTAA-GAAGAACACTAGATTGTGAATCTAGCAGTAAGAGAT-CAAAACTCTTTACCTACC
Hyemoschus aquaticus trnH             GTAAATATAGTTTAA-AAAAAACACTAGATTGTGAATCTAGCAATAGAAGCCCATCACCTTCTTATCTACC
Tragulus kanchil trnH                 GTAAATATAGTTTAA-AAAAAACGCTAGATTGTGAATCTAATAATAGAAGCC-CACACCTTCTTATTTACC
Giraffa camelopardalis trnH           GTAAATGTAGTTTAA-AAAAAACATTAGATTGTGAATCTAATAATAGAAGCTTACTACCCTCTCATTTACC
Okapia johnstoni trnH                 GTAAATATAGTTTAA-AAAAAACATTAGATTGTGAATCTAATAACAGAAGCTTACTGCCTTCTTATTTACC
Antilocapra americana trnH            GTAAATATAGTTTAA-AAAAAACATTAGATTGTGAATCTAAAAATAGAAGCTCGCCACCTTCTTATTTACC
Moschus moschiferus trnH              GTGAATATAGTTTAA-AAAAAACACTAGATTGTGAATCTAATGATAGAAGTCCACTACCTTCTTATTTACC
Muntiacus muntjak trnH                GTAAATATAGTTTAA-AAAAAACATTAGATTGTGAATCTAATAATAGAAACT-TATACCTTCTTATTTACC
Capreolus capreolus trnH              GTAAATATAGTTTAA-AAAAAACATTAGATTGTGAGTCTAACAATAGAAGCC-TATAACTTCTTATTCACC
Alces alces trnH                      GTAAATATAGTTTAA-AAAAAACACTAGACTGTGAATCTGATAATAGGAGTT-TATATCTCCTTATTTACC
Cervus elaphus trnH                   GTAAATATAGTTTAA-AAAAAACATTAGATTGTGGATCTAATAATAGAAGCT-TATATCTCCTTATTTACC
Dama dama trnH                        GTAAATATAGTTTAA-AAAAAACATTAGATTGTGAATCTAATAATAGAAGCT-TATATCTCCTTATTTACC
Boselaphus tragocamelus trnH          GTAAATATAGTTTAA-AAAAAACATTAGATTGTGAATCTAATAATAGAGGCTTATTACCCTCTTACTTACC
Tragelaphus oryx trnH                 GTAAATATAGTTTAA--AAAAACATTAGACTGTGAATCTAATAATAGAAGCTCACCACCTTCTTATTTACC
Bos taurus trnH                       GTAAATATAGTTTAA-CAAAAACATTAGATTGTGAATCTAACAATAGAAACTCATTACCTTCTTATTTACC
Bubalus bubalis trnH                  GTGAATATAGTTTAA-CAAAAACATTAGACTGTGAATCTAACAATAGAAGCCCATTGCCTTCTTATTTACC
Syncerus caffer trnH                  GTAAATATAGTTTAA-CAAAAACATTAGACTGTGAATCTAACAATAGAAGCCCATTGTCTTCTTATTTACC
Neotragus moschatus trnH              GTAAGTGTAGTTTAA-AAAAAACATTAGATTGTGAATCTAATAATAGAAGTCCACCACCTTCTCACTAACC
Alcelaphus buselaphus trnH            GTAGATATAGTTTAA-AAAAAACATTAGACTGTGAATCTAACAATAGAAGCCCGCAGCCTTCTTATTTACC
Oryx gazella trnH                     GTGAATATAGTTTAA-AAAAAACATTAGATTGTGAATCTAACAATAGAAGCCTACTATCTTCTTATTTACC
Pantholops hodgsonii trnH             GTAAATATAGTTTAA-AAAAAACATTAGATTGTGAATCTTACAATAGAAGTCCATTACCTTCTTATTTACC
Ovibos moschatus trnH                 GTAAATATAGTTTAA--AAAAACATTAGACTGTGAATCTAACAATAGAAGCCTACTATCTTCTTATTTACC
Capra hircus trnH                     GTAAATATAGTTTAAAAAAAAACATTAGATTGTGAATCTAACAATAGAAGCCTATCACCTTCTTATTTACC
Ovis aries trnH                       GTAAATATAGTTTAA--GAAAACATTAGATTGTGGATCTAATAATAGAAGACTATCACCTTCTTATTTACC
Cephalophus natalensis trnH           GTAAATATAGTTTAAGAAAAAACATTAGATTGTGAATCTAATAACAGAAGCT-GCCATCTTCTTATTTACC
Redunca fulvorufula trnH              GTAAATATAGTTTAA-AAAAAACATTAGATTGTGAATCTAACAATAGAAGCC-ACCACCTTCTTATTTACC
Ourebia ourebi trnH                   GTAAATATAGTTTTAAGAAAAACATTAGATTGTGAATCTAATAATAGAAGCCCGCTATCTTCTTATTTACC
Antilope cervicapra trnH              GTAAATATAGTTTAA-AAAAAACATTAGATTGTGAATCTAACAACAGAAGCTCGCTACCTTCTTATTTACC
Gazella gazella trnH                  GTAAATATAGTTTAA-AAAAAACATTAGATTGTGAATCTAACAACAGAAGCTCATTATCTTCTTATTTACC
Procapra gutturosa trnH               GTAAATATAGTTTAA-AAAAAACATTAGATTGTGAATCTAACAATAGAAGTTTAACATCTTCTTATTTACC
Raphicerus campestris trnH            GTGAATATAGTTTAA-ATAAAACATTAGATTGTGGATCTAGTAACAGAAGCT-TATACCTTCTTATTTACC
Hexaprotodon liberiensis trnH         GTAAGTATAGTTTAA-GAAAAACACTAGATTGTGAATCTATTAATAGAAGCTCCAACCCTTCTTACTTACC
Hippopotamus amphibius trnH           GTAAGCATAGTTTAA-GAAAAACACTAGATTGTGAATCTACCAATAGAAGCTCTAACCCTTCTTACTTACC
Eubalaena australis trnH              GTAAGTATAGTTTAA--AAAAATATTAGTTTGTGAAACTAATGACAGAAGGC-CAAAACTTCTTACTTACC
Eubalaena japonica trnH               GTAAGTATAGTTTAA-GAAAAATATTAGTTTGTGAAACTAATGACAGAAGAC-CAAAACTTCTTACTTACC
Balaena mysticetus trnH               GTAAGTATAGTTTAA-GAAAAATATTAGTTTGTGAAACTAACGATAGGAGAC-CAAAACTCCTTACTTACC
Caperea marginata trnH                GTAAGTATAGTTTAA-ACAAAACATTAGTTTGTGAAACTAATAATAGAAGAT-CAAAATTTCTTACTTACC
Eschrichtius robustus trnH            GTAAGTATAGTTTAA-TAAAAACACTAGTTTGTGAGACTAGCAATAGAAGAT-CAAAACTCCTTACTTACC
Balaenoptera acutorostrata trnH       GTAAGTATAGTTTAA-GAAAAACATTAGTTTGTGAAACTAACAATAGAAGACCTAAAACTTCTTACTTACC
Balaenoptera bonaerensis trnH         GTAAGTATAGTTTAA-GAAAGACATTAGTTTGTGAAACTAACGATAGAAGACCTAAAACTTCTTACTTACC
Balaenoptera physalus trnH            GTAAGTATAGTTTAA-AAAAGACGCTAGTTTGTGAAACTAACAATAGAAGAT-CAAAACTTCTTACTTACC
Megaptera novaeangliae trnH           GTAAGTATAGTTTAA-AGAAAACGCTAGTTTGTGAAACTAGCAATAGAAGAT-CAAAACCTCTTACTTACC
Balaenoptera musculus trnH            GTAAGTATAGTTTAA-AAAAGACATTAGTTTGTGAAACTAGCGATAGAAGAT-TAAAACTTCTTACTTACC
Balaenoptera omurai trnH              GTAAGTATAGTTTAA-AAAAGACATTAGTTTGTGAGACTAACAATAGAAGAC-CGAAACTTCTTACTTACC
Balaenoptera borealis trnH            GTAAGTATAGTTTAA-AAAAGATATTAGTTTGTGAGACTAACGATAGAAGAC-TAAAACTTCTTACTTACC
Balaenoptera brydei trnH              GTAAGTATAGTTTAA-AAAAGATGTTAGTTTGTGACACTAACGATAGAAGAC-CAAAACTTCTTACTTACC
Balaenoptera edeni trnH               GTAAGTATAGTTTAA-AAAAGATATTAGTTTGTGACACTAACGATAGAAGAC-CAAAACTTCTTACTTACC
Kogia breviceps trnH                  GTAAGTATAGTTTAA-AAAAAACACTAGCTTGTGGAGCCAGCAACAGAAGATA-AAAACTTCTTGCTTACC
Physeter macrocephalus trnH           GTAAATATAGTTTAA-GAAAAACACTAGCTTGTGGAGCCAGTAATAGAAGAC-TAAAACTTCTTATTTACC
Platanista minor trnH                 GTAAATATAGTTTAA-AAAAAACATTAGTTTGTGAAACTAACAACAGAAGAC-TGAAGCTTCTTATTTACC
Ziphius cavirostris trnH              GTAAATATAATTTAA-CAAAAATACTAGTTTGTGGAACTAGCAATAGAAGATATAGACCTTCTTATTTACC
Mesoplodon densirostris trnH          GTAAATATAATTTAA-CAAAAATTCTAGTTTGTGAAACTAGCAATAGAAGTTATAG-CCTTCTTATTTACC
Mesoplodon europaeus trnH             GTAAATATAATTTAA-TAAAAATACTAGTTTGTGAAACTAGCAATAGAGGATA-AAATCCTCTTATTTACC
**Mesoplodon grayi trnH               GTAAATATAATTTAA-TAAAAATTCTAGTTTGTGAAACTAGCAATAGGAGACTT-AATCTCCTTATTTACC
Berardius bairdii trnH                GTAAATATAATTTAA-CAAAAATACTAGTTTGTGGAACTAGCAATAGAAGAT-TAAATCTTCTTATTTACC
Hyperoodon ampullatus trnH            GTAAATATAATTTAA-TAAAAATACTAGTTTGTGAAGCTAGCAATAGAAGTCA-AAACCTTCTTATTTACC
Lipotes vexillifer trnH               GTAAGTATAGTTTAA--AAAAACACTAGCCTGTGAAGTTAGTAATAGAAGAT-TAAAACTTCTTACTTACC
Inia geoffrensis trnH                 GTAGACATAGTTTAA-ATAAAACATTAGTTTGTGAAACTAACAATAGAAGAT-CAAAACTTCTTACCTACC
Pontoporia blainvillei trnH           GTAGATGTAGTTTAA-AAAGAACACTAGTTTGTGAAGCTAGCAACAGAAGATA-AAACCTTCTTATCTACC
Monodon monoceros trnH                GTAAGTATAGTTTAA-AAAGAACATTAGCCCGTGAAGCTAAAAACAGAGGAC--AAAACTTCTTACTTACC
**Neophocaena asiaeorientalis trnH    GTAAGTATAGTTTAA-AAAGAACATTAGTCCGTGAAGCTAAAAACAGAAGAT--AGAACTTCTTACTTACC
Neophocaena phocaenoides trnH         GTAAGTATAGTTTAA-AAAGAACATTAGTCCGTGAAGCTAAAAACAGAAGAT--AGAACTTCTTACTTACC
Phocoena phocoena trnH                GTAAGTATAGTTTAA-AAAGAACATTAGTCCGTGAAGCTAAAAACAGAAGGC--AAAACTTCTTACTTACC
Cephalorhynchus heavisidii trnH       GTAAGTATAGTTTAA-AAAAACCATTAGTTTGTGAAACTAAAAACAGAAGATA-AAGCCTTCTTACTTACC
Sousa chinensis trnH                  GTAAGTATAGTTTAA-AAAAACCATTAGTTTGTGAAACTAAAAACAGAAGATA-AGACCTTCTTACTTACC
Stenella attenuata trnH               GTAAGTATAGTTTAA-AAAAACCATTAGTTTGTGAAACTAAAAACAGAAGATA-AGACCTTCTTACTTACC
Tursiops australis trnH               GTAAGTATAGTTTAA-AAAAACCATTAGTTTGTGGAACTAAAAACAGAAGATA-AAACCTTCTTACTTACC
Tursiops truncatus trnH               GTAAGTATAGTTTAA-AAAAACCATTAGTTTGTGAAACTAAAAACAGAAGATA-AGACCTTCTTACTTACC
Tursiops aduncus trnH                 GTAAGTATAGTTTAA-AAAAACCATTAGTTTGTGAAACTAAAAACAGAAGATA-AGACCTTCTTACTTACC
Delphinus capensis trnH               GTAAGTATAGTTTAA-AAAAACCATTAGTTTGTGAAACTAAAAACAGAAGATA-AGACCTTCTTACTTACC
Stenella coeruleoalba trnH            GTAAGTATAGTTTAA-AAAAACCATTAGTTTGTGAAACTAAAAACAGAAGATA-AGACCTTCTTACTTACC
Orcaella brevirostris trnH            GTAAGTATAGTTTAA-AAAAACCATTAGTTTGTGAAACTAAAAACAGAAGATA-AAACCTTCTTACTTACC
Orcaella heinsohni trnH               GTAAGTATAGTTTAA-AAAAACCATTAGCTTGTGAAACTAAAAACAGAAGATA-AAACCTTCTTACTTACC
Grampus griseus trnH                  GTAAGTATAGTTTAA-AAAAACCATTAGTTTGTGAAACTAAAAACAGAAGATATAAACCTTCTTACTTACC
Pseudorca crassidens trnH             GTAAGTATAGTTTAA-AAAAACCATTAGTTTGTGAAGCTAAAAACAGAAGATATAAACCTTCTTACTTACC
Feresa attenuata trnH                 GTAAGTATAGTTTAA-AAAAACCATTAGTTTGTGAAACTAAAAATAGAAGATATAAACCTTCTTACTTACC
Peponocephala electra trnH            GTAAGTATAGTTTAA--AAAACCGTTAGTTTGTGAAACTAAAAATAGAAGATATAAACCTTCTTACTTACC
Globicephala macrorhynchus trnH       GTAAGTATAGTTTAA-AAAAACCATTAGTTTGTGAAACTAAAAATAGAAGATATAAACCCTCTTACTTACC
Globicephala melas trnH               GTAAGTATAGTTTAA-AAAAACCATTAGTTTGTGAAACTAAAAATAGAAGATATAAACCCTCTTACTTACC
Lagenorhynchus albirostris trnH       GTAAGTATAGTTTAA-AAAAACCATTAGTTTGTGAAACTAAAAACAGAAGATACAAACCTTCTTACTTACC
Orcinus orca WNPTRU1 trnH             GTAAATATAGTTTAA-AAAAACCATTAGTTTGTGAGACTAAAAATAGAAGATA-AAGCCTTCTTATTTACC
Orcinus orca AntA1 trnH               GTAAATATAGTTTAA-AAAAACCATTAGTTTGTGAGACTAAAAATAGAAGATA-AAGCCTTCTTATTTACC
Orcinus orca AntB1 trnH               GTAAATATAGTTTAA-AAAAACCATTAGTTTGTGAGACTAAAAATAGAAGATA-AAGCCTTCTTATTTACC
Orcinus orca AntC1 trnH               GTAAATATAGTTTAA-AAAAACCATTAGTTTGTGAGACTAAAAATAGAAGATA-AAGCCTTCTTATTTACC
Orcinus orca ENAHN1 trnH              GTAAATATAGTTTAA-AAAAACCATTAGTTTGTGAGACTAAAAATAGAAGATA-AAGCCTTCTTATTTACC
Orcinus orca CNPNRAL trnH             GTAAATATAGTTTAA-AAAAACCATTAGTTTGTGAGACTAAAAATAGAAGATA-AAGCCTTCTTATTTACC
Orcinus orca ENPOAL2 trnH             GTAAATATAGTTTAA-AAAAACCATTAGTTTGTGAGACTAAAAATAGAAGATA-AAGCCTTCTTATTTACC
                                      1234567  1234      4321 12345  ant  54321    12345        543217654321d
                                      0000000  1111      2222 22222  333  33344    44445        566666666667
                                      1234567  0123      0123 56789  234  78901    67890        901234567890 1
```

N, the most common base for the position.

N, half compensatory base change in the stem pair (e.g. T – G vs C – G; A-T vs G-T).

N, N, half compensatory base change in the stem pair exhibiting a mismatch (e.g. T–A vs A–A). Different colours are used to better differentiate the changes.

N, fully compensatory base change in the stem pair exhibiting a mismatch (e.g. C–G vs T–T).

N, type I fully compensatory base change in the stem pair (i.e. purine – pyrimidine vs purine – pyrimidine, e.g. G – C vs A – T)

N, N, type II fully compensatory base change in the stem pair (i.e. purine – pyrimidine vs pyrimidine – purine, e.g. A – T vs T – A). Different colours are used to better differentiate the changes.

N, a mismatch in the in the stem pair; N, substitution pattern not modelled; *, pair in the stem in which a mismatch is prominent. N, N, molecular signature for a taxon.

X, position 1-7 in the acceptor stem; X position 1-4 in the DHU stem; X position 1-5 in the anticodon stem; X position 1-5 in the TΨC stem; ant, anticodon; d, discriminator nucleotide.

trnI (**ILE**) multiple alignment

```
                                             10        20        30        40        50        60        70
                                              |         |         |         |         |         |         |
                                     1234567  123      321 12345  ant  54321     12345        543217654321d
Ceratotherium simum trnI             AGAAATATGTCTGATAAAAGAGTTACTTTGATAGAGTAAATAATAGAGGTTCA-AGCCCTCTTATTTCTA
Equus caballus trnI                  GGAAATATGTCTGACAAAAGAGTTACTTTGATAGAGTAAAACATAGAGGCTCA-AACCCTCTTATTTCTA
Pecari tajacu trnI                   AGAAATATGTCTGACAAAAGAGTTACTTTGATAGAGTAAAAAATAGAGGTTTATAACCCTCTTATTTCTA
Phacochoerus africanus trnI          AGAAATATGTCTGACAAAAGAGTTACTTTGATAGAGTAAAAAATAGAGGTTCA-AGTCCTCTTATTTCTA
Potamochoerus porcus trnI            AGAAATATGTCTGATAAAAGAGTTACTTTGATAGAGTAAAAAATAGAGGTTCA-AACCCTCTTATTTCTA
Sus scrofa trnI                      AGAAATATGTCTGATAAAAGAGTTACTTTGATAGAGTAAAAAATAGAGGTTCA-AACCCTCTTATTTCTA
Camelus bactrianus trnI              AGAAATATGTCTGACAAAAGAGTTACTTTGATAGAGTAAATAATAGAGGTTTG-AGCCCTCTTGTTTCTA
Camelus dromedarius trnI             AGAAATATGTCTGACAAAAGAGTTACTTTGATAGAGTAAATAATAGAGGTTTA-AGTCCTCTTGTTTCTA
Lama guanicoe trnI                   AGAAATATGTCTGACAAAAGAATTACTTTGATAGAGTAAATAATAGAGGTTTA-AGCCCTCTTATTTCTA
Vicugna pacos trnI                   AGAAATATGTCTGACAAAAGAATTACTTTGATAGAGTAAATAATAGAGGTTTA AGCCCTCTTATTTCTA
Hyemoschus aquaticus trnI            AGAAACATGTCTGATAAAAGAATTACTTTGATAGAGTAAATAATAGAGGTTCA AGCCCTCTTGTTTCTA
Tragulus kanchil trnI                AGAAACATGTCTGATAAAAGAGTTACTTTGATAGAGTAAATAATAGAGGTTTA AGCCCCCTTGTTTCTA
Giraffa camelopardalis trnI          AGAAATATGTCTGATAAAAGAGTTACTTTGATAGAGTAAATAATAGAGGTTTA AATCCTCTTATTTCTA
Okapia johnstoni trnI                AGAAATATGTCTGACAAAAGAGTTACTTTGATAGAGTAAATAATAGAGGTTTA AATCCTCTTATTTCTA
Antilocapra americana trnI           AGAAATATGTCTGACAAAAGAGTTACTTTGATAGAGTAAATAATAGAGGTTTC-AACCCTCTTATTTCTA
Moschus moschiferus trnI             AGAAATATGTCTGACAAAAGAGTTACTTTGATAGAGTAAATAATAGAGGTTTA-AATCCTCTTATTTCTA
Muntiacus muntjak trnI               AGAAATATGTCTGACAAAAGAGTTACTTTGATAGAGTAAATAATAGAGGTTCA-AGCCCTCTTATTTCTA
Capreolus capreolus trnI             AGAAATATGTCTGACAAAAGAGTTACTTTGATAGAGTAAATAATAGAGGTTTA-AACCCTCTTATTTCTA
Alces alces trnI                     AGAAATATGTCTGATAAAAGAATTACTTTGATAGAGTAAATAATAGAGGTTTA-AGCCCTCTTATTTCTA
Cervus elaphus trnI                  AGAAATATGTCTGACAAAAGAGTTACTTTGATAGAGTAAACAATAGAGGTTTA-AGCCCTCTTATTTCTA
Dama dama trnI                       AGAAATATGTCTGACAAAAGAGTTACTTTGATAGAGTAAATAATAGAGGTTTA-AGCCCTCTTATTTCTA
Boselaphus tragocamelus trnI         AGAAATATGTCTGATAAAAGAGTTACTTTGATAGAGTAAATAATAGAGGTTTA-AGCCCTCTTATTTCTA
Tragelaphus oryx trnI                AGAAATATGTCTGACAAAAGAGTTACTTTGATAGAGTAAATAATAGAGGTCAA-AACCCTCTTATTTCTA
Bos taurus trnI                      AGAAATATGTCTGACAAAAGAGTTACTTTGATAGAGTAAATAATAGAGGTTCA-AACCCTCTTATTTCTA
Bubalus bubalis trnI                 AGAAATATGTCTGACAAAAGAGTTACTTTGATAGAGTAAATAATAGAGGTTTA-AACCCTCTTATTTCTA
Syncerus caffer trnI                 GGAAATATGTCTGACAAAAGAGTTACTTTGATAGAGTAAATAATAGAGGTTTA-AATCCTCTTATTTCTA
Neotragus moschatus trnI             AGAAATATGTCTGACAAAAGAATTACTTTGATAGAGTAAATAATAGAGGTTTA-AGCCCTCTTATTTCTA
Alcelaphus buselaphus trnI           AGAAATATGTCTGACAAAAGAGTTACTTTGATAGAGTAAATAATAGAGGTTTA-AACCCTCTTATTTCTA
Oryx gazella trnI                    AGAAATATGTCTGACAAAAGAGTTACTTTGATAGAGTAAATAATAGAGGTTCA-AGCCCTCTTATTTCTA
Pantholops hodgsonii trnI            AGAAATATGTCTGACAAAAGAGTTACTTTGATAGAGTAAATAATAGAGGTTTA-AATCCTCTTATTTCTA
Ovibos moschatus trnI                AGAAATATGTCTGATAAAAGAATTACTTTGATAGAGTAAATAATAGAGGTTTA-AGCCCTCTTATTTCTA
Capra hircus trnI                    AGAAATATGTCTGACAAAAGAGTTACTTTGATAGAGTAAATAATAGAGGTTTA-AATCCTCTTATTTCTA
Ovis aries trnI                      AGAAATATGTCTGACAAAAGAGTTACTTTGATAGAGTAAATAATAGAGGTTTA-AATCCTCTTATTTCTA
Cephalophus natalensis trnI          AGAAATATGTCTGACAAAAGAGTTACTTTGATAGAGTAAATAATAGAGGTTCA-AGCCCTCTTATTTCTA
Redunca fulvorufula trnI             AGAAATATGTCTGACAAAAGAGTTACTTTGATAGAGTAAATAATAGAGGTTCA-AATCCTCTTATTTCTA
Ourebia ourebi trnI                  AGAAATATGTCTGATAAAAGAGTTACTTTGATAGAGTAAATAATAGAGGTCCA-AACCCTCTTATTTCTA
Antilope cervicapra trnI             AGAAATATGTCTGATAAAAGAGTTACTTTGATAGAGTAAATAATAGAGGTTTA-AGCCCTCTTATTTCTA
Gazella gazella trnI                 AGAAATATGTCTGACAAAAGAGTTACTTTGATAGAGTAAATAATAGAGGTTCA-AGCCCTCTTATTTCTA
Procapra gutturosa trnI              AGAAATATGTCTGATAAAAGAATTACTTTGATAGAGTAAATAATAGAGGTTTA-AGCCCTCTTATTTCTA
Raphicerus campestris trnI           AGAAATATGTCTGACAAAAGAGTTACTTTGATAGAGTAAATAATAGAGGTTTG-AATCCTCTTATTTCTA
Hexaprotodon liberiensis trnI        AGAAATATGTCTGACAAAAGAATTACTTTGATAGAGTAAATAATAGAGGTTTA AGCCCTCTTATTTCTA
Hippopotamus amphibius trnI          AGAAATATGTCTGACAAAAGAATTACTTTGATAGAGTAAATAATAGAGGTTCA AGCCCTCTTATTTCTA
Eubalaena australis trnI             AGAAATATGTCTGACAAAAGAGTTACTTTGATAGAGTAAATAATAGAGGTTTA-AACCCTCTTATTTCTA
Eubalaena japonica trnI              AGAAATATGTCTGACAAAAGAGTTACTTTGATAGAGTAAATAATAGAGGTTTA-AACCCTCTTATTTCTA
Balaena mysticetus trnI              AGAGATATGTCTGATAAAAGAGTTACTTTGATAGAGTAAATAATAGAGGTTTA-AACCCTCTTATTTCTA
Caperea marginata trnI               AGAAATATGTCTGATAAAAGAGTTACTTTGATAGAGTAAATAATAGAGGCCTA-AATCCTCTTATTTCTA
Eschrichtius robustus trnI           AGAAATATGTCTGATAAAAGAGTTACTTTGATAGAGTAAATAATAGAGGCCTACAATCCTCTTATTTCTA
Balaenoptera acutorostrata trnI      AGAAATATGTCTGACAAAAGAGTTACTTTGATAGAGTAAATAATAGAGGCCCATAATCCTCTTATTTCTA
Balaenoptera bonaerensis trnI        AGAAATATGTCTGACAAAAGAGTTACTTTGATAGAGTAAATAATAGAGGCCTA-AATCCTCTTATTTCTA
Balaenoptera physalus trnI           AGAAATATGTCTGATAAAAGAGTTACTTTGATAGAGTAAATAATAGAGGCCCA-AATCCTCTTATTTCTA
Megaptera novaeangliae trnI          AGAAATATGTCTGATAAAAGAGTTACTTTGATAGAGTAAATAATAGAGGCCTA-AACCCTCTTATTTCTA
Balaenoptera musculus trnI           AGAAATATGTCTGATAAAAGAGTTACTTTGATAGAGTAAATAATAGAGGCCTA-AATCCTCTTATTTCTA
Balaenoptera omurai trnI             AGAAATATGTCTGACAAAAGAGTTACTTTGATAGAGTAAATAATAGAGGCCTA-AATCCTCTTATTTCTA
Balaenoptera borealis trnI           AGAAATATGTCTGATAAAAGAGTTACTTTGATAGAGTAAATAATAGAGGCCTATAATCCTCTTATTTCTA
Balaenoptera brydei trnI             AGAAATATGTCTGACAAAAGAGTTACTTTGATAGAGTAAATAATAGAGGCCTATAATCCTCTTATTTCTA
Balaenoptera edeni trnI              AGAAATATGTCTGATAAAAGAGTTACTTTGATAGAGTAAATAATAGAGGCCTATAACCCTCTTATTTCTA
Kogia breviceps trnI                 AGAAATATGTCTGACAAAAGAGTTACTTTGATAGAGTAAATAATAGGGGTTTA-AACCCCCTTGTTTCTA
Physeter macrocephalus trnI          AGAAATATGTCTGATAAAAGAGTTACTTTGATAGAGTAAATAATAGAGGCTCA-AATCCTCTTATTTCTA
Platanista minor trnI                AGAAATATGTCTGACAAAAGAGTTACTTTGATAGAGTAAATAATAGAGGTTTA-AATCCTCTTATTTCTA
Ziphius cavirostris trnI             AGAAATATGTCTGATAAAAGAGTTACTTTGATAGAGTAAATAATAGAGGTTCA-AACCCTCTTATTTCTA
Mesoplodon densirostris trnI         AGAAATATGTCTGACAAAAGAATTACTTTGATAGAGTAAACAATAGAGGTTTA-AGCCCTCTTATTTCTA
Mesoplodon europaeus trnI            AGAAATATGTCTGACAAAAGAATTACTTTGATAGAGTAAATAATAGAGGTTTA-AGCCCTCTTATTTCTA
**Mesoplodon grayi trnI              AGAAATATGTCTGACAAAAGAATTACTTTGATAGAGTAAACAATAGAGGTTTA-AGCCCTCTTATTTCTA
Berardius bairdii trnI               AGAAATATGTCTGACAAAAGAATTACTTTGATAGAGTAAATAATAGAGGTTTA-AACCCTCTTATTTCTA
Hyperoodon ampullatus trnI           AGAGATATGTCTGACAAAAGAATTACTTTGATAGAGTAAACAATAGAGGTTTA-AGCCCTCTTATTTCTA
Lipotes vexillifer trnI              AGAAATATGTCTGACAAAAGAATTACTTTGATAGAGTAAATAATAGAGGTTTA-AATCCCCTTATTTCTA
Inia geoffrensis trnI                AGAAATATGTCTGACAAAAGAATTACTTTGATAGAGTAAATAATAGAGGTTCG-AACCCTCTTATTTCTA
Pontoporia blainvillei trnI          AGAAATATGTCTGAT-AAAGAGTTACTTTGATAGAGTAAATAATAGAGGTTTA--GCCCTCTTATTTCTA
Monodon monoceros trnI               AGAAATATGTCTGACAAAAGAGTTACTTTGATAGAGTAAATAATAGAGGGTTA-AATCCCCTTATTTCTA
**Neophocaena asiaeorientalis trnI   AGAAATATGTCTGACAAAAGAATTACTTTGATAGAGTAAATAATAGAGGTTTG-AGCCCTCTTATTTCTA
Neophocaena phocaenoides trnI        AGAAATATGTCTGACAAAAGAATTACTTTGATAGAGTAAATAATAGAGGTTTG-AGCCCTCTTATTTCTA
Phocoena phocoena trnI               AGAAATATGTCTGACAAAAGAATTACTTTGATAGAGTAAATAATAGAGGTTTA-AGCCCTCTTATTTCTA
Cephalorhynchus heavisidii trnI      AGAAATATGTCTGACAAAAGAATTACTTTGATAGAGTAAATAATAGAGGTTTA-AGTCCTCTTATTTCTA
Sousa chinensis trnI                 AGAAATATGTCTGACAAAAGAGTTACTTTGATAGAGTAAATAATAGAGGCTTA-AATCCTCTTATTTCTA
Stenella attenuata trnI              AGAAATATGTCTGACAAAAGAGTTACTTTGATAGAGTAAATAATAGAGGTTTA-AATCCTCTTATTTCTA
Tursiops australis trnI              AGAAATATGTCTGACAAAAGAGTTACTTTGATAGAGTAAATAATAGAGGTTTA-AATCCTCTTATTTCTA
Tursiops truncatus trnI              AGAAATATGTCTGACAAAAGAGTTACTTTGATAGAGTAAATAATAGAGGTTTA-AATCCTCTTATTTCTA
Tursiops aduncus trnI                AGAAATATGTCTGACAAAAGAATTACTTTGATAGAGTAAATAATAGAGGTTCA-AGTCCTCTTATTTCTA
Delphinus capensis trnI              AGAAATATGTCTGACAAAAGAGTTACTTTGATAGAGTAAATAATAGAGGTTTA-AGTCCTCTTATTTCTA
Stenella coeruleoalba trnI           AGAAATATGTCTGACAAAAGAGTTACTTTGATAGAGTAAATAATAGAGGTTTA-AATCCTCTTATTTCTA
Orcaella brevirostris trnI           AGAAATATGTCTGACAAAAGAATTACTTTGATAGAGTAAATAATAGAGGTTTA-AGTCCTCTTATTTCTA
Orcaella heinsohni trnI              AGAAATATGTCTGACAAAAGAATTACTTTGATAGAGTAAATAATAGAGGTTTA-AGTCCTCTTATTTCTA
Grampus griseus trnI                 AGAAATATGTCTGATAAAAGAATTACTTTGATAGAGTAAATAATAGAGGTTTA-AATCCTCTTATTTCTA
Pseudorca crassidens trnI            AGAAATATGTCTGACAAAAGAATTACTTTGATAGAGTAAATAATAGAGGTTTA-AATCCTCTTATTTCTA
Feresa attenuata trnI                AGAAATATGTCTGACAAAAGAATTACTTTGATAGAGTAAATAATAGAGGTTTA-AGTCCTCTTATTTCTA
Peponocephala electra trnI           AGAAATATGTCTGACAAAAGAATTACTTTGATAGAGTAAATAATAGAGGTTTA-AGCCCTCTTATTTCTA
Globicephala macrorhynchus trnI      AGAAATATGTCTGACAAAAGAATTACTTTGATAGAGTAAATAATAGAGGTTTA-AGTCCTCTTATTTCTA
Globicephala melas trnI              AGAAATATGTCTGACAAAAGAATTACTTTGATAGAGTAAATAATAGAGGTTTA-AGTCCTCTTATTTCTA
Lagenorhynchus albirostris trnI      AGAAATATGTCTGACAAAAGAATTACTTTGATAGAGTAAATAATAGAGGTTTA-AGTCCTCTTATTTCTA
Orcinus orca WNPTRU1 trnI            AGAAATATGTCTGACAAAAGAATTACTCTGATGGAGTAAATAATAGAGGTTCA-AGTCCTCTTATTTCTA
Orcinus orca AntA1 trnI              AGAAATATGTCTGACAAAAGAATTACTCTGATGGAGTAAATAATAGAGGTTCA-AGTCCTCTTATTTCTA
Orcinus orca AntB1 trnI              AGAAATATGTCTGACAAAAGAATTACTCTGATGGAGTAAATAATAGAGGTTCA-AGTCCTCTTATTTCTA
Orcinus orca AntC1 trnI              AGAAATATGTCTGACAAAAGAATTACTCTGATGGAGTAAATAATAGAGGTTCA-AGTCCTCTTATTTCTA
Orcinus orca ENAHN1 trnI             AGAAATATGTCTGACAAAAGAATTACTCTGATGGAGTAAATAATAGAGGTTCA-AGTCCTCTTATTTCTA
Orcinus orca CNPNRAL trnI            AGAAATATGTCTGACAAAAGAATTACTCTGATGGAGTAAATAATAGAGGTTCA-AGTCCTCTTATTTCTA
Orcinus orca ENPOAL2 trnI            AGAAATATGTCTGACAAAAGAATTACTCTGATGGAGTAAATAATAGAGGTTCA-AGTCCTCTTATTTCTA
                                     1234567  123      321 12345  ant  54321     12345        543217654321d
                                     0000000  111      122 22222  333  33333     44444        5566666666667
                                     1234567  012      901 34567  012  56789     56789        8901234567890
```

N, the most common base for the position.

N, half compensatory base change in the stem pair (e.g. T – G vs C – G; A-T vs G-T).

N, N, half compensatory base change in the stem pair exhibiting a mismatch (e.g. T–A vs A–A). Different colours are used to better differentiate the changes.

N, fully compensatory base change in the stem pair exhibiting a mismatch (e.g. C–G vs T–T).

N, type I fully compensatory base change in the stem pair (i.e. purine – pyrimidine vs purine – pyrimidine, e.g. G – C vs A – T).

N, N, type II fully compensatory base change in the stem pair (i.e. purine – pyrimidine vs pyrimidine – purine, e.g. A – T vs T – A). Different colours are used to better differentiate the changes.

N, a mismatch in the in the stem pair; N, substitution pattern not modelled; *, pair in the stem in which a mismatch is prominent. N, N, molecular signature for a taxon.

X, position 1-7 in the acceptor stem; X position 1-3 in the DHU stem; X position 1-5 in the anticodon stem; X position 1-5 in the TΨC stem; **ant**, anticodon; **d**, discriminator nucleotide.

***trnK* (LYS) multiple alignment**

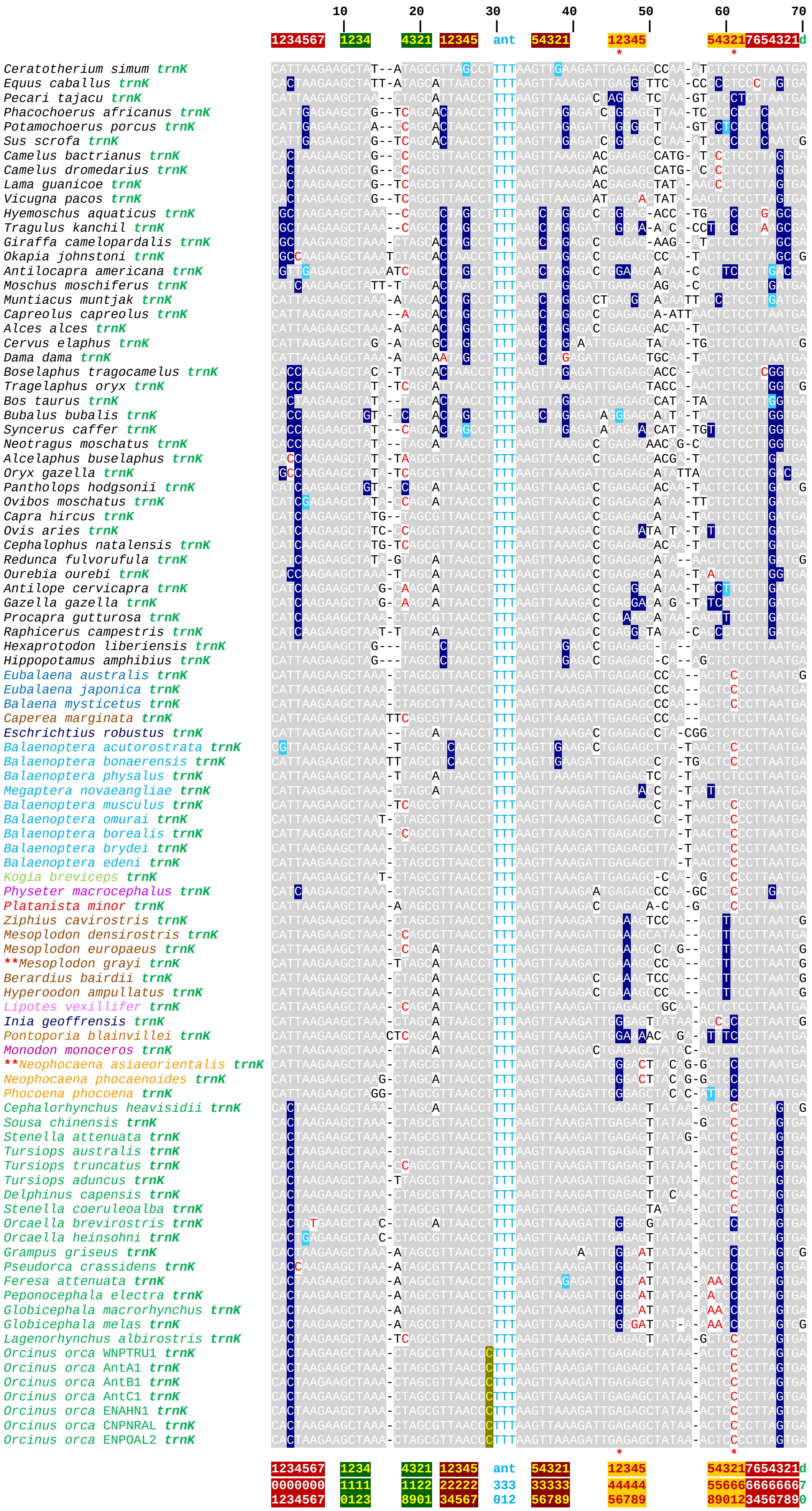

N, the most common base for the position.
N, half compensatory base change in the stem pair (e.g. T – G vs C – G; A-T vs G-T).
N, N, half compensatory base change in the stem pair exhibiting a mismatch (e.g. T–A vs A–A). Different colours are used to better differentiate the changes.
N, fully compensatory base change in the stem pair exhibiting a mismatch (e.g. C–G vs T–T).
N, type I fully compensatory base change in the stem pair (i.e. purine – pyrimidine vs purine – pyrimidine, e.g. G – C vs A – T)
N, N, type II fully compensatory base change in the stem pair (i.e. purine – pyrimidine vs pyrimidine – purine, e.g. A – T vs T – A). Different colours are used to better differentiate the changes.
N, a mismatch in the in the stem pair; N, substitution pattern not modelled; *, pair in the stem in which a mismatch is prominent. N, N, molecular signature for a taxon.
X, position 1-7 in the acceptor stem; X position 1-4 in the DHU stem; X position 1-5 in the anticodon stem; X position 1-5 in the TΨC stem; ant, anticodon; d, discriminator nucleotide.

trnL1 [LEU (L1, CUN)] multiple alignment

```
                                                 10        20        30        40        50        60        70
                                                  |         |         |         |         |         |         |
                                          1234567 1234         4321 12345 ant 54321     1234        43217654321d
Ceratotherium simum trnL1                 ACTTTTAAAGGATGGTAGT-TATCCGTTGGTCTTAGGAATCAAAA-AATTGGTGCAACTCCAAATAAAAGTA
Equus caballus trnL1                      ACTTTTAAAGGATAGGAGC-TATCCGTTGGTCTTAGGAACCAAAA-AATTGGTGCAACTCCAAATAAAAGTA
Pecari tajacu trnL1                       ACTTTTAAAGGATAGCAGT-TATCCGTTGGTCTTAGGAACCAAAA-ACTTGGTGCAACTCCAAATAAAAGTA
Phacochoerus africanus trnL1              ACTTTTAAAGGATAACAGC-TATCCGTTGGTCTTAGGAACCAAAA-AATTGGTGCAACTCCAAATAAAAGTA
Potamochoerus porcus trnL1                ACTTTTAAAGGATAACAGT-TATCCGTTGGTCTTAGGAACCAAAA-AATTGGTGCAATTCCAAATAAAAGTA
Sus scrofa trnL1                          ACTTTTAAAGGATAACAGC-TATCCGTTGGTCTTAGGAACCAAAA-AATTGGTGCAACTCCAAATAAAAGTA
Camelus bactrianus trnL1                  ACTTTTAAAGGATAGAAGT-AATCCGTTGGCCTTAGGAGCCAAAA-AATTGGTGCAACTCCAAATAAAAGTA
Camelus dromedarius trnL1                 ACTTTTAAAGGATAGAAGG-AATCCGTTGGCCTTAGGAGCCAAAA-AATTGGTGCAACTCCAAATAAAAGTA
Lama guanicoe trnL1                       ACTTTTAAAGGATAGAAGT-AATCCGTTGGCCTTAGGAGCCAAAA-AATTGGTGCAACTCCAAATAAAAGTA
Vicugna pacos trnL1                       ACTTTTAAAGGATAGAAGT-AATCCGTTGGCCTTAGGAGCCAAAA-AATTGGTGCAACTCCAAATAAAAGTA
Hyemoschus aquaticus trnL1                ACTTTTAAAGGATAACAGT-AATCCATTGGCCTTAGAAGCCAAAA-AATTGGTGCAACTCCAAATAAAAGTA
Tragulus kanchil trnL1                    ACTTTTAAAGGATAGAAGT-AATCCGTTGGTCTTAGGAGCCAAAA-AATTGGTGCAACTCCAAGTAAAAGTA
Giraffa camelopardalis trnL1              ACTTTTAAAGGATAGTAGT-CATCCGTTGGTCTTAGGAACCAAAA-AATTGGTGCAACTCCAAATAAAAGTA
Okapia johnstoni trnL1                    ACTTTTAAAGGATGGAAGT-TATCCGTTGGTCTTAGGAACCGAAA-AATTGGTGCAACTCCAAATAAAAGTA
Antilocapra americana trnL1               ACTTTTAAAGGATAGTAGT-TATCCGTTGGTCTTAGGAACCAAAA-AATTGGTGCAACTCCAAATAAAAGTA
Moschus moschiferus trnL1                 ACTTTTAAAGGATAGTAGT-TATCCATTGGTCTTAGGAACCAAAA-AATTGGTGCAACTCCAAATAAAAGTA
Muntiacus muntjak trnL1                   ACTTTTAGAGGATGGTAGA-TATCCGTTGGTCTTAGGAATCAAA--AATTGGTGCAACTCCAAATAAAAGTA
Capreolus capreolus trnL1                 ACTTTTAAAGGATAGTAGT-AATCCATTGGTCTTAGGAACCAAAA-AATTGGTGCAACTCCAAATAAAAGTA
Alces alces trnL1                         ACTTTTAGAGGATGGCAGT-AATCCGTTGGTCTTAGGAATCAAAA-AATTGGTGCAACTCCAAATAAAAGTA
Cervus elaphus trnL1                      ACTTTTAGAGGATGACAGA-AATCCGTTGGTCTTAGGAACCAAAA-AATTGGTGCAACTCCAAATAAAAGTA
Dama dama trnL1                           ACTTTTAGAGGATGACAGA-AATCCGTTGGTCTTAGGAACCAAAA-AATTGGTGCAACTCCAAATAAAAGTA
Boselaphus tragocamelus trnL1             ACTTTTAAAGGATGGAAGT-TATCCGTTGGTCTTAGGAACCAAAA-AATTGGTGCAACTCCAAATAAAAGTA
Tragelaphus oryx trnL1                    ACTTTTAAAGGATAGCAGT-TATCCGTTGGTCTTAGGAACCAAAA-AATTGGTGCAACTCCAAATAAAAGTA
Bos taurus trnL1                          ACTTTTAAAGGATAGTAGTTTATCCGTTGGTCTTAGGAACCAAAA-AATTGGTGCAACTCCAAATAAAAGTA
Bubalus bubalis trnL1                     ACTTTTAAAGGATAGTAGT-TATCCGTTGGTCTTAGGAGCCAAAA-AATTGGTGCAACTCCAAATAAAAGTA
Syncerus caffer trnL1                     ACTTTTAAAGGATAGTAGT-TATCCGTTGGTCTTAGGAACCAAAA-AATTGGTGCAACTCCAAATAAAAGTA
Neotragus moschatus trnL1                 ACTTTTAAAGGATAGTAGT-TATCCATTGGTCTTAGGAACCAAAA-AATTGGTGCAACTCCAAATAAAAGTA
Alcelaphus buselaphus trnL1               ACTTTTAAAGGATAGTAGT-TATCCATTGGTCTTAGGAACCAAAA-AATTGGTGCAACTCCAAATAAAAGTA
Oryx gazella trnL1                        ACTTTTAAAGGATGGTAGT-TATCCATTGGTCTTAGGAACCAAAA-AATTGGTGCAACTCCAAATAAAAGTA
Pantholops hodgsonii trnL1                ACTTTTAAAGGATAGTAGT-TATCCATTGGTCTTAGGAACCAAAA-AATTGGTGCAACTCCAAATAAAAGTA
Ovibos moschatus trnL1                    ACTTTTAAAGGATGGCAGT-TATCCATTGGTCTTAGGAACCAAAA-AATTGGTGCAACTCCAAATAAAAGTA
Capra hircus trnL1                        ACTTTTAAAGGATAGTAGT-TATCCGTTGGTCTTAGGAGCCAAAA-AATTGGTGCAACTCCAAATAAAAGTA
Ovis aries trnL1                          ACTTTTAAAGGATAGTAGT-TATCCATTGGTCTTAGGAACCAAAA-AATTGGTGCAACTCCAAATAAAAGTA
Cephalophus natalensis trnL1              ACTTTTAAAGGATAGTAGT-TATCCATTGGTCTTAGGAACCAAAA-AATTGGTGCAACTCCAAATAAAAGTA
Redunca fulvorufula trnL1                 GCTTTTAAAGGATAGAAGT-TATCCGTTGGTCTTAGGAACCAAAA-AATTGGTGCAACTCCAAATAAAAGCA
Ourebia ourebi trnL1                      ACTTTTAAAGGATGGTAGT-TATCCGTTGGCCTTAGGAGCCAAAA-AATTGGTGCAACTCCAAATAAAAGTA
Antilope cervicapra trnL1                 ACTTTTAAAGGATAGTAGT-TATCCATTGGCCTTAGGAGCCAAAA-AATTGGTGCAACTCCAAATAAAAGTA
Procapra gutturosa trnL1                  ACTTTTAAAGGATAGAAGT-TATCCATTGGTCTTAGGAACCAAAA-AATTGGTGCAACTCCAAATAAAAGTA
Raphicerus campestris trnL1               ACTTTTAAAGGATAGTAGT-TATCCGTTGGTCTTAGGAACCAAAA-AATTGGTGCAACTCCAAATAAAAGTA
Gazella gazella trnL1                     ACTTTTAAAGGATAGAAGT-CATCCGTTGGCCTTAGGAGCCAAAA-AATTGGTGCAACTCCAAATAAAAGTA
Hexaprotodon liberiensis trnL1            GCTTTTAAAGGATGGTAGC-TATCCATTGGTCTTAGGAGCCAAAG-AATTGGTGCAACTCCAAATAAAAGCA
Hippopotamus amphibius trnL1              GCTTTTAAAGGATGGTAGC-TATCCATTGGTCTTAGGAACCAAAA-AATTGGTGCAACTCCAAATAAAAGCA
Eubalaena australis trnL1                 ACTTTTACAGGATAGTAGT-TATCCATTGGTCTTAGGAACCAAAA-AATTGGTGCAACTCCAAATAAAAGTA
Eubalaena japonica trnL1                  ACTTTTACAGGATAGTAGT-TATCCATTGGTCTTAGGAACCAAAA-AATTGGTGCAACTCCAAATAAAAGTA
Balaena mysticetus trnL1                  ACTTTTACAGGATAGTAGT-TATCCATTGGTCTTAGGAACCAAAA-AATTGGTGCAACTCCAAATAAAAGTA
Caperea marginata trnL1                   ACTTTTATAGGATAGCAGT-AATCCATTGGTCTTAGGAACCAAAA-ATTTGGTGCAACTCCAAATAAAAGTA
Eschrichtius robustus trnL1               ACTTTTACAGGATGGTAGC-CATCCATTGGTCTTAGGAACCAAAA-ATTTGGTGCAACTCCAAATAAAAGTA
Balaenoptera acutorostrata trnL1          ACTTTTACAGGATAACAGT-TATCCATTGGTCTTAGGAACCAAAA-AATTGGTGCAACTCCAAATAAAAGTA
Balaenoptera bonaerensis trnL1            ACTTTTACAGGATAACAGT-TATCCATTGGTCTTAGGAACCAAAAAAATTGGTGCAACTCCAAATAAAAGTA
Balaenoptera physalus trnL1               ACTTTTACAGGATAGTAGT-TATCCATTGGTCTTAGGAGCCAAAA-AATTGGTGCAACTCCAAATAAAAGTA
Megaptera novaeangliae trnL1              ACTTTTACAGGATAGTAGT-TATCCATTGGTCTTAGGAACCAAAA-AATTGGTGCAACTCCAAATAAAAGTA
Balaenoptera musculus trnL1               ACTTTTACAGGATAGTAGT-TATCCATTGGTCTTAGGAACCAAAA-AATTGGTGCAACTCCAAATAAAAGTA
Balaenoptera omurai trnL1                 ACTTTTACAGGATAGTAGT-TATCCATTGGTCTTAGGAACCAAAA-AATTGGTGCAACTCCAAATAAAAGTA
Balaenoptera borealis trnL1               ACTTTTATAGGATAGTAGT-TATCCCTTGGTCTTAGGAACCAAAA-AATTGGTGCAACTCCAAATAAAAGTA
Balaenoptera brydei trnL1                 ACTTTTATAGGATAGTAGT-TATCCATTGGTCTTAGGAACCAAAG-AATTGGTGCAACTCCAAATAAAAGTA
Balaenoptera edeni trnL1                  ACTTTTATAGGATAGTAGT-TATCCATTGGTCTTAGGAACCAAAA-AATTGGTGCAACTCCAAATAAAAGTA
Kogia breviceps trnL1                     ACTTTTAAAGGATGGTAGT-TATCCGTTGGCCTTAGGAGCCAAAA-AATTGGTGCAACTCCAAATAAAAGTA
Physeter microcephalus trnL1              GCTTTTAAAGGATGGGAGT-TATCCATTGGCCTTAGGAGCCAAAA-AATTGGTGCAACTCCAAATAAAAGTA
Platanista minor trnL1                    ACTTTTAAAGGATAATAGT-TATCCATTGGCCTTAGGAGCCAAAA-AATTGGTGCAAGTCCAAATAAAAGTA
Ziphius cavirostris trnL1                 ACTTTTAAAGGATAGAAGT-TATCCGTTGGTCTTAGGAACCAAAA-ACTTGGTGCAACTCCAAATAAAAGTA
Mesoplodon densirostris trnL1             ACTTTTAAAGGATAGAAGT-TATCCATTGGTCTTAGGAACCAAAA-ACTTGGTGCAACTCCAAATAAAAGTA
Mesoplodon europaeus trnL1                ACTTTTAAAGGATGGAAGT-TATCCATTGGTCTTAGGAACCAAAA-ACTTGGTGCAACTCCAAATAAAAGTA
**Mesoplodon grayi trnL1                  ACTTTTAAAGGATGGAAGT-TATCCATTGGTCTTAGGAACCAAAA-ACTTGGTGCAACTCCAAATAAAAGTA
Berardius bairdii trnL1                   ACTTTTAAAGGATAGAAGT-CATCCGTTGGTCTTAGGAACCAAAA-GCTTGGTGCGACTCCAAATGAGAGTG
Hyperoodon ampullatus trnL1               ACTTTTAAAGGATAGAAGT-TATCCATTGGTCTTAGGAACCAAAA-AATTGGTGCAACTCCAAATAAAAGTA
Lipotes vexillifer trnL1                  ACTTTTAAAGGATAACAGC-TATCCATTGGTCTTAGGAACCAAAA-AATTGGTGCAACTCCAAATAAAAGTA
Inia geoffrensis trnL1                    GCTTTTAAAGGATAGTAGT-TATCCATTGGCCTTAGGAGCCAAAA-AATTGGTGCAACTCCAAATAAAAGCA
Pontoporia blainvillei trnL1              ACTTTTAAAGGATAGTAGT-AATCCATTGGTCTTAGGAACCAAAA-AATTGGTGCAACTCCAAATAAAAGTA
Monodon monoceros trnL1                   ACTTTTAAAGGATGGTAGC-TATCCATTGGTCTTAGGAACCAAAA-AATTGGTGCAACTCCAAATAAAAGTA
**Neophocaena asiaeorientalis trnL1       ACTTTTAAAGGATAGCAGT-TATCCGTTGGTCTTAGGAACCAAAA-ACTTGGTGCAACTCCAAATAAAAGTA
Neophocaena phocaenoides trnL1            ACTTTTAAAGGATAGCAGT-TATCCGTTGGTCTTAGGAACCAAAA-ACTTGGTGCAACTCCAAATAAAAGTA
Phocoena phocoena trnL1                   ACTTTTAAAGGATGGTAGT-TATCCGTTGGTCTTAGGAACCAAAA-ACTTGGTGCAACTCCAAATAAAAGTA
Cephalorhynchus heavisidii trnL1          ACTTTTAAAGGATGGTAGT-TATCCATTGGTCTTAGGAACCAAAA-AATTGGTGCAACTCCAAATAAAAGTA
Sousa chinensis trnL1                     GCTTTTAAAGGATAGTAGT-TATCCATTGGTCTTAGGAACCAAAA-AATTGGTGCAACTCCAAATAAAAGCA
Stenella attenuata trnL1                  GCTTTTAAAGGATAGTAGT-TATCCATTGGTCTTAGGAACCAAAA-AATTGGTGCAACTCCAAATAAAAGTA
Tursiops australis trnL1                  GCTTTTAAAGGATAGTAGT-TATCCATTGGTCTTAGGAACCAAAA-AATTGGTGCAACTCCAAATAAAAGTA
Tursiops truncatus trnL1                  ACTTTTAAAGGATAGTAGT-TATCCATTGGTCTTAGGAACCAAAA-AATTGGTGCAACTCCAAATAAAAGTA
Tursiops aduncus trnL1                    GCTTTTAAAGGATAGTAGT-TATCCATTGGTCTTAGGAACCAAAA-AATTGGTGCAACTCCAAATAAAAGTA
Delphinus capensis trnL1                  ACTTTTAAAGGATAGTAGT-TATCCATTGGTCTTAGGAACCAAAA-AATTGGTGCAACTCCAAATAAAAGTA
Stenella coeruleoalba trnL1               GCTTTTAAAGGATAGTAGT-TATCCATTGGTCTTAGGAACCAAAA-AATTGGTGCAACTCCAAATAAAAGTA
Orcaella brevirostris trnL1               GCTTTTAAAGGATGGTAGT-TATCCATTGGTCTTAGGAACCAAAA-AATTGGTGCAACTCCAAATAAAAGTA
Orcaella heinsohni trnL1                  GCTTTTAAAGGATAGTAGT-TATCCATTGGTCTTAGGAACCAAAA-AATTGGTGCAACTCCAAATAAAAGTA
Grampus griseus trnL1                     GCTTTTAAAGGATAGTAGT-TATCCATTGGTCTTAGGAACCAAAA-AATTGGTGCAACTCCAAATAAAAGTA
Pseudorca crassidens trnL1                GCTTTTAAAGGATAGTAGT-TATCCATTGGTCTTAGGAACCAAAA-AATTGGTGCAACTCCAAATAAAAGCA
Feresa attenuata trnL1                    GCTTTTAAAGGATAGTAGT-TATCCATTGGTCTTAGGAACCAAAA-AATTGGTGCAACTCCAAATAAAAGTA
Peponocephala electra trnL1               GCTTTTAAAGGATAGCAGT-TATCCATTGGTCTTAGGAACCAAAA-AATTGGTGCAACTCCAAATAAAAGTA
Globicephala macrorhynchus trnL1          ACTTTTAAAGGATGGCAGT-TATCCATTGGTCTTAGGAACCAAAA-AATTGGTGCAACTCCAAATAAAAGTA
Globicephala melas trnL1                  ACTTTTAAAGGATGGTAGT-TATCCATTGGTCTTAGGAACCAAAA-AATTGGTGCAACTCCAAATAAAAGTA
Lagenorhynchus albirostris trnL1          ACTTTTAAAGGATAGTAGT-TATCCATTGGTCTTAGGAACCAAAA-AATTGGTGCAACTCCAAATAAAAGTA
Orcinus orca WNPTRU1 trnL1                GCTTTTAAAGGATAGTAGT-TATCCACTGGTCTTAGGAACCAAAA-AATTGGTGCAACTCCAAGTAAAAGTA
Orcinus orca AntA1 trnL1                  ACTTTTAAAGGATAGTAGT-TATCCACTGGTCTTAGGAACCAAAA-AATTGGTGCAACTCCAAGTAAAAGTA
Orcinus orca AntB1 trnL1                  ACTTTTAAAGGATAGTAGT-TATCCACTGGTCTTAGGAACCAAAA-AATTGGTGCAACTCCAAGTAAAAGTA
Orcinus orca AntC1 trnL1                  ACTTTTAAAGGATAGTAGT-TATCCACTGGTCTTAGGAACCAAAA-AATTGGTGCAACTCCAAGTAAAAGTA
Orcinus orca ENAHN1 trnL1                 ACTTTTAAAGGATAGTAGT-TATCCACTGGTCTTAGGAACCAAAA-AATTGGTGCAACTCCAAGTAAAAGTA
Orcinus orca CNPNRAL trnL1                ACTTTTAAAGGATAGTAGT-TATCCACTGGTCTTAGGAACCAAAA-AATTGGTGCAACTCCAAGTAAAAGTA
Orcinus orca ENPOAL2 trnL1                ACTTTTAAAGGATAGTAGT-TATCCACTGGTCTTAGGAACCAAAA-AATTGGTGCAACTCCAAGTAAAAGTA
                                          1234567 1234         4321 12345 ant 54321     1234        43217654321d

                                          0000000 1111         2222 22233 333 34444     4555        66666666777
                                          1234567 0123         2345 78901 456 90123     9012        12345678901 2
```

N, the most common base for the position.

N, half compensatory base change in the stem pair (e.g. T – G vs C – G; A-T vs G-T).

N, N, half compensatory base change in the stem pair exhibiting a mismatch (e.g. T–A vs A–A). Different colours are used to better differentiate the changes.

N, fully compensatory base change in the stem pair exhibiting a mismatch (e.g. C–G vs T–T).

N, type I fully compensatory base change in the stem pair (i.e. purine – pyrimidine vs purine – pyrimidine, e.g. G – C vs A – T).

N, N, type II fully compensatory base change in the stem pair (i.e. purine – pyrimidine vs pyrimidine – purine, e.g. A – T vs T – A). Different colours are used to better differentiate the changes.

N, a mismatch in the in the stem pair; N, substitution pattern not modelled; *, pair in the stem in which a mismatch is prominent. N, N, molecular signature for a taxon.

X, position 1-7 in the acceptor stem; X position 1-4 in the DHU stem; X position 1-5 in the anticodon stem; X position 1-4 in the TΨC stem; ant, anticodon; d, discriminator nucleotide.

trnL2 [LEU (L2, UUR)] multiple alignment

	10 20 30 40 50 60 70
	1234567 1234 4321 12345 ant 54321 12345 543217654321d
Ceratotherium simum **trnL2**	GTTAGGATGGCAGAGC-CGGAAATTGCATAAAACTTAAACCTTTAT-AATCAGAGGTTCAACTCCTCTTCCTAACA
Equus caballus **trnL2**	GTTAGGGTGGCAGAGCCCGGAAATTGCATAAAACTTAAACCTTTAC-ACTCAGAGGTTCAACTCCTCTCCCTAACA
Pecari tajacu **trnL2**	ATTAGGGTGGCAGAGACCGGTTATTGCATAAAACTTAAACTTTTAC-AAACAGAGGTTCAACCCCTCTCCCTAATA
Phacochoerus africanus **trnL2**	GTTAGGGTGGCAGAGACCGGTAATTGCGTAAAACTTAAACCTTTAT-TACCAGAGGTTCAACTCCTCTCCCTAACA
Potamochoerus porcus **trnL2**	ATTAGGGTGGCAGAGACCGGTAATTGCGTAAAACTTAAACCTTTAT-TACCAGAGGTTCAACTCCTCTCCCTAATA
Sus scrofa **trnL2**	ATTAGGGTGGCAGAGACCGGTAATTGCGTAAAACTTAAACCTTTAT-TACCAGAGGTTCAACTCCTCTCCCTAATA
Camelus bactrianus **trnL2**	ATTAGGGTGGCAGAGACCGGTAATTGCATAAAACTTAAGATTTTAG-ACCCAGAGGTTCAACCCCTCTCCCTAATA
Camelus dromedarius **trnL2**	ATTAGGGTGGCAGAGACCGGTAATTGCATAAAACTTAAGATTTTAG-ACCCAGAGGTTCAATTCCTCTCCCTAATA
Lama guanicoe **trnL2**	GTTAGGGTGGCAGAGACCGGTAATTGCATAAAACTTAAGATTTTAC-ACCCAGAGGTTCAATTCCTCTCCCTAACA
Vicugna pacos **trnL2**	GTTAGGGTGGCAGAGACCGGTAATTGCATAAAACTTAAGATTTTAC-ACCCAGAGGTTCAATTCCCCTCCCTAACA
Hyemoschus aquaticus **trnL2**	GTTAAGGTGGCAGAGCCCGGCAATTGCATAAAACTTAAAACTTTAC-AATCAGAGGTTCAAATCCTCTCCCTAACA
Tragulus kanchil **trnL2**	GTTAAGGTGGCAGAGCCCGGTAATTGCATAAGACTTAAAACTTTAT-AACCAGAGATTCAAATCCTCTCCTTAACA
Giraffa camelopardalis **trnL2**	GTTAAGGTGGCAGAGCCCGGTAATTGCATAAAACTTAAACCTTTAT-AATCAGAGATTCAAATTCTCTCCTTAACA
Okapia johnstoni **trnL2**	GTTAAGGTGGCAGAGCCCGGTAATTGTATAAAGCTTAAACCTTTAT-GACCAGAGGTCCAAATCCTCTCCTTAACA
Antilocapra americana **trnL2**	GTTAAGGTGGCAGAGCCCGGTAATTGCGTAAAACTTAAACCTTTAT-ACTCAGAGATTCAAATCCTCTCCTTAACA
Moschus moschiferus **trnL2**	GTTAAGGTGGCAGAGCCCGGTAATTGCGTAAAACTTAAACCTTTAT-AATCAGAGATTCAAATCCTCTCCTTAACA
Muntiacus muntjak **trnL2**	GTTAAGGTGGCAGAGCCCGGTAATTGCGTAAAACTTAAAACTTTAT-AATCAGAGATTCAAATCCTCTCCTTAACA
Capreolus capreolus **trnL2**	GTTAAGGTGGCAGAGCCCGGTAATTGCGTAAAACTTAAACTTTTAT-ATTCAGAGATTCAAATTCTCTCCTTAACA
Alces alces **trnL2**	GTTAAGGTGGCAGAGCCCGGTAATTGCGTAAAACTTAAACTTTTAT-ATTCAGAGATTCAAATCCTCTCCTTAACA
Cervus elaphus **trnL2**	GTTAAGGTGGCAGAGCCCGGTAATTGCGTAAAACTTAAAACTTTAT-AATCAGAGATTCAAATCCTCTCCTTAACA
Dama dama **trnL2**	GTTAAGGTGGCAGAGCCCGGTAATTGCGTAAAACTTAAAACTTTAT-AATCAGAGATTCAAATCCTCTCCTTAACA
Boselaphus tragocamelus **trnL2**	GTTAAGGTGGCAGAGCCCGGTAATTGCGTAAAACTTAAACTTTTAT-ATCCAGAGATTCAAATCCTCTCCTTAACA
Tragelaphus oryx **trnL2**	GTTAAGGTGGCAGAGCCCGGTAATTGCATAAAACTTAAGCTTTTAT-ACCCAGAGATTCAAATCCTCTCCTTAACA
Bos taurus **trnL2**	GTTAAGGTGGCAGAGCCCGGTAATTGCATAAAACTTAAACTTTTAT-ATCCAGAGATTCAAATCCTCTCCTTAACA
Bubalus bubalis **trnL2**	GTTAAGGTGGCAGAGCCCGGTAATTGCATAAAACTTAAACTTTTAT-ATCCAGAGATTCAAATCCTCTCCTTAACA
Syncerus caffer **trnL2**	GTTAAGGTGGCAGAGCCCGGTAATTGCGTAAAACTTAAACTTTTATTACCAGAGATTCAAATCCTCTCCTTAACA
Neotragus moschatus **trnL2**	GTTAAGGTGGCAGAGCCCGGTAATTGCGTAAAACTTAAACCTTTAT-ATTCAGAGATTCAAATCCTCTCCTTAACA
Alcelaphus buselaphus **trnL2**	GTTAAGGTGGCAGAGCCCGGTAATTGCGTAAAACTTAAACCTTTAT-ATTCAGAGATTCAAATTCTCTCCTTAACA
Oryx gazella **trnL2**	GTTAAGGTGGCAGAGCCCGGTAATTGCGTAAAATTTAAACCTTTAT-ATTCAGAGATTCAAATCCTCTCCTTAACA
Pantholops hodgsonii **trnL2**	GTTAAGGTGGCAGAGCCCGGTAATTGCATAAAACTTAAACCTTTAT-ATCCAGAGGTTCAAATCCTCTCCTTAACA
Ovibos moschatus **trnL2**	GTTAAGGTGGCAGAGCCCGGTAATTGCGTAAAACTTAAACCTTTAT-ACTCAGAGATTCAAATTCTCTCCTTAACA
Capra hircus **trnL2**	GTTAAGGTGGCAGAGCCCGGTAATTGCGTAAAACTTAAACCTTTAT-ACTCAGAGATTCAAATCCTCTCCTTAACA
Ovis aries **trnL2**	GTTAAGGTGGCAGAGCCCGGTAATTGCGTAAAACTTAAACCTTTAT-ACTCAGAGATTCAAATCCTCTCCTTAACA
Cephalophus natalensis **trnL2**	GTTAAGGTGGCAGAGCCCGGTAATTGCGTAAAACTTAAACCTTTAT-ACTCAGAGATTCAAATCCTCTCCTTAACA
Redunca fulvorufula **trnL2**	GTTAAGGTGGCAGAGCCCGGTAATTGCGTAAAACTTAAACCTTTAT-ACTCAGAGATTCAAATCCTCTCCTTAACA
Ourebia ourebi **trnL2**	GTTAAGGTGGCAGAGCCCGGTAATTGCGTAAAACTTAAACCTTTAT-ACTCAGAGATTCAAATCCTCTCCTTAACA
Antilope cervicapra **trnL2**	GTTAAGGTGGCAGAGCCCGGTAATTGCGTAAAACTTAAACCTTTAT-ACTCAGAGATTCAAATCCTCTCCTTAACA
Gazella gazella **trnL2**	GTTAAGGTGGCAGAGCCCGGTAATTGCGTAAAACTTAAACCTTTAT-ATTCAGAGATTCAAATCCTCTCCTTAACA
Procapra gutturosa **trnL2**	GTTAAGGTGGCAGAGCCCGGTAATTGCGTAAAACTTAAACCTTTAT-ACTCAGAGATTCAAATCCTCTCCTTAACA
Raphicerus campestris **trnL2**	GTTAAGGTGGCAGAGCCCGGTAATTGCGTAAAACTTAAACCTTTAT-ACTCAGAGATTCAAATCCTCTCCTTAACA
Hexaprotodon liberiensis **trnL2**	GTTGCGATGGCAGAGCCCGGTAATTGCATAAAACTTAAGCCTTTAC-A-TCAGAGGTTCAAATCCTCTTCACAACA
Hippopotamus amphibius **trnL2**	GTTGCGATGGCAGAGCCCGGTAATTGCATAAAACTTAAACCTTTAC-A-CCAGAGGTTCAAATCCTCTTCACAACA
Eubalaena australis **trnL2**	GTTGAGGTGGCAGAGTTCGGTAATTGCATAAAACTTAAACTTTTAC-ACCCAGAGGTTCAAATCCTCTCCCCAACA
Eubalaena japonica **trnL2**	GTTGAGGTGGCAGAGTTCGGTAATTGCATAAAACTTAAACTTTTAC-ACCCAGAGGTTCAAATCCTCTCCCCAACA
Balaena mysticetus **trnL2**	GTTGAGGTGGCAGAGTTCGGTAATTGCATAAAACTTAAACTTTTAC-ACCCAGAGGTTCAAATCCTCTCCCCAACA
Caperea marginata **trnL2**	GTTGAGGTGGCAGAGTTCGGTAATTGCATAAAACTTAAACCTTTAT-ACCCAGAGGTTCAAATCCTCTCCCCAACA
Eschrichtius robustus **trnL2**	GTTGAGGTGGCAGAGTTCGGTAATTGCATAAAACTTAAACTTTTAC-ACCCAGAGGTTCAAATCCTCTCCCCAACA
Balaenoptera acutorostrata **trnL2**	GTTGAGGTGGCAGAGTTCGGCAATTGCATAAAACTTAAACTTTTAC-ACCCAGAGGTTCAAATCCTCTCCCCAACA
Balaenoptera bonaerensis **trnL2**	GTTGAGGTGGCAGAGTTCGGTAATTGCATAAAACTTAAACTTTTAC-ACCCAGAGGTTCAAATCCTCTCCCCAACA
Balaenoptera physalus **trnL2**	GTTGAGGTGGCAGAGTTCGGTAATTGCATAAAACTTAAACTTTTAC-ACCCAGAGGTTCAAATCCTCTCCCCAACA
Megaptera novaeangliae **trnL2**	GTTGGGGTGGCAGAGTTCGGCAATTGCATAAAACTTAAACTTTTAC-ACCCAGGGGTTCAAATCCTCTCCCCAACA
Balaenoptera musculus **trnL2**	GTTGAGGTGGCAGAGTTCGGTAATTGCATAAAACTTAAACCTTTAC-ACTCAGAGGTTCAAATCCTCTCCCCAACA
Balaenoptera omurai **trnL2**	GTTGAGGTGGCAGAGATTGGTAATTGCATAAAACTTAAACTTTTAC-ACCCAGAGGTTCAAATCCTCTCCCCAACA
Balaenoptera borealis **trnL2**	GTTGAGGTGGCAGAGTTCGGCAATTGCATAAAACTTAAACTTTTAC-ACCCAGAGGTTCAAATCCTCTCCCCAACA
Balaenoptera brydei **trnL2**	GTTGAGGTGGCAGAGTTCGGTAATTGCATAAAACTTAAACTTTTAC-ACCCAGAGGTTCAAATCCTCTCCCCAACA
Balaenoptera edeni **trnL2**	GTTGAGGTGGCAGAGTTCGGTAATTGCATAAAACTTAAACTTTTAC-ACCCAGAGGTTCAAATCCTCTCCCCAACA
Kogia breviceps **trnL2**	GTTGAGGTGGCAGAGTCAGGCAATTGCATAAAACTTAAGCTTTTAT-ACCCAGAGGTTCAAATCCCCTCCCCAACA
Physeter macrocephalus **trnL2**	GTTGTGGTGGCAGAGTCCGGTAATTGCATAAAACTTAAACTTTTAC-ACCCAGAGGTTCAAATCCCCTCCTCAACA
Platanista minor **trnL2**	GTTGAGGTGGCAGAGTCCGGCAATTGTATAAAACTTAAACTTTTAC-ACTCAGAGGTTCAAATCCTCTCCCCAACA
Ziphius cavirostris **trnL2**	GTTGAGGTGGCAGAGTCCGGCAATTGTATAAAACTTAAACTTTTAC-ACTCAGAGGTTCAAATCCTCTCCCCAACA
Mesoplodon europaeus **trnL2**	GTTGAGGTGGCAGAGTCCGGTAATTGTATAAAACTTAAACTTTTAC-TCCCAGAGGTTCAAATCCTCTTCTCAACA
Mesoplodon densirostris **trnL2**	GTTGAGGTGGCAGAATCCGGCAATTGCATAAAACTTAAACTTTTAC-ATCCAGAGGTTCAAATCCTCTCCCCAACA
***Mesoplodon grayi* **trnL2**	GTTGAGATGGCAGAGTCCGGCAATTGCATAAAACTTAAACTTTTAC-ACCCAGAGGTTCAAATCCTCTCCTCAACA
Berardius bairdii **trnL2**	GTTGAGGTGGCAGAGTCCGGCAATTGCATAAAACTTAAACCTTTAC-ACCCAGAGGTTCAAATCCTCTCCTCAACA
Hyperoodon ampullatus **trnL2**	GTTGGGGTGGCAGAGTCTGGCAACTGTATAAAACTTAAACTTTTAC-ACCCAGAGGTTCAAATCCTCTCCTCAACA
Lipotes vexillifer **trnL2**	GTTAAGATGGCAGAGTACGGTAATTGCATAAAACTTAAACTTTTAT-ACCCAGAGGTTCAAATCCTCTCCTTAACA
Inia geoffrensis **trnL2**	GTTGAGGTGGCAGAGCACGGGAATTGCATAAAACTTAAGCTTTTAT-TCTCAGAGGTTCAAATCCTCTCCCCAAGC
Pontoporia blainvillei **trnL2**	GTTGGGGTGGCAGAGTACGGTAATTGCATAAAACTTAAACCTTTAT-ACCCAGAGGTTCAAATCCTCTCCTCAACT
Monodon monoceros **trnL2**	GTTGGGATGGCAGAGTACGGCAATTGCATAAAACTTAAACCTTTAT-ACCCAGAGGTTCAAATCCTCTTCCCAACA
***Neophocaena asiaeorientalis* **trnL2**	GTTGGGGTGGCAGAGTACGGCAATTGCATAAAACTTAAGCCTTTAT-ACCCAGAGGTTCAAATCCTCTCCCCAACA
Neophocaena phocaenoides **trnL2**	GTTGGGGTGGCAGAGTACGGCAATTGCATAAAACTTAAGCCTTTAT-ACCCAGAGGTTCAAATCCTCTCCCCAACA
Phocoena phocoena **trnL2**	GTTGGGGTGGCAGAGTACGGCAATTGCATAAAACTTAAGCCTTTAT-ATCCAGAGGTTCAAATCCTCTCCTCAACA
Cephalorhynchus heavisidii **trnL2**	GTTGAAGTGGCAGAGTACGGCAATTGCATAAAACTTAAGCTTTTAT-ACCCAGAGGTTCAAATCCCCTCTTCAACA
Sousa chinensis **trnL2**	GTTGAAGTGGCAGAGTACGGCAATTGCATAAAACTTAAACTTTTAT-ACCCAGAGGTTCAAATCCCCTCTTCAACA
Stenella attenuata **trnL2**	GTTGAAGTGGCAGAGTACGGCAATTGCATAAAACTTAAGCTTTTAT-ACCCAGAGGTTCAAATCCTCTCTTCAACA
Tursiops australis **trnL2**	GTTGAAGTGGCAGAGTACGGCAATTGCATAAAACTTAAGCTTTTAT-ACCCAGAGGTTCAAATCCCCTCTTCAACA
Tursiops truncatus **trnL2**	GTTGAAGTGGCAGAGTACGGCAATTGCATAAAACTTAAGCTTTTAT-ACCCAGAGATTCAAATCCCCTCTTCAACA
Tursiops aduncus **trnL2**	GTTGAAGTGGCAGAGTACGGCAATTGCATAAAACTTAAGCTTTTAT-ACCCAGAGGTTCAAATCCCCTCTTCAACA
Delphinus capensis **trnL2**	GTTGAAGTGGCAGAGTACGGCAATTGCATAAAACTTAAGCTTTTAT-ACCCAGAGGTTCAAATCCCCTCTTCAACA
Stenella coeruleoalba **trnL2**	GTTGAAGTGGCAGAGTACGGCAATTGCATAAAACTTAAGCTTTTAT-ACCCAGAGGTTCAAATCCCCTCTTCAACA
Orcaella brevirostris **trnL2**	GTTGAAGTGGCAGAGTAAGGCAATTGCATAAAACTTAAGCTTTTAT-ACTCAGAGGTTCAAATCCTCTCTTCAACA
Orcaella heinsohni **trnL2**	GTTGAAGTGGCAGAGTAAGGCAATTGCATAAAACTTAAGCTTTTAT-ACTCAGAGGTTCAAATCCTCTCTTCAACA
Grampus griseus **trnL2**	GTTGAAGTGGCAGAGTACGGCAATTGCATAAAATTTAAGCTTTTAT-ACTCAGAGGTTCAAATCCTCTCTTCAACA
Pseudorca crassidens **trnL2**	GTTGAAGTGGCAGAGTACGGCAATTGCATAAAATTTAAGCTTTTAT-ACTCAGAGGTTCAAATCCTCTCTTCAACA
Feresa attenuata **trnL2**	GTTGAAGTGGCAGAGTGTGGCAATTGCATAAAATTTAAGCTTTTAT-ACTCAGAGGTTCAAGTCCTCTCTTCAACA
Peponocephala electra **trnL2**	GTTGAAGTGGCAGAGTATGGCAATTGCATAAAATTTAAGCTTTTAT-ACTCAGAGGTTCAAATCCTCTCTTCAACA
Globicephala macrorhynchus **trnL2**	GTTGAAGTGGCAGAGTATGGCAATTGCATAAAACTTAAACTTTTAT-ATTCAGAGGTTCAAATCCTCTCTTTAACA
Globicephala melas **trnL2**	GTTGAAGTGGCAGAGTATGGCAATTGCATAAAACTTAAACTTTTAT-ATTCAGAGGTTCAAATCCTCTCTTTAACA
Lagenorhynchus albirostris **trnL2**	GTTGAAGTGGCAAAGTACGGCAATTGCATAAAACTTAAGCTTTTAC-ACTCAGAGGTTCAAATCCTCTCTTCAACA
Orcinus orca WNPTRU1 **trnL2**	GCTGAAGTGGCAGAGTATGGCAATTGCATAAAACTTAAACTTTTAT-ACTCAGAGGTTCAAATCCTCTCTTCAACA
Orcinus orca AntA1 **trnL2**	GCTGAAGTGGCAGAGTATGGCAATTGCATAAAACTTAAACTTTTAT-AATCAGAGGTTCAAATCCTCTCTTCAACA
Orcinus orca AntB1 **trnL2**	GCTGAAGTGGCAGAGTATGGCAATTGCATAAAACTTAAACTTTTAT-AATCAGAGGTTCAAATCCTCTCTTCAACA
Orcinus orca AntC1 **trnL2**	GCTGAAGTGGCAGAGTATGGCAATTGCATAAAACTTAAACTTTTAT-AATCAGAGGTTCAAATCCTCTCTTCAACA
Orcinus orca ENAHN1 **trnL2**	GCTGAAGTGGCAGAGTATGGCAATTGCATAAAACTTAAACTTTTAT-ACTCAGAGGTTCAAATCCTCTCTTCAACA
Orcinus orca CNPNRAL **trnL2**	GCTGAAGTGGCAGAGTATGGCAATTGCATAAAACTTAAACTTTTAT-ACTCAGAGGTTCAAATCCTCTCTTCAACA
Orcinus orca ENPOAL2 **trnL2**	GCTGAAGTGGCAGAGTATGGCAATTGCATAAAACTTAAACTTTTAT-ACTCAGAGGTTCAAATCCTCTCTTCAACA
	1234567 1234 4321 12345 ant 54321 12345 543217654321d
	0000000 1111 2222 23333 333 44444 55555 666667777777 7
	1234567 0123 4567 90123 678 12345 23456 456789012345 6

N, the most common base for the position.

N, half compensatory base change in the stem pair (e.g. T – G vs C – G; A-T vs G-T).

N, N, half compensatory base change in the stem pair exhibiting a mismatch (e.g. T–A vs A–A). Different colours are used to better differentiate the changes.

N, fully compensatory base change in the stem pair exhibiting a mismatch (e.g. C–G vs T–T).

N, type I fully compensatory base change in the stem pair (i.e. purine – pyrimidine vs purine – pyrimidine, e.g. G – C vs A – T).

N, N, type II fully compensatory base change in the stem pair (i.e. purine – pyrimidine vs pyrimidine – purine, e.g. A – T vs T – A). Different colours are used to better differentiate the changes.

N, a mismatch in the in the stem pair; N, substitution pattern not modelled; *, pair in the stem in which a mismatch is prominent. N, N, molecular signature for a taxon.

X, position 1-7 in the acceptor stem; X position 1-4 in the DHU stem; X position 1-5 in the anticodon stem; X position 1-5 in the TΨC stem; **ant**, anticodon; **d**, discriminator nucleotide.

trnM (MET) multiple alignment

```
                                       10        20        30        40        50        60        70
                                        |         |         |         |         |         |         |
                               1234567  1234     4321 12345  ant  54321    12345      543217654321d
                                  *                                          **          **    *
Ceratotherium simum trnM       AGTAAGGTCAGCTAAA-CAAGCTATCGGGCCCATACCCCGAAAATGTTGGATTATACCCTTCCCGTACTA
Equus caballus trnM            AGTAAGGTCAGCTAAA-TAAGCTATCGGGCCCATACCCCGAAAATGTTGGATTACACCCTTCCCGTACTA
Pecari tajacu trnM             AGTAAGGTCAGCTAAATTAAGCTATCGGGCCCATACCCCGAAAATGTTGGTTCATACCCCTCCCGTACTA
Phacochoerus africanus trnM    AGTAAGGTCAGCTAAATTAAGCTATCGGGCCCATACCCCGAAAATGTTGGTTCATATCCTTCCCATACTA
Potamochoerus porcus trnM      AGTAAGGTCAGCTAAATCAAGCTATCGGGCCCATACCCCGAAAATGTTGGTTCATATCCTTCCCGTACTA
Sus scrofa trnM                AGTAAGGTCAGCTAAGCTAAGCTATCGGGCCCATACCCCGAAAATGTTGGTTCATACCCTTCCCATACTA
Camelus bactrianus trnM        AGTAAGGTCAGCTAAA-TAAGCTATCGGGCCCATACCCCGAAAATGTTGGTTTATACCCTTCCCGTACTA
Camelus dromedarius trnM       AGTAAGGTCAGCTAAA-TAAGCTATCGGGCCCATACCCCGAAAATGTTGGTTTATATCCTTCCCGTACTA
Lama guanicoe trnM             AGTAAGGTCAGCTAAA-TAAGCTATCGGGCCCATACCCCGAAAATGTTGGTTTATACCCTTCCCGTACTA
Vicugna pacos trnM             AGTAAGGTCAGCTAAA-TAAGCTATCGGGCCCATACCCCGAAAATGTTGGTTTATACCCTTCCCGTACTA
Hyemoschus aquaticus trnM      AGTAAGGTCAGCTAA-TTAAGCTATCGGGCCCATACCCCGAAAATGTTGGTTCATATCCTTCCCGTACTA
Tragulus kanchil trnM          AGTAAGGTCAGCTAATTTAAGCTATCGGGCCCATACCCCGAAAATGTTGGTTCATATCCTTCCCGTACTA
Giraffa camelopardalis trnM    AGTAAGGTCAGCTAA-TTAAGCTACCGGGCCCATACCCCGGAAATGTTGGTTTATACCCTTCCCGTACTA
Okapia johnstoni trnM          AGTAAGGTCAGCTAA-TTAAGCTACCGGGCCCATACCCCGGAAATGTTGGTTTACATCCTTCCCGTACTA
Antilocapra americana trnM     AGTAAGGTCAGCTAA-TTAAGCTATCGGGCCCATACCCCGAAAATGTTGGTTTATATCCTTCCCGTACTA
Moschus moschiferus trnM       AGTAAGGTCAGCTAA-TTAAGCTATCGGGCCCATACCCCGAAAATGTTGGTTTATATCCTTCCCGTACTA
Muntiacus muntjak trnM         AGTAAGGTCAGCTAA-TTAAGCTATCGGGCCCATACCCCGAAAATGTTGGTTTATATCCTTCCCGTACTA
Capreolus capreolus trnM       AGTAAGGTCAGCTAA-TTAAGCTATCGGGCCCATACCCCGAAAATGTTGGTTTATACCCTTCCCGTACTA
Alces alces trnM               AGTAAGGTCAGCTAA-TTAAGCTATCGGGCCCATACCCCGAAAATGTTGGTTTATATCCTTCCCGTACTA
Cervus elaphus trnM            AGTAAGGTCAGCTAA-TTAAGCTATCGGGCCCATACCCCGAAAATGTTGGTTTATACCCTTCCCGTACTA
Dama dama trnM                 AGTAAGGTCAGCTAA-TTAAGCTATCGGGCCCATACCCCGAAAATGTTGGTTTAAACCCTTCCCGTACTA
Boselaphus tragocamelus trnM   AGTAAGGTCAGCTAA-TTAAGCTATCGGGCCCATACCCCGAAAATGTTGGTTTATACCCTTCCCGTACTA
Tragelaphus oryx trnM          AGTAAGGTCAGCTAA-TTAAGCTATCGGGCCCATACCCCGAAAATGTTGGTCTAAACCCTTCCCATACTA
Bos taurus trnM                AGTAAGGTCAGCTAA-TTAAGCTATCGGGCCCATACCCCGAAAATGTTGGTTTATATCCTTCCCGTACTA
Bubalus bubalis trnM           AGTAAGGTCAGCTAA-TTAAGCTATCGGGCCCATACCCCGGAAATGTTGGTTCACATCCTTCCCGTACTA
Syncerus caffer trnM           AGTAAGGTCAGCTAA-TTAAGCTATCGGGCCCATACCCCGAAAATGTTGGTTTATACCCTTCCCGTACTA
Neotragus moschatus trnM       AGTAAGGTCAGCTAA-TCAAGCTATCGGGCCCATACCCCGAAAATGTTGGTTTATACCCTTCCCGTACTA
Alcelaphus buselaphus trnM     AGTAAGGTCAGCTAA-TTAAGCTATCGGGCCCATACCCCGAAAATGTTGGTTCATATCCTTCCCGTACTA
Oryx gazella trnM              AGTAAGGTCAGCTAA-TTAAGCTATCGGGCCCATACCCCGAAAATGTTGGTTTATATCCTTCCCGTACTA
Pantholops hodgsonii trnM      AGTAAGGTCAGCTAA-TTAAGCTATCGGGCCCATACCCCGAAAATGTTGGTTCATATCCTTCCCGTACTA
Ovibos moschatus trnM          AGTAAGGTCAGCTAA-TTAAGCTATCGGGCCCATACCCCGAAAATGTTGGTTTATATCCTTCCCGTACTA
Capra hircus trnM              AGTAAGGTCAGCTAA-TTAAGCTATCGGGCCCATACCCCGAAAATGTTGGTTTATATCCTTCCCGTACTA
Ovis aries trnM                AGTAAGGTCAGCTAA-TTAAGCTATCGGGCCCATACCCCGAAAATGTTGGTTCATATCCTTCCCGTACTA
Cephalophus natalensis trnM    AGTAAGGTCAGCTAA-TTAAGCTATCGGGCCCATACCCCGAAAATGTTGGTTTATATCCTTCCCGTACTA
Redunca fulvorufula trnM       AGTAAGGTCAGCTAA-TTAAGCTATCGGGCCCATACCCCGAAAATGTTGGTTCACATCCTTCCCGTACTA
Ourebia ourebi trnM            AGTAAGGTCAGCTAA-TTAAGCTACCGGGCCCATACCCCGGAAATGTTGGTTCATACCCTTCCCGTACTA
Antilope cervicapra trnM       AGTAAGGTCAGCTAA-TTAAGCTATCGGGCCCATACCCCGAAAATGTTGGTTTATATCCTTCCCGTACTA
Gazella gazella trnM           AGTAAGGTCAGCTAA-TTAAGCTATCGGGCCCATACCCCGAAAATGTTGGTTTATATCCTTCCCGTACTA
Procapra gutturosa trnM        AGTAAGGTCAGCTAA-TTAAGCTATCGGGCCCATACCCCGAAAATGTTGGTTCATATCCTTCCCGTACTA
Raphicerus campestris trnM     AGTAAGGTCAGCTAA-TTAAGCTATCGGGCCCATACCCCGAAAATGTTGGTTTACACCCTTCCCGTACTA
Hexaprotodon liberiensis trnM  AGTAAGGTCAGCTAAA-CAAGCTATCGGGCCCATACCCCGAAAATGTTGGTTTAAATCCTTCCCGTACTA
Hippopotamus amphibius trnM    AGTAAGGTCAGCTAAA-CAAGCTATCGGGCCCATACCCCGAAAATGTTGGTTTAAACCCCTCCCGTACTA
Eubalaena australis trnM       AGTAAGGTCAGCTAAA-CAAGCTATCGGGCCCATACCCCGAAAATGTTGGTTCATATCCTTCCCATACTA
Eubalaena japonica trnM        AGTAAGGTCAGCTAAA-CAAGCTATCGGGCCCATACCCCGAAAATGTTGGTTCATATCCTTCCCATACTA
Balaena mysticetus trnM        AGTAAGGTCAGCTAAA-TAAGCTATCGGGCCCATACCCCGAAAATGTTGGTTCATATCCTTCCCATACTA
Caperea marginata trnM         AGTAAGGTCAGCTAAA-TAAGCTATTGGGCCCATACCCCGAAAATGTTGGTTCATATCCTTCCCATACTA
Eschrichtius robustus trnM     AGTAAGGTCAGCTAAA-TAAGCTATCGGGCCCATACCCCGAAAATGTTGGTTTACATCCTTCCCATACTA
Balaenoptera acutorostrata trnM AGTAAGGTCAGCTAAA-CAAGCTATCGGGCCCATACCCCGAAAATGTTGGTTTATATCCTTCCCATACTA
Balaenoptera bonaerensis trnM  AGTAAGGTCAGCTAAA-CAAGCTATCGGGCCCATACCCCGAAAATGTTGGTCTATATCCTTCCCATACTA
Balaenoptera physalus trnM     AGTAAGGTCAGCTAAA-CAAGCTATCGGGCCCATACCCCGAAAATGTTGGTTCATATCCTTCCCATACTA
Megaptera novaeangliae trnM    AGTAAGGTCAGCTAAA-TAAGCTATCGGGCCCATACCCCGAAAATGTTGGTTTACATCCTTCCCATACTA
Balaenoptera musculus trnM     AGTAAGGTCAGCTAAA-CAAGCTATCGGGCCCATACCCCGAAAATGTTGGTTTATATCCTTCCCATACTA
Balaenoptera omurai trnM       AGTAAGGTCAGCTAAA-CAAGCTATCGGGCCCATACCCCGAAAATGTTGGTTTATATCCCTCCCATACTA
Balaenoptera borealis trnM     AGTAAGGTCAGCTAAA-CAAGCTATCGGGCCCATACCCCGAAAATGTTGGTTTATATCCTTCCCATACTA
Balaenoptera brydei trnM       AGTAAGGTCAGCTAAA-CAAGCTATCGGGCCCATACCCCGAAAATGTTGGTTTATATCCTTCCCATACTA
Balaenoptera edeni trnM        AGTAAGGTCAGCTAAA-CAAGCTATCGGGCCCATACCCCGAAAATGTTGGTTTATACCCTTCCCATACTA
Kogia breviceps trnM           AGTAAGGTCAGCTAAA-CAAGCTATCGGGCCCATACCCCGAAAATGTTGGTTTATACCCTTCCCATACTA
Physeter macrocephalus trnM    AGTAAGGTCAGCTAAA-CAAGCTATCGGGCCCATACCCCGAAAATGTTGGTTTATACCCTTCCCATACTA
Platanista minor trnM          AGTAAGGTCAGCTAAA-CAAGCTATCGGGCCCATACCCCGAAAATGTTGGTTCACACCCTTCCCGTACTA
Ziphius cavirostris trnM       AGTAAGGTCAGCTAAA-TAAGCTATCGGGCCCATACCCCGAAAATGTTGGTTTATATCCTTCCCATACTA
Mesoplodon densirostris trnM   AGTAAGGTCAGCTAAA-TAAGCTATCGGGCCCATACCCCGAAAATGTTGGTTTATATCCTTCCCATACTA
Mesoplodon europaeus trnM      AGTAAGGTCAGCTAAA-TAAGCTATCGGGCCCATACCCCGAAAATGTTGGTTTATACCCTTCCCATACTA
**Mesoplodon grayi trnM        AGTAAGGTCAGCTAAA-TAAGCTATCGGGCCCATACCCCGAAAATGTTGGTTCATATCCTTCCCATACTA
Berardius bairdii trnM         AGTAAGGTCAGCTAAG-TAAGCTATCGGGCCCATACCCCGAAAATGTTGGTTTATATCCTTCCCATACTA
Hyperoodon ampullatus trnM     AGTAAGGTCAGCTAAA-TAAGCTATCGGGCCCATACCCCGAAAATGTTGGTTTATACCCTTCCCATACTA
Lipotes vexillifer trnM        AGTAAGGTCAGCTAAA-TAAGCTATTGGGCCCATACCCCAAAAATGTTGGTTTATACCCTTCCCATACTA
Inia geoffrensis trnM          AGTAAGGTCAGCTAAA-TAAGCTATCGGGCCCATACCCCGAAAATGTTGGTTTATATCCCTCCCGTACTA
Pontoporia blainvillei trnM    AGTAAGGTCAGCTAAA-TAAGCTATCGGGCCCATACCCCGAAAATGTTGGTTCACATCCTTCCCGTACTA
Monodon monoceros trnM         AGTAAGGGCAGCTAAG-TAAGCGATCGGGCCCATACCCCGAAAATGTTGGTTTATACCCTTCCCATACTA
**Neophocaena asiaeorientalis trnM AGTAAGGTCAGCTAAT-TAAGCTATCGGGCCCATACCCCGAAAATGTTGGTTCATACCCTTCCCATACTA
Neophocaena phocaenoides trnM  AGTAAGGTCAGCTAAT-TAAGCTATCGGGCCCATACCCCGAAAATGTTGGTTCATACCCTTCCCATACTA
Phocoena phocoena trnM         AGTAAGGTCAGCTAAT-TAAGCTATCGGGCCCATACCCCGAAAATGTTGGTTCATACCCTTCCCATACTA
Cephalorhynchus heavisidii trnM AGTAAGGTCAGCTAAA-TAAGCTACCGGGCCCATACCCCGGAAATGTTGGTTCATACCCTTCCCATACTA
Sousa chinensis trnM           AGTAAGGTCAGCTAAA-TAAGCTACCGGGCCCATACCCCGGAAATGTTGGTTTACACCCTTCCCATACTA
Stenella attenuata trnM        AGTAAGGTCAGCTAAA-TAAGCTACCGGGCCCATACCCCGGAAATGTTGGTTTATATCCTTCCCATACTA
Tursiops australis trnM        AGTAAGGTCAGCTAAA-TAAGCTACCGGGCCCATACCCCGGAAATGTTGGTTTATATCCTTCCCATACTA
Tursiops truncatus trnM        AGTAAGGTCAGCTAAA-TAAGCTACCGGGCCCATACCCCGGAAATGTTGGTTTATATCCTTCCCATACTA
Tursiops aduncus trnM          AGTAAGGTCAGCTAAA-TAAGCTACCGGGCCCATACCCCGGAAATGTTGGTTTATATCCTTCCCATACTA
Delphinus capensis trnM        AGTAAGGTCAGCTAAA-TAAGCTACCGGGCCCATACCCCGGAAATGTTGGTTTATACCCTTCCCATACTA
Stenella coeruleoalba trnM     AGTAAGGTCAGCTAAA-TAAGCTACCGGGCCCATACCCCGGAAATGTTGGTTTATATCCTTCCCATACTA
Orcaella brevirostris trnM     AGTAAGGTCAGCTAAA-TAAGCTATCGGGCCCATACCCCGAAAATGTTGGTTTATACCCTTCCCATACTA
Orcaella heinsohni trnM        AGTAAGGTCAGCTAAA-TAAGCTACCGGGCCCATACCCCGGAAATGTTGGTTCATACCCTTCCCATACTA
Grampus griseus trnM           AGTAAGGTCAGCTAAA-TAAGCTACCGGGCCCATACCCCGGAAATGTTGGTTCATATCCTTCCCATACTA
Pseudorca crassidens trnM      AGTAAGGTCAGCTAAA-CAAGCTACCGGGCCCATACCCCGGAAATGTTGGTTCATATCCTTCCCATACTA
Feresa attenuata trnM          AGTAAGGTCAGCTAAA-TAAGCTACCGGGCCCATACCCCGGAAATGTTGGTTCATATCCTTCCCATACTA
Peponocephala electra trnM     AGTAAGGTCAGCTAAA-TAAGCTACCGGGCCCATACCCCGGAAATGTTGGTTCATATCCTTCCCATACTA
Globicephala macrorhynchus trnM AGTAAGGTCAGCTAAA-TAAGCTACCGGGCCCATACCCCGGAAATGTCGGTTCATATCCTTCCCATACTA
Globicephala melas trnM        AGTAAGGTCAGCTAAA-TAAGCTACCGGGCCCATACCCCGGAAATGTTGGTTCATATCCTTCCCATACTA
Lagenorhynchus albirostris trnM AGTAAGGTCAGCTAAA-TAAGCTATCGGGCCCATACCCCGGAAATGTTGGTTCATATCCTTCCCATACTA
Orcinus orca WNPTRU1 trnM      AGTAAGGTCAGCTAAA-TAAGCTACCGGGCCCATACCCCGGAAATGTTGGTTCACACCCCTCCCATACTA
Orcinus orca AntA1 trnM        AGTAAGGTCAGCTAAA-TAAGCTACCGGGCCCATACCCCGGAAATGTTGGTTCACACCCCTCCCATACTA
Orcinus orca AntB1 trnM        AGTAAGGTCAGCTAAA-TAAGCTACCGGGCCCATACCCCGGAAATGTTGGTTCACACCCCTCCCATACTA
Orcinus orca AntC1 trnM        AGTAAGGTCAGCTAAA-TAAGCTACCGGGCCCATACCCCGGAAATGTTGGTTCACACCCCTCCCATACTA
Orcinus orca ENAHN1 trnM       AGTAAGGTCAGCTAAA-TAAGCTACCGGGCCCATACCCCGGAAATGTTGGTTCACACCCCTCCCATACTA
Orcinus orca CNPNRAL trnM      AGTAAGGTCAGCTAAA-TAAGCTACCGGGCCCATACCCCGGAAATGTTGGTTCACACCCCTCCCATACTA
Orcinus orca ENPOAL2 trnM      AGTAAGGTCAGCTAAA-TAAGCTACCGGGCCCATACCCCGGAAATGTTGGTTCACACCCCTCCCATACTA
                                  *                                          **          **    *
                               1234567  1234     4321 12345  ant  54321    12345      543217654321d
                               0000000  1111     2222 22222  333  33344    44445      556666666667
                               1234567  0123     0123 56789  234  78901    67890      890123456789 0
```

N, the most common base for the position.

N, half compensatory base change in the stem pair (e.g. T – G vs C – G; A-T vs G-T).

N, N, half compensatory base change in the stem pair exhibiting a mismatch (e.g. T–A vs A–A). Different colours are used to better differentiate the changes.

N, fully compensatory base change in the stem pair exhibiting a mismatch (e.g. C–G vs T–T).

N, type I fully compensatory base change in the stem pair (i.e. purine – pyrimidine vs purine – pyrimidine, e.g. G – C vs A – T).

N, N, type II fully compensatory base change in the stem pair (i.e. purine – pyrimidine vs pyrimidine – purine, e.g. A – T vs T – A). Different colours are used to better differentiate the changes.

N, a mismatch in the in the stem pair; N, substitution pattern not modelled; *, pair in the stem in which a mismatch is prominent. N, N, molecular signature for a taxon.

X, position 1-7 in the acceptor stem; X position 1-4 in the DHU stem; X position 1-5 in the anticodon stem; X position 1-5 in the TΨC stem; ant, anticodon; d, discriminator nucleotide.

trnN (ASN) multiple alignment

```
                                       10        20        30        40        50        60        70
                                        |         |         |         |         |         |         |
                                 1234567 123         321 12345  ant  54321       12345        543217654321d
                                                                                   *             *
Ceratotherium simum trnN         TAGATTGAAGCCAGTTGATTAGGGTATTTAGCTGTTAACTAAAA-TTTCGTGGGATA--GTGGCCCACCAATCTAG
Equus caballus trnN              TAGATTGAAGCCAGTTGATTAGGGTATTTAGCTGTTAACTAAAA-TTTCGTGGGATG--GTAGCCCACCAATCTAG
Pecari tajacu trnN               TAGATTGAAGCCAGTTGACTAGGGTATTTAGCTGTTAACTAAAAGTTTCGTGGGTTG--TAGTCCACCAGTCTAG
Phacochoerus africanus trnN      TAGATTGAAGCCAGTTGATTAGGGTATTTAGCTGTTAACTAAAAGTTTCGTGGGTAT-GTAATCCCGCCAATCTAG
Potamochoerus porcus trnN        TAGATTGAAGCCAGTTGATTAGGGTATTTAGCTGTTAACTAAAAGTTTCGTGGGTGT-GTAATCCCGCCAATCTAG
Sus scrofa trnN                  TAGATTGAAGCCAGTTGATTAGGGGTTTAGCTGTTAACTAAAAGTTTCGTGGGTAT-GTAATCCCACCAATCTAG
Camelus bactrianus trnN          TAGATTGAAGCCAGTTGACTAGGGTATTTAGCTGTTAACTAAAA-TTTCGTGGGATA--AAGGCCCATCAATCTAG
Camelus dromedarius trnN         TAGATTGAAGCCAGTTGACTAGGGCACTTAGCTGTTAACTAAAA-TTTCGTGGGATA--AAGGCCCGCCAATCTAG
Lama guanicoe trnN               TAGATTGAAGCCAGTTGACTAGGGTATTTAGCTGTTAACTAAAA-TTTCGTGGGATA--CAGGCCCACCAATCTAG
Vicugna pacos trnN               TAGATTGAAGCCAGTTGACTAGGGTATTTAGCTGTTAACTAAAA-TTTCGTGGGATA--CAGGCCCACCAATCTAG
Hyemoschus aquaticus trnN        TAGATTGAAGCCAGTTGATTAAGGTGTTTAGCTGTTAACTAAAGG-TTTGTAGGATA--AAAGCCTACCAGTCTAG
Tragulus kanchil trnN            TAGATTGAAGCCAGCTGATTAGGGTACTTAGCTGTTAACTAAG-GTTTTGCAGGATA--GAGGCCTGTCAATCTAG
Giraffa camelopardalis trnN      TAGATTGAAGCCAGTTGATTAGGGTATTTAGCTGTTAACTAAA-GTTTCGTGGGG-G-TGGAGCCCACCAATCTAG
Okapia johnstoni trnN            TAGATTGAAGCCAGTTGATTAGGGGTTTAGCTGTTAACTAAA-GTTTCGTGGGG-G-TGGATCCCACCAATCTAG
Antilocapra americana trnN       TAGATTGAAGCCAGTTGATTAGGGTATTTAGCTGTTAACTAAA-GTTTCGTGGGG-G-CGGAGCCCACCAATCTAG
Moschus moschiferus trnN         TAGATTGAAGCCAGTTGATTAGGGGTTTAGCTGTTAACTAAA-GTCTCGTGGGG-G-TGGATCCCACCAATCTAG
Muntiacus muntjak trnN           TAGATTGAAGCCAGTTGATTAGGGTATTTAGCTGTTAACTAAA-GTTTCGTGGGG-G-TGGAGCCCACCAATCTAG
Capreolus capreolus trnN         TAGATTGAAGCCAGTTGACTAGGGGTTTAGCTGTTAACTAAA-GTTTCGTGGGG-G-TGGAGCCCACCAATCTAG
Alces alces trnN                 TAGATTGAAGCCAGTTGATTAGGGGTTTAGCTGTTAACTAAA-GTTTCGTGGGG-G-TGGAGCCCACCAATCTAG
Cervus elaphus trnN              TAGATTGAAGCCAGTTGATTAGGGGTTTAGCTGTTAACTAAA-GTTTCGTGGGG-G-TGGAGCCCACCAATCTAG
Dama dama trnN                   TAGATTGAAGCCAGTTGATTAGGGGTTTAGCTGTTAACTAAA-GTTTCGTGGGG-G-TGGAGCCCACCAATCTAG
Boselaphus tragocamelus trnN     TAGATTGAAGCCAGTTGATTAGGGTATTTAGCTGTTAACTAAA-GTTTCGTGGGG-G-TGGATCCCACCAATCTAG
Tragelaphus oryx trnN            TAGATTGAAGCCAGTTATGTAGGGGTTTAGCTGTTAACTAAA-GTTTCGTGGGG-G-TGGAGCCCACCAATCTAG
Bos taurus trnN                  TAGATTGAAGCCAGTTAGCTAGGGGTTTAGCTGTTAACTAAA-GTTTCGTGGGG-G-TGGAGCCCACCAGTCTAG
Bubalus bubalis trnN             TAGATTGAAGCCAGTTGATTAGGGCATTTAGCTGTTAACTAAA-GTTTCGTGGGG-G-TGGAGCCCACCAGTCTAG
Syncerus caffer trnN             TAGATTGAAGCCAGTTGATTAGGGCGTTTAGCTGTTAACTAAA-GTTTCGTGGGG-G-TGGAGCCCACCAGTCTAG
Neotragus moschatus trnN         TAGATTGAAGCCAGTTGTTTAGGGTATTTAGCTGTTAACTAAAA-CTTCGTGGGG-G-AGGATCCCACCAATCTAG
Alcelaphus buselaphus trnN       TAGATTGAAGCCAGTTGTTTAGGGGTTTAGCTGTTAACTAAA-GTTTCGTGGGG-G-TGGAGCCCACCAATCTAG
Oryx gazella trnN                TAGATTGAAGCCAGTTGTTTAGGGGTTTAGCTGTTAACTAAA-GTTTCGTGGGG-G-TGGAGCCCACCAATCTAG
Pantholops hodgsonii trnN        TAGATTGAAGCCAGTTGTCTAGGGTATTTAGCTGTTAACTAAA-GTTTCGTGGGG-G-TGGAGCCCACCAATCTAG
Ovibos moschatus trnN            TAGATTGAAGCCAGTTGTCTAGGGGTTTAGCTGTTAACTAAA-GTTTCGTGGGG-G-TGGAGCCCACCAATCTAG
Capra hircus trnN                TAGATTGAAGCCAGTTATCTAGGGTATTTAGCTGTTAACTAAA-GTTTCGTGGGG-G-TGGAGCCCACCAGTCTAG
Ovis aries trnN                  TAGATTGAAGCCAGTTGTTTAGGGGTTTAGCTGTTAACTAAA-GTTTCGTGGGG-G-TGGAGCCCACCAATCTAG
Cephalophus natalensis trnN      TAGATTGAAGCCAGTTGTTTAGGGTATTTAGCTGTTAACTAAAT-TTTCGTGGGG-G-TGGAGCCCACCAATCTAG
Redunca fulvorufula trnN         TAGATTGAAGCCAGTTGTTTAGGGTATTTAGCTGTTAACTAAAA-TCTCGTGGGG-G-TGGAGCCCACCAATCTAG
Ourebia ourebi trnN              TAGATTGAAGCCAGTATTTTAGGGGTTTAGCTGTTAACTAAA-GTTTCGTGGGG-G-TGGAGCCCACCAATCTAG
Antilope cervicapra trnN         TAGATTGAAGCCAGTATGTTAGGGTATTTAGCTGTTAACTAAAA-CTTCGTGGGG-G-TAGAGCCCACCAATCTAG
Gazella gazella trnN             TAGATTGAAGCCAGTATATTAGGGTATTTAGCTGTTAACTAAAA-CTTCGTGGGG-G-TGGAGCCCACCAATCTAG
Procapra gutturosa trnN          TAGATTGAAGCCAGTATTTTAGGGTATTTAGCTGTTAACTAAAT-TTTCGTGGGG-G-TGGAGCCCACCAATCTAG
Raphicerus campestris trnN       TAGATTGAAGCCAGTACGTTAGGGTTTTAGCTGTTAACTAAA-GTTTCGTGGGG-G-CGGAGCCCACCAGTCTAG
Hexaprotodon liberiensis trnN    TAGATTGAAGCCAGTTGATTAGGGTATTTAGCTGTTAACTAAAAG-TTCGTGGGGAG-CATGTCCCACCAATCTAG
Hippopotamus amphibius trnN      TAGATTGAAGCCAGTTGATTAGGGTATTTAGCTGTTAACTAAAAG-TTCGTGGGGAG-CATGCCCCACCAATCTAG
Eubalaena australis trnN         TAGATTGAAGCCAGTTGATTAGGGTATTTAGCTGTTAACTAAAAG-TTCGTGGGA-GATGTATCCCTCCAATCTAG
Eubalaena japonica trnN          TAGATTGAAGCCAGTTGATTAGGGTATTTAGCTGTTAACTAAAAG-TTCGTGGGA-GATGTATCCCTCCAATCTAG
Balaena mysticetus trnN          TAGATTGAAGCCAGTTGACTAGGGTATTTAGCTGTTAACTAAAAG-TTCGTGGGA-GATGTATCCCTCCAATCTAG
Caperea marginata trnN           TAGATTGAAGCCAGTTGACTAGGGTATTTAGCTGTTAACTAAAAG-TTCGTGGGA-GATGTATCCCTCCAATCTAG
Eschrichtius robustus trnN       TAGATTGAAGCCAGTTGACTAGGGTATTTAGCTGTTAACTAAAAA-TTCGTGGGA-GATGTATCCCTCCAATCTAG
Balaenoptera acutorostrata trnN  TAGATTGAAGCCAGTTGATTAGGGTATTTAGCTGTTAACTAAAAA-TTCGTGGGA-GATGTATCCCTCCAATCTAG
Balaenoptera bonaerensis trnN    TAGATTGAAGCCAGTTGATTAGGGTATTTAGCTGTTAACTAAAAA-TTCGTGGGA-GATGTATCCCTCCAATCTAG
Balaenoptera physalus trnN       TAGATTGAAGCCAGTTGATTAGGGTATTTAGCTGTTAACTAAAAA-TTCGTGGGA-GATGTATCCCTCCAATCTAG
Megaptera novaeangliae trnN      TAGATTGAAGCCAGTTGATTAGGGTATTTAGCTGTTAACTAAAAA-TTCGTGGGA-GATGTATCCCTCCAATCTAG
Balaenoptera musculus trnN       TAGATTGAAGCCAGTTGATTAGGGTATTTAGCTGTTAACTAAAAA-TTCGTGGGA-GATGTATCCCTCCAATCTAG
Balaenoptera omurai trnN         TAGATTGAAGCCAGTTGATTAGGGTATTTAGCTGTTAACTAAAAA-TTCGTGGGA-GATGTATCCCTCCAATCTAG
Balaenoptera borealis trnN       TAGATTGAAGCCAGTTGACTAGGGTATTTAGCTGTTAACTAAAAG-TTCGTGGGAAGATGTATCCCTCCAATCTAG
Balaenoptera brydei trnN         TAGATTGAAGCCAGTTGATTAGGGTATTTAGCTGTTAACTAAAAG-TTCGTGGGAAGATGTATCCCTCCAATCTAG
Balaenoptera edeni trnN          TAGATTGAAGCCAGTTGATTAGGGTATTTAGCTGTTAACTAAAAG-TTCGTGGGAAGATGTATCCCTCCAATCTAG
Kogia breviceps trnN             TAGATTGAAGCCAGTTGACTAGGGTATTTAGCTGTTAACTAAAAG-TTCGAGGGAAG-TATACCCCTCCAATCTAG
Physeter macrocephalus trnN      TAGATTGAAGCCAGTTGACTAGGGTATTTAGCTGTTAACTAAAAG-TTCGTGGGAAG-TGTATCCCTCCAATCTAG
Platanista minor trnN            TAGATTGAAGCCAGTTGTTTAGGGTATTTAGCTGTTAACTAAAAG-TTCGTGGGA-AATGTATCCCTCCAATCTAG
Ziphius cavirostris trnN         TAGATTGAAGCCAGTTGACTAGGGTATTTAGCTGTTAACTAAAAG-TTCGTGGGA-AATGTATCCCTCCAATCTAG
Mesoplodon densirostris trnN     TAGATTGAAGCCAGTTGACTAGGGTATTTAGCTGTTAACTAAAAA-TTCGTGGGA-AATGTATCCCTCCAATCTAG
Mesoplodon europaeus trnN        TAGATTGAAGCCAGTTGATTAGGGTATTTAGCTGTTAACTAAAAA-TTCGTGGGA-AATGTATCCCTCCAATCTAG
**Mesoplodon grayi trnN          TAGATTGAAGCCAGTTGATTAGGGTATTTAGCTGTTAACTAAAAA-TTCGAGGGA-AATGTATCCCTCCAATCTAG
Berardius bairdii trnN           TAGATTGAAGCCAGTTGACTAGGGTATTTAGCTGTTAACTAAAAA-TTCGTGGGA-AATGTATCCCTCCAATCTAG
Hyperoodon ampullatus trnN       TAGATTGAAGCCAGTTGACTAGGGTATTTAGCTGTTAACTAAAAG-TTCGTGGGA-AATGTATCCCTCCAATCTAG
Lipotes vexillifer trnN          TAGATTGAAGCCAGTTGATTAGGGTATTTAGCTGTTAACTAAAAG-TTCGTGGGA-GATGTATCCCTCCAATCTAG
Inia geoffrensis trnN            TAGATTGAAGCCAGTTGATTAGGGTATTTAGCTGTTAACTAAAAG-TTCGTGGGA-GATGTATCCCTCCAATCTAG
Pontoporia blainvillei trnN      TAGATTGAAGCCAGTTGATTAGGGTATTTAGCTGTTAACTAAAAG-TTCGTGGGA-GATGTATCCCTCCAATCTAG
Monodon monoceros trnN           TAGATTGAAGCCAGTTGATTAGGGTATTTAGCTGTTAACTAAAAG-TTCGTGGGA-GATGTATCCCTCCAATCTAG
**Neophocaena asiaeorientalis trnN TAGATTGAAGCCAGTTGATTAGGGTATTTAGCTGTTAACTAAAAG-TTCGTGGGA-GATGTATCCCTCCAATCTAG
Neophocaena phocaenoides trnN    TAGATTGAAGCCAGTTGATTAGGGTATTTAGCTGTTAACTAAAAG-TTCGTGGGA-AGATATCCCTCCAATCTAG
Phocoena phocoena trnN           TAGATTGAAGCCAGTTGACTAGGGTATTTAGCTGTTAACTAAAAG-TTCGTGGGA-GATGTATCCCTCCAATCTAG
Cephalorhynchus heavisidii trnN  TAGATTGAAGCCAGTTTACTAGGGTATTTAGCTGTTAACTAAAAG-TTCGTGGGA-GATGTATCCCTCCAATCTAG
Sousa chinensis trnN             TAGATTGAAGCCAGTTTACTAGGGTATTTAGTTGTTAACTAAAAG-TTCGTGGGA-GATGTATCCCTCCAATCTAG
Stenella attenuata trnN          TAGATTGAAGCCAGTTTACTAGGGTATTTAGCTGTTAACTAAAAG-TTCGTGGGA-GATGTATCCCTCCAATCTAG
Tursiops australis trnN          TAGATTGAAGCCAGTTTACTAGGGTATTTAGCTGTTAACTAAAAG-TTCGTGGGA-GATGTATCCCTCCAATCTAG
Tursiops truncatus trnN          TAGATTGAAGCCAGTTTACTAGGGTATTTAGCTGTTAACTAAAAG-TTCGTGGGA-GATGTATCCCTCCAATCTAG
Tursiops aduncus trnN            TAGATTGAAGCCAGTTTACTAGGGTATTTAGCTGTTAACTAAAAG-TTCGTGGGA-GATGTATCCCTCCAATCTAG
Delphinus capensis trnN          TAGATTGAAGCCAGTTTACTAGGGTATTTAGCTGTTAACTAAAAG-TTCGTGGGA-GATGTATCCCTCCAATCTAG
Stenella coeruleoalba trnN       TAGATTGAAGCCAGTTTACTAGGGTATTTAGCTGTTAACTAAAAG-TTCGTGGGA-GATGTATCCCTCCAATCTAG
Orcaella brevirostris trnN       TAGATTGAAGCCAGTTTATTAGGGTATTTAGCTGTTAACTAAAAG-TTCGTGGGA-GATGTATCCCTCCAATCTAG
Orcaella heinsohni trnN          TAGATTGAAGCCAGTTTACTAGGGTATTTAGCTGTTAACTAAAAG-TTCGTGGGA-GATGTATCCCTCCAATCTAG
Grampus griseus trnN             TAGATTGAAGCCAGTTTACTAGGGTATTTAGCTGTTAACTAAAAG-TTCGTGGGA-GATGTATCCCTCCAATCTAG
Pseudorca crassidens trnN        TAGATTGAAGCCAGTTTATTAGGGTATTTAGCTGTTAACTAAAAG-TTCGTGGGA-GATATATCCCTCCAATCTAG
Feresa attenuata trnN            TAGATTGAAGCCAGTTTATTAGGGTATTTAGCTGTTAACTAAAAG-TTCGTGGGA-GATGTATCCCTCCAATCTAG
Peponocephala electra trnN       TAGATTGAAGCCAGTTTATTAGGGTATTTAGCTGTTAACTAAAAG-TTCGTGGGA-AATGTATCCCTCCAATCTAG
Globicephala macrorhynchus trnN  TAGATTGAAGCCAGTTTATTAGGGTATTTAGCTGTTAACTAAAAG-TTCGTGGGA-AATGTATCCCTCCAATCTAG
Globicephala melas trnN          TAGATTGAAGCCAGTTTATTAGGGTATTTAGCTGTTAACTAAAAG-TTCGTGGGA-AATGTATCCCTCCAATCTAG
Lagenorhynchus albirostris trnN  TAGATTGAAGCCAGTTTACTAGGGTATTTAGCTGTTAACTAAAAG-TTCGTGGGA-GATCTATCCCTCCAATCTAG
Orcinus orca WNPTRU1 trnN        TAGATTGAAGCCAGTTTACTAGGGTATTTAGCTGTTAACTAAAAG-TTCGTGGGA-GATGTATCCCTCCAATCTAG
Orcinus orca AntA1 trnN          TAGATTGAAGCCAGTTTACTAGGGTATTTAGCTGTTAACTAAAAG-TTCGTGGGA-GATGTATCCCTCCAATCTAG
Orcinus orca AntB1 trnN          TAGATTGAAGCCAGTTTACTAGGGTATTTAGCTGTTAACTAAAAG-TTCGTGGGA-GATGTATCCCTCCAATCTAG
Orcinus orca AntC1 trnN          TAGATTGAAGCCAGTTTACTAGGGTATTTAGCTGTTAACTAAAAG-TTCGTGGGA-GATGTATCCCTCCAATCTAG
Orcinus orca ENAHN1 trnN         TAGATTGAAGCCAGTTTACTAGGGTATTTAGCTGTTAACTAAAAG-TTCGTGGGA-GATGTATCCCTCCAATCTAG
Orcinus orca CNPNRAL trnN        TAGATTGAAGCCAGTTTACTAGGGTATTTAGCTGTTAACTAAAAG-TTCGTGGGA-GATGTATCCCTCCAATCTAG
Orcinus orca ENPOAL2 trnN        TAGATTGAAGCCAGTTTACTAGGGTATTTAGCTGTTAACTAAAAG-TTCGTGGGA-GATGTATCCCTCCAATCTAG
                                                                                   *             *
                                 1234567 123         321 12345  ant  54321       12345        543217654321d
                                 0000000 111         222 22233  333  34444       55555        666666777777
                                 1234567 012         345 78901  456  90123       01234        456789012345
```

N, the most common base for the position.
N, half compensatory base change in the stem pair (e.g. T – G vs C – G; A-T vs G-T).
N, N, half compensatory base change in the stem pair exhibiting a mismatch (e.g. T–A vs A–A). Different colours are used to better differentiate the changes.
N, fully compensatory base change in the stem pair exhibiting a mismatch (e.g. C–G vs T–T).
N, type I fully compensatory base change in the stem pair (i.e. purine – pyrimidine vs purine – pyrimidine, e.g. G – C vs A – T).
N, N, type II fully compensatory base change in the stem pair (i.e. purine – pyrimidine vs pyrimidine – purine, e.g. A – T vs T – A). Different colours are used to better differentiate the changes.
N, a mismatch in the in the stem pair; N, substitution pattern not modelled; *, pair in the stem in which a mismatch is prominent. N, N, molecular signature for a taxon.
X, position 1-7 in the acceptor stem; X position 1-3 in the DHU stem; X position 1-5 in the anticodon stem; X position 1-5 in the TΨC stem; ant, anticodon; d, discriminator nucleotide.

trnP (PRO) multiple alignment

Species	Sequence
Position	10 / 20 / 30 / 40 / 50 / 60
Structure	1234567 1234 4321 12345 ant 54321 1234 4321 7654321 d
Ceratotherium simum trnP	CAAGGAGTAGTTTAA-GTAGAATTTCAGCTTTGGGTGTTGATGGTGAGGCTA-GG-GCCTCTTCCTTGA
Equus caballus trnP	CAAGGAATAGTTTAA-GTAGAATTTCAGCTTTGGGTGTTGATGGTGGAGCTA-GA-GCTTCTTCCTTGA
Pecari tajacu trnP	CAGGGAATAGTTTAAT-TAGAATTTCAGCTTTGGGTGCTGATGGTGGAGTCA--G-TCTCCTTCCTTGA
Phacochoerus africanus trnP	CAGGGAATAGTTTAGT-TAGAATTTCAGCTTTGGGTGCTGATGGCGGAGTTG---TTCTCCTTCCTTGA
Potamochoerus porcus trnP	CAGGGAATAGTTTAGT-TAGAATTTCAGCTTTGGGTGCTGATGGCGGAGTTG---TTCTCCTTCCTTGA
Sus scrofa trnP	CAGGGAATAGTTTAGT-TAGAATTTCAGCTTTGGGTGCTGATGGCGGAGTTA--G-TCTCCTTCCTTGA
Camelus bactrianus trnP	CAGGGGGTAGTTTAATG-AGAATTCCAGCTTTGGGTGCTGGTGGTGGGGCTTT-G-GCCTCTTCCCTGA
Camelus dromedarius trnP	CAGGGGGTAGTTTAATA-AGAATTCCAGCTTTGGGTGCTGGTGGTGGGGCTTT-G-GCCTCTCCCCTGA
Lama guanicoe trnP	CAGGGGGTAGTTTAATA-AGAATTCCAGCTTTGGGTGCTGGTGGTAGGGCTTT-G-GCCCTTTCCCTGA
Vicugna pacos trnP	CAGGGGGTAGTTTAATA-AGAATTCCAGCTTTGGGTGCTGGTGGTAGGGCTTT-G-GCTCTTTCCCTGA
Hyemoschus aquaticus trnP	CAGGGGATAGTTTAA-GTAGAATTTCAGCTTTGGGTGCTGATGGTGGAGCGTT-A-GCTTCTTCCTTGA
Tragulus kanchil trnP	CAAGGAATAGTTTAA-CTAGAATTTCAGCTTTGGGTGTTGATGGTGAGGTTTG-AGGCCTCTTCCTTGA
Giraffa camelopardalis trnP	CAGGGAATAGTTTAA-ATAGAACTTCAGCTTTGGGTGTTGATGGTGGGGCAAT-A-GCTCCTTCCTTGA
Okapia johnstoni trnP	CAGAGAATAGTTTAA-ACAGAACTTCAGCTTTGGGTGTTGATGGTGGGGCCGT-G-GCCTCTTCTTTGA
Antilocapra americana trnP	CAGGGAATAGTTTAA-GTAGAACTTCAGCTTTGGGTGTTGAAAGTGGGGCTAT--TGCTTCTTCCTTGA
Moschus moschiferus trnP	CAGGGAATAGTTTAA-ATAGAACTTCAGCTTTGGGTGTTGATAGTGGGGCTAT-A-GCTTCTTCCTTGA
Muntiacus muntjak trnP	CAGGGAATAGTTTAA-ATAGAACTTCAGCTTTGGGTGTTGATGGTGGGGCTAT-G-GCTTCTTCCTTGA
Capreolus capreolus trnP	CAGGGAATAGTTTAAT-TAGAACTTCAGCTTTGGGTGTTGATGGTGGGGCTAT-A-GCTTCTTCCTTGA
Alces alces trnP	CAGGGAATAGTTTAA-ATAGAACTTCAGCTTTGGGTGTTGATAGTGGGGCTAT-A-GCTTCTTCCTTGA
Cervus elaphus trnP	CAGGGAATAGTTTAA-ATAGAACTTCAGCTTTGGGTGTTGATAGTGGGGCTAT-G-GCTTCTTCCTTGA
Dama dama trnP	CAGGGAATAGTTTAA-ATAGAACTTCAGCTTTGGGTGTTGATAGTGGGGTTAT-A-GCTCCTTCCTTGA
Boselaphus tragocamelus trnP	CAGGGAATAGTTTAA-ATAGAACTTCAGCTTTGGGTGTTGATGGTGGAGCTAT-G-GCTTCTTCCTTGA
Tragelaphus oryx trnP	CAGGGAATAGTTTAA-ATAGAACTTCAGCTTTGGGAGTTGATGGTGGGGCGGT--TGCTTCTTCCTTGA
Bos taurus trnP	CAGGGAATAGTTTAA-ATAGAACTTCAGCTTTGGGGGTTGATGGTGAGACTGC-A-GTTTCTTCCTTGA
Bubalus bubalis trnP	CAGGGAGTAGTTTAA-ATAGAACTTCAGCTTTGGGTGTTGGTAGTGGGGCTAT-A-GCCTCTTCCCTGA
Syncerus caffer trnP	CAGGGAGTAGTTTAA-ATAGAACTTCAGCTTTGGGTGTTGGTAGTGGGGCTAT-A-GCCTCTTCCCTGA
Neotragus moschatus trnP	CAGGGAATAGTTTAA-GTAGAACTTCAGCTTTGGGTGTTGATGGTGGAGCTAT-A-GCTTCTTCCTTGA
Alcelaphus buselaphus trnP	CAGGGAATAGTTTAA-ATAGAACTTCAGCTTTGGGTGCTGATAGTGGGGCTATTAGGCTTCTTCCTTGA
Oryx gazella trnP	CAGGGAATAGTTTAA-ATAGAACTTCAGCTTTGGGTGTTGATAGTGGGGCTAT-A-GCTTCTTCCTTGA
Pantholops hodgsonii trnP	CAGGGAATAGTTTAA-ATAGAACTTCAGCTTTGGGTGTTGATAGTGGGGCTAT-A-GCTTCTTCCTTGA
Ovibos moschatus trnP	CAGGGAGTAGTTTAA-ATAGAACTTCAGCTTTGGGTGTTGATAGTGGAGCTAT-A-GCTTCTTCCTTGA
Capra hircus trnP	CAGGGAATAGTTTAA-ATAGAATTTCAGCTTTGGGTGCTGATAGTGAGGCTAT-G-GCTTCTTCCTTGA
Ovis aries trnP	CAGGGAATAGTTTAA-GTAGAACTTCAGCTTTGGGTGTTGATAGTGGGGCTAT-A-GCTTCTTCCTTGA
Cephalophus natalensis trnP	CAGGGAATAGTTTAA-ATAGAACTTCAGCTTTGGGTGTTGATAGTGGGGCTCT-A-GTCTCTTCCTTGA
Redunca fulvorufula trnP	CAGGGAATAGTTTAA-GTAGAACTTCAGCTTTGGGTGTTGATAGTGGGGCTAT-A-GCTTCTTCCTTGA
Ourebia ourebi trnP	CAGGGAATAGTTTAA-ATAGAACTTCAGCTTTGGGTGCTGATGGTGGAGTTAT-A-GCTTCTTCCTTGA
Antilope cervicapra trnP	CAGGGAATAGTTTAA-ATAGAACTTCAGCTTTGGGTGTTGATAGTGGGGCTAT-G-GCTTCTTCCTTGA
Gazella gazella trnP	CAGGGAATAGTTTAA-ATAGAACTTCAGCTTTGGGTGTTGATAGTGGGGCTAT-G-GCTTCTTCCTTGA
Procapra gutturosa trnP	CAGGGAATAGTTTAA-GCAGAACTTCAGCTTTGGGTGTTGATAGTGGAGCCAT-A-GCTTCTTCCTTGA
Raphicerus campestris trnP	CAGGGAATAGTTTAA-ATAGAACTTCAGCTTTGGGTGTTGATAGTGGGGCTAT-A-GTCTCTTCCTTGA
Hexaprotodon liberiensis trnP	CAGGGAATAGTTTA-TCTAGAATTTCAGCTTTGGGTGCTGATGGTGGAGCTT-GG-GCTTCTTCCTTGA
Hippopotamus amphibius trnP	CAGGGAATAGTTTA-TCTAGAATTTCAGCTTTGGGTGCTGATGGTGGAGCTTGAG-CTTTCTTCCTTGA
Eubalaena australis trnP	CAGGGAATAGTTTAA-GTAGAATTTCAGCTTTGGGTGCTGATGGTGGAGTGT-AGTACTTCTTCCTTGA
Eubalaena japonica trnP	CAGGGAATAGTTTAA-GTAGAATTTCAGCTTTGGGTGCTGATGGTGGAGTAT-AGTACTTCTTCCTTGA
Balaena mysticetus trnP	CAGGGAATAGTTTAATGTAGAATTTCAGCTTTGGGTGCTGATGGTGGAGTGT-AGTGCTTCTTCCTTGA
Caperea marginata trnP	CAGGGAATAGTTTA-TGTAGAATTTCAGCTTTGGGTGCTGGCGGTGGAGTAT-AGTACTTCTTCCTTGA
Eschrichtius robustus trnP	CAGGGAATAGTTTA-TGTAGAATCTCAGCTTTGGGTGCTGATGGTGGAGTGT-GATGCTTCTTCCTTGA
Balaenoptera acutorostrata trnP	CAGGGAATAGTTTA-TGTAGAATTTCAGCTTTGGGTGCTGATGGTGGAGTGT-GATGCTTCTTCCTTGA
Balaenoptera bonaerensis trnP	CAGGGAATAGTTTA-TGTAGAATTTCAGCTTTGGGTGCTGATGGTGGAGTGT-GATGCTTCTTCCTTGA
Balaenoptera physalus trnP	CAGGGAATAGTTTA-TGTAGAACTTCAGCTTTGGGTGCTGATGGTGGAGTGT-AATACTTCTTCCTTGA
Megaptera novaeangliae trnP	CAGGGAATAGTTTA-TGTAGAATTTCAGCTTTGGGTGCTGATGGTGGAGTGT-AATACTTCTTCCTTGA
Balaenoptera musculus trnP	CAGGGAATAGTTTA-TATAGAATTTCAGCTTTGGGTGCTGATGGTGGAGTGT-GATGCTTCTTCCTTGA
Balaenoptera omurai trnP	CAGGGAATAGTTTA-TGTAGAATTTCAGCTTTGGGTGCTGATGGTGGAGTAG-AACACTTCTTCCTTGA
Balaenoptera borealis trnP	CAGGGAATAGTTTA-TGTAGAATTTCAGCTTTGGGTGCTGATGGTGGAGTGT-AATACTTCTTCCTTGA
Balaenoptera brydei trnP	CAGGGAATAGTTTA-TGTAGAATTTCAGCTTTGGGTGCTGATGGTGGAGTGT-AATACTTCTTCCTTGA
Balaenoptera edeni trnP	CAGGGAATAGTTTA-TGTAGAATTTCAGCTTTGGGTGCTGATGGTGGAGTGT-AATACTTCTTCCTTGA
Kogia breviceps trnP	CAAGGAATAGTTTA-TGCAGAATTTCAGCTTTGGGTGTTGATGGTGGGGTATGAATGCCTCTTCCTTGA
Physeter macrocephalus trnP	CAAGGAATAGTTTA-TGTAGAATTTCAGCTTTGGGTGCTGATGGTGAGGTTT-AGTGCTTCTTCCTTGA
Platanista minor trnP	CAGGGAATAGTTTA-TGTAGAATTTCAGCTTTGGGTGCTGGCGGTGAGGTAT-AGTACTTCTTCCTTGA
Ziphius cavirostris trnP	CAGGGAGTAGTTTA-TGTAGAATTTCAGCTTTGGGTGTTGATGGTGAGGCGT-AATGCTTCTTCCCTGA
Mesoplodon densirostris trnP	CAGGGAATAGTTTA-TGTAGAATTTCAGCTTTGGGTGTTGATGGTGGAGCGT-AATGCTTCTTCCCTGA
Mesoplodon europaeus trnP	CAGGGAATAGTTTA-TGTAGAATTTCAGCTTTGGGTGTTGATGGTGAAGTGT-GATGCTTCTTCCCTGA
***Mesoplodon grayi* trnP	CAGGGAATAGTTTA-TGTAGAATTTCAGCTTTGGGTGTTGATGGTGAAGCGT-AATGCTTCTTCCCTGA
Berardius bairdii trnP	CAGGGAATAGTTTA-TGTAGAATTTCAGCTTTGGGTGTTGATGGTGAAGTGT-AATGCTTCTTCCCTGA
Hyperoodon ampullatus trnP	CAGGGAATAGTTTA-TGTAGAATTTCAGCTTTGGGTGTTGATGGTGAAGTGTAATACTTCTTCCCTGA
Lipotes vexillifer trnP	CAAGAAATAGTTTA-TGTAGAACTCCAGCTTTGGGGGTTGGTGGTGAGGTTT-GATATCTCTTTCTTGA
Inia geoffrensis trnP	CAAGGAATAGTTTA-TAGAGAATCCCAGCTTTGGGTGTTGGTGGTGAGGTTT-GGTGCTTCTTCCTTGA
Pontoporia blainvillei trnP	CAGGGAATAGTTTA-TAAAGAATTCCAGCTTTGGGTGTTGGTGGTGAGTTTT-AATATCTCTTCCTTGA
Monodon monoceros trnP	CAAGGAATAGTTTA-TGTAGAATCCCAGCTTTGGGTGTTGGTGGTGAGGTTC-AATGTCTCTTCCTTGA
***Neophocaena asiaeorientalis* trnP	CAAGGAGTAGTTTA-TAAAGAATTCCAGCTTTGGGTGTTGGTGGTGAGGTTTAAATGTCTCTTCCTTGA
Neophocaena phocaenoides trnP	CAAGGAGTAGTTTA-TAAAGAATTCCAGCTTTGGGTGTTGGTGGTGAGGTTTAAATGTCTCTTCCTTGA
Phocoena phocoena trnP	CAAGGAGTAGTTTA-TAAAGAATTCCAGCTTTGGGTGTTGGTGGTGAGGTTTGAATGTCTCCTCCTTGA
Cephalorhynchus heavisidii trnP	CAAGGAATAGTTTA-TGTAGAATTCCAGCTTTGGGTGTTGGTGGTGAGGTTT-AATGTCTCTTCCTTGA
Sousa chinensis trnP	CAAGGAATAGTTTA-TGTAGAATTCCAGCTTTGGGTGTTGGTGGTGAGGTTT-AATGTCTCTTCCTTGA
Stenella attenuata trnP	CAAGGAATAGTTTA-TGTAGAATTCCAGCTTTGGGTGTTGGTGGTGAGGTTT-AATGTCTCTTCCTTGA
Tursiops australis trnP	CAAGGAATAGTTTA-TGTAGAATTCCAGCTTTGGGTGTTGGTGGTGAGGTTT-AATATCTCTTCCTTGA
Tursiops truncatus trnP	CAAGGAATAGTTTA-TGTAGAATTCCAGCTTTGGGTGTTGGTGGTGAGGTTT-AATGTCTCTTCCTTGA
Tursiops aduncus trnP	CAAGGAATAGTTTA-TGTAGAATTCCAGCTTTGGGTGTTGGTGGTGAGGTTT-AATGTCTCTTCCTTGA
Delphinus capensis trnP	CAAGGAATAGTTTA-TGTAGAATTCCAGCTTTGGGTGTTGGTGGTGAGGTTT-AATGTCTCTTCCTTGA
Stenella coeruleoalba trnP	CAAGGAATAGTTTA-TGTAGAATTCCAGCTTTGGGTGTTGGTGGTGAGGTTT-AATGTCTCTTCCTTGA
Orcaella brevirostris trnP	CAAGGAATAGTTTA-TGTAGAATTCCAGCTTTGGGTGTTGGTGGTGAGGTTT-AATGTCTCTTCCTTGA
Orcaella heinsohni trnP	CAAGGAATAGTTTA-TGTAGAATTCCAGCTTTGGGTGTTGGTGGTGAGGTTT-AATGTCTCTTCCTTGA
Grampus griseus trnP	CAAGGAATAGTTTA-TGTAGAATTCCAGCTTTGGGTGTTGGTGGTGAGGTTT-AATGTCTCTTCCTTGA
Pseudorca crassidens trnP	CAAGGAATAGTTTA-TGTAGAATTCCAGCTTTGGGTGTTGGTGGTGAGGTTT-AATGTCTCTTCCTTGA
Feresa attenuata trnP	CAAGGAATAGTTTA-TGTAGAATTCCAGCTTTGGGTGTTGGTGGTGAGGTTT-AATGTCTCTTCCTTGA
Peponocephala electra trnP	CAAGGAATAGTTTA-TGTAGAATTCCAGCTTTGGGTGTTGGTGGTGAGGTTT-AATGTCTCTTCCTTGA
Globicephala macrorhynchus trnP	CAAGGAATAGTTTA-TGTAGAATTCCAGCTTTGGGTGTTGGTGGTGAGGTTT-AATGTCTCTTCCTTGA
Globicephala melas trnP	CAAGGAATAGTTTA-TGTAGAATTCCAGCTTTGGGTGTTGGTGGTGAGGTTT-AATGTCTCTTCCTTGA
Lagenorhynchus albirostris trnP	CAAGGAATAGTTTA-TGTAGAATTCCAGCTTTGGGTGTTGGTGGTGAGGTTT-AATGTCTCTTCCTTGA
Orcinus orca WNPTRU1 trnP	CAAGGAATAGTTTA-TGTAGAATTCCAGCTTTGGGTGTTGGTGGTGAGGTTT-AATGTCTCTTCCTTGA
Orcinus orca AntA1 trnP	CAAGGAATAGTTTA-TGTAGAATTCCAGCTTTGGGTGTTGGTGGTGAGGTTT-AATGTCTCTTCCTTGA
Orcinus orca AntB1 trnP	CAAGGAATAGTTTA-TGTAGAATTCCAGCTTTGGGTGTTGGTGGTGAGGTTT-AATGTCTCTTCCTTGA
Orcinus orca AntC1 trnP	CAAGGAATAGTTTA-TGTAGAATTCCAGCTTTGGGTGTTGGTGGTGAGGTTT-AATGTCTCTTCCTTGA
Orcinus orca ENAHN1 trnP	CAAGGAATAGTTTA-TGTAGAATTCCAGCTTTGGGTGTTGGTGGTGAGGTTT-AATGTCTCTTCCTTGA
Orcinus orca CNPNRAL trnP	CAAGGAATAGTTTA-TGTAGAATTCCAGCTTTGGGTGTTGGTGGTGAGGTTT-AATGTCTCTTCCTTGA
Orcinus orca ENPOAL2 trnP	CAAGGAATAGTTTA-TGTAGAATTCCAGCTTTGGGTGTTGGTGGTGAGGTTT-AATGTCTCTTCCTTGA
Structure	1234567 1234 4321 12345 ant 54321 1234 4321 7654321 d
Position (tens)	0000000 1111 2222 22222 333 33344 4444 5566 6666666 6
Position (units)	1234567 0123 0123 56789 234 78901 6789 8901 2345678 9

N, the most common base for the position.
N, half compensatory base change in the stem pair (e.g. T – G vs C – G; A-T vs G-T).
N, N, half compensatory base change in the stem pair exhibiting a mismatch (e.g. T–A vs A–A). Different colours are used to better differentiate the changes.
N, fully compensatory base change in the stem pair exhibiting a mismatch (e.g. C–G vs T–T).
N, type I fully compensatory base change in the stem pair (i.e. purine – pyrimidine vs purine – pyrimidine, e.g. G – C vs A – T).
N, N, type II fully compensatory base change in the stem pair (i.e. purine – pyrimidine vs pyrimidine – purine, e.g. A – T vs T – A). Different colours are used to better differentiate the changes.
N, a mismatch in the in the stem pair; N, substitution pattern not modelled; *, pair in the stem in which a mismatch is prominent. N, N, molecular signature for a taxon.
X, position 1-7 in the acceptor stem; X position 1-4 in the DHU stem; X position 1-5 in the anticodon stem; X position 1-4 in the TΨC stem; ant, anticodon; d, discriminator nucleotide.

trnQ (GLN) multiple alignment

```
                                     10        20        30        40        50        60        70
                                      |         |         |         |         |         |         |
                                1234567  1234              4321 12345  ant  54321    12345     543217654321d
                                    *                                                                 *
Ceratotherium simum trnQ        TAGAATGTGGTGTAG---TTTGGTAGCACGAAGATTTTTGAGTTCTTAGGAGTAGGTTCAATTCCTATAATTCTAG
Equus caballus trnQ             TAGGACATGGTGTAA---TTCGGTAGCACGGAGGATTTTGAATTCTCAGGAGCAGGTTCAATTCCTGTAGTTCTAG
Pecari tajacu trnQ              TAGAATGTGGTGTAG---TTTGGTAGCACAAGGAATTTTGAGTTCTTAGTTATAGGTTCGAGTCCTATTGTTCTAG
Phacochoerus africanus trnQ     TAGAATGTGGTGTAT---TTTGGTAGCACGGAGAATTTTGAATTCTCAGGTTTAGGTTCGAGTCCTATTGTTCTAG
Potamochoerus porcus trnQ       TAGAATGTGGTGTAT---TTTGGTAGCACGGAGAATTTTGAATTCTCAGGTTTAGGTTCGAGTCCTATTGTTCTAG
Sus scrofa trnQ                 TAGAATGTGGTGTAT---TTTGGTAGCACGGAGAATTTTGAATTCTCAGGTTTAGGTTCGAGTCCTATTGTTCTAG
Camelus bactrianus trnQ         TAGAATGTGGTGTAG---CGTGGTAGCACGAAGAATTTTGAATTCTTAGGGGTAGGTTCAATTCCTACGGTTCTAG
Camelus dromedarius trnQ        TAGAATGTGGTGTAG---TGTGGTAGCACGGAGAATTTTGAATTCTCAGGGGTAGGTTCAATTCCTACGGTTCTAG
Lama guanicoe trnQ              TAGAATATGGTGTAG---TGTGGTAGCACGAAGAATTTTGAATTCTTAGGGGTAGGTTCAACTCCTGCAGTTCTAG
Vicugna pacos trnQ              TAGAACATGGTGTAG---TGTGGTAGCACGAAGAATTTTGAATTCTTAGGGGTAGGTTCAACTCCTGCAGTTCTAG
Tragulus kanchil trnQ           TAGAATGTGGTGTGA---TCAGGTAGCACGAAGAATTTTGAATTCTTAGGGTGGGTTCAATTCCCATAGTTCTAG
Hyemoschus aquaticus trnQ       TAGAATGTGGTGTAAA--T-TGGTAGCACGGAGAATTTTGAATTCTTAGGGGTAGGTTCGATTCCTATTGTTCTAG
Giraffa camelopardalis trnQ     TAGAGTTTGGTGTAA-----TGGGAGCACGGAGAGTTTTGGATTCTTAAGGATAGGTTCAATTCCTGTAGCTCTAG
Okapia johnstoni trnQ           TAGAATTTGGTGTAG---T-TGGGAGCACGGAGAGTTTTGGGTTCTTAGGGGTAGGTTCAATTCCTATAATTCTAG
Antilocapra americana trnQ      TAGAATTTGGTGTAA---T-TGGGAGCACGGAGAGTTTTGGGTTCTCAGGAGTGGGTTCAATTCCTATAATTCTAG
Moschus moschiferus trnQ        TAGAATTTGGTGTAA---T-TGGGAGCACGAAGAGTTTTGGGTTCTTAGGAGTAGGTTCAATTCCTATAGTTCTAG
Muntiacus muntjak trnQ          TAGAATTTGGTGTAA---T-TGGGAGCACAAAGAGTTTTGGATTCTTGAGAGTAGGTTCAACTCCTATAGTTCTAG
Capreolus capreolus trnQ        TAGAATTTGGTGTAA---T-TGGGAGCACGAAGAGTTTTGGATTCTTAGGAGTAGGTTCAATTCCTATAGTTCTAG
Alces alces trnQ                TAGAATTTGGTGTAA---T-TGGGAGCACAAAGAGTTTTGGATTCTTAGGAGTAGGTTCAATTCCTGTAGTTCTAG
Cervus elaphus trnQ             TAGAATTTGGTGTAT---T-TGGGAGCACAAAGAGTTTTGGATTCTTGGGAGTAGGTTCAATTCCTATAATTCTAG
Dama dama trnQ                  TAGAATTTGGTGTAT---T-TGGGAGCACGAAGAGTTTTGGATTCTTGGGAGTAGGTTCAATTCCTATAATTCTAG
Boselaphus tragocamelus trnQ    TAGAATTTGGTGTAAA--T-TGGGAGCACGAAGAGTTTTGAGTTCTTAGGAGTAGGTTCAATTCCTATAGTTCTAG
Tragelaphus oryx trnQ           TAGAGTTTGGTGTAC---T-TGGTAGCACGAAGAATTTTGGGTTCTTAGGGGTAGGTTCAATTCCTATAGTTCTAG
Bos taurus trnQ                 TAGAATTTGGTGTAA---T-TGGGAGCACGAAGAGTTTTGGATTCTTAGGAGTAGGTTCGATTCCTATAGTTCTAG
Bubalus bubalis trnQ            TAGGACTTGGTGTAA---T-TGGGAGCACGAAGAGTTTTGGGTTCTTAGGATTAGGTTCAATTCCTATAGTTCTAG
Syncerus caffer trnQ            TAGGACTTGGTGTAA---T-TGGGAGCACGAAGAGTTTTGGGTTCTTAGGATTAGGTTCAACTCCTATAGTCCTAG
Neotragus moschatus trnQ        TAGAATTTGGTGTAA---T-AGGGAGCACGGAGAGTTTTGGATTCTCAAGAGTAGGTTCAATTCCTATGGTTCTAG
Alcelaphus buselaphus trnQ      TAGAATTTGGTGTAA---C-TGGGAGCACGAGGAGTTTTGAGTTCTTAGGAGTAGGTTCGATCCCTATAGTTCTAG
Oryx gazella trnQ               TAGAATTTGGTGTAA---T-TGGGAGCACAAAGAGTTTTGGGTTCTTAGGAGTAGGTTCAATTCCTATAGTTCTAG
Pantholops hodgsonii trnQ       TAGAATTTGGTGTAA---T-TGGGAGCACGAAGAGTTTTGGGTTCTTAGGAGTAGGTTCAATTCCTATAGTTCTAG
Ovibos moschatus trnQ           TAGAATTTGGTGTAC---T-TGGGAGCACGAAGAGTTTTGGGTTCTTAGGAGTAGGTTCGATCCCTATAATTCTAG
Capra hircus trnQ               TAGAATTTGGTGTAA---T-TGGGAGCACGAAGAGTTTTGGGTTCTTAGGAGTAGGTTCAATTCCTATAATTCTAG
Ovis aries trnQ                 TAGAATTTGGTGTAA---T-TGGGAGCACGAAGAGTTTTGGGTTCTTAGGAGTAGGTTCAATTCCTATAGTTCTAG
Cephalophus natalensis trnQ     TAGGATTTGGTGTAA---T-TGGGAGCACGAAGAGTTTTGGGTTCTTAGGAGTAGGTTCAATTCCTATAGTTCTAG
Redunca fulvorufula trnQ        TAGAACTTGGTGTAA---T-TGGGAGCACGAAGAGTTTTGGGTTCTTAGGAGTAGGTTCAACTCCTATAGTTCTAG
Ourebia ourebi trnQ             TAGAGTTTGGTGTAA---T-TGGGAGCACGAAGAGTTTTGAGTTCTTAGGAGTAGGTTCAATTCCTATAATTCTAG
Antilope cervicapra trnQ        TAGAATTTGGTGTAT---T-TGGGAGCACGAAGAGTTTTGGATTCTTAGGAGTAGGTTCAATTCCTATGATTCTAG
Gazella gazella trnQ            TAGAATTTGGTGTAT---T-TGGGAGCACGAAGAGTTTTGGGTTCTTAGGAGTAGGTTCAATTCCTATAGTTCTAG
Procapra gutturosa trnQ         TAGAATTTGGTGTAA---T-TGGGAGCACGAAGAGTTTTGGACTCTTAGGAGTAGGTTCGATTCCTGTGATTCTAG
Raphicerus campestris trnQ      TAGAATTTGGTGTAA---T-TGGGAGCACGAAGAGTTTTGGGTTCTTAGGAGTAGGTTCAATTCCTATAGTTCTAG
Hexaprotodon liberiensis trnQ   TGGAACAGGGTGTGA---T-TGGTAGCACAGGGAATTTTGAATTCTCAGGAGTAGGTTCGATTCCTATAGTTCTAG
Hippopotamus amphibius trnQ     TGGAACGGGGTGTGA---T-TGGTAGCACGGGGAATTTTGAGTTCTCAGGAGTAGGTTCGATTCCTATAGTTCTAG
Eubalaena australis trnQ        TAGATTGTAGTGTAA---CATGGTAGCACGAAGAACTTTGAATTCTTAAGGGTAGGTTCAATTCCTATTATTCTAG
Eubalaena japonica trnQ         TAGATTGTAGTGTAA---CATGGTAGCACGAAGAACTTTGAATTCTTAAGGGTAGGTTCAATTCCTATTATTCTAG
Balaena mysticetus trnQ         TAGATTGTAGTGTAA---TATGGTAGCACGAAGAACTTTGAATTCTTAAGGGTAGGTTCAATTCCTATTATTCTAG
Caperea marginata trnQ          TAGATTATGGTGTGA---TGTGGTAGCACGAAGAACTTTGAATTCTTAAGGGTAGGTTCAATTCCTATTATTCTAG
Eschrichtius robustus trnQ      TAGATTGTAGTGTAG---CGTGGTAGCACGAAGAACTTTGAATTCTTAAGGGTAGGTTCGATTCCTATTATTCTAG
Balaenoptera acutorostrata trnQ TAGATTGTAGTGTAA---TATGGTAGCACGAAGAACTTTGAATTCTTAAGGGTAGGTTCGACTCCTATTATTCTAG
Balaenoptera bonaerensis trnQ   TAGATTGTAGTGTAA---TATGGTAGCACGAAGAACTTTGAATTCTTAAGGGTAGGTTCGATTCCTATTATTCTAG
Balaenoptera physalus trnQ      TAGATTGTAGTGTAA---TATGGTAGCACGAAGAACTTTGAATTCTTAAGGGTAGGTTCGATTCCTATTATTCTAG
Megaptera novaeangliae trnQ     TAGATTGTAGTGTAA---TGTGGTAGCACGAAGAACTTTGAATTCTTAAGGGTAGGTTCGATTCCTATTATTCTAG
Balaenoptera musculus trnQ      TAGATTGTAGTGTAA---TATGGTAGCACGAAGAACTTTGAATTCTTAAGGGTAGGTTCGATTCCTATTATTCTAG
Balaenoptera omurai trnQ        TAGATTGTAGTGTAA---TATGGTAGCACGAAGAACTTTGAATTCTTAAGGGTAGGTTCGATTCCTATTATTCTAG
Balaenoptera borealis trnQ      TAGATTGTAGTGTAA---TATGGTAGCACGAAGAATTTTGAATTCTTAAGGGTAGGTTCGATTCCTATTATTCTAG
Balaenoptera brydei trnQ        TAGATTGTAGTGTAA---CATGGTAGCACGAAGAATTTTGAATTCTTAAGGGTAGGTTCGATTCCTATTATTCTAG
Balaenoptera edeni trnQ         TAGATTGTAGTGTAA---CATGGTAGCACGAAGAATTTTGAATTCTTAAGGGTAGGTTCGATTCCTATTATTCTAG
Kogia breviceps trnQ            TAGATTGTAGTGTAGCAATGTGGTAGCACGAAGAACTTTGAATTCTTAAGGGTAGGTTCGACTCCTATTATTCTAG
Physeter macrocephalus trnQ     TGGATTGTAGTGTAGCAATGTGGTAGCACGAAGAACTTTGAATTCTTAAGGATAGGTTCAACTCCTATTATTCTAG
Platanista minor trnQ           TAGATTGTGGTGTAG---TGTGGTAGCACAGAGAATTTTGAATTCTCAGGGATAGGTTCAATTCCTATTGTTCTAG
Ziphius cavirostris trnQ        TAGATTATGGTGTAA---TATGGTAGCACGGAGAACTTTGAATTCTTAGGGGTAGGTTCAATTCCTACTATTCTAG
Mesoplodon densirostris trnQ    TAGATTATAGTGTAA---TATGGTAGCACGGAGAACTTTGAATTCTTAAGGGTAGGTTCGATTCCTATTATTCTAG
Mesoplodon europaeus trnQ       TAGATTGTAGTGTAA---TATGGTAGCACGGAGAACTTTGAATTCTTAAGAGTAGGTTCGATTCCTATTATTCTAG
**Mesoplodon grayi trnQ         TAGATTGTGGTGTAA---TATGGTAGCACGGGGAATTTTGAATTCTCAAGAGTAGGTTCGATTCCTATTATTCTAG
Berardius bairdii trnQ          TAGATTATGGTGTAA---TATGGTAGCACGGAGAACTTTGAATTCTTAGGGGTAGGTTCGATTCCTATTATTCTAG
Hyperoodon ampullatus trnQ      TAGATTATAGTGTAA---TGTGGTAGCACGAAGAAGTTTGAATTCTTAAGGGTAGGTTCGATTCCTATTATTCTAG
Lipotes vexillifer trnQ         TGGATTATGGTGTAA---TATGGTAGCACGGAGAACTTTGAATTCTTAAGGGTAGGTTCGATTCCTACTGTTCTAG
Inia geoffrensis trnQ           TAGATTATGGTGTAA---TATGGTAGCACGGAGAACTTTGAATTCTTAGGGGTAGGTTCGATTCCTATTATTCTAG
Pontoporia blainvillei trnQ     TAGGGTATGGTGTTA---TATGGTAGCACGGAGAATTTTGAATTCTCAGGGGTAGGTTCGATTCCTATTATTCTAG
Monodon monoceros trnQ          TAGATTATAGTGTGA---TACGGTAGCACGGAGAATTTTGAATTCTTAAGGATAGGTTCAATTCCTATTGTTCTAG
**Neophocaena asiaeorientalis trnQ TAGATTATGGTGTGA---TGTGGTAGCACGGGGAATTTTGAATTCTCAGGAGTAGGTTCGATTCCTATTATTCTAG
Neophocaena phocaenoides trnQ   TAGATTATGGTGTGA---TGTGGTAGCACGGGGAATTTTGAATTCTCAGGAGTAGGTTCGATTCCTATTATTCTAG
Phocoena phocoena trnQ          TAGATTATGGTGTGA---TATGGTAGCACGGAGAATTTTGAATTCTCAGGGGTAGGTTCGATTCCTATTATTCTAG
Cephalorhynchus heavisidii trnQ TAGATTATGGTGTAG---TGTGGTAGCACGGAGAATTTTGAATTCTTAAGGGTAGGTTCGATTCCTATTATTCTAG
Sousa chinensis trnQ            TAGATTATGGTGTAG---TGTGGTAGCACGAAGAATTTTGAATTCTTAAGGGTAGGTTCGACTCCTATTGTTCTAG
Stenella attenuata trnQ         TAGATTATGGTGTAG---TGTGGTAGCACGAAGAATTTTGAATTCTTAAGGGTAGGTTCGATTCCTATTATTCTAG
Tursiops australis trnQ         TAGATTATGGTGTAG---TGTGGTAGCACTAAGAATTTTGAATTCTTAAGGGTAGGTTCGACTCCTATTATTCTAG
Tursiops truncatus trnQ         TAGATTATGGTGTAG---TGTGGTAGCACAAAGAATTTTGAATTCTTAAGGGTAGGTTCGACTCCTATTGTTCTAG
Tursiops aduncus trnQ           TAGATTATGGTGTAG---TGTGGTAGCACGAAGAATTTTGAATTCTTAAGGGTAGGTTCGATTCCTATTGTTCTAG
Delphinus capensis trnQ         TAGATTATGGTGTAG---TGTGGTAGCACGAAGAATTTTGAATTCTTAAGGGTAGGTTCGATTCCTATTGTTCTAG
Stenella coeruleoalba trnQ      TAGATTATGGTGTAG---TGTGGTAGCACGAAGAATTTTGAATTCTTAAGGGTAGGTTCGATTCCTATTGTTCTAG
Orcaella brevirostris trnQ      TAGATTATGGTGTAG---TGTGGTAGCACAAAGAATTTTGAATTCTTAAGGGTAGGTTCGATTCCTATTATTCTAG
Orcaella heinsohni trnQ         TAGATTATGGTGTAG---TGTGGTAGCACGAAGAATTTTGAATTCTTAAGGGTAGGTTCGATTCCTATTGTTCTAG
Grampus griseus trnQ            TAGATTATGGTGTAG---CGTGGTAGCACGAAGAATTTTGAATTCTTAAGGGTAGGTTCGATTCCTATTATTCTAG
Pseudorca crassidens trnQ       TAGATTATGGTGTAG---TGTGGTAGCACGAAGAATTTTGAATTCTTAGGGGTAGGTTCGATTCCTATTGTTCTAG
Feresa attenuata trnQ           TAGATTATGGTGTAG---TGTGGTAGCACGAAGAATTTTGAATTCTTAAGGGTAGGTTCGATTCCTATTGTTCTAG
Peponocephala electra trnQ      TAGATTATGGTGTAG---TGTGGTAGCACGAAGAATTTTGAATTCTTAAGGGTAGGTTCGATTCCTATTGTTCTAG
Globicephala macrorhynchus trnQ TAGATTATGGTGTAG---TGTGGTAGCACGAAGAACTTTGAATTCTTAAGGGTAGGTTCGATTCCTATTGTTCTAG
Globicephala melas trnQ         TAGATTATGGTGTAG---TGTGGTAGCACGAAGAACTTTGAATTCTTAAGGGTAGGTTCGATTCCTATTGTTCTAG
Lagenorhynchus albirostris trnQ TAGATTATGGTGTAG---TGTGGTAGCACGAAGAACTTTGAATTCTTAAGGGTAGGTTCGATTCCTATTATTCTAG
Orcinus orca WNPTRU1 trnQ       TAGACTATGGTGTGG---TGTGGTAGCACGGAGAATTTTGAATTCTTAGGGGTAGGTTCGATTCCTACTATTCTAG
Orcinus orca AntA1 trnQ         TAGACTATGGTGTGG---TGTGGTAGCACGGAGAATTTTGAATTCTTAAGGGTAGGTTCGATTCCTACTATTCTAG
Orcinus orca AntB1 trnQ         TAGACTATGGTGTGG---TGTGGTAGCACGGAGAATTTTGAATTCTTAAGGGTAGGTTCGATTCCTACTATTCTAG
Orcinus orca AntC1 trnQ         TAGACTATGGTGTGG---TGTGGTAGCACGGAGAATTTTGAATTCTTAAGGGTAGGTTCGATTCCTACTATTCTAG
Orcinus orca ENAHN1 trnQ        TAGACTATGGTGTGG---TGTGGTAGCACGGAGAATTTTGAATTCTTAAGGGTAGGTTCGATTCCTACTATTCTAG
Orcinus orca CNPNRAL trnQ       TAGACTATGGTGTGG---TGTGGTAGCACGGAGAATTTTGAATTCTTAAGGGTAGGTTCGATTCCTACTATTCTAG
Orcinus orca ENPOAL2 trnQ       TAGACTATGGTGTGG---TGTGGTAGCACGGAGAATTTTGAATTCTTAAGGGTAGGTTCGATTCCTACTATTCTAG
                                    *                                                                  *
                                1234567  1234              4321 12345  ant  54321    12345     543217654321d
                                0000000  1111              2222 33333  334  44444    55555     666667777777
                                1234567  0123              6789 12345  890  34567    23456     456789012345 6
```

- N, the most common base for the position.
- N, half compensatory base change in the stem pair (e.g. T – G vs C – G; A-T vs G-T).
- N, N, half compensatory base change in the stem pair exhibiting a mismatch (e.g. T–A vs A–A). Different colours are used to better differentiate the changes.
- N, fully compensatory base change in the stem pair exhibiting a mismatch (e.g. C–G vs T–T).
- N, type I fully compensatory base change in the stem pair (i.e. purine – pyrimidine vs purine – pyrimidine, e.g. G – C vs A – T).
- N, N, type II fully compensatory base change in the stem pair (i.e. purine – pyrimidine vs pyrimidine – purine, e.g. A – T vs T – A). Different colours are used to better differentiate the changes.
- N, a mismatch in the in the stem pair; N, substitution pattern not modelled; *, pair in the stem in which a mismatch is prominent. N, N, molecular signature for a taxon.
- X, position 1-7 in the acceptor stem; X position 1-4 in the DHU stem; X position 1-5 in the anticodon stem; X position 1-5 in the TΨC stem; **ant**, anticodon; **d**, discriminator nucleotide.

***trnR* (ARG) multiple alignment**

```
                                                  10        20        30        40        50        60        70
                                                  |         |         |         |         |         |         |
                                       1234567  1234        4321 12345   ant   54321     12345        543217654321d
Ceratotherium simum trnR               TGATAATTAGTTTAAAC-TAAAATAAATGATTTCGACTCATTAGACTATGATT-T-ACTTCATAATTATCAA
Equus caballus trnR                    TGGTAATTAGTTTAAAC-CAAAACAAATGATTTCGACTCATTAAACTATGATT--AACTTCATAATTACCAA
Pecari tajacu trnR                     TGATAATTAGTTTAAG--TAAAATAAATGATTTCGACTCATTAGACTATGATT-C-ATCTCATAATTATCAT
Phacochoerus africanus trnR            TGATAATTAGTTTAAAA-CAAAACAAATGATTTCGACTCATTAGACTATGATT-T-ACTTCATAATTATCAA
Potamochoerus porcus trnR              TGATAATTAGTTTAAAA-CAAAACAAATGATTTCGACTCATTAGACTATGATT-T-ATCTCATAATTATCAA
Sus scrofa trnR                        TGATAATTAGTTTAAAA-CAAAACAAATGATTTCGACTCATTAGACTATGATT-T-ACTTCATAATTATCAA
Camelus bactrianus trnR                TGGTAGTTAGTTTAAA--CAAAATTAATGATTTCGACTCATTAGATTATGATT--AACTTCATAACTACCAA
Camelus dromedarius trnR               TGGTAGTTAGTTTAAA--CAAAATTATTGATTTCGACTCATTAGATTATGATT--AACCTCATAACTACCAA
Lama guanicoe trnR                     TGATAATTAGTTTAAA--TAAAATTAATGATTTCGACTCATTAGATTATGATT--AAGTTCATAATTATCAA
Vicugna pacos trnR                     TGATAATTAGTTTAAA--TAAAATTAATGATTTCGACTCATTAGATTATGATT--AAGTTCATAATTATCAA
Hyemoschus aquaticus trnR              TGGTATTTAGTTTAAA--CAAAATAAATGATTTCGACTCATTAGATTATGATT--AAGTTCATAACTACCAA
Tragulus kanchil trnR                  TGGTACTTAGTTTAAA--CAAAACAAATGATTTCGACTCATTAGATTATGATT--AAGCTCATAACTACCAA
Giraffa camelopardalis trnR            TGGTAATTAGTTTAAGC-AAAAATAAATGATTTCGACTCATTAGATTATGATC--AAATTCATAATTACCAA
Okapia johnstoni trnR                  TGGTAATTAGTTTAAAA-CAAAATAAATGATTTCGACTCATTAGACTATGACT--AAACTCATAATTACCAA
Antilocapra americana trnR             TGGTATTTAGTTTAAAA-CAAAATAAATGATTTCGACTCATTAGACTATGATT--AAATTCATAATTACCAA
Moschus moschiferus trnR               TGGTACTTAGTTTAAAA-TAAAATAAATGATTTCGACTCATTAGATTATGATC--AAGTTCATAATTACCAA
Muntiacus muntjak trnR                 TGGTATGTAGTTTAAAA-TAAAATAAATGATTTCGACTCATTAGATTATGATT-T-AATTCATAATTACCAA
Capreolus capreolus trnR               TGGTACTTAGTTTAAAG-AAAAACAAATGATTTCGACTCATTAGATTATGATT---ACCTCATAATTACCAA
Alces alces trnR                       TGGTACTTAGTTTAAAA-TAAAATAAATGATTTCGACTCATTAGATTATGATT--AAATTCATAATTACCAA
Cervus elaphus trnR                    TGGTATTTAGTTTAAAA-CAAAATAAATGATTTCGACTCATTAGATTGTGATT--AAATTCATAATTACCAA
Dama dama trnR                         TGGTATTTAGTTTAAAA-CAAAATAAATGATTTCGACTCATTAGATTATGATT--AAATTCATAACTACCAA
Boselaphus tragocamelus trnR           TGGTATTTAGTTTAAAA-CAAAATAAATGATTTCGACTCATTAGATTATGATT--AAACTCATAATTACCAA
Tragelaphus oryx trnR                  TGGTACTTAGTTTAAAA-TAAAATGAATGATTTCGACTCATTAGATTATGATT-TAAACTCATAATTACCAA
Bos taurus trnR                        TGGTACTTAGTTTAAAA-TAAAATAAATGATTTCGACTCATTAGATTATGATT-T-AATTCATAATTACCAA
Bubalus bubalis trnR                   TGGTATTTAGTTTAAAA-TAAAATAAATGATTTCGACTCATTAGATTATGATC--AAACTCATAACTACCAA
Syncerus caffer trnR                   TGGTACTTAGTTTAAAA-TAAAATAAATGATTTCGACTCATTAGATTGTGATT--AAACTCATAATTACCAA
Neotragus moschatus trnR               TGGTATTTAGTTTAAAA-CAAAATAAATGATTTCGACTCATTAGATTATGATC--AAATTCATAATTACCAA
Alcelaphus buselaphus trnR             TGGTATTTAGTTTAAAG-CAAAATAAATGATTTCGACTCATTAGATTATGATC--AAACTCATAATTACCAA
Oryx gazella trnR                      TGGTACTTAGTTTAAAA-TAAAATAAATGATTTCGACTCATTAGATTATGATT--AAACTCATAATTACCAA
Pantholops hodgsonii trnR              TGGTATTTAGTTTAAGA-CAAAATAAATGATTTCGACTCATTAGATTATGATT--AAACTCATAACTACCAA
Ovibos moschatus trnR                  TGGTATTTAGTTTAAAA-CAAAATAAATGATTTCGACTCATTAGATTATGATT--AAACTCATAATTACCAT
Capra hircus trnR                      TGGTATTTAGTTTAAAA-CAAAATAAGTGATTTCGACTCATTAGATTATGATT--AAACTCATAATTACCAA
Ovis aries trnR                        TGGTATTTAGTTTAAAA-CAAAATAAATGATTTCGACTCATTAGATTATGATT--AAGCTCATAACTACCAA
Cephalophus natalensis trnR            TGGTATTTAGTTTAAAA-CAAAATAAATGATTTCGACTCATTAGATTATGATC--AAACTCATAATTACCAA
Redunca fulvorufula trnR               TGGTATTTAGTTTAAAA-TAAAATAAATGATTTCGACTCATTAGATTGTGATT--AAGCTCATAATTACCAA
Ourebia ourebi trnR                    TGGTATTTAGTTTAAAAATAAAATAAATGATTTCGACTCATTAGATTATGATC-T-AATTCATAACTACCAA
Antilope cervicapra trnR               TGGTACTTAGTTTAAAA-TAAAATAAATGATTTCGACTCATTAGATTATGATT-T-AACTCATAATTACCAA
Gazella gazella trnR                   TGGTATTTAGTTTAAA--TAAAATAAATGATTTCGACTCATTAGATTATGATT-T-AACTCATAATTACCAA
Procapra gutturosa trnR                TGGTAATTAGTTTAAAA-TAAAACAAATGATTTCGACTCATTAGATTATGACT-C-AATTCATAATTACCAT
Raphicerus campestris trnR             TGGTACTTAGTTTAAC--TAAAATAAATGATTTCGACTCATTAGATTATGATT--AAACTCATAATTACCAA
Hexaprotodon liberiensis trnR          TGGTATTTAGTTTAAAA-CAAAACAAATGATTTCGACTCATTAATTATGAAT-C-AACCCGTAAATACCAA
Hippopotamus amphibius trnR            TGGTATTTAGTTTAAAA-CAAAACAAATGATTTCGACTCATTAATTATGAAC-C-AACTCATAAATACCAA
Eubalaena australis trnR               TGGTATTTAGTTTAAAA-CAAAACAAGTGATTTCGACCCACTAAACTGTGATT--AAACTCACAACTACCAA
Eubalaena japonica trnR                TGGTATTTAGTTTAAAA-CAAAACAAGTGATTTCGACTCACTAAACTGTGATT--AAACTCACAACTACCAA
Balaena mysticetus trnR                TGGTATTTAGTTTAAAA-CAAAACAAGTGATTTCGACTCACTAAACTGTGATC--AAACTCACAACTACCAA
Caperea marginata trnR                 TGGTATTTAGTTTAAAA-CAAAACAAGTGATTTCGACTCACTAAATTGTGATC--AAACTTACAACTACCAA
Eschrichtius robustus trnR             TGGCATTTAGTTTAAAA-TAAAACAAGTGGTTTCGACCCACTAGACTGTGATC--AAATTCACAATTACCAA
Balaenoptera acutorostrata trnR        TGGTATTTAGTTTAAAA-TAAAACAAGTGATTTCGACCCACTAGACTGTGACT--AGATTCACAATTACCAA
Balaenoptera bonaerensis trnR          TGGTACTTAGTTTAAAA-TAAAACAAGTGATTTCGACCCACTAGACTGTGACT--AAATTCACAATTACCAA
Balaenoptera physalus trnR             TGGTATTTAGTTTAAGA-TAAAACAAGTGATTTCGACCCACTAGACTGTGATC--AAATTCACAATTACCAA
Megaptera novaeangliae trnR            TGGTATTTAGTTTAAAA-CAAAACAAGTGATTTCGACCCACTAGACTGTGATC--AAATTCACAACTACCAA
Balaenoptera musculus trnR             TGGTATTTAGTTTAAAA-TAAAACAAGTGATTTCGACCCACTAGACTGTGATC--AAATTCACAACTACCAA
Balaenoptera omurai trnR               TGGTATTTAGTTTAAAA--AAAACAAGTGATTTCGACCCACTAGACTGTGATC--AAATTCACAACTACCAA
Balaenoptera borealis trnR             TGGTATTTAGTTTAAAA-TAAAACAAGTGATTTCGACCCACTAGACTGTGATC--AAATTCACAATTACCAA
Balaenoptera brydei trnR               TGGTATTTAGTTTAAGA-TAAAACAAGTGATTTCGACCCACTAGACTGTGATC--AAATTCACAATTACCAA
Balaenoptera edeni trnR                TGGTACTTAGTTTAAGA-TAAAACAAGTGATTTCGACCCACTAGACTGTGATC--AAATTCACAATTACCAA
Kogia breviceps trnR                   TGGTACTTAGTTTAAAA-CAAAACAAGTGATTTCGACTCACTAGACTGTGATC--AAACTCACAAATACCAA
Physeter macrocephalus trnR            TGGTACTTAGTTTAAAA-CAAAACAGGTGATTTCGACTCACTGGACTGTGATC--AAACTCACAAGTACCAA
Platanista minor trnR                  TGATACTTAGTTTAAAA-TAAAACAAGTGATTTCGACCCACTAAACTGTGACT-TAAGCCCACAAGTATCAA
Ziphius cavirostris trnR               TGGTACTTAGTTTAAAA-TAAAATAAGTGATTTCGACTCACTAGACTGTGATC-CAAACTCACAACTACCAA
Mesoplodon densirostris trnR           TGGTATTTAGTTTAAAC-TAAAATAAGTGATTTCGACTCACTAGACTGTGACCCAAAAATCACAAATACCAA
Mesoplodon europaeus trnR              TGGTATTTAGTTTAAAC-CAAAATAAGTGATTTCGACTCACTAGATTGCGATC-CAAACTCACAAATACCAA
**Mesoplodon grayi trnR                TGGTATTTAGTTTAAAA-CAAAATAAGTGATTTCGACTCACTAGACTGCGATC-CAAACTCACAAATACCAA
Berardius bairdii trnR                 TGGTACTTAGTTTAAAA-CAAAATAAGTGATTTCGACTCACTAGACTGTGATC-CAAACTCACAATTACCAA
Hyperoodon ampullatus trnR             TGGTACTTAGTTTAAAA-CAAAACAAGTGATTTCGACTCACTAGACTGTGATC-C-AACTCACAAATACCAG
Lipotes vexillifer trnR                TGGCACTTAGTTTAAAA--AAAACAAACGGTTTCGACCCGCTAGACTGCGATT-CAAGCTCGCAAGTACCAA
Inia geoffrensis trnR                  TGGTATTTAGTTTAAAA-TAAAATAAGTGATTTCGACCCACTCGACTGTGACA--AAACTCACAAATACCAA
Pontoporia blainvillei trnR            TGGTACTTAGTTTAAAA-CAAAACAAGTGATTTCGACTCACTAGATTGCGATC-TAAACTCACAAATACCAA
Monodon monoceros trnR                 TGGCACTTAGTTTAAAA-TAAAATGAGTGATTTCGACCCACTAGACTGTGATT-TAAATTCACAAGTACCAA
**Neophocaena asiaeorientalis trnR     TGATACTTAGTTTAAAG-TAAAATAAGTGATTTCGACCCACTAGACTGTGATT-TAAATTCACAAGTACCAA
Neophocaena phocaenoides trnR          TGATACTTAGTTTAAAG-TAAAATAAGTGATTTCGACCCACTAGACTGTGATT-TAAATTCACAAGTACCAA
Phocoena phocoena trnR                 TGGTACTTAGTTTAAGA-TAAAATAAGTGATTTCAACTCACTAGACTGTGATT-TAAACTCACAAGTACCAA
Cephalorhynchus heavisidii trnR        TGGTACTTAGTTTAAAG-TAAAACAAGTGATTTCGACCCACTAGACTGTGATC-TAAACTCACAAGTACCAA
Sousa chinensis trnR                   TGGTACTTAGTTTAAAG-TAAAACAAGTGGTTTCGACCCACTAGACTGTGATC-TAAACTCACAAGTACCAA
Stenella attenuata trnR                TGATACTTAGTTTAAAA-TAAAACAAGTGGTTTCGACCCACTAGACTGTGATC-TAAACTCACAAGTACCAA
Tursiops australis trnR                TGGTGCTTAGTTTAAAG-TAAAACAAGTGATTTCGACCCACTAGATTATGATC-CAAACTCACCAGTACCAA
Tursiops truncatus trnR                TGGTACTTAGTTTAAAG-TAAAACAAGTGGTTTCGACCCATTAGACTGTGATC-TAAACTCACAAGTACCAA
Tursiops aduncus trnR                  TGGTACTTAGTTTAAAG-TAAAATAAATGGTTTCGACCCATTAGACTGTGATC-TAAATTCACAAGTACCAA
Delphinus capensis trnR                TGGTACTTAGTTTAAAG-TAAAATAAATGGTTTCGACCCATTAGACTGTGATC-TAAACTCACAAGTACCAA
Stenella coeruleoalba trnR             TGGTACTTAGTTTAAAG-TAAAACAAGTGGTTTCGACCCATTAGACTGTGATC-CAAACTCACAAGCACCAA
Orcaella brevirostris trnR             TGATACTTAGTTTAAAA-TAAAACAAGTGATTTCGACCCACTAGACTGTGATC-CAAACTCACAAGTACCAA
Orcaella heinsohni trnR                TGATACTTAGTTTAAAT-CAAAACAAGTGGTTTCGACCCACTAGACTGTGATC-CAAACTCACAAGTACCAA
Grampus griseus trnR                   TGATACTTAGTTTAAAG-TAAAACAAGTGGTTTCGACCCACTAGACTGTGATC-CAAACTCACAAGTACCAA
Pseudorca crassidens trnR              TGGTACTTAGTTTAAAG-TAAAACAAGTGGTTTCGACCCACTGGACTGTGATC-CAAACTCACAAGTACCAG
Feresa attenuata trnR                  TGGTACTTAGTTTAAAA-TAAAACAAGTGATTTCGACCCACTAGACTGTGATC-TAAACTCACAAGTACCAA
Peponocephala electra trnR             TGGTACTTAGTTTAAAG-TAAAACAAGTGATTTCGACCCTCTAGACTGTGATC-TAAACTCACAAGTACCAA
Globicephala macrorhynchus trnR        TGGTATTTAGTTTAAAG-TAAAACAAGTGATTTCGACCCACTAGACTGTGATC-TAAACTCACAAATACCAA
Globicephala melas trnR                TGGTATTTAGTTTAAAG-TAAAACAAGTGATTTCGACCCACTAGACTGTGATC-TAAACTCACAAATACCAA
Lagenorhynchus albirostris trnR        TGATACTTAGTTTAAAA-TAAAACAAGTGGTTTCGACCCACTAGATTGTGACC-TAAGCTCACAAGTACCAA
Orcinus orca WNPTRU1 trnR              TGATACTTAGTTTAAAA-TAAAACAAGTGGTTTCGACCCACTAGACTGTGACC-TAAACCCACAAGTACCAA
Orcinus orca AntA1 trnR                TGATACTTAGTTTAAAA-TAAAACAAGTGGTTTCGACCCACTAGACTGTGACC-TAAACCCACAAGTACCAA
Orcinus orca AntB1 trnR                TGATACTTAGTTTAAAA-TAAAACAAGTGGTTTCGACCCACTAGACTGTGACC-TAAACCCACAAGTACCAA
Orcinus orca AntC1 trnR                TGATACTTAGTTTAAAA-TAAAACAAGTGGTTTCGACCCACTAGACTGTGACC-TAAACCCACAAGTACCAA
Orcinus orca ENAHN1 trnR               TGATACTTAGTTTAAAA-TAAAACAAGTGGTTTCGACCCACTAGACTGTGACC-TAAACCCACAAGTACCAA
Orcinus orca CNPNRAL trnR              TGATACTTAGTTTAAAA-TAAAACAAGTGGTTTCGACCCACTAGACTGTGACC-TAAACCCACAAGTACCAA
Orcinus orca ENPOAL2 trnR              TGATACTTAGTTTAAAA-TAAAACAAGTGGTTTCGACCCACTAGACTGTGACC-TAAACCCACAAGTACCAA
                                       1234567  1234        4321 12345   ant   54321     12345        543217654321d
                                       0000000  1111        2222 22223   333   33444     44455        666666666677 7
                                       1234567  0123        1234 67890   345   89012     78901        012345678901 2
```

- N, the most common base for the position.
- N, half compensatory base change in the stem pair (e.g. T – G vs C – G; A-T vs G-T).
- N, N, half compensatory base change in the stem pair exhibiting a mismatch (e.g. T–A vs A–A). Different colours are used to better differentiate the changes.
- N, fully compensatory base change in the stem pair exhibiting a mismatch (e.g. C–G vs T–T).
- N, type I fully compensatory base change in the stem pair (i.e. purine – pyrimidine vs purine – pyrimidine, e.g. G – C vs A – T).
- N, N, type II fully compensatory base change in the stem pair (i.e. purine – pyrimidine vs pyrimidine – purine, e.g. A – T vs T – A). Different colours are used to better differentiate the changes.
- N, a mismatch in the in the stem pair; N, substitution pattern not modelled; *, pair in the stem in which a mismatch is prominent. N, N, molecular signature for a taxon.
- X, position 1-7 in the acceptor stem; X position 1-4 in the DHU stem; X position 1-5 in the anticodon stem; X position 1-4(5) in the TΨC stem; **ant**, anticodon; **d**, discriminator nucleotide.

trnS1 [**SER** (**S1**, **AGY**)] multiple alignment

```
                                              10        20        30        40        50        60
                                               |         |         |         |         |         |
                                      1234567     012345 ant  543210    12345          5432176543210d
                                                    *             *
Ceratotherium simum trnS1             GAGAAAGAA-TGCAAGAACTGCTAACTCATG-CCCCCGTACT-TAACAG-TATGGCTCTCTCA
Equus caballus trnS1                  GAGAAAGTA-TGCAAGAACTGCTAATTCATGC-CCCCATGTC-CAACAAACATGGCTCTCTCA
Pecari tajacu trnS1                   GAAAAAGTA-TGCAAGAACTGCTAACTCATGC-CCCCGCACC-TAACAG-TGCGGCTTTTTCA
Phacochoerus africanus trnS1          GAAAAAGT-TTGCAAGAACTGCTAACTCATGC-TCCCACACC-TAAAAA-CGTGGCTTTTTCA
Potamochoerus porcus trnS1            GAAAAAGT-CTGCAAGAACTGCTAACTCATGC-TTCCACACC-TAAAAA-TGTGGCTTTTTCA
Sus scrofa trnS1                      GAAAAAGT-TTGCAAGAACTGCTAACTCATGC-TTCCACACT-TAAAAA-TGTGGCTTTTTCA
Camelus bactrianus trnS1              GAAAAAGCG-TGCAAGAACTGCTAATTCATGC-TACCACACC-TAACAA-TGTGGCTTTTTCA
Camelus dromedarius trnS1             GAAAAAGCA-TGCAAGAACTGCTAATTCATGC-TACCACACC-TAACAA-TGTGGCTTTTTCA
Lama guanicoe trnS1                   GAAAAAGTA-TGCAAGAACTGCTAACTCATGC-CACCATGCC-TAACAG-CATGGCTTTTTCA
Vicugna pacos trnS1                   GAAAAAGTA-TGCAAGAACTGCTAACTCATGC-CACCATGCC-TAACAG-CATGGCTTTTTCA
Hyemoschus aquaticus trnS1            GAGAAAGC-TCGCAAGGACTGCTAATTCTATGCCTCCATGGC-TAATAC-CATGGCTTCCTCA
Tragulus kanchil trnS1                GAAAAAGTA-TGCAAGAACTGCTAATTCTATGCTCCCACGAC-TAACAC-CGTGGCTCTTTCA
Giraffa camelopardalis trnS1          GAAAAAGCG-CACAAGAACTGCTAACTCTGTA-TCCCGTGCC-TAACAG-CACGGCTTTTTCG
Okapia johnstoni trnS1                GAAAAAGCA-TGCAAGAACTGCTAACTCTACA-TCCCGTGTC-TAACAA-CACGGCTTTTTCA
Antilocapra americana trnS1           GAAAAAGTA-TGCAAGAACTGCTAACTCTATGCCCCCATGCC-TAACAA-CATGGCTTTTTCG
Moschus moschiferus trnS1             GAAAAAGTA-CACAAGAACTGCTAATTCTATGTCCCCATGTC-TAACAA-CATGGCTTTTTCA
Muntiacus muntjak trnS1               GAAAAAGTT-CATAGGAACTGCTAATTCCTATAACCCGTGTA-TAATAA-CACGGCTTTTTCG
Capreolus capreolus trnS1             GAAAAAGTA-TGCAAGAACTGCTAACTCTATGCTCCCATATC-TAACAA-TATGGCTTTTTCG
Alces alces trnS1                     GAAAAAGCA-CGCAAGAACTGCTAATTCTATGCCTCCATGTA-TAACAA-CATGGCTTTTTCG
Cervus elaphus trnS1                  GAAAAAGTA-CGCAAGAACTGCTAACTCTATGCCCCCGTGTA-TAATAA-CACGGCTTTTTCG
Dama dama trnS1                       GAAAAAGTA-TGCAAGAACTGCTAACTCTTATGCCCCCGTGCATAATAG-CACGGCTTTTTCG
Boselaphus tragocamelus trnS1         GAAAAAGTA-TGCAAGAACTGCTAACTCTATGCCCCCATGCC-TAACAA-CATGGCTTTTTCA
Tragelaphus oryx trnS1                GAAAAAGTA-TGCAAGAACTGCTAATTCTATG-CCCCATGCC-TAACAA-CATGGCTTTTTCA
Bos taurus trnS1                      GAAAAAGTA-TGCAAGAACTGCTAATTCTATGCTCCCATATC-TAATAG-TATGGCTTTTTCG
Bubalus bubalis trnS1                 GAAAAAGCA-CGCAAGAACTGCTAATTCTATGCCCCCATGCC-TAACAG-TATGGCTTTTTCA
Syncerus caffer trnS1                 GAAAAAGTA-CGCAAGAACTGCTAATTCTATGCCCCCATACC-TAACAG-TATGGCTTTTTCA
Neotragus moschatus trnS1             GAAAAAGTA-TGCAAGAACTGCTAATTCTATGCCCCCATGCT-TAACAA-CATGGCTTTTTCA
Alcelaphus buselaphus trnS1           GAAAAAGTA-TGCAAGAACTGCTAATTCTACGCCCCCATGTC-TAACAA-CATGGCTTTTTCA
Oryx gazella trnS1                    GAAAAAGTA-CGCAAGAACTGCTAATTCTATGCCCCCATGCC-TAACAA-CATGGCTTTTTCA
Pantholops hodgsonii trnS1            GAAAAAGCA-TGCAAGAACTGCTAATTCTATGCTCCCGTGTT-TAACAA-CACGGCTTTTTCG
Ovibos moschatus trnS1                GAAAAAGAA-TGCAAGAACTGCTAATTCCACGCCCCCATGTC-TAACAA-CATGGCTTTTTCA
Capra hircus trnS1                    GAAAAAGTA-TGCAAGAACTGCTAACTCTATGCTTCCATGTC-TAACAA-CATGGCTTTTTCA
Ovis aries trnS1                      GAAAAAGTA-TGCAAGAACTGCTAATTCTATGCCCCCATGTC-TAATAG-CATGGCTTTTTCA
Cephalophus natalensis trnS1          GAAAAAGTA-TACAAGAACTGCTAACTCTATGTCCCCATGCC-TAACAA-CATGGCTTTTTCA
Redunca fulvorufula trnS1             GAAAAAGCA-TGCAAGAACTGCTAACTCTATG-CCCCATGTC-TAACAA-CATGGCTTTTTCA
Ourebia ourebi trnS1                  GAAAAAGTA-CGCAAGAACTGCTAATTCTTACGCCCCGTGTC-TGACAA-CATGGCTTTTTCA
Antilope cervicapra trnS1             GAAAAAGTA-TGCAAGAACTGCTAACTCTGTGCCCCCGTGTC-TAACAA-CACGGCTTTTTCA
Gazella gazella trnS1                 GAAAAAGTA-TGCAAGAACTGCTAACTCTATGCCTCCATGTC-TAACAG-CATGGCTTTTTCA
Procapra gutturosa trnS1              GAAAAAGTA-TGCAAGAACTGCTAACTCTATGCCTCCATGTT-TAACAA-CATGGCTTTTTCA
Raphicerus campestris trnS1           GAAAAAGTACTGCAAGAACTGCTAATTCTATGCTTCCATGTT-TAACAA-CATGGCTTTTTTA
Hexaprotodon liberiensis trnS1        GAGAAAGCA-TGCAAGAACTGCTAATTCATGC-CCCCATATC-TAACAA-TATGGCTTTCCCA
Hippopotamus amphibius trnS1          GAGAAAGCA-TGCAAGAACTGCTAATTCATGC-CCCCATATC-TAACAA-TATGGCTTTCCCA
Eubalaena australis trnS1             GAAAAAGTATTGCAAGAACTGCTAACTCATGC-TCCCACACC-TAACAGCTGTGGCTTTTTCA
Eubalaena japonica trnS1              GAAAAAGTATTGCAAGAACTGCTAACTCATGC-TCCCACACC-TAACAGCTGTGGCTTTTTCA
Balaena mysticetus trnS1              GAAAAAGCATTGCAAGAACTGCTAACTCATGC-TCCCACACC-TAACAACTGTGGCTTTTTCA
Caperea marginata trnS1               GAAAAAGTATTGCAAGAACTGCTAATTCATGC-TCCCACACT-TAACAGTTGTGGCTTTTTCA
Eschrichtius robustus trnS1           GAAAAAGTATCGCAAGAACTGCTAACTCATGC-CCCCACACC-TGATAACTGTGGCTTTTTCA
Balaenoptera acutorostrata trnS1      GAAAAAGTACCGCAAGAACTGCTAATTCATGC-TCCCACACC-TAACAACTGTGGCTTTTTCT
Balaenoptera bonaerensis trnS1        GAAAAAGTACCGCAAGAACTGCTAATTCATGC-TCCCACACC-TAACAACTGTGGCTTTTTCA
Balaenoptera physalus trnS1           GAAAAAGTACTGCAAGAACTGCTAATTCATGC-TCCCACACC-TAACAGCTGTGGCTTTTTCA
Megaptera novaeangliae trnS1          GAAAAAGTATTGCAAGAACTGCTAACTCATGC-TCCCACACC-TGACAACTGTGGCTTTTTCA
Balaenoptera musculus trnS1           GAAAAAGTATCGCAAGAACTGCTAACTCATGC-TCCCACACC-TAACAGCTGTGGCTTTTTCA
Balaenoptera omurai trnS1             GAAAAAGTACTGCAAGAACTGCTAATTCATGC-TCCCACACC-TAACAGCTGTGGCTTTTTCA
Balaenoptera borealis trnS1           GAAAAAGTATAGCAAGAACTGCTAACTCATGC-TTCCACACC-TAACAGCTGTGGCTTTTTCA
Balaenoptera brydei trnS1             GAAAAAGTACAGCAAGAACTGCTAATTCATGC-TTCCACACC-TAACAACTGTGGCTCTTTCA
Balaenoptera edeni trnS1              GAAAAAGTATAGCAAGAACTGCTAATTCATGC-TTCCACACC-TAACAGCTGTGGCTTTTTCA
Kogia breviceps trnS1                 GAGAAAGTACCGCAAGAACTGCTAATTCCCGC-CTCCATGCC-TAATAG-CATGGCTTTCTTA
Physeter macrocephalus trnS1          GAAAAAGTACTGCAAGAACTGCTAACTCACGC-TTCCATATC-TAACAA-TATGGCTTTTTCA
Platanista minor trnS1                GAAAAAGTACTGCAAGAACTGCTAACTCATGC-TTCCATGCC-TAATAG-CATGGCTTTTTCA
Ziphius cavirostris trnS1             GAAAAAGTACTGCAAGAACTGCTAATTCATGC-TCCCACACC-TAACA-CCGTGGCTTTTTCA
Mesoplodon densirostris trnS1         GAAAAAGTACTGCAAGAACTGCTAACTCATGC-TTCCACACC-TAATA-CTGTGGCTTTTTCA
Mesoplodon europaeus trnS1            GAAAAAGTACTGCAAGAACTGCTAACTCATGC-TCCCACACC-TAACA-CTGTGGCTTTTTCA
**Mesoplodon grayi trnS1              GAAAAAGTACTGCAAGAACTGCTAACTCATGC-TCCCACATC-TAACA-CTGTGGCTTTTTCA
Berardius bairdii trnS1               GAAAAAGTACTGCGAGAGCTGCTAACTCACGC-TCCCACACC-TAACAA-TGTGGCTTTTTCA
Hyperoodon ampullatus trnS1           GAAAAAGTACCGCAAGAACTGCTAACTCATGC-TCCCACACC-TAACA-CTGTGGCTTTTTCA
Lipotes vexillifer trnS1              GAAAAAGTAATGCAAGAACTGCTAACTCATGT-TACCACACC-TAATAG-TGTGGCTTTTTCA
Inia geoffrensis trnS1                GAAAAAGCACTGCAAGAACTGCTAATTCATGCTTCCCACACC-TAATAG-TGTGGCTTTTTCA
Pontoporia blainvillei trnS1          GAAAAAGTATCGCAAGAACTGCTAACTCCTGCTTCCCATACT-TAACAG-TATGGCTTTTTCA
Monodon monoceros trnS1               GAAAAAGTATTGCAAGAACTGCTAATTCATGCCCCCCAC-C--TAATAA-TGTGGCTTTTTCA
**Neophocaena_asiaeorientalis trnS1   GAAAAAGTACTGCAAGAACTGCTAATTCATGCCACCCACACC-TAACAA-TGTGGCTTTTTCA
Neophocaena phocaenoides trnS1        GAAAAAGTACTGCAAGAACTGCTAATTCATGCCACCCACACC-TAACAA-TGTGGCTTTTTCA
Phocoena phocoena trnS1               GAAAAAGTACTGCAAGAACTGCTAATTCATGCTCTCCACACC-TAACAA-TGTGGCTTTTTCG
Cephalorhynchus heavisidii trnS1      GAAAAAGAATTGCAAGAACTGCTAATTCATGCACCCCACACT-TAACAA-TGTGGCTTTTTCA
Sousa chinensis trnS1                 GAAAAAGAATTGCAAGAACTGCTAATTCATGTGTTCCACACT-TAACAA-TGTGGCTTTTTCA
Stenella attenuata trnS1              GAAAAAGAACTGCAAGAACTGCTAATTCATGCGTTCCACACT-TAACAA-TGTGGCTTTTTCA
Tursiops australis trnS1              GAAAAAGAATTGCAAGAACTGCTAATTCGTGCGTTCCACACT-TAACAA-TGTGGCTTTTTCA
Tursiops truncatus trnS1              GAAAAAGAATTGCAAGAACTGCTAATTCATGCGTTCCACACT-TAACAA-TGTGGCTTTTTCA
Tursiops aduncus trnS1                GAAAAAGAATTGCAAGAACTGCTAATTCATGCGTTCCGCACT-TAACAA-TGTGGCTTTTTCA
Delphinus capensis trnS1              GAAAAAGAATTGCAAGAACTGCTAATTCATGCGTTCCACACT-TAACAA-TGTGGCTTTTTCA
Stenella coeruleoalba trnS1           GAAAAAGAATTGCAAGAACTGCTAATTCATGCGTTCCGCACT-TAACAA-TGTGGCTTTTTCA
Orcaella brevirostris trnS1           GAAAAAGAATTGCAAGAACTGCTAATTCATGCGCCCCATACT-TAATAA-TATGGCTTTTTCA
Orcaella heinsohni trnS1              GAAAAAGAATTGCAAGAACTGCTAATTCATGCACTCCATACT-TAACAA-TGTGGCTTTTTCA
Grampus griseus trnS1                 GAAAAAGAATTGCAAGAACTGCTAATTCATGCGCTCCACACT-TAACAA-TGCGGCTTTTTCA
Pseudorca crassidens trnS1            GAAAAAGAATTGCAAGAACTGCTAATTCATGCACCCCACACT-TAACAA-TGTGGCTTTTTCA
Feresa attenuata trnS1                GAAAAAGAACTGCAAGAACTGCTAATTCATGCACTCCACACT-TAACAA-TGTGGCTTTTTCA
Peponocephala electra trnS1           GAAAAAGAATTGCAAGAACTGCTAATTCATGTGCTCCACACT-TAACAA-TGTGGCTTTTTCA
Globicephala macrorhynchus trnS1      GAAAAAGAACTGCAAGAACTGCTAATTCATGCCCTCCACACT-TAACAA-TGTGGCTTTTTCA
Globicephala melas trnS1              GAAAAAGAACTGCAAGAACTGCTAATTCATGCCCTCCACACT-CAACAA-TGTGGCTTTTTCA
Lagenorhynchus albirostris trnS1      GGAAAAGAATTGCAAGAACTGCTAATTCATGCGCTCCACACC-TAACAA-TGTGGCTTTTTCA
Orcinus orca WNPTRU1 trnS1            GAAAAAGAACTACAAGAACTGCTAATTCATGTGCTCCACACC-TAACAA-TGTGGCTTTTTCA
Orcinus orca AntA1 trnS1              GAAAAAGAACTACAAGAACTGCTAATTCATGTGCTCCACACC-TAACAA-TGTGGCTTTTTCG
Orcinus orca AntB1 trnS1              GAAAAAGAACTACAAGAACTGCTAATTCATGTGCTCCACACC-TAACAA-TGTGGCTTTTTCA
Orcinus orca AntC1 trnS1              GAAAAAGAACTACAAGAACTGCTAACTCATGTGCTCCACACC-TAACAA-TGTGGCTTTTTCA
Orcinus orca ENAHN1 trnS1             GAAAAAGAACTACAAGAACTGCTAATTCATGTGCTCCACACC-TAACAA-TGTGGCTTTTTCA
Orcinus orca CNPNRAL trnS1            GAAAAAGAACTACAAGAACTGCTAATTCATGTGCTCCACACC-TAACAA-TGTGGCTTTTTCA
Orcinus orca ENPOAL2 trnS1            GAAAAAGAACTACAAGAACTGCTAATTCATGTGCTCCACACC-TAACAA-TGTGGCTTTTTCA
                                                    *             *
                                      1234567     012345 ant  543210    12345          5432176543210d
                                      0000000     111111 222  222233    33334          5555555556666
                                      1234567     345678 123  678901    67890          1234567890123
```

N, the most common base for the position.

N, half compensatory base change in the stem pair (e.g. T – G vs C – G; A-T vs G-T).

N, N, half compensatory base change in the stem pair exhibiting a mismatch (e.g. T–A vs A–A). Different colours are used to better differentiate the changes.

N, fully compensatory base change in the stem pair exhibiting a mismatch (e.g. C–G vs T–T).

N, type I fully compensatory base change in the stem pair (i.e. purine – pyrimidine vs purine – pyrimidine, e.g. G – C vs A – T).

N, N, type II fully compensatory base change in the stem pair (i.e. purine – pyrimidine vs pyrimidine – purine, e.g. A – T vs T – A). Different colours are used to better differentiate the changes.

N, a mismatch in the in the stem pair; N, substitution pattern not modelled; *, pair in the stem in which a mismatch is prominent. N, N, molecular signature for a taxon.

X, position 1-7 in the acceptor stem; XX position (0)1-5 in the anticodon stem; X position 1-5 in the TΨC stem; **ant**, anticodon; **d**, discriminator nucleotide.

trnS2 [SER (S2, UCN)] multiple alignment

```
                                            10        20        30        40        50        60        70
                                             |         |         |         |         |         |         |
                                    1234567 1234     4321 012345  ant  543210    12345       543217654321d
                                                          *                 *
Ceratotherium simum trnS2           GAGAAAGACATAAAGGCTATGATATTGGCTTGAAACCAGTTA-GAGGGGGTTCGATTCCTTCCTTTCTTA
Equus caballus trnS2                GAGAAAGACATAGTGGTTATGATATTGGCTTGAAACCAGTTA-GAGGGGGTTCGATTCCTTCCTTTCTTA
Pecari tajacu trnS2                 GAGAAAGACATAGAGGCTGTGGCGTTGGCTTGAAACCAGTGG-AAGAGGGTTCGATTCCTTCCTTTCTTA
Phacochoerus africanus trnS2        GAGAAAGACATAGTGGCTATGACGTTGGCTTGAAACCAGTGA-GAGAGGGTTCGATTCCTTCCTTTCTTA
Potamochoerus porcus trnS2          GAGAAAGACATAGTGGCTATGGCGTTGGCTTGAAACCAGTGA-AAGAGGGTTCGATTCCTTCCTTTCTTG
Sus scrofa trnS2                    GAGAAAGACATAGTGGTTATGACGTTGGCTTGAAACCAGTGG-GAGAGGGTTCGATTCCTTCCTTTCTTA
Camelus bactrianus trnS2            GAGAAAGTTATAGTGGCTATAGGGTTGGCTTGAAACCAATTA-AAGAGGGTTCGATTCCTTCCTTTCTTA
Camelus dromedarius trnS2           GAGAAAGTTATAGTGGCTATAGGGTTGGCTTGAAACCAATTA-AAGAGGGTTCGATTCCTTCCTTTCTTA
Lama guanicoe trnS2                 GAGAAAGTCATAGTGGCTATGGGGTTGGCTTGAAACCAATTA-GAGAGGGTTCGATTCCTTCCTTTCTTA
Vicugna pacos trnS2                 GAGAAAGTCATAGTGGCTATGGGGTTGGCTTGAAACCAATTA-GAGAGGGTTCGATTCCTTCCTTTCTTA
Hyemoschus aquaticus trnS2          GAGAAAGATATAGTGGCTATAATGTTGGCTTGAAACCGATAG-TTGGGGGTTCGATTCCTTCCTTTCTTA
Tragulus kanchil trnS2              GAGAAAGATACAGTGGCTGTGGTGTTGGCTTGAAACCAATAG-TAGGGGGTTCGATTCCTTCCTTTCTTA
Giraffa camelopardalis trnS2        GAGAAAGACATAGTGGTTATGATGTTGGCTTGAAACCAATAG-TAGGGGGTTCAATTCCTTCCTTTCTTA
Okapia johnstoni trnS2              GAGAAAGATATAGTGGTTATAGTGTTGGCTTGAAACCAGTAA-TAGAGGGTTCGATTCCTTCCTTTCTTA
Antilocapra americana trnS2         GAGAAAGACATAGTGGCTATGATATTGGCTTGAAACCAATTG-TAGGGGGTTCGATTCCTTCCTTTCTTA
Moschus moschiferus trnS2           GAGAAAGACATAATGGTTATGATGTTGGCTTGAAACCAGTAA-TAGGGGGTTCGATTCCTTCCTTTCTTA
Muntiacus muntjak trnS2             GAGAAAGACATAGTGGTTATGATGTTGGCTTGAAACCAGCTA-TGGAGGGTTCGATTCCTTCCTTTCTTA
Capreolus capreolus trnS2           AAGAAAGACATAGTGGTTATGATGTTGGCTTGAAACCAGCTA-TAGGGGGTTCGATTCCTTCCTTTCTTA
Alces alces trnS2                   GAGAAAGACATAATGGTTATGATGTTGGCTTGAAACCAGCTA-TGGGGGGTTCGATTCCTTCCTTTCTTA
Cervus elaphus trnS2                GAGAAAGACATAGTGGTTATGATGTTGGCTTGAAACCAGCTA-TGGGGGGTTCGATTCCTTCCTTTCTTA
Dama dama trnS2                     GAGAAAGACATAGTGGTTATGATGTTGGCTTGAAACCAGCTA-TAGGGGGTTCGATTCCTTCCTTTCTTA
Boselaphus tragocamelus trnS2       GAGAGAGACATAGTGGTTATGATGTTGGCTTGAAACCAATAG-TAGGGGGTTCGATTCCTTCCTTTCTTA
Tragelaphus oryx trnS2              GAGAAAGACATAGTGGTTATGATGTTGGCTTGAAACCAATAA-TAGGGGGTTCGATTCCTTCCTTTCTTA
Bos taurus trnS2                    GAGAGAGACATAGAGGTTATGATGTTGGCTTGAAACCAATAG-TAGGGGGTTCGATTCCTTCCTTTCTTA
Bubalus bubalis trnS2               GAGAGAGACATAGTGGTTATGATGTTGGCTTGAAACCAATAA-CAGGGGGTTCGATTCCTTCCTTTCTTA
Syncerus caffer trnS2               GAGAGAGACATAGTGGTTATGATGTTGGCTTGAAACCAATAG-TAGGGGGTTCGATTCCTTCCTTTCTTA
Neotragus moschatus trnS2           GAGAGAGACATAGTGGTTATGATGTTGGCTTGAAACCAATAGTTGGGGGGTTCGATTCCTTCCTCTCTTA
Alcelaphus buselaphus trnS2         GAGAAAGACATAGTGGTTATGGTGTTGGCTTGAAACCAATAA-TAGGGGGTTCGATTCCTTCCTTTCTTA
Oryx gazella trnS2                  GAGAAAGACATAGTGGTTATGGTGTTGGCTTGAAACCAATTA-TAGGGGGTTCGACTCCTTCCTTTCTTA
Pantholops hodgsonii trnS2          GAGAAAGACATAGTGGTTATGGTGTTGGCTTGAAACCAATAA-TAGGGGGTTCGATTCCTTCCTTTCTTA
Ovibos moschatus trnS2              GAGAGAGACATAGTGGTTATGGTGTTGGCTTGAAACCAATGG-CAGGGGGTTCGATTCCTTCCTTTCTTA
Capra hircus trnS2                  GAGAGAGACATAGTGGTTATGGTGTTGGCTTGAAACCAATAA-TAGGGGGTTCGATTCCTTCCTTTCTTA
Ovis aries trnS2                    GAGAGAGTCATAGTGGCTATGGTGTTGGCTTGAAACCAATAA-TAGGAGGTTCGATTCCTTCCTTTCTTA
Cephalophus natalensis trnS2        GAGAGAGTCATAGTGGTTATGGTGTTGGCTTGAAACCAATAA-TAGGGGGTTCGATTCCTTCCTTTCTTA
Redunca fulvorufula trnS2           GAGAAAGACATGATGGTTATGGTGTTGGCTTGAAACCAATAA-TAGGGGGTTCGATTCCTTCCTTTCTTA
Ourebia ourebi trnS2                GAGAAAGACATAGTGGTTATGGTGTTGGCTTGAAACCAATTT-TAGGGGGTTCGATTCCTTCCTTTCTTA
Antilope cervicapra trnS2           AAGAAAGACATAGTGGTTATGATGTTGGCTTGAAACCAATAA-CAGAGGGTTCGATTCCTTCCTTTCTTA
Gazella gazella trnS2               AAGAAAGACATAGTGGTTATGGTGTTGGCTTGAAACCAATAG-TAGGGGGTTCGATTCCTTCCTTTCTTA
Procapra gutturosa trnS2            GAGAGAGACATAATGGTTATGGTGTTGGCTTGAAACCAATAG-TAGGGGGTTCGATTCCTTCCTTTCTTA
Raphicerus campestris trnS2         GAGAAAGACATAGTGGTTATGGTGTTGGCTTGAAACTAATGT-TAGAGGGTTCGATTCCTTCCTTTCTTA
Hippopotamus amphibius trnS2        GAGAGAGACATAGTGGTTATGATGTTGGCTTGAAACCAACAG-GAGGAGGTTCGATTCCTTCCTCTCTTG
Hexaprotodon liberiensis trnS2      GAGAGAGACATAGTGGTTATGATGTTGGCTTGAAACCAACAG-GAGGAGGTTCGATTCCTTCCTCTCTTG
Eubalaena australis trnS2           AAGAAAGACATAGTGGTTATGATGTTGGCTTGAAACCAATGG-GAGAGGGTTCGATTCCTTCCTTTCTTG
Eubalaena japonica trnS2            AAGAAAGACATAGTGGTTATGATGTTGGCTTGAAACCAATGG-GAGAGGGTTCGATTCCTTCCTTTCTTG
Balaena mysticetus trnS2            AAGAAAGACATAATGGTTATGATATTGGCTTGAAACCAATGG-GAGAGGGTTCGATTCCTTCCTTTCTTG
Caperea marginata trnS2             AAGAAAGACATAGTGGTTATGATGTTGGCTTGAAACCAATGG-GAGAGGGTTCGATTCCTTCCTTTCTTG
Eschrichtius robustus trnS2         AAGAAAGACATAATGGTTATGATGTTGGCTTGAAACCAATGG-GAGAGGGTTCGATTCCTTCCTTTCTTG
Balaenoptera acutorostrata trnS2    AAGAAAGACATAGTGGTTATGATGTTGGCTTGAAACCAATGG-GAGAGGGTTCGATTCCTTCCTTTCTTG
Balaenoptera bonaerensis trnS2      AAGAAAGACATAGTGGTTATGATGTTGGCTTGAAACCAATAG-GAGAGGGTTCGATTCCTTCCTTTCTTG
Balaenoptera physalus trnS2         AAGAAAGACATAGTAATTATGATGTTGGCTTGAAACCAATGG-GAGAGGGTTCGATTCCTTCCTTTCTTG
Megaptera novaeangliae trnS2        AAGAAAGACATAGTGGTTATGATGTTGGCTTGAAACCAATGG-GAGAGGGTTCGATTCCTTCCTTTCTTG
Balaenoptera musculus trnS2         AAGAAAGACATAGTGGTTATGATATTGGCTTGAAACCAATGG-GAGAAGGTTCGATTCCTTCCTTTCTTG
Balaenoptera omurai trnS2           AAGAAAGACATAGTGGTTATGGTGTTGGCTTGAAACCAATAG-GAGAGGGTTCGATTCCTTCCTTTCTTG
Balaenoptera borealis trnS2         AAGAAAGACATAGTGGTTATGATGTTGGCTTGAAACCAATGG-GAGAGGGTTCGATTCCTTCCTTTCTTG
Balaenoptera brydei trnS2           AAGAAAGACATAGTGGTTATGATGTTGGCTTGAAACCAATGG-GAGAGGGTTCGATTCCTTCCTTTCTTG
Balaenoptera edeni trnS2            AAGAAAGACATAGTGGTTATGATGTTGGCTTGAAACCAATGG-GAGAGGGTTCGATTCCTTCCTTTCTTG
Kogia breviceps trnS2               AAGAAAGTCATAGTGGTTATGGTGTTGGCTTGAAACCAACTA-AAGGAGGTTCGATTCCTTCCTTTCTTG
Physeter macrocephalus trnS2        AAGAGAGACATAAAGGTTATGATGTTGGCTTGAAACCAGCTA-GAGAGGGTTCGAGTCCTTCCTTTCTTG
Platanista minor trnS2              AAGAAAGACATAGTGGTTATGGTGTTGGCTTGAAACCAATTT-TAGGGGGTTCGAATCCTTCCTTTCTTG
Ziphius cavirostris trnS2           AAGAAAGACATAATGGTTATGGTGTTGGCTTGAAACCGATTA-TAGAAGGTTCGATTCCTTCCTTTCTTG
Mesoplodon densirostris trnS2       AAGAAAGACATAATGGTTATGGTGTTGGCTTGAAACCAATTA-TAGGAGGTTCGATCCCTTCCTTTCTTG
Mesoplodon europaeus trnS2          AAGAAAGACATAGTGGTTATGATGTTGGCTTGAAACCGATTA-TAGGAGGTTCGATTCCTTCCTTTCTTG
**Mesoplodon grayi trnS2            AAGAAAGACATAATGGTTATGGTGTTGGCTTGAAACCGATTA-TAGGGGGTTCGATTCCTTCCTTTCTTG
Berardius bairdii trnS2             AAGAAAGACATAGTGGTTATGGTATTGGCTTGAAACCAGTTA-CAGGGGGTTCGATTCCTTCCTTTCTTG
Hyperoodon ampullatus trnS2         AAGAAAGACATAATGGTTATGGTGTTGGCTTGAAACCAATTA-TAGGGGGTTCGATTCCTTCCTTTCTTG
Lipotes vexillifer trnS2            AAGAAAGACATAGTGGTTATGGTGTTGGCTTGAAACCAATTG-GAGAGGGTTCGATTCCTTCCTTTCTTG
Inia geoffrensis trnS2              AAGAAAGACATATTGGTTATGGTGTTGGCTTGAAACCAATTG-AAGAGGGTTCGATTCCTTCCTTTCTTG
Pontoporia blainvillei trnS2        AAGAGAGACATGTTGGTTATGGGGTTGGCTTGAAACCAATTA-GAGAAGGTTCGATTCCTTCCTTTCTTG
Monodon monoceros trnS2             AAGAAAGACATAGTGGTTATGATGTTGGCTTGAAACCAATTA-GAGGGGGTTCGATTCCTTCCTTTCTTG
**Neophocaena asiaeorientalis trnS2 AAGAAAGACATAGTGATTATGGTGTTGGCTTGAAACCAATTA-GAGAGGGTTCAATTCCTTCCTTTCTTG
Neophocaena phocaenoides trnS2      AAGAAAGACATAAAGGTTATGCTGTTGGCTTGAAACCGATTA-GAGGGGGTTCGATTCCTTCCTTTCTTG
Phocoena phocoena trnS2             AAGAAAGACATAAAGGTTATGCTGTTGGCTTGAAACCGACTA-GAGAGGGTTCGACTCCTTCCTTTCTTG
Cephalorhynchus heavisidii trnS2    AAGAAAGACATAATGGTTATGATGTTGGCTTGAAACCGATTA-GAGGGGGTTCGATTCCTTCCTTTCTTG
Sousa chinensis trnS2               AAGAAAGACATAATGGTTATGATGTTGGCTTGAAACCGATTA-GAGGAGGTTCGATTCCTTCCTTTCTTG
Stenella attenuata trnS2            AAGAAAGACATAATGGTTATGATGTTGGCTTGAAACCGATTA-GAGGGGGTTCGATTCCTTCCTTTCTTG
Tursiops australis trnS2            AAGAAAGACATAATGGTTATGATGTTGGCTTGAAACCAATTA-GAGGGGGTTCGATTCCTTCCTTTCTTG
Tursiops truncatus trnS2            AAGAAAGACATAATGGTTATGATGTTGGCTTGAAACCGATTA-GAGGGGGTTCGATTCCTTCCTTTCTTG
Tursiops aduncus trnS2              AAGAAAGACATAATGGTTATGATGTTGGCTTGAAACCGATTA-GAGGGGGTTCGATTCCTTCCTTTCTTG
Delphinus capensis trnS2            AAGAAAGACATAATGGTTATGATGTTGGCTTGAAACCGATTA-GAGGGGGTTCGATTCCTTCCTTTCTTG
Stenella coeruleoalba trnS2         AAGAAAGACATAATGGTTATGATGTTGGCTTGAAACCGATTA-GAGGGGGTTCGATTCCTTCCTTTCTTG
Orcaella brevirostris trnS2         AAGAAAGACATAATGGTTATGATGTTGGCTTGAAACCGATTA-GAGGGGGTTCGATTCCTTCCTTTCTTG
Orcaella heinsohni trnS2            AAGAAAGACATAATGGTTATGATGTTGGCTTGAAACCGATTA-GAGGGGGTTCGATTCCTTCCTTTCTTG
Grampus griseus trnS2               AAGAAAGACATAATGGTTATGCTGTTGGCTTGAAACCGATTA-GAGGGGGTTCGATTCCTTCCTTTCTTG
Pseudorca crassidens trnS2          AAGAAAGACATAGTGGTTATGATGTTGGCTTGAAACCGATTA-GAGGGGGTTCGATTCCTTCCTTTCTTG
Feresa attenuata trnS2              AAGAAAGACATAATGGTTATGATGTTGGCTTGAAACCGATTA-GAGGGGGTTCGATTCCTTCCTTTCTTG
Peponocephala electra trnS2         AAGAAAGACATAATGGTTATGATGTTGGCTTGAAACCGATTA-GAGGGGGTTCGATTCCTTCCTTTCTTG
Globicephala macrorhynchus trnS2    AAGAAAGACATAATGGTTATGATGTTGGCTTGAAACCGATTA-GAGGGGGTTCGATTCCTTCCTTTCTTG
Globicephala melas trnS2            AAGAAAGACATAATGGTTATGATGTTGGCTTGAAACCAATTA-GAGGGGGTTCGATTCCTTCCTTTCTTG
Lagenorhynchus albirostris trnS2    AAGAAAGACATAGTGGTTATGATATTGGCTTGAAACCGATTA-GAGGGGGTTCGATTCCTTCCTTTCTTG
Orcinus orca WNPTRU1 trnS2          AAGAAAGACACAATGGCTGTGATATTGGCTTGAAACCGATTA-GAGGGGGTTCGATTCCTTCCTTTCTTG
Orcinus orca AntA1 trnS2            AAGAAAGACACAATGGCTGTGATATTGGCTTGAAACCGATTA-GAGGGGGTTCGATTCCTTCCTTTCTTG
Orcinus orca AntB1 trnS2            AAGAAAGACACAATGGCTGTGATATTGGCTTGAAACCGATTA-GAGGGGGTTCGATTCCTTCCTTTCTTG
Orcinus orca AntC1 trnS2            AAGAAAGACACAATGGCTGTGATATTGGCTTGAAACCGATTA-GAGGGGGTTCGATTCCTTCCTTTCTTG
Orcinus orca ENAHN1 trnS2           AAGAAAGACACAATGGCTGTGATATTGGCTTGAAACCGACTA-GAGGGGGTTCGATTCCTTCCTTTCTTG
Orcinus orca CNPNRAL trnS2          AAGAAAGACACAATGGCTGTGATATTGGCTTGAAACCGATTA-GAGGGGGTTCGATTCCTTCCTTTCTTG
Orcinus orca ENPOAL2 trnS2          AAGAAAGACACAATGGCTGTGATATTGGCTTGAAACCGATTA-GAGGGGGTTCGATTCCTTCCTTTCTTG
                                                          *                 *
                                    1234567 1234     4321 012345  ant  543210    12345       543217654321d
                                    0000000 9111     1122 222222  333  333344    44445       5566666666667
                                    1234567 0012     8901 345678  123  678901    67890       8901234567890
```

N, the most common base for the position.
N, half compensatory base change in the stem pair (e.g. T – G vs C – G; A-T vs G-T).
N, N, half compensatory base change in the stem pair exhibiting a mismatch (e.g. T–A vs A–A). Different colours are used to better differentiate the changes.
N, fully compensatory base change in the stem pair exhibiting a mismatch (e.g. C–G vs T–T).
N, type I fully compensatory base change in the stem pair (i.e. purine – pyrimidine vs purine – pyrimidine, e.g. G – C vs A – T).
N, N, type II fully compensatory base change in the stem pair (i.e. purine – pyrimidine vs pyrimidine – purine, e.g. A – T vs T – A). Different colours are used to better differentiate the changes.
N, a mismatch in the in the stem pair; N, substitution pattern not modelled; *, pair in the stem in which a mismatch is prominent. N, N, molecular signature for a taxon.
X, position 1-7 in the acceptor stem; X position 1-4 in the DHU stem; XX position (0)1-5 in the anticodon stem; X position 1-5 in the TΨC stem; **ant**, anticodon; **d**, discriminator nucleotide.

trnT (THR) multiple alignment

```
                                         10        20        30        40        50        60        70
                                          |         |         |         |         |         |         |
                                  1234567  1234      4321 12345  ant  54321    1234          43217654321d
                                       *                                                          *
Ceratotherium simum trnT          GTCTTTGTAGTATATG---AATTACTCTGGTCTTGTAAACCAGAAAAGGAAAGC-A-CCAC-TTTCCCCAAGACT
Equus caballus trnT               GTCCCTGTAGTATATCGCACATTACCCTGGTCTTGTAAACCAGAAAAGGGGGAAACGTTT-CCTCCCAAGGACT
Pecari tajacu trnT                GTCTTAGTAGTATATTT--AAATACACTGGTCTTGTAAACCAAAGACGGAGGATAA--CTC-CCTCCCCAAGACT
Phacochoerus africanus trnT       GTCTTTGTAGTATATA---AAATACCCTGGTCTTGTAAACCAGAAAAGGAGAAT----CACTCCTCCCCAAGACT
Potamochoerus porcus trnT         GTCTTTGTAGTATATC---AAATACCCTGGTCTTGTAAACCAGAAAAGGAGAATGA--C-T-CCTCCCTAAGACT
Sus scrofa trnT                   GTCTTCGTAGTATATA---AAATACCCTGGTCTTGTAAACCAGAAAAGGAGGGC----CACCCCTCCCCAAGACT
Camelus bactrianus trnT           GTCTTTGTAGTATACT---GATTACGCTGGTCTTGTAAACCATAAAAGGAGAGCCA--CAC-TCTCCCTAAGACT
Camelus dromedarius trnT          GTCTTTGTAGTATACT---GATTACACTGGTCTTGTAAACCATAGAAGGAGGGCCA--CAC-CCTCCCTAAGACT
Lama guanicoe trnT                GTCTTCGTAGTATATT---GATTACGCTGGTCTTGTAAGCCATCAAAGGAGAATCG--TAC-CCTCCCTAAGACT
Vicugna pacos trnT                GTCTTCGTAGTATATT---GATTACACTGGTCTTGTAAGCCATCAAAGGAGAATCG--TAC-CCTCCCTAAGACT
Hyemoschus aquaticus trnT         GTCTTTGTAGTATATT---AATTACCCTGGTCTTGTAAACCAGAAAAGAAGTATAA-CTAACCTTCCTAAGACT
Tragulus kanchil trnT             GTCTTTGTAGTATATC---CATTACCCTGGTCTTGTAAACCAGAAAAGAAGTATCA-CTACCCTTCCTAAGACT
Giraffa camelopardalis trnT       GTCTTTGTAGTATACT---AAATACATTGGTCTTGTAAGCCAAAAAGGAGTACAA-CCA-ATCTCCCTAAGACT
Okapia johnstoni trnT             GTCTTTGTAGTACACT---AAATACGCTGGTCTTGTAAACCAGAAAAGGAGTACAA-CCA-ACCTCCCTAAGACT
Antilocapra americana trnT        GTCTTTGTAGTATATT---AAATACGCTGGTCTTGTAAACCAGAAAAGGAGAACGA-CCAC-CCTCCCCAAGACT
Moschus moschiferus trnT          GTCTTTGTAGTACAAT-ATAATACACTGGTCTTGTAAACCAGAAAAGGAGAACAA-CTA-ACCTCCCTAAGACT
Muntiacus muntjak trnT            GTCTTTGTAGTATACT---CAATACACTGGTCTTGTAAACCAGAAAAGGAGAACAA-CCA-ACCTCCCTAAGACT
Capreolus capreolus trnT          GTCTTTGTAGTAAATT---CAATACACTGGTCTTGTAAACCAGAAAAGGAGAACTA-CTA-ATCTCCCTAAGACT
Alces alces trnT                  GTCTTTGTAGTACATT---TAATACACTGGTCTTGTAAACCAGAAAAGGAGTACAA-CCA-ACCTCCCTAAGACT
Cervus elaphus trnT               GTCTTTGTAGTATACT---CAATACACTGGTCTTGTAAACCAGAAAAGGAGAGCAA-CTA-ACCTCCCTAAGACT
Dama dama trnT                    GTCTTTGTAGTATACT---CAATACACTGGTCTTGTAAATCAGAAAAGGAGAACAA-TCA-ACCTCCCTAAGACT
Boselaphus tragocamelus trnT      GTCTTTGTAGTATACT---AAATACACTGGTCTTGTAAACCAGAAAAGGAGAACAA-CCA-ACCTCCCCAAGACT
Tragelaphus oryx trnT             GTCTTTGTAGTATATT---AAATACGCTGGTCTTGTAAACCAATAAAGGAGAACAA-CTA-ATCTCCCCAAGACT
Bos taurus trnT                   GTCTTTGTAGTACATCT---AATATACTGGTCTTGTAAACCAGAGAAGGAGAACAA-CTA-ACCTCCCTAAGACT
Bubalus bubalis trnT              GTCTTTGTAGTATATT---AAATACACTGGTCTTGTAAACCAGAAAAGGAGAACAA-CCA-ACCTCCCCAAGACT
Syncerus caffer trnT              GTCTTTGTAGTATATT---AAATACACTGGTCTTGTAAACCAGAAAAGGAGAACAA-CCA-ACCTCCCCAAGACT
Neotragus moschatus trnT          GTCTTCGTAGTATATGG---AATATACTGGTCTTGTAAACCAGAGAAGGAGAGTAA-TTG-TCCTCCCCAAGGCT
Alcelaphus buselaphus trnT        GTCTCTGTAGTACATT---AAATATACTGGTCTTGTAAACCAGAGAAGGAGAACAA-CCA-ACCTCCCTGAGACT
Oryx gazella trnT                 GTCTTTGTAGTATATTT---AATACATTGGTCTTGTAAACCAAAAAGGAGAACAA-CCAACCCCCCCTAAGACT
Pantholops hodgsonii trnT         GTCTTTGTAGTATAAT---AAATACCCTGGTCTTGTAAACCAGAGAAGGAGAACTCACTA-ACCTCCCTAAGACT
Ovibos moschatus trnT             GTCTTTGTAGTACAAC---TAGTATACTGGTCTTGTAAACCAGAGAAGGAGAACAA-CCA-GCCTCCCCAAGACT
Capra hircus trnT                 GTCTTTGTAGTACAAT---CAATACACTGGTCTTGTAAACCAGAAAAGGAGAATAG-CCA-ATCTCCCTAAGACT
Ovis aries trnT                   GTCTTTGTAGTACAAT---CAATATACTGGTCTTGTAAACCAGAGAAGGAGAACAA-CCA-ACCTCCCTAAGACT
Cephalophus natalensis trnT       GTCTTTGTAGTACATT---AAATACACTGGTCTTGTAAACCAGAAAAGGAGAACAA-CCA-ACCTCCCTAAGACT
Redunca fulvorufula trnT          GTCTTTGTAGTATATT---AAATACACTGGTCTTGTAAACCAGAAAAGGAGCATAA-CAA-ACCTCCCTAAGACT
Ourebia ourebi trnT               GTCTTTGTAGTACATT---TAATACACTGGTCTTGTAAACCAGAAAAGGAGAACAA-CTA-ACCTCCCTAAGACT
Antilope cervicapra trnT          GTCTTTGTAGTACATT---AAATATACTGGTCTTGTAAACCAGAGAAGGAGAACAA-GCA-ACCTCCCTAAGACT
Gazella gazella trnT              GTCTTTGTAGTACATT---TAGTATACTGGTCTTGTAAACCAGAGAAGGAGAACAA-GCA-ACCTCCCTAGGACT
Procapra gutturosa trnT           GTCTTTGTAGTATATC---AAATACACTGGCCTTGTAAACCAGAAAAGGAGAACAAACCA-ACCTCCCTAAGACT
Raphicerus campestris trnT        GTCTTTGTAGTACACT---AAGTATACTGGTCTTGTAAACCAGAGATGGAGAACAA---ATACCTCCCTGAGACT
Hexaprotodon liberiensis trnT     GTCTCTGTAGTATATGG--CATTACCTCGGTCTTGTAAACCGAAAAAGGAAG-CAA--CACACCTCCCTGAGACT
Hippopotamus amphibius trnT       GTCTCTGTAGTATATGA--CATTACCCCGGTCTTGTAAGCCGAAAAAGGAAG-CGA--CACACCTCCCTGAGACT
Eubalaena australis trnT          GTCTTTGTAGTATAATT--AAATACCCCGGCCTTGTAAACCGGAAAAGGAGA-CAAACCACATCTCCCTAAGACT
Eubalaena japonica trnT           GTCTTTGTAGTATAATT--AAATACCCCGGCCTTGTAAACCGGAAAAGGAGA-CGAACCACATCTCCCTAAGACT
Balaena mysticetus trnT           GTCTTTGTAGTATAATT--GAATACCCCGGCCTTGTAAACCGGAAAAGGAGA-CAAACCACATCTCCCTAAGACT
Caperea marginata trnT            GTCTTTGTAGTATAATC--AAATACCCCGGTTTTGTAAACCGAAGAAGGAGA-TAAACCACACCTCCCTAAGACT
Eschrichtius robustus trnT        GTCTTTGTAGTATAACT--AAATACCCCGGTTTTGTAAACCGGAAAAGGAGA-CAAACCACACCTCCCTAAGACT
Balaenoptera acutorostrata trnT   GTCTTTGTAGTATAACT--AATTACCCCGGTCTTGTAAACCGGAAAAGGAGAGCGAACCACACCTCCCTAAGACT
Balaenoptera bonaerensis trnT     GTCTTTGTAGTATAATT--AATTACCCCGGTCTTGTAAACCGGAAAAGGAGAGCAAACCACACCTCCCTAAGACT
Balaenoptera physalus trnT        GTCTTTGTAGTATAATT--AAATACCCCGGTTTTGTAAACCGGAAAAGGAGA-CAAGACACACCTCCCTAAGACT
Megaptera novaeangliae trnT       GTCTTTGTAGTATAATT--AAATACCCCGGTTTTGTAAACCGGAAAAGGAGA-CAAACCACACCTCCCTAAGACT
Balaenoptera musculus trnT        GTCTTTGTAGTATAATT--AAATACCTCGGTTTTGTAAACCGAAGAAGGAGA-CAAACCACACCTCCCTAAGACT
Balaenoptera omurai trnT          GTCTTTGTAGTATA-TT--AAATACCCCGGTTTTGTAAACCGAAAAAGGAGA-CA-GCCATACCTCCCTAAGACT
Balaenoptera borealis trnT        GTCTTTGTAGTATAACT--AAATACCCCGGTTTTGTAAACCGGAGAAGGAGA-CAAACCACACCTCCCTAAGACT
Balaenoptera brydei trnT          GTCTTTGTAGTATAACT--AAATATCCCGGTTTTGTAAACCGGAGAAGGAGA-CAAACCACACCTCCCTAAGACT
Balaenoptera edeni trnT           GTCTTTGTAGTATAACT--AAATACCCCGGTTTTGTAAACCGGAGAAGGAGA-CAAACCACACCTCCCTAAGACT
Kogia breviceps trnT              GTCTTTGTAGTATAAT---AAATACCCCGGCCTTGTAAACCGGAAAAGGAGA--AAATTACACCTCCCCAAGACT
Physeter macrocephalus trnT       GTCTTCGTAGTATAAC---AAATACCCCGGCCTTGTAAACCGGAAAAGGAGA--GAGTTACGCCTCCCTAAGACT
Platanista minor trnT             GTCCTCGTAGTATAACG--AAATACCCTGGTTTTGTAAACCAAAAACGGAGG--AAACTACACCTCCCTAGGACT
Ziphius cavirostris trnT          GTCTTTGTAGTATAACA--AAATACCCCGGCTTTGTAAACCGAAAATGGAGA--AAACCACACCTCCCTAAGACT
Mesoplodon densirostris trnT      GTCTTTGTAGTATAATA--AAATACTTCGGTTTTGTAAACCGAAAACGGAGA--AAACTACGCCTCCCTAAGACT
Mesoplodon europaeus trnT         GTCTTTGTAGTATAATA--AAATACCCCGGTTTTGTAAACCGAAAACGGAGA--AAACTACACCTCCCTAAGACT
**Mesoplodon grayi trnT           GTCTTTGTAGTATAACA--AAATACTTCGGTTTTGTAAACCGAAAATGGAGA--AAATTATACCTCCCTAAGACT
Berardius bairdii trnT            GTCTTTGTAGTATAACA--AAATACCCCGACCTTGTAAATCGGAAAAGGAGA--AAACCATATCTCCCTAAGACT
Hyperoodon ampullatus trnT        GTCTTTGTAGTATAACA--AAATACCCCGGCTTTGTAAACCGGAAACGGAAG--AAACTACACTTCCCTAAGACT
Lipotes vexillifer trnT           GTCTTTGTAGTATAAT---AAATACCCCGGTCTTGTAAACCGAAAAAGGAGA--AACCTACTCCTCCCTAAGACT
Inia geoffrensis trnT             GTCTTCGTAGTATAATA--AAATACCCCGGTCTTGTAAACCGGAAAAGGAGA--AATATACCCCTCCCTGAGACT
Pontoporia blainvillei trnT       GTCTTCGTAGTATAACA--AAATACACTGGTCTTGTAAACCAAAATGGAGA--ATAA-AC-CCTCCCCAAGACT
Monodon monoceros trnT            GTCTTCGTAGTATAATA--AAATACCCCGGTCTTGTAAACCGGAAAAGGAGA--ACCC-ATCTCTCCCTAAGACT
**Neophocaena asiaeorientalis trnT GTCTTTGTAGTATA-TA--AAATACTTCGGTCTTGTAAACCGAAAAAGGAGA--ACCCCACTCCTCCCTAAGACT
Neophocaena phocaenoides trnT     GTCTTTGTAGTATA-TA--AAATACTTCGGTCTTGTAAACCGAAAAAGGAGA--ACCCCACTCCTCCCTAAGACT
Phocoena phocoena trnT            GTCTTTGTAGTATA-TA--AAATACCTCGGTCTTGTAAACCGAAAAAGGAGA--ACCTCACTCCTCCCTAAGACT
Cephalorhynchus heavisidii trnT   GTCTTTGTAGTATAACA--AAATACCCCGGTCTTGTAAACCGGAAAAGGAGA--ACCCCATTCCTCCCTAAGACT
Sousa chinensis trnT              GTCTTTGTAGTATAACA--AAATACCCCGGTCTTGTAAACCGGAAAAGGAGA--ACCCCATTCCTCCCTAAGACT
Stenella attenuata trnT           GTCTTTGTAGTATAACA--AAATACCTCGGTCTTGTAAACCGGAAAAGGAGA--ATCCCATTCCTCCCTAAGACT
Tursiops australis trnT           GTCTTTGTAGTATAATA--AAATACCCTGGTCTTGTAAACCGGAAAAGGAGA--ACCCCATTCCTCCCTAAGACT
Tursiops truncatus trnT           GTCTTTGTAGTATAACA--AAATACCTCGGTCTTGTAAACCGGAAAAGGAGA--ACCCCATTCCTCCCTAAGACT
Tursiops aduncus trnT             GTCTTTGTAGTATAACA--AAATACCCCGGTCTTGTAAACCGGAAAAGGAGA--ACTCCATTCCTCCCTAAGACT
Delphinus capensis trnT           GTCTTTGTAGTATAACA--AAATACCCCGGTCTTGTAAACCGGAAAAGGAGA--ACCCCATTCCTCCCTAAGACT
Stenella coeruleoalba trnT        GTCTTTGTAGTATAACA--AAATACCTCGGTCTTGTAAACCGGAAAAGGAGA--ACCCCATTCCTCCCTAAGACT
Orcaella brevirostris trnT        GTCTTTGTAGTATAACA--AAATACCCTGGCCTTGTAAACCGGAAAAGGAGG--ACCCCATTCCTCCCTAAGACT
Orcaella heinsohni trnT           GTCTTTGTAGTATAACA--AAATACCCCGGTCTTGTAAACCGGAAAAGGAGA--ACCCCATTCCTCCCTAAGACT
Grampus griseus trnT              GTCTTTGTAGTATAACA--AAATACCTCGGTCTTGTAAACCGGAAAAGGAGA--ACCCCA-TCCTCCCTAAGACT
Pseudorca crassidens trnT         GTCTTTGTAGTATAACA--AAATACCCCGGTCTTGTAAACCGGAAAAGGAGA--ACCCCA-TCCTCCCTAAGACT
Feresa attenuata trnT             GTCTTTGTAGTATAACA--AAATACCCCGGTCTTGTAAACCGGAAAAGGAGA--ACCCCA-TCCTCCCTAAGACT
Peponocephala electra trnT        GTCTTTGTAGTATAACA--AAATACCCCGGTCTTGTAAACCGGAAAAGGAGG--ACT-CA-TCCTCCCTAAGACT
Globicephala macrorhynchus trnT   GTCTTTGTAGTATAACA--AAATACCCCGGTCTTGTAAACCGGAAAAGGAGA--ACCCCAC-CCTCCCCAAGACT
Globicephala melas trnT           GTCTTTGTAGTATAACA--AAATACCCCGGTCTTGTAAACCGGAAAAGGAGA--ACCCCAC-CCTCCCCAAGACT
Lagenorhynchus albirostris trnT   GTCTTTGTAGTATAACA--AAATACCCCGGCCTTGTAAACCGGAAAAGGAGA--ATCCCATTCCTCCCTAAGACT
Orcinus orca WNPTRU1 trnT         GTCTTTGTAGTATAATA--AAATACCCCGGTCTTGTAAACCGGAAAAGGAGA--ACCCTACTCCTCCCTAAGACT
Orcinus orca AntA1 trnT           GTCTTTGTAGTATAATA--AAATACCCCGGTCTTGTAAACCGGAAAAGGAGA--ACCCTACTCCTCCCTAAGACT
Orcinus orca AntB1 trnT           GTCTTTGTAGTATAATA--AAATACCCCGGTCTTGTAAACCGGAAAAGGAGA--ACCCTACTCCTCCCTAAGACT
Orcinus orca AntC1 trnT           GTCTTTGTAGTATAATA--AAATACCCCGGTCTTGTAAACCGGAAAAGGAGA--ACCCTACTCCTCCCTAAGACT
Orcinus orca ENAHN1 trnT          GTCTTTGTAGTATAATA--AAATACCCCGGTCTTGTAAACCGGAAAAGGAGA--ACCCTACTCCTCCCTAAGACT
Orcinus orca CNPNRAL trnT         GTCTTTGTAGTATAATA--AAATACCCCGGTCTTGTAAACCGGAAAAGGAGA--ACCCTACTCCTCCCTAAGACT
Orcinus orca ENPOAL2 trnT         GTCTTTGTAGTATAATA--AAATACCCCGGTCTTGTAAACCGGAAAAGGAGA--ACCCTACTCCTCCCTAAGACT
                                       *                                                          *
                                  1234567  1234      4321 12345  ant  54321    1234          43217654321d
                                  0000000  1111      2222 22233  333  34444    4455          66666677777 7
                                  1234567  0123      2345 78901  456  90123    8901          45678901234 5
```

N, the most common base for the position.
N, half compensatory base change in the stem pair (e.g. T – G vs C – G; A-T vs G-T).
N, N, half compensatory base change in the stem pair exhibiting a mismatch (e.g. T–A vs A–A). Different colours are used to better differentiate the changes.
N, fully compensatory base change in the stem pair exhibiting a mismatch (e.g. C–G vs T–T).
N, type I fully compensatory base change in the stem pair (i.e. purine – pyrimidine vs purine – pyrimidine, e.g. G – C vs A – T).
N, N, type II fully compensatory base change in the stem pair (i.e. purine – pyrimidine vs pyrimidine – purine, e.g. A – T vs T – A). Different colours are used to better differentiate the changes.
N, a mismatch in the in the stem pair; N, substitution pattern not modelled; *, pair in the stem in which a mismatch is prominent. N, N, molecular signature for a determined taxa.
X, position 1-7 in the acceptor stem; X position 1-4 in the DHU stem; X position 1-5 in the anticodon stem; X position 1-4 in the TΨC stem; ant, anticodon; d, discriminator nucleotide.

trnV (VAL) multiple alignment

```
                                            10        20        30        40        50        60        70
                                             |         |         |         |         |         |         |
                                    1234567  1234       4321 12345  ant  54321    1234        43217654321d
                                                                                 *             *
Ceratotherium simum trnV            CAAAG GTAGCTTAAA--CAAAGCACCTAGC TACACCCAGGAGATTTCATACAA-AATGACCACTTTGA
Equus caballus trnV                 CAAAG GTAGCTTAAA--CAAAGCATCCAGC TACACCTAGAAGATTTCACTCAA-AATGAACACTTTGA
Pecari tajacu trnV                  CAAAGTATAGCTTAAAT-AAAAGCATCTAGTTTACACCCAGAAGACCTCACCAAG-TATGAGTACTTTGA
Phacochoerus africanus trnV         CAAAGTATAGCTTAAAC-TAAAGCACCTAGTTTACACCTAGAAGATCCCAC-AACACATGAGTACTTTGA
Potamochoerus porcus trnV           CAAAGTATAGCCTAAAA-TAAAGTACCTAGC TACACCTAGGAGATCCCAC-AACGTATGGGTACTTTGA
Sus scrofa trnV                     CAAAGCATAGCTTAAAC-TAAAGCACCTAGTTTACACCTAGAAGATCCCAC-AATGTATGGGTACTTTGA
Camelus bactrianus trnV             CAAAACGTAGCTTAAA--AAAAGTACCTAATTTACACTTAGGAGATTTCGT-GGA-AATGAACGTTTTGA
Camelus dromedarius trnV            CAAAACGTAGCTTAAA--AAAAGTACCTAATTTACACTTAGGAGATTTCAT-AGA-AATGAACGTTTTGA
Lama guanicoe trnV                  CAAAACGTAGCTTAAG--AAAAGCACCTAGTTTACACCTAGGAGATTTCAT-AAA-AATGAACGTGTTGA
Vicugna pacos trnV                  CAAAACGTAGCTTAAG--AAAAGCACCTAGTTTACACCTAGGAGATTTCAT-AAA-AATGAACGTGTTGA
Hyemoschus aquaticus trnV           CAAAGCATAGCTTAAA--CAAAGCATCTAGTTTACACCTAGAAGATTTCAT-GAGCCATGAGTGCCTTGA
Tragulus kanchil trnV               CAAAGTATAGCTTAAA--CAAAGCATCTAGTTTACACCTAGAAGATTTCAT-ACTTTATGAATGCCTTGA
Giraffa camelopardalis trnV         CAAGGTATAGCTTAAA--CAAAGCACCTAGTTTACACCTAGAAGATTTCAC-ATATCATGAATACCCTGA
Okapia johnstoni trnV               CAAGACATAGCTTAAA--CAAAGCACCTAGTTTACACCTAGAAGATCTCGCACGCCAACGAGTATCTTGA
Antilocapra americana trnV          CAAGACATAGCTTAAA--CAAAGCACCTAGTTTACACCTAGAAGATTTCAC-GCATAACGAATGTCTTGA
Moschus moschiferus trnV            CAAGATATAGCTTAAA--CAAAGCACCTAGTTTACACCTAGAAGATTTCAT-ATATCACGAATATCTTGA
Muntiacus muntjak trnV              CAAGATATAGCTTAAA--TAAAGCACCTAGTTTACACCTAGAAGATTTCACTACACCACGAATATCTTGA
Capreolus capreolus trnV            CAAGATATAGCTTAAAC-TAAAGCACCTAGTTTACACCTAGAAGATTTCAT-ACACCATGAATACCTTGA
Alces alces trnV                    CAAGATATAGCTTAAAC-TAAAGCATCTAGTTTACACCTAGAAGATTTCAC-CTATCATGAATATCTTGA
Cervus elaphus trnV                 CAAGATATAGCTTAAA--CAAAGCATCTAGTTTACACCTAGAAGATTTCAT-ATATCATGAATATCTTGA
Dama dama trnV                      CAAGATATAGCTTAAA--CAAAGCGCCTAGTCTACACCTAGAAGATTTCAC-ATATTATGAATATCTTGA
Boselaphus tragocamelus trnV        CAAGATATAGCTTAAA--CAAAGCATCTAGTTTACACCTAGAAGATTTCAC-ATACTATGAATGTCTTGA
Tragelaphus oryx trnV               CAAGATATAGCTTAAA--TAAAGCATCTAGTTTACACCTAGAAGATTTTAC-ACACCATGAATATCTTGA
Bos taurus trnV                     CAAGATATAGCTTAAA--CAAAGCATCCAGTTTACACCTAGAAGACTTCAT-TCATTATGAATATCTTGA
Bubalus bubalis trnV                CAAGATATAGCTTAAA--TAAAGCATCCAGTTTACACCTAGAAGATTTCAC-ACACCATGAATATCTTGA
Syncerus caffer trnV                CAAGACATAGCTTAAA--TAAAGTATCCAGTTTACACCTAGAAGATTTCAT-ATACTATGAATGTCTTGA
Neotragus moschatus trnV            CAAGGCATAGCTTAAA--TAAAGCACTTAGTTTACACCTAAGAGATTTCAT-ACACTATGAATGCCTTGA
Alcelaphus buselaphus trnV          CAAGATATAGCTTAAA--CAAAGCATCTAGTTTACACCTAGAAGATTTCAC-ACATCATGAATGTCTTGA
Oryx gazella trnV                   CAAAACATAGCTTAAA--CAAAGCATCTAGTTTACACCTAGAAGATTTCAC-ATATCATGAATGTTTTGA
Pantholops hodgsonii trnV           CAAGACATAGCTTAA-C-TAAAGCATTTAGTTTACACCTAAAAGATTTCAC-ACACTATGAATGTCTTGA
Ovibos moschatus trnV               CAAGATATAGCTTAA-C-TAAAGCATCTAGTTTACACCTAGAAGATTTCAC-ATGTCATGAATATCTTGA
Capra hircus trnV                   CAAGATATAGCTTAA-C-CAAAGCACCTAGTTTACACCTAGAAGATTTCAC-ATATTATGAATATCTTGA
Ovis aries trnV                     CAAGATATAGCTTAA-T-TAAAGCATCTAGTTTACACCTAGAAGATTTCAC-ACATTATGAGTATCTTGA
Cephalophus natalensis trnV         CAAGATATAGCTTAAA--TAAAGCACCTAGTTTACACCTAGAAGATTTCAC-ACACCATGAATATCTTGA
Redunca fulvorufula trnV            CAAGATATAGCTTAAA--TAAAGCACCTAGTTTACACCTAGAAGATTTCAC-ACTCCATGAATATCTTGA
Ourebia ourebi trnV                 CAAGATATAGCTTAAA--TAAAGCATCTAGTTTACACCTAGGAGATTTTAC-ACACCATGAATATCTTGA
Antilope cervicapra trnV            CAAGACATAGCTTAAA--TAAAGCATCTAGTTTACACCTAGAAGATTCCAT-ACACTATGAATGTCTTGA
Gazella gazella trnV                CAAGACATAGCTTAAA--CAAAGCATCTAGTTTACACCTAGAAGATTCCAT-ACACTATGAATGTCTTGA
Procapra gutturosa trnV             CAAGATATAGCTTAAA--GAAAGCATCTAGTTTACACCTAGAAGATTCCAT-ATACCATGAATATCTTGA
Raphicerus campestris trnV          CAAGACATAGCTTAAA--TAAAGCATCTAGTTTACACCTAGAAGATTTCAT-ACACTATGAATGTCTTGA
Hexaprotodon liberiensis trnV       CAAAGCATAGCTTAG CCCAAAGCATCTAGTTTACACCTAGAAGATTTCAC-AATAAGTGAATGCCTTGA
Hippopotamus amphibius trnV         CAAAGCATAGCTTAG TTTAAAGCATCTAGTTTACACCCAGAAGATTTCAC-AATAAGTGAATGCCTTGA
Eubalaena australis trnV            CAAGATATAGCTTAAA--CAAAGCATCTAGTTTACACCTAGAAGATTCCAC-ACTCTGTGTATATCTTGA
Eubalaena japonica trnV             CAAGATATAGCTTAAA--CAAAGCATCTAGTTTACACCTAGAAGATTTCAC-ACTCTGTGTATATCTTGA
Balaena mysticetus trnV             CAAGATATAGCTTAAA--CAAAGCATCTAGTTTACACCTAGAAGATTCCAC-ACCCTGTGTATATCTTGA
Caperea marginata trnV              CAAGATATAGCTTAAA--TAAAGCATCTAGTTTACACCTAGAAGATTCCAC-GGCCTGTGCATATCTTGA
Eschrichtius robustus trnV          CAAGATATAGCTTAAA--CAAAGCATTTAGTTTACACCTAGAAGATTCCAC-AGTCCGTGTATATCTTGA
Balaenoptera acutorostrata trnV     CAAGATATAGCTTAAA--TAAAGCATCTAGTTTACACCTAGAAGATTCCAC-AACCCGTGCATATCTTGA
Balaenoptera bonaerensis trnV       CAAGATATAGCTTAAA--CAAAGCATCTAGTTTACACCTAGAAGATTTCAC-AATCCGTGCATATCTTGA
Balaenoptera physalus trnV          CAAGATATAGCTTAAA--CAAAGCATGTAGTTTACACCTAGAAGATTCCAC-AGCCCGTGTATATCTTGA
Megaptera novaeangliae trnV         CAAGATATAGCTTAAA--TAAAGCATCTAGTTTACACCTAGAAGATTACAC-AGCCCGTGCATATCTTGA
Balaenoptera musculus trnV          CAAGATATAGCTTAAA--CAAAGCATCTAGTTTACACCTAGAAGATTCCAC-AGCCCGTGCATATCTTGA
Balaenoptera omurai trnV            CAAGATATAGCTTAAA--CAAAGCATCTAGTTTACACCTAGAAGATTCCAC-AGTTCGTGTATGTCTTGA
Balaenoptera borealis trnV          CAAGATATAGCTTAAA--CAAAGCATCTAGTTTACACCTAGAAGATTCCAC-AGTCCGTGTATATCTTGA
Balaenoptera brydei trnV            CAAGATATAGCTTAAA--CAAAGCATCTAGTTTACACCTAGAAGATTCCAC-AGTCCGTGTATATCTTGA
Balaenoptera edeni trnV             CAAGATATAGCTTAAA--TAAAGCATCTAGTTTACACCTAGAAGATTCCAC-AGTCCGTGTATATCTTGA
Kogia breviceps trnV                CAAGATATAGCTTAAA--TAAAGCCCCTAGTTTACACCTAGAAGATTCCAC-AGCCCGTGTATATCTTGA
Physeter macrocephalus trnV         CAAGACATAGCTTAAA--TAAAGCATCTAGTTTACACCTAGAAGATTCCAC-AGCTCGTGTATATCTTGA
Platanista minor trnV               CAAGATATAGCTTAAA--CAAAGCATCTAGTTTACACCTAGAAGATTCCAT-CATA ATGTATATCTTGA
Ziphius cavirostris trnV            CAAGATGTAGCTTAAA--CAAAGCATCTAGTTTACACCCAGAAGATTCCAC-AGTCCGTGTACATCTTGA
Mesoplodon densirostris trnV        CAAGATATAGCTTAAA--TAAAGCATCTAGTTTACACCTAGAAGATTCCAC-AGTCCGTGTATATCTTGA
Mesoplodon europaeus trnV           CAAGATGTAGCTTAAA--TAAAGCATCTAGTTTACACCTAGAAGATTCCAC-AGTCCGTGTACATCTTGA
**Mesoplodon grayi trnV             CAAGATATAGCTTAAA--TAAAGCATCTAGTTTACACCCAGAAGATTCCAC-AGTCCGTGTATATCTTGA
Berardius bairdii trnV              CAAGGCGTAGCTTAAA--TAAAGCATCTAGTTTACACCTAGAAGATTTCAC-AGCCCGTGCACACCTTGA
Hyperoodon ampullatus trnV          CAAGATGTAGCTTAAA--CAAAGCATCTAGTTTACACCTAGAAGATTCCAC-AATCCGTGTACATCTTGA
Lipotes vexillifer trnV             CAAGATATAGCTTAAA--TAAAGCATCTAGTTTACACCTAGAAGATTCCAT-AATCAGTGTATATCTTGA
Inia geoffrensis trnV               CAAGATATAGCTTAAA--CAAAGCATCTAGTTTACACCTAGAAGATTCCAC-AATCCGTGCATATCTTGA
Pontoporia blainvillei trnV         CAAGACATAGCTTAAA--AAAAGCATTTAGC TACACCTAAAAAATCCCAC-AATCCGTGTG GTCTTGA
Monodon monoceros trnV              CAAGATGTAGCTTAAA--TAAAGCATCTAGTTTACACCTAGAAGATTTCAC-AATTCGTGCACATCTTGA
**Neophocaena asiaeorientalis trnV  CAAGATGTAGCTTAAA--CAAAGCATCTAGTTTACACCTAGAAGATTCCAC-AATTCGTGCACATCTTGA
Neophocaena phocaenoides trnV       CAAGATGTAGCTTAAA--CAAAGCATCTAGTTTACACCTAGAAGATTCCAC-AATTCGTGCACATCTTGA
Phocoena phocoena trnV              CAAGATGTAGCTTAAA--CAAAGCATCTAGTTTACACCTAGAAGATTCCAC-AACTCGTGCACATCTTGA
Cephalorhynchus heavisidii trnV     CAAGATGTAGCTTAAA--CAAAGCATCTAATTTACACCTAGAAGATTCCAC-AACTCGTGTACATCTTGA
Sousa chinensis trnV                CAAGACGTAGCTTAAA--CAAAGCATCTAGTTTACACCTAGAAGATTCCAC-AACTCGTGCACGTCTTGA
Stenella attenuata trnV             CAAGATGTAGCTTAAA--CAAAGCATCTAGTTTACACCTAGAAGATTTCAC-AACTCGTGCACATCTTGA
Tursiops australis trnV             CAAGATGTAGCTTAAA--CAAAGCATCTAGTTTACACCTAGAAGATTCCAC-AACTCGTGTACATCCTGA
Tursiops truncatus trnV             CAAGATGTAGCTTAAA--CAAAGCATCTAGTTTACACCTAGAAGATTCCAC-AACTCGTGCACATCTTGA
Tursiops aduncus trnV               CAAGATGTAGCTTAAA--CAAAGCATCTAGTTTACACCTAGAAGATTCCAC-AACTCGTGCACATCTTGA
Delphinus capensis trnV             CAAGATGTAGCTTAAA--CAAAGCATCTAGTTTACACCTAGAAGATTCCAC-AACTCGTGCACATCTTGA
Stenella coeruleoalba trnV          CAAGATGTAGCTTAAA--CAAAGCATCTAGTTTACACCTAGAAGATTCCAC-AACTCGTGCACATCTTGA
Orcaella brevirostris trnV          CAAGACGTAGCTTAAA--AAAAGCATCTAGTTTACACCTAGAAGATTCCAC-AACTCGTGTACATCTTGA
Orcaella heinsohni trnV             CAAGATGTAGCTTAAA--AAAAGCATCTAGTTTACACCTAGAAGATTCCAC-AACTCGTGTACATCTTGA
Grampus griseus trnV                CAAGATGTAGCTTAAA--CAAAGCATCTAGTTTACACCTAGAAGATTTCAC-AACTCGTGCACATCTTGA
Pseudorca crassidens trnV           CAAGATGTAGCTTAAA--CAAAGCATCTAGTTTACACCTAGAAGATTTCAC-GACTCGTGCACATCTTGA
Feresa attenuata trnV               CAAGATGTAGCTTAAA--CAAAGCATTTAGTTTACACCTAGAAGATTTCAC-AATTCGTGCACATCTTGA
Peponocephala electra trnV          CAAGATGTAGCTTAAA--CAAAGCATCTAGTTTACACCTAGAAGATTTCAC-AATTCGTGCACATCTTGA
Globicephala macrorhynchus trnV     CAAGATGTAGCTTAAA--CAAAGCATCTAGTTTACACCTAGAAGATTTCAC-AATCCGTGCACATCTTGA
Globicephala melas trnV             CAAGATGTAGCTTAAA--CAAAGCATCTAGTTTACACCTAGAAGATTTCAC-AATCCGTGCACATCTTGA
Lagenorhynchus albirostris trnV     CAAGATGTAGCTTAAA--TAAAGCATCTAGTTTACACCTAGAAGATTCCAC-AACCCGTGCACATCTTGA
Orcinus orca WNPTRU1 trnV           CAAGATGTAGCTTAAA--CAAAGCATCTAGTCTACACCTAGAAGATTCCAC-AACCCGTGTACATCTTGA
Orcinus orca ENAHN1 trnV            CAAGATGTAGCTTAAA--CAAAGCATCTAGTCTACACCTAGAAGATTCCAC-AACCCGTGTACATCTTGA
Orcinus orca AntA1 trnV             CAAGATGTAGCTTAAA--CAAAGCATCTAGTCTACACCTAGAAGATTCCAC-AACCCGTGTACATCTTGA
Orcinus orca AntB1 trnV             CAAGATGTAGCTTAAA--CAAAGCATCTAGTCTACACCTAGAAGATTCCAC-AACCCGTGTACATCTTGA
Orcinus orca AntC1 trnV             CAAGATGTAGCTTAAA--CAAAGCATCTAGTCTACACCTAGAAGATTCCAC-AACCCGTGTACATCTTGA
Orcinus orca CNPNRAL trnV           CAAGATGTAGCTTAAA--CAAAGCATCTAGTCTACACCTAGAAGATTCCAC-AACCCGTGTACATCTTGA
Orcinus orca ENPOAL2 trnV           CAAGATGTAGCTTAAA--CAAAGCATCTAGTCTACACCTAGAAGATTCCAC-AACCCGTGTACATCTTGA
                                                                                 *             *
                                    1234567  1234       4321 12345  ant  54321    1234        43217654321d
                                    0000000  1111       2222 22223  333  33444    4445        566666666667
                                    1234567  0123       1234 67890  345  89012    7890        901234567890
```

N, the most common base for the position.

N, half compensatory base change in the stem pair (e.g. T – G vs C – G; A-T vs G-T).

N, N, half compensatory base change in the stem pair exhibiting a mismatch (e.g. T–A vs A–A). Different colours are used to better differentiate the changes.

N, fully compensatory base change in the stem pair exhibiting a mismatch (e.g. C–G vs T–T).

N, type I fully compensatory base change in the stem pair (i.e. purine – pyrimidine vs purine – pyrimidine, e.g. G – C vs A – T).

N, N, type II fully compensatory base change in the stem pair (i.e. purine – pyrimidine vs pyrimidine – purine, e.g. A – T vs T – A). Different colours are used to better differentiate the changes.

N, a mismatch in the in the stem pair; N, substitution pattern not modelled; *, pair in the stem in which a mismatch is prominent. N, N, molecular signature for a determined taxa.

X, position 1-7 in the acceptor stem; X position 1-3 in the DHU stem; X position 1-5 in the anticodon stem; X position 1-4 in the TΨC stem; **ant**, anticodon; **d**, discriminator nucleotide.

trnW (**TRP**) multiple alignment

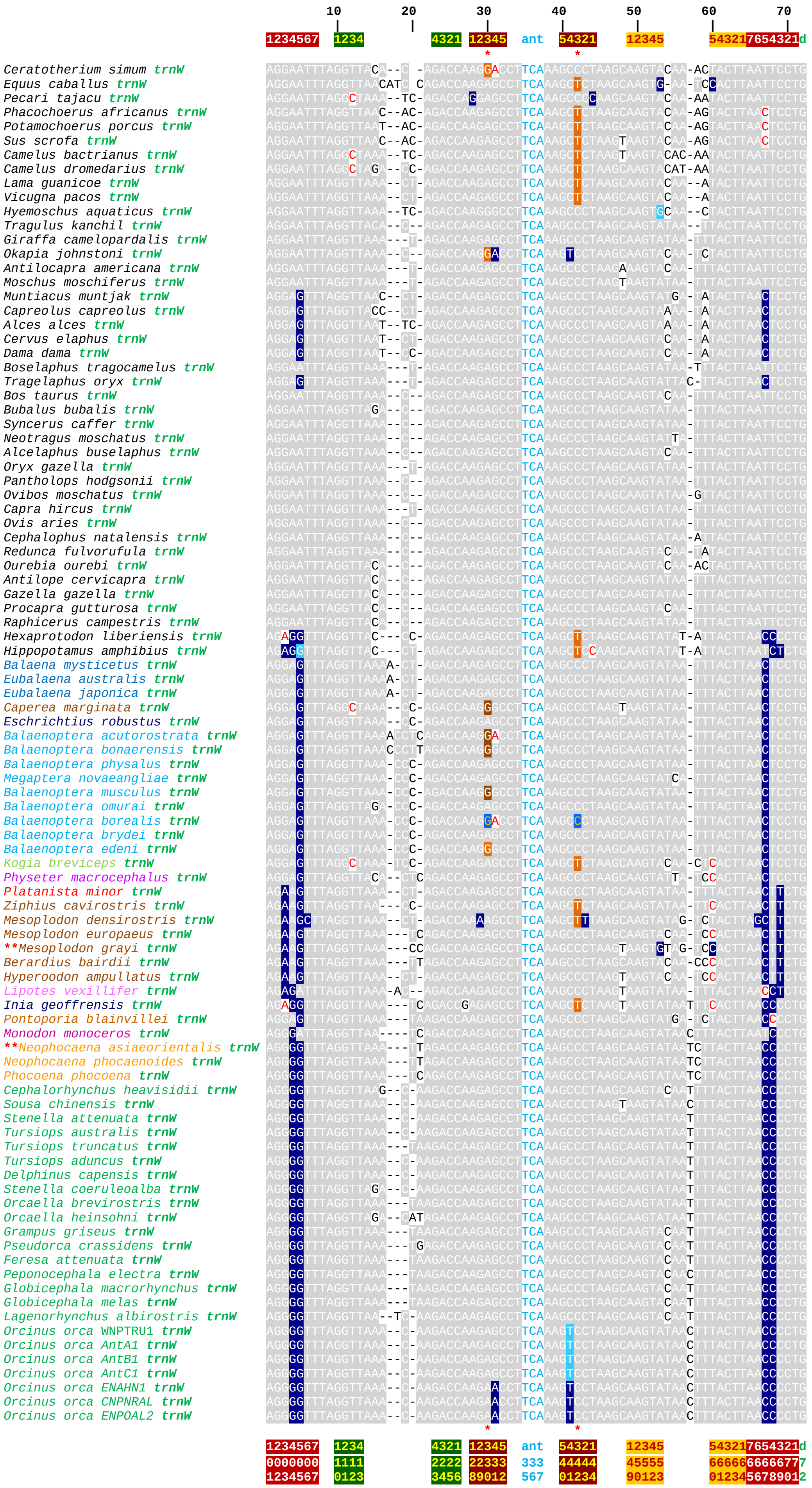

N, the most common base for the position.
N, half compensatory base change in the stem pair (e.g. T – G vs C – G; A-T vs G-T).
N, N, half compensatory base change in the stem pair exhibiting a mismatch (e.g. T–A vs A–A). Different colours are used to better differentiate the changes.
N, fully compensatory base change in the stem pair exhibiting a mismatch (e.g. C–G vs T–T).
N, type I fully compensatory base change in the stem pair (i.e. purine – pyrimidine vs purine – pyrimidine, e.g. G – C vs A – T).
N, N, type II fully compensatory base change in the stem pair (i.e. purine – pyrimidine vs pyrimidine – purine, e.g. A – T vs T – A). Different colours are used to better differentiate the changes.
N, a mismatch in the in the stem pair; N, substitution pattern not modelled; *, pair in the stem in which a mismatch is prominent. N, N, molecular signature for a determined taxa.
X, position 1-7 in the acceptor stem; X position 1-4 in the DHU stem; X position 1-5 in the anticodon stem; X position 1-5 in the TΨC stem; **ant**, anticodon; **d**, discriminator nucleotide.

trnY (TYR) multiple alignment

```
                                            10        20        30        40        50        60        70
                                             |         |         |         |         |         |         |
                                     1234567  123      321 12345  ant  54321     12345         543217654321d
Ceratotherium simum trnY             GGTAAAATGGCTGAGA--TAGCATTAGACTGTAAATCTAAAGACAGAGGTTGTA-GCCCTCTTTTTACCA
Equus caballus trnY                  GGTAAGATGGCTGAGT--AAGCATTAGACTGTAAATCTAAAGACAGGGGTTGGA-ATCCTCTTCTTATCA
Pecari tajacu trnY                   GGTAAAATGGCTGAGT--AAGCATTAGACTGTAAATCTAAAGACGGAGGTCCAAG-TCCTCTTTTTGCCA
Phacochoerus africanus trnY          GGTAAAATGGCTGAGT--AAGCATTAGACTGTAAATCTAAAGACAGAGGTTGA-G-CCCTCTTTTTGCCA
Potamochoerus porcus trnY            GGTAAAATGGCTGAGT--AAGCGTTAGACTGTAAATCTAAAGACAGAGGTTGA-G-TCCTCTTTTTACCA
Sus scrofa trnY                      GGTAAAATGGCTGAGT--AAGCATTAGACTGTAAATCTAAAGACAGAGGTTAA-G-CCCTCTTTTTGCCA
Camelus bactrianus trnY              GGTAAGATGGCCGAGT--AGGCATTAGACTGTAAATCTAAAGACAGAGGT-GAGAGCCCTCTTCTTACCA
Camelus dromedarius trnY             GGTAAGATGGCCGAGT--AGGCATTGGACTGTAAATCTAAAGACAGAGGT-GAGAGCCCTCTTCTTACCA
Lama guanicoe trnY                   GGTAAGATGGCCGAGT--AGGCATTAGACTGTAAATCTAAAGACAGAGGT-GACAACCCTCTTCTTATCA
Vicugna pacos trnY                   GGTAAGATGGCCGAGT--AGGCATTAGACTGTAAATCTAAAGACAGAGGT-GACAACCCTCTTCTTATCA
Hyemoschus aquaticus trnY            GATAGGGTGGCTGAGC--AAGCATTAGACTGTAAATCTAAAGACAGAGGTTTAAG-TCCTCTTCCTATCA
Tragulus kanchil trnY                GGTAGGATGGCCGAGT--AGGCATTAGACTGTAAATCTAAAGACAGGGGTTTTA-GTCCCCTTTCTACCA
Giraffa camelopardalis trnY          GGTAGAGTGGCTGAGT-AAAGCATTAGACTGTAAATCTAAGGACAGAGGTTTGA-TTCCTCTCTCTATCA
Okapia johnstoni trnY                GGTAAAATGGCTGAGC-ATAGCATTAGACTGTAAATCTAAAGACAGGGGTTTAA-TTCCTCTTTTTACCA
Antilocapra americana trnY           GGTAGAATGGCTGAGT-AAAGCATTAGACTGTAAATCTAAAGACAGAGGTTCGA-CCCCTCTTTTTACCA
Moschus moschiferus trnY             GGTAGAATGGCTGAGT-GAGGCATTAGACTGTAAATCTAAAGACAGAGGTTGAA-TTCCTCTTTTTACCA
Muntiacus muntjak trnY               GGTAAGATGGCTGAGT-GAAGCATTAGACTGTAAATCTAAAGATAGAGGTTTATAATCCTCTTTTTACCA
Capreolus capreolus trnY             GGTAAAATGGCTGAGT-AAAGCAATAGACTGTAAATCTAAGAACAGAGGTTTGTAATCCTCTTTTTACCA
Alces alces trnY                     GGTAAAATGGCTGAGT-GAAGCATTAGACTGTAAATCTAAAGACAGAGGTTCATACTCCTCTTTTTACCA
Cervus elaphus trnY                  GGTAGAATGGCTGAGTTGAAGCATTAGACTGTAAATCTAAAGACAGAGGTTTGTATTCCTCTTTTTACCA
Dama dama trnY                       GGTAGAATGGCTGAGT-GAAGCATTAGACTGTAAATCTAAAGACAGAGGTTTATATTCCTCTTTTTACCA
Boselaphus tragocamelus trnY         GGTAAAATGGCTGAGT-TAGGCATTAGACTGTAAATCTAAGGACAGAGGTTTGA-TTCCTCTTTTTACCA
Tragelaphus oryx trnY                GGTAAAATGGCTGAGT-AAGGCATTAGACTGTAAATCTAAGAACAGGGGTT-GA-TTCCTCTTTTTACCA
Bos taurus trnY                      GGTAAAATGGCTGAGC-AAAGCATTAGACTGTAAATCTAAAGA AGAGGTTTGA-CTCCTCTTTTTACCA
Bubalus bubalis trnY                 GGTAAAATGGCTGAGT--AAGCATTAGACTGTAAATCTAAAGACAGAGGTTTGA-GTCCTCTTTTTACCA
Syncerus caffer trnY                 GGTAAGGTGGCTGAGT--AAGCATTAGACTGTAAATCTAAAGACAGAGGTTTGA-GCCCTCTTTTTACCA
Neotragus moschatus trnY             GGTAAAATGGCTGAGT-AAAGCATTAGACTGTAAATCTAAGGACAGAGGTTGGA-TTCCTCTTTTTGCCA
Alcelaphus buselaphus trnY           GGTAAAATGGCTGAGT-GAAGCATTAGACTGTAAATCTAAGGACAGAGGTTTGA-TTCCTCTTTTTACCA
Oryx gazella trnY                    GGTAAAATGGCTGAGT-GAAGCATTAGACTGTAAATCTAAGGACAGGGGTTTAA-TTCCTCTTTTTACCA
Pantholops hodgsonii trnY            GGTAAAATGGCTGAGT-AAAGCATTAGACTGTAAATCTAAGAACAGGGGTTTGA-TTCCTCTTTTTACCA
Ovibos moschatus trnY                GGTAAAATGGCTGAGT-AAAGCAATAGACTGTAAATCTAAGAACGGAGGTTTGA-TTCCTCTTTTTACCA
Capra hircus trnY                    GGTAAAATGGCTGAGC-AAAGCAATAGACTGTAAATCTAAGAACAGAGGTTTGA-TTCCTCTTTTTACCA
Ovis aries trnY                      GGTAAAATGGCTGAGT-AAAGCAATAGACTGTAAATCTAAGAACAGAGGTTTAA-TTCCTCTTTTTACCA
Cephalophus natalensis trnY          GGTAAGATGGCTGAGT-GAAGCATTAGACTGTAAATCTAAGGACAGAGGTGAGA-TCCCTCTTTTTACCA
Redunca fulvorufula trnY             GGTAAAATGGCCGAGT-TAAGCATTAGACTGTAAATCTAAAGACAGAGGTTT-AGTCCCTCTTTTTACCA
Ourebia ourebi trnY                  GGTAAAATGGCTGAGC--AAGTATTAGACTGTAAATCTAAAGACAGAGGTTTGA-GTCCTCTTTTTACCA
Antilope cervicapra trnY             GGTAAAATGGCTGAGC-AAAGCATTAGACTGTAAATCTGAGGACAGAGGTTTGA-CTCCTCTTTTTACCA
Gazella gazella trnY                 GGTAAAATGGCTGAGT-AAAGCATTAGACTGTAAATCTAAAGACAGAGGTTTGA-CTCCTCTTTTTACCA
Procapra gutturosa trnY              GGTAAAATGGCTGAGT-AAAGCATTAGACTGTAAATCTAAGAACAGAGGTTTTA-ATCCTCTTTTTACCA
Raphicerus campestris trnY           GGTAAAATGGCTGAGT-AAAGCATTAGACTGTAAATCTAAGAACAGGGGTT-AA-TTCCTCTTTTTGCCA
Hexaprotodon liberiensis trnY        GGTAAAATGGCTGAGT--AAGCATTAGACTGTAAATCTAAGTACAGGGGTTGA-G-TCCTCTTTTTACCA
Hippopotamus amphibius trnY          GGTAAAATGGCTGAGT--AAGCATTAGACTGTAAATCTAAGTACAGGGGTTGA-G-TCCTCTTTTTGCCA
Eubalaena australis trnY             GGTAAAATGGCCGAGT--AGGCATTAGACTGTAAATCTAAAGACAGAGGTTGAAG-TCCTCTTTTTACCA
Eubalaena japonica trnY              GGTAAAATGGTCGAGT--AGGCATTAGACTGTAAATCTAAAGACAGAGGTTGAAG-TCCTCTTTTTACCA
Balaena mysticetus trnY              GGTAAAATGGTCGAGT--AGGCATTAGGCTGTAAATCTAAAGACAGAGGTTG-AG-TCCTCTTTTTACCA
Caperea marginata trnY               GGTAAAATGGCCGAGT--AGGTATTAGACTGTAAATCTAAAGACAGAGGTTG-AG-TCCTCTTTTTACCA
Eschrichtius robustus trnY           GGTAAGATGGCCGAGT--AGGTATTAGACTGTAAATCTAAAGACAGAGGTTG-AG-TCCTCTTTTTACCA
Balaenoptera acutorostrata trnY      GGTAAAATGGCCGAGT--AGGTATTAGACTGTAAATCTAAAGACAGAGGTTA-AG-TCCTCTTTTTACCA
Balaenoptera bonaerensis trnY        GGTAAAATGGCTGAGT--AAGTATTAGACTGTAAATCTAAAGACAGAGGTTG-AG-TCCTCTTTTTACCA
Balaenoptera physalus trnY           GGTAAAATGGCCGAGT--AGGTATTAGACTGTAAATCTAAAGACAGAGGTTG-AG-TCCTCTTTTTACCA
Megaptera novaeangliae trnY          GGTAAAATGGCCGAGT--AGGTATTAGACTGTAAATCTAAAGACAGAGGTTG-AG-TCCTCTTTTTACCA
Balaenoptera musculus trnY           GGTAAAATGGCCGAGT--AGGTATTAGACTGTAAATCTAAAGACAGAGGTTG-AG-TCCTCTTTTTACCA
Balaenoptera omurai trnY             GGTAAAATGGCCGAGT--AGGTATTAGACTGTAAATCTAAAGACAGAGGTTG-AG-TCCTCTTTTTACCA
Balaenoptera borealis trnY           GGTAAAATGGCCGAGT--AGGTATTAGACTGTAAATCTAAAGACAGAGGTTG-AG-TCCTCTTTTTACCA
Balaenoptera brydei trnY             GGTAAAATGGCCGAGT--AGGCATTAGACTGTAAATCTAAAGACAGAGGTTG-AG-TCCTCTTTTTGCCA
Balaenoptera edeni trnY              GGTAAAATGGCCGAGT--AGGCATTAGACTGTAAATCTAAAGACAGAGGTTG-AG-TCCTCTTTTTACCA
Kogia breviceps trnY                 GGTAAAATGGCCGAGT--AGGTATTAGACTGTAAATCTAAAGACAGAGGTTA-AG-CCCTCTTTTTGCCA
Physeter macrocephalus trnY          GGTAAGATGGCCGAGT--AGGCATTAGACTGTAAATCTAAAGACAGGGGTTA-AG-TCCTCTTTTTACCA
Platanista minor trnY                GGTAAAATGGCCGAGT--AGGCATTAGACTGTAAATCTAAAGACAGAGGT-AAAA-CCCTCTTTTTGCCA
Ziphius cavirostris trnY             GGTAAAATGGCCGAGT--AGGCATTAGACTGTAAATCTAAAGACAGAGGT-AAAG-TCCTCTTTTTGCCA
Mesoplodon densirostris trnY         GGTAAAATGGCCGAGT--AGGTATTAGACTGTAAATCTAAAGACAGAGGT-AGAG-TCCTCTTTTTGCCA
Mesoplodon europaeus trnY            GGTAAAATGGCCGAGT--AGGCATTAGACTGTAAATCTAAAGACAGGGGT-GGAG-CCCCCTTTTTGCCA
**Mesoplodon grayi trnY              GGTAAAATGGCCGAGT--AGGTATTAGACTGTAAATCTAAAGACAGAGGT-AGAG-CCCTCTTTTTGCCA
Berardius bairdii trnY               GGTAAAATGGCCGAGT--AGGCATTAGACTGTAAATCTAAAGACAGAGGT-GAAG-TCCTCTTTTTGCCA
Hyperoodon ampullatus trnY           GGTAAAATGGCCGAGT--AGGCATTAGACTGTAAATCTAAAGACAGAGGT-GGAG-TCCTCTTTTTGCCA
Lipotes vexillifer trnY              GGTAAAATGGCCGAGT--AGGTATTAGACTGTAAATCTAAAGACAGGGGT-AAAA-TCCTCTTTTTGCCA
Inia geoffrensis trnY                GGTAAAATGGCCGAGT--AGGCATTAGACTGTAAATCTAAGGACAGGGGTAGAAA-CCCCCTTTTTGCCA
Pontoporia blainvillei trnY          GGTAAAATGGCCGAGT--AGGCATTAGACTGTAAATCTAAAGACAGGGGT-GAAG-TCCTCTTTTTGCCA
Monodon monoceros trnY               GGTAAAATGGCTGAGT--AGGCATTAGACTGTAAATCTAAACCAGGGGT-AAAG-TCCTCTTTTTACCA
**Neophocaena asiaeorientalis trnY   GGTAAAATGGCCGAGT--AGGCATTAGACTGTAAATCTAAAGACAGGGGTAAAAA-TCCCCTTTTTGCCA
Neophocaena phocaenoides trnY        GGTAAAATGGCCGAGT--AGGCATTAGACTGTAAATCTAAAGACAGGGGTAAAAA-TCCCCTTTTTGCCA
Phocoena phocoena trnY               GGTAAAATGGCCGAGT--AGGCATTAGACTGTAAATCTAAAGACGGGGGTAAAAG-TCCCCTTTTTACTA
Cephalorhynchus heavisidii trnY      GGTAAAATGGTCGAGT--AGGCATTAGACTGTAAATCTAAAGACAGGGGT-AAAG-TCCCCTTTTTATCA
Sousa chinensis trnY                 GGTAAAATGGTCGAGT--AGACATTAGACTGTAAATCTAAAGACAGAGGTAAAAAATCCTCTTTTTATCA
Stenella attenuata trnY              GGTAAAATGGTCGAGT--AGGCATTAGACTGTAAATCTAAAGACAGAGGT-AGAG-TCCTCTTTTTACCA
Tursiops australis trnY              GGTAAAATGGTCGAGT--AGGCATTAGACTGTAAATCTAAAGACAGAGGT-AAAG-TCCTCTTTTTACCA
Tursiops truncatus trnY              GGTAAAATGGTCGAGT--AGGCATTAGACTGTAAATCTAAAGACAGAGGT-AAAG-TCCTCTTTTTACCA
Tursiops aduncus trnY                GGTAAAATGGTCGAGT--AGGCATTAGACTGTAAATCTAAAGACAGAGGT-AAAG-TCCTCTTTTTACCA
Delphinus capensis trnY              GGTAAAATGGTCGAGT--AGGCATTAGACTGTAAATCTAAAGACAGAGGT-AAAG-TCCTCTTTTTACCA
Stenella coeruleoalba trnY           GGTAAAATGGTCGAGT--AGGCATTAGACTGTAAATCTAAAGACAGAGGT-AAAG-TCCTCTTTTTACCA
Orcaella brevirostris trnY           GGTAAAATGGCCGAGT--AGGCATTAGACTGTAAATCTAAAGACAGAGGT-AAAG-TCCTCTTTTTGCCA
Orcaella heinsohni trnY              GGTAAAATGGCCGAGT--AGGCATTAGACTGTAAATCTAAAGACAGGGGT-AAAG-TCCTCTTTTTACCA
Grampus griseus trnY                 GGTAAAATGGCCGAGT--AGGTATTAGACTGTAAATCTAAAGACAGGGGT-GAAG-TCCTCTTTTTACCA
Pseudorca crassidens trnY            GGTAAAATGGCCGAGT--AGGCATTAGACTGTAAATCTAAAGACAGGGGT-GAAG-TCCTCTTTTTACCA
Feresa attenuata trnY                GGTAAAATGGTCGAGT--AGGCATTAGACTGTAAATCTAAAGACAGGGGT-GAAG-TCCTCTTTTTACCA
Peponocephala electra trnY           GGTAAAATGGTCGAGT--AGGCATTAGACTGTAAATCTAAAGACAGGGGT-GAAG-TCCTCTTTTTACCA
Globicephala macrorhynchus trnY      GGTAAAATGGCCGAGT--AGGCATTAGACTGTAAATCTAAAGACAGGGGT-GAAG-TCCTCTTTTTACCA
Globicephala melas trnY              GGTAAAATGGCCGAGT--AGGCATTAGACTGTAAATCTAAAGACAGGGGT-GAAG-TCCTCTTTTTACCA
Lagenorhynchus albirostris trnY      GGTAAAATGGCCGAGT--AGGCATTAGACTGTAAATCTAAAGACAGGGGTAAGAG-TCCTCTTTTTACCA
Orcinus orca WNPTRU1 trnY            GGTAAAATGGTCGAGT--AGGCATTAGACTGTAAATCTAAAGACAGGGGT-AAAG-TCCTCTTTTTACCA
Orcinus orca ENAHN1 trnY             GGTAAAATGGTCGAGT--AGGCATTAGACTGTAAATCTAAAGACAGGGGT-AAAG-TCCTCTTTTTACCA
Orcinus orca AntA1 trnY              GGTAAAATGGTCGAGT--AGGCATTAGACTGTAAATCTAAAGACAGGGGT-AAAG-TCCTCTTTTTACCA
Orcinus orca AntB1 trnY              GGTAAAATGGTCGAGT--AGGCATTAGACTGTAAATCTAAAGACAGGGGT-AAAG-TCCTCTTTTTACCA
Orcinus orca AntC1 trnY              GGTAAAATGGTCGAGT--AGGCATTAGACTGTAAATCTAAAGACAGGGGT-AAAG-TCCTCTTTTTACCA
Orcinus orca CNPNRAL trnY            GGTAAAATGGTCGAGT--AGGCATTAGACTGTAAATCTAAAGACAGGGGT-AAAG-TCCTCTTTTTACCA
Orcinus orca ENPOAL2 trnY            GGTAAAATGGTCGAGT--AGGCATTAGACTGTAAATCTAAAGACAGGGGT-AAAG-TCCTCTTTTTACCA
                                     1234567  123      321 12345  ant  54321     12345         543217654321d
                                     0000000  111      222 22222  333  33334     44444         555666666666667
                                     1234567  012      012 45678  123  67890     56789         890123456789 0
```

N, the most common base for the position.
N, half compensatory base change in the stem pair (e.g. T – G vs C – G; A-T vs G-T).
N, N, half compensatory base change in the stem pair exhibiting a mismatch (e.g. T–A vs A–A). Different colours are used to better differentiate the changes.
N, fully compensatory base change in the stem pair exhibiting a mismatch (e.g. C–G vs T–T).
N, type I fully compensatory base change in the stem pair (i.e. purine – pyrimidine vs purine – pyrimidine, e.g. G – C vs A – T).
N, N, type II fully compensatory base change in the stem pair (i.e. purine – pyrimidine vs pyrimidine – purine, e.g. A – T vs T – A). Different colours are used to better differentiate the changes.
N, a mismatch in the in the stem pair; N, substitution pattern not modelled; *, pair in the stem in which a mismatch is prominent. N, N, molecular signature for a determined taxa.
X, position 1-7 in the acceptor stem; X position 1-3 in the DHU stem; X position 1-5 in the anticodon stem; X position 1-5 in the TΨC stem; ant, anticodon; d, discriminator nucleotide.
